# RNA-seq reveals multifaceted gene expression response to Fab production in *Escherichia coli* fed-batch processes with particular focus on ribosome stalling

**DOI:** 10.1186/s12934-023-02278-w

**Published:** 2024-01-05

**Authors:** Sophie Vazulka, Matteo Schiavinato, Christopher Tauer, Martin Wagenknecht, Monika Cserjan-Puschmann, Gerald Striedner

**Affiliations:** 1 Christian Doppler Laboratory for Production of Next-Level Biopharmaceuticals in E. Coli, Department of Biotechnology, University of Natural Resources and Life Sciences, Muthgasse 18, 1190 Vienna, Austria; 2 Department of Biotechnology, Institute of Computational Biology, University of Natural Resources and Life Sciences, Muthgasse 18, 1190 Vienna, Austria; 3 Boehringer Ingelheim RCV, GmbH & Co KG, Dr.-Boehringer-Gasse 5-11, A-1120 Vienna, Austria

**Keywords:** Recombinant protein production, Periplasmic expression, Transcriptomics, Envelope stress, Ribosome stalling, Polyproline

## Abstract

**Background:**

*Escherichia coli* is a cost-effective expression system for production of antibody fragments like Fabs. Various yield improvement strategies have been applied, however, Fabs remain challenging to produce. This study aimed to characterize the gene expression response of commonly used *E. coli* strains BL21(DE3) and HMS174(DE3) to periplasmic Fab expression using RNA sequencing (RNA-seq). Two Fabs, Fabx and FTN2, fused to a post-translational translocation signal sequence, were produced in carbon-limited fed-batch cultivations.

**Results:**

Production of Fabx impeded cell growth substantially stronger than FTN2 and yields of both Fabs differed considerably. The most noticeable, common changes in Fab-producing cells suggested by our RNA-seq data concern the cell envelope. The Cpx and Psp stress responses, both connected to inner membrane integrity, were activated, presumably by recombinant protein aggregation and impairment of the Sec translocon. The data additionally suggest changes in lipopolysaccharide synthesis, adjustment of membrane permeability, and peptidoglycan maturation and remodeling. Moreover, all Fab-producing strains showed depletion of Mg^2+^, indicated by activation of the PhoQP two-component signal transduction system during the early stage and sulfur and phosphate starvation during the later stage of the process. Furthermore, our data revealed ribosome stalling, caused by the Fabx amino acid sequence, as a contributor to low Fabx yields. Increased Fabx yields were obtained by a site-specific amino acid exchange replacing the stalling sequence. Contrary to expectations, cell growth was not impacted by presence or removal of the stalling sequence. Considering ribosome rescue is a conserved mechanism, the substantial differences observed in gene expression between BL21(DE3) and HMS174(DE3) in response to ribosome stalling on the recombinant mRNA were surprising.

**Conclusions:**

Through characterization of the gene expression response to Fab production under industrially relevant cultivation conditions, we identified potential cell engineering targets. Thereby, we hope to enable rational approaches to improve cell fitness and Fab yields. Furthermore, we highlight ribosome stalling caused by the amino acid sequence of the recombinant protein as a possible challenge during recombinant protein production.

**Supplementary Information:**

The online version contains supplementary material available at 10.1186/s12934-023-02278-w.

## Background

Recombinant production of protein-based biopharmaceuticals has been established in various platforms of prokaryotic and eukaryotic origin [1]. Which host organism is used, strongly depends on the product characteristics [2]. *Escherichia coli* offers advantages due to its rapid growth and cost effectiveness. It is therefore the expression system of choice for proteins of interest (POI) like antibody fragments that do not require post-translational modifications other than disulfide bonds [3, 4]. One common format is the fragment, antigen binding (Fab). Fabs consist of a light chain (LC) and a heavy chain (HC), each of which comprises a constant (C_L_ and C_H_1) and a variable domain (V_L_ and V_H_). The variable domains contain the complementary determining regions (CDRs) which form the antigen binding site [5]. As a result, Fabs maintain the antigen binding properties of a full-length antibody. Expression of Fabs in *E. coli* often results in low protein yields due to various challenges such as misfolding, aggregation, toxicity effects, degradation and inefficient translocation to the periplasmic space, where disulfide bonds are formed [6]. Another potential hurdle during expression of recombinant proteins is ribosome stalling which has been widely attributed to the use of rare codons within the recombinant coding sequence in this context [7]. Codons common in one species might be rare in another, which hampers translation and results in low yields [8, 9]. Due to this codon usage effect, codon optimization or harmonization of the target gene sequence and overexpression of rare tRNAs are commonly used to increase expression [10–13]. However, ribosome stalling mediated by the nascent peptide chain, which is intrinsic to the amino acid sequence of the respective recombinant POI, has not been addressed in detail so far.

A vital part of gene expression in all organisms is translation of the genetic code into proteins, which is carried out by ribosomes [14]. Whereas translation initiation has been reported as a rate-limiting step, elongation has recently been recognized as an important determinant of gene expression as well [15, 16]. Translation elongation does not occur at uniform rates and is modulated by ribosomal pauses [17]. These pauses are dictated by codon usage, tRNA availability, mRNA secondary structure and the amino acid sequence of the nascent protein. During synthesis, the nascent polypeptide chain passes through a tunnel within the large ribosomal subunit. Interactions between the polypeptide chain and the tunnel can modulate the translation rate [18]. Translational pausing is implicated in gene expression regulation, and folding and targeting of the nascent protein in both eukaryotes and prokaryotes [15, 17, 19–22]. Polypeptide chains are synthesized through peptide bond formation at the peptidyl-transferase center. The amino acid proline (P) is a particularly poor peptidyl acceptor and donor, resulting in slow peptide bond formation [23–27]. Ribosome stalling at three or more consecutive proline residues (PPP) or certain diprolyl motifs (XPP/PPX) has been reported by numerous studies [17, 28, 29]. The stalling strength of diprolyl motifs differs depending on the identity of amino acid X. Glycine (G) is among the amino acids that cause strong stalling in both positions relative to the two prolines and especially PPG has been found to induce translational stalling by multiple studies [23, 30–32]. Bacteria depend on elongation factor P (EF-P) to resume translation upon transient stalling at polyproline and some diprolyl motifs [30, 31, 33–35]. To ensure functionality, EF-P is post-translationally lysinylated at the Lys34 residue by the enzymes YjeA and YjeK. Additionally, it is hydroxylated by YfcM, which is not essential for functionality [36]. Furthermore, *E. coli* relies on three distinct mechanisms to rescue terminally stalled ribosomes. The main rescue system is called t*rans*-translation and involves the small stable RNA A (SsrA) which combines tRNA and mRNA functions (and is therefore also called tmRNA), and the small protein B (SmpB). The SsrA-SmpB complex structurally mimics a tRNA and is aminoacylated with alanine. Furthermore, SsrA contains a short open reading frame (ORF) coding for a degradation tag ending with a stop codon. Thereby, the ribosome is able to switch template, the SsrA-degradation tag is added to the incompletely synthesized protein and the translating ribosome is released. Hence, *trans*-translation not only recycles stalled ribosomes, but also targets truncated proteins and faulty mRNAs for degradation [22, 37–42]. In addition, two alternative ribosome rescue factors (ArfA, ArfB) have been identified*.* ArfA recruits release factor 2 to facilitate hydrolysis of the peptidyl-tRNAs. Interestingly, ArfA itself is produced as a truncated transcript and targeted for degradation by *trans*-translation. It is therefore assumed to serve as a back-up that only accumulates when *trans*-translation capacity is overwhelmed. Double deletion of *ssrA* and *arfA* is lethal. ArfB shows intrinsic peptidyl-tRNA hydrolase activity independent of canonical termination factors. However, its biological function has not yet been fully elucidated [43–48].

In a preceding publication, we found major differences in Fab yield between 32 *E. coli* expression systems based on T7 RNA polymerase (T7 RNAP), during investigation of genome-integrated and plasmid-based expression systems producing four different Fabs [49]. The respective HCs and LCs were fused to the signal sequences of the *E. coli* protein OmpA (ompA^SS^, post-translational) for translocation to the periplasmic space. The two commonly used host strains BL21(DE3) and HMS174(DE3) were used to produce the Fabs in microbioreactor cultivations. Especially low titers were observed in all combinations for one of the Fabs, Fabx.

In the present study, we investigated Fab production in *E. coli* in an industrially relevant, C-limited fed-batch process. Four of the above-mentioned *E. coli* expression systems were selected. Strains BL21(DE3) and HMS174(DE3) with a single, genome-integrated copy of the gene of interest (GOI) were used to remove confounding plasmid-mediated effects. Two Fabs, Fabx and FTN2, were produced in lab scale, and cell growth and Fab yields were evaluated. RNA sequencing (RNA-seq) was used to determine the transcriptional response to Fab expression on a genome-wide scale, to investigate common effects of Fab expression on the host strains as well as to highlight the impact on gene expression caused by production of the challenging Fabx.

## Results

All used *E. coli* strains and expressions systems including abbreviations are listed in Table 1 To distinguish between process-related effects and direct or indirect consequences of Fab production, wild-type *E. coli* BL21(DE3) and HMS174(DE3) were included as references. The wild-type strains were cultivated using the same fed-batch process and induction strategy as the production strains, thus, also expressing T7 RNAP.

**Table 1:** *E. coli* wild-type strains and genome-integrated expression systems

Strain	Abbreviation	Source
NEB 5-alpha	–	NEB
BL21(DE3)	–	NEB
HMS174(DE3)	–	Novagen
BL21(DE3) expressing ompA^SS^-Fabx	B < oFabx >	[49]
BL21(DE3) expressing ompA^SS^-FTN2	B < oFTN2 >	[49]
HMS174(DE3) expressing ompA^SS^-Fabx	H < oFabx >	[49]
HMS174(DE3) expressing ompA^SS^-FTN2	H < oFTN2 >	[49]
BL21(DE3) expressing ompA^SS^-Fabx(P40A)	B < oFabx(P40A) >	This work
BL21(DE3) expressing ompA^SS^-FTN2(A40P)	B < oFTN2(A40P) >	This work

### Impaired cell growth of Fab-producing expression systems in fed-batch cultivations

**Fig. 1:**
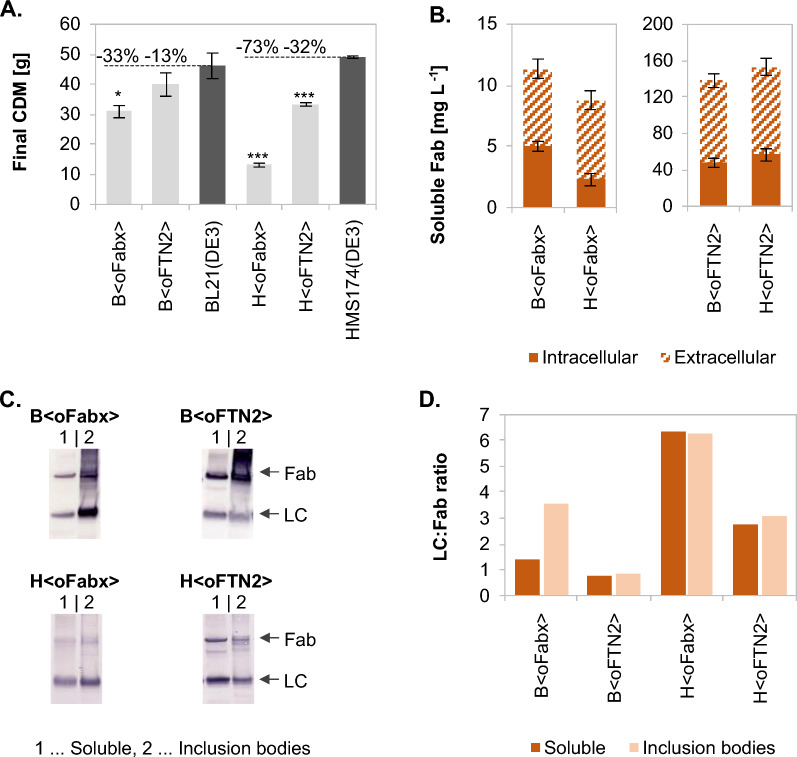
Final cell dry mass **A**, and intra- and extracellular soluble Fab yields **B** produced in different recombinant *E. coli* expression systems in fed-batch processes after 16 h of induction. Samples were analyzed in biological triplicates (n = 3, error bars represent standard error of the mean (SEM)). CDM of Fab-producing strains was compared to the respective wild-type strains BL21(DE3) and HMS174(DE3) (* … p < 0.05, ** … p < 0.01, *** p < 0.001, two sample t-test). The soluble Fab yields in the intra- and extracellular fractions were determined using ELISA. Fab expression patterns were analyzed in LC-specific WBs **C** and the ratio of LC to Fab in the soluble and IB fractions was estimated **D**

Recombinant protein expression increases the demand for cellular resources thus, exerting a metabolic burden on the host cell that can affect cell growth [50, 51]. The growth and, hence, final biomass of all Fab-producing strains was compromised to different extent compared to the wild-type references BL21(DE3) and HMS174(DE3), which reached a final cell dry mass (CDM) of 46.2 ± 4.2 g and 49.0 ± 0.4 g respectively after 16 h of induction (Fig. 1A). Biomass accumulation was impacted to different extent, depending on which Fab was produced. BL21(DE3) producing FTN2 showed 13% less final CDM than the BL21(DE3) wild-type (40.0 ± 3.9 g). B < oFabx > reached a final CDM of 30.9 ± 2.0 g, corresponding to 33% less biomass than the wild-type. The negative impact of Fabx production on growth was even more distinct in the HMS174(DE3)-based Fabx-producing strain, as 73% (13.1 ± 0.7 g) less final CDM were obtained for H < oFabx > compared to the HMS174(DE3) wild-type. FTN2 production in HMS174(DE3) led to less pronounced growth disturbances resulting in 32% less final CDM (33.4 ± 0.6 g) for H < oFTN2 > .

### Fab expression yields in fed-batch cultivations

Intracellular yields of correctly folded, soluble Fab were determined from cell extracts using Enzyme-linked immunosorbent assay (ELISA). Extracellular Fab yields were determined directly from the culture supernatant of the fed-batch cultivations using ELISA, in order to measure the amount of Fab released into the culture media (e.g. by cell lysis) as well. Fab yields are shown in Fig. 1B. The pronounced difference between Fabx and FTN2 yields determined previously in microtiter cultivations [49] was also observed in fed-batch processes. Total (intra- and extracellular) final FTN2 concentration was higher in H < oFTN2 > with 153.4 ± 15.6 mg L^−1^, followed by 136.9 ± 14.4 mg L^−1^ in B < oFTN2 > . Production of Fabx was considerably lower compared to FTN2 with 9.3 ± 0.9 mg L^−1^ in H < oFabx > and 11.4 ± 1.2 mg L^−1^ in B < oFabx > . In all Fab-producing strains, substantial amounts of Fab were found extracellularly (56–70%) in addition to the intracellular fraction. Fab located in the fermentation broth can mainly be attributed to cell lysis, as extracellular levels of Fab and DNA (measured by Hoechst dye staining) followed the same trend over the course of the cultivation (data not shown). However, additional lysis-independent leakage cannot be excluded. LC-specific western blots (WB) revealed considerable inclusion body (IB) formation in all Fab production strains (Fig. 1C). Furthermore, substantial amounts of LCs were detected, which had not been incorporated into Fab molecules. The free LCs were present in the soluble fraction as well as aggregated in IBs. The ratio of LC to Fab was estimated for the different expression systems (Fig. 1D). Fabx-LC to Fabx ratios were generally higher than the FTN2-LC to FTN2 ratios in both the soluble fractions and the insoluble IB fractions. In the HMS174(DE3)-based strains the ratio of LC to Fab was 2 – 2.6 × higher in H < oFabx > compared to H < oFTN2 > . B < oFabx > exhibited a 1.8 × higher LC to Fab ratio in the soluble fraction when compared to B < oFTN2 > and a 4.3 × higher ratio in IBs. Thus, the excess of LCs was higher in strains producing Fabx compared to FTN2-producers, which led to aggregation of LCs in IBs at rather high levels, especially in B < oFabx > .

### Gene expression response to Fab production in fed-batch cultivations

To get a comprehensive view on changes in gene expression elicited in response to Fab production, genome-wide transcription profiling was performed by means of RNA-seq. We analyzed differential gene expression (DGE) after 2 and 12 h of induction relative to the respective non-induced samples drawn immediately prior to induction. Genes that were differentially expressed in Fab-producing strains and the respective wild-type strains alike, were not considered in the following analyses, in order to exclude effects not related to Fab expression.

**Fig. 2:**
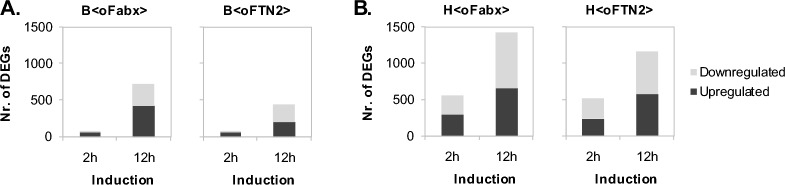
Number of up- and downregulated genes in recombinant *E. coli* BL21(DE3)- (**A**) and HMS174(DE3)-based expression systems (**B**) after 2 and 12 h of Fab production relative to the non-induced cells in fed-batch processes. Differential gene expression was analyzed using DESeq2 from biological triplicates (n = 3)

Generally, the response to Fab expression was more severe in HMS174(DE3)-based Fab-producing strains than BL21(DE3)-based ones, with respect to the number of differentially expressed genes (DEGs) (Fig. 2). In Fabx-producing strains, the number of DEGs was higher compared to the FTN2-producing counterparts. Additionally, the fraction of DEGs with a log2 fold change (log2FC) of > 1 or < − 1 was higher in the Fabx-producing strains. At the earlier tested timepoint (2 h after induction) genes with a high log2FC were among the upregulated genes, while most downregulated genes showed a rather low log2FC (Additional file 1: Figure S1).

**Fig. 3:**
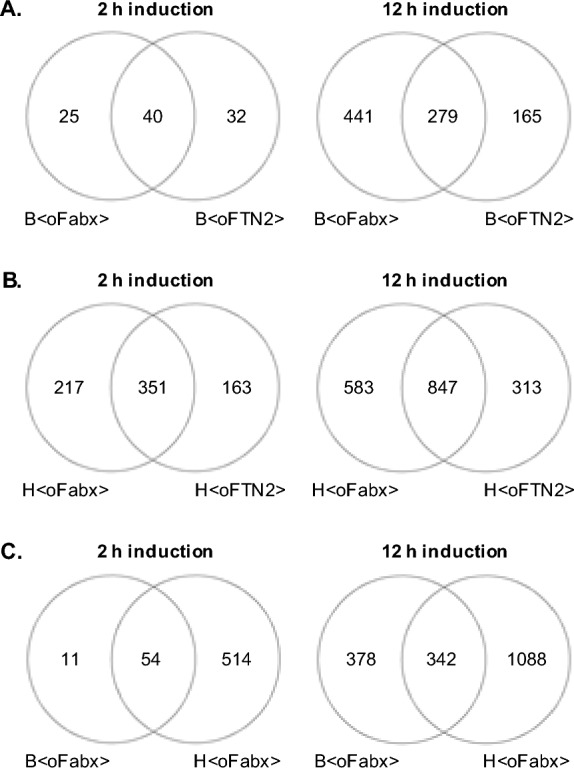
Venn diagrams of differentially expressed genes in Fab-producing *E. coli* expression systems 2 and 12 h after induction. Gene expression was compared between Fabx and FTN2 producing expression systems for BL21(DE3) (**A**) and HMS174(DE3) (**B**). Gene expression was compared between the two Fabx-producing strains (**C**). Genes also differentially expressed in wild-type BL21(DE3) and HMS174(DE3) were excluded from the analysis

The analysis aimed at showing common features in the gene expression response to production of both Fabs as well as highlight gene expression in response to the challenge of producing Fabx. Therefore, we focused first on genes differentially expressed in all expression systems producing the same Fab alike, in a strain-wise comparison in Venn diagrams (Fig. 3A and Fig. 3B). Secondly, comparison of gene expression in the three strains producing Fabx after 2 and 12 h of induction is shown in Venn diagrams in Fig. 3C.

All genes found to be differentially expressed are listed in Additional file 2: Tables S1, S3, S4, S5, S6, S7, S8. Selected genes are described and discussed in detail in the following and the discussion section. Only differentially expressed genes with a log2FC of > 0.5 or < -0.5 were considered.

### Production of Fabx causes signs of ribosome stalling

**Fig. 4:**
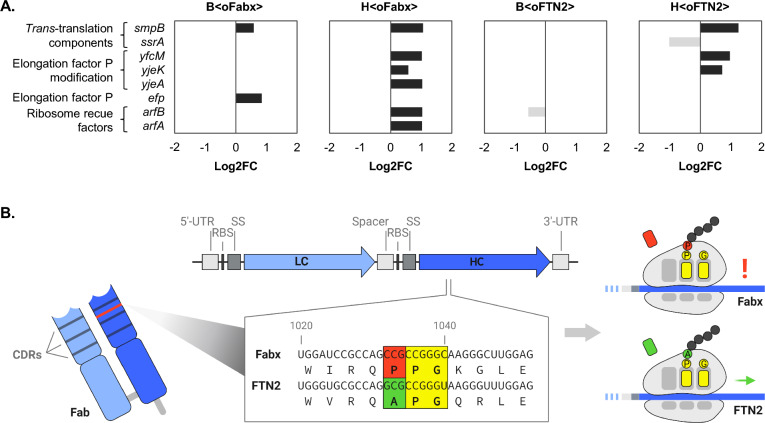
Log2FC of genes involved in ribosome stalling and rescue in Fab-producing strains after 12 h of induction relative to the respective non-induced samples (**A**). Alignment of the region of the Fabx sequence that causes ribosome stalling to the FTN2 sequence (**B**). The ribosome stalling sequence, PPG, is located in the variable domain of the Fabx-HC

Several genes coding for proteins involved in translation were differentially expressed after 12 h of induction. Notably, this group of genes included genes implicated in ribosome stalling (Fig. 4A). As the Fab sequences were codon harmonized, but some amino acid sequences are known to induce ribosome stalling, we compared the amino acid sequences of both our model Fabs. Upon alignment of the amino acid sequence of Fabx to FTN2, it became obvious that Fabx harbors a polyproline motif (PPG) within the sequence forming the variable domain of the HC (Fig. 4B). Interestingly, the response elicited differed considerably between H < oFabx > and B < oFabx > . In agreement with the generally stronger response to Fab production, H < oFabx > also showed a stronger response to ribosome stalling than B < oFabx > . Ribosome rescue factors A (*arfA*) and B (*arfB*) were upregulated in H < oFabx > , but not in B < oFabx > . On the other hand, translation elongation factor EF-P (*efp*) was upregulated in B < oFabx > . The enzymes ensuring EF-P activity by post-translational modifications (*yjeA*, *yjeK* and *yfcM*) were upregulated in H < oFabx > , but not B < oFabx > . Increased transcript levels of *yfcM* and *yjeK* were also found in H < oFTN2 > . Differential expression of *trans*-translation system components *ssrA* and *smpB* was inconclusive, as upregulation of *smpB* was observed in all strains but B < oFTN2 > , while *ssrA* was downregulated in H < oFTN2 > .

### Removal of PPG from the Fabx sequence improves Fabx yield but not cell growth

Fabx negatively impacted cell growth to a higher extent than FTN2 and obtained Fabx yields were low (Fig. 1A and Fig. 1B). As a “proof of concept”, site-specific point mutations were introduced into the nucleotide sequence of Fabx and FTN2, to investigate the effect of the stalling sequence, PPG, on cell growth and Fabx yield. Specifically, the C1032G mutation in the Fabx sequence replaced PPG with APG (P40A), while the G1032C mutation introduced the stalling sequence into FTN2 (A40P). Codon usage was considered during nucleotide exchange. The resulting constructs, Fabx(P40A) and FTN2(A40P), were fused to the post-translational ompA^SS^ and integrated into the BL21(DE3) genome. Subsequently, B < oFabx(P40A) > and B < oFTN2(A40P) > were cultivated using the same fed-batch process as described for the expression of the original Fabs as well as the same induction strategy. BL21(DE3) was chosen for the “proof of concept” experiments, as it proved to be more robust in terms of Fabx yield and cell lysis during Fabx production. Therefore, we reasoned that improvements seen in B < oFabx(P40A) > would presumably result in at least comparable improvements in the HMS174(DE3)-based counterpart.

**Fig. 5:**
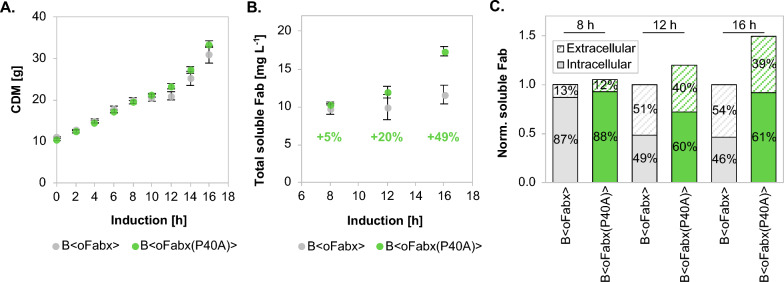
Cell growth (**A**) and Fab yields (**B**) in fed-batch cultivations of recombinant BL21(DE3)-based expression systems producing Fabx and Fabx(P40A), with and without PPG in the HC sequence, respectively. Intra- and extracellular Fabx(P40A) content as determined by ELISA, was compared to Fabx at different timepoints (**C**). Cultivations were done in triplicates (n = 3, error bars represent SEM)

Contrary to expectation, the introduction of PPG into FTN2 did not negatively affect the biomass formation of B < oFTN2(A40P) > . In fact, the strain grew identically to the comparator strain B < oFTN2 > (Additional file 1: Figure S2A). Likewise, when the stalling sequence PPG was replaced with APG in Fabx, B < oFabx(P40A) > exhibited a slight, but not statistically significant improvement in growth (Fig. 5A). In contrast to cell growth, which was not affected by removal or introduction of PPG, the amino acid sequence changes had a significant impact on Fab yield. Introduction of PPG into the FTN2 HC sequence resulted in a 41% decrease in total Fab yield of B < oFTN2(A40P) > after 16 h of production, with similar intra- to extracellular Fab ratios in both strains (Additional file 1: Figure S2B). Consistent with the findings of introducing PPG in the FTN2 sequence, elimination of PPG from the Fabx sequence resulted in enhanced Fabx production. The productivity of B < oFabx > ceased with increasing production time, resulting in a consistently low total Fabx concentration of approximately 10 mg L^−1^ after 8, 12, and 16 h of production (Fig. 5B). Unlike Fabx, Fabx(P40A) yield increased steadily during the production phase, resulting in a 49% increase in the total Fab yield after 16 h of production. B < oFabx > exhibited increasing cell lysis with progressing production time, which caused accumulation of substantial amounts of Fabx in the culture supernatant. The intracellular fraction accordingly decreased from 87 to 46% between 8 and 16 h of induction (Fig. 5C). In addition to the elevated product yield, the strain devoid of the stalling sequence exhibited a twofold increase in intracellular Fab yield, with 61% of the product located intracellularly at the end of the fermentation process.

## Discussion

In this study, we produced two industrially relevant model Fabs, Fabx and FTN2, in C-limited fed-batch cultivations employing the widely utilized *E. coli* strains BL21(DE3) and HMS174(DE3). Production of the two Fabs impacted cell growth to different degree and considerable differences in yield were observed. Our objectives were to 1) investigate the common gene expression response to Fab production in both *E. coli* strains to enable identification of cell engineering targets, and 2) shed light on the underlying reasons for the significantly lower expression of Fabx compared to FTN2.

It is noteworthy that recombinant protein production is commonly associated with a higher demand of cellular resources, which is referred to as the metabolic burden and can cause decreased cell growth [50, 51]. The presence of plasmids is a significant contributor to this metabolic pressure, as the plasmids need to be replicated, vary in copy number, can exert a high gene dosage and commonly carry resistance genes that need to be translated [52]. To alleviate this burden, we employed genome-integrated systems in place of plasmid-based ones. By doing so, we eliminated the plasmid-mediated load, enabling us to directly observe effects of Fab production.

### Gene expression response of BL21(DE3) and HMS174(DE3) to production of two model Fabs

In the following sections, we provide a more detailed description of specific aspects of the gene expression response elicited in BL21(DE3)- and HMS174(DE3)-based expression systems upon Fab production. The RNA-seq data provide fundamental insights into the effects of Fab production on transcription level and allow formulation of hypotheses regarding the physiological impact on the cells, which need to be investigated in future studies. It should be noted that the described effects can be solely attributed to production of the two model Fabs, as gene expression changes due to cultivation conditions or T7 RNAP production following induction were analyzed in the wild-type strains and were not considered here.

*Envelope stress – induction of conjugative pilus expression (Cpx) and Phage shock protein (Psp) stress responses.* The bacterial cell envelope is composed of two distinct membranes: the inner membrane (IM; a phospholipid bilayer) and the outer membrane (OM; an asymmetric structure consisting of phospholipids in the inner leaflet and lipopolysaccharides (LPS) on the outer leaflet). Enclosed within this membrane system is the periplasmic space [53]. A common theme among the gene expression data sets generated for all tested Fab-producing strains was envelope stress. Specifically, the Cpx and Psp stress responses were induced, both of which have been associated with compromised integrity of the IM [54].

The Cpx response is mediated by the CpxRA two-component system via sensor histidine kinase CpxA and response regulator CpxR. Various triggers of the Cpx stress response have been described, many with the common feature of generating misfolded proteins in the periplasm or IM. Additionally, acetyl phosphate, which is generated via the Pta-AckA pathway can phosphorylate CpxR [55]. The Cpx regulon encompasses numerous targets among which are genes coding for the periplasmic chaperones *cpxP* and *spy*, chaperone/protease *degP*, oxidoreducatase *dsbA*, peptidy-prolyl isomerase *ppiA*, IM protease *htpX* and its modulator *yccA*. After 12 h of induction, the Cpx stress response was activated in all tested Fab-producing strains. HMS174(DE3)-based strains showed an even earlier onset of the response, already after 2 h. Interestingly, in these strains, *spy* was upregulated after 2 h but not after 12 h, despite an apparent increase in the response during the production process. No upregulation of *degP* was observed in the BL21(DE3)-based strains. Additionally, the activation of the Cpx stress response resulted in the downregulation of sigma factor *rpoE* (σ^E^) transcription, which is mediated by CpxR. σ^E^ mediates another envelope stress response responsible mainly for maintenance of the OM [56]. Recombinant protein expression patterns on LC-specific WBs showed aggregated, properly assembled Fabs, as well as LCs in the IB fraction (Fig. 1). HCs were hardly detectable in HC-specific WBs (data not shown). As Fabs rely on the oxidizing environment of the periplasmic space to form disulfide bonds needed for folding, it is reasonable to assume aggregated Fabs are located in the periplasm. As the used signal sequence (ompA^SS^) mediates post-translational translocation, LCs are likely to aggregate in both the cytoplasm and the periplasm. Recombinant, rather than endogenous proteins likely represent the main part of aggregated protein in the periplasm, thereby triggering Cpx response in our study. However, additional aggregation of endogenous proteins cannot be ruled out.

The Psp response is mediated by phage shock proteins encoded by the *pspABCDE* operon, regulated by transcription factor PspF [57]. Transcription upregulation of *pspABCDE* was observed in all tested Fab-producing strains after 12 h of induction. Induction as well as response mechanisms of the Psp response are not yet completely understood. One established inducer of *psp* expression is the blockage of protein translocation across the IM. Vice versa, a functional Psp response is required for the efficient secretion of proteins which depends on the proton motive force (PMF) and ATP [58]. Depletion of YidC, an insertase associated with the Sec translocon, a dysfunctional Sec translocation machinery, and signal recognition particle (SRP) depletion have been identified as activators of the Psp response [59, 60]. T7-based expression systems as the ones used in this study were designed to induce strong expression of recombinant mRNAs [61] and translocation is a known bottleneck during production of secretory and membrane proteins during high level expression [62–64]. It has to be noted that only a single copy of the GOI was employed for Fab expression. Nevertheless, limitation of the Sec translocon seems a plausible explanation for Fab production-dependent elicitation of the Psp response and is in line with results published in other studies. Schlegel et al*.* [65] have previously described the saturation of the Sec translocon capacity resulting from periplasmic production of recombinant proteins. In a proteomics study, Wagner et al*.* [66] observed induction of the heat shock response by aggregation of mistargeted secretory proteins in the cytoplasm, inefficient ATP production through the acetate-phosphotransacetylase pathway, and downregulation of the TCA cycle as consequences of saturation of the Sec translocon capacity arising from overexpression of membrane proteins. Our transcription-level results are consistent with the proteomics findings of Wagner et al*.* In our study, we observed that all Fab-producing strains exhibited signs of heat shock, with earlier onset and a stronger response in HMS174(DE3)-based strains. Genes with increased expression levels include those coding for chaperones and proteases regulated by heat shock transcription factor σ^32^ (e.g., DnaK, DnaJ, GrpE, GroEL, GroES, ClpB, and HslVU) that counteract protein aggregation [67, 68]. Additionally, one of the highest log2FC was observed for *ibpB* in all Fab-producing expression systems after 12 h. Fabx production elicited the strongest response. We speculate that a combination of aggregated recombinant and endogenous proteins (presumably caused by translocation impairment) triggered the σ^32^ heat shock response. Furthermore, we observed similar changes in metabolic pathways as Wagner et al*.*, such as downregulation of genes involved in the TCA and glyoxylate cycles (*mdh, fumC, fumA, acnB*), and upregulation of genes involved in later glycolysis reactions (*gapA, pgk, gpmM, pykF*) as well as ATP generation through dephosphorylation of acetyl phosphate (*aceE* and *ackA*) in the HMS174(DE3)-based strains. Only *mdh*, *fumA*, *fumC*, and *gapA* were differentially expressed in all strains, genes *gpmM* and *pykF* were differentially expressed in H < oFabx > and H < oFTN2 > , and upregulation of *gapA* and *gpmM* was also observed in B < oFabx > . The observed upregulation of the acetate pathway only in HMS174(DE3)-based strains is consistent with the reported lower production of acetate in BL21(DE3) [69], even though acetate levels were not measured in our experiments. Some of the differentially expressed proteins identified by Wagner et al*.* are known to be regulated by ArcA [66]. The two-component system ArcBA is involved in gene expression regulation during the shift from aerobic to anaerobic growth. It was suggested that ArcBA may be activated by the oxidation status of the quinone pool [70]. Consequently, it was hypothesized that less efficient assembly of the respiratory chain enzymes in the IM could activate ArcBA, leading to accumulation of reduced quinones when membrane proteins are overexpressed. Our data show downregulation of members of the *nuo* operon, which encodes subunits of the NADH dehydrogenase I complex, in all strains after 12 h. Assuming this downregulation is reflected also at protein level, it might have a similar effect as less efficient assembly of the respiratory chain. In a preceding study we reported a connection between Fab production and disturbance of the cells redox balance and increased Fab yields upon ubiquinone supplementation [71]. We hypothesized that increased demand for disulfide bonds (through Fab formation) could impact the respiratory chain. Here we show that impairment of the Sec translocon presumably played a role as well. Interestingly, transcription regulator *arcA* was downregulated after 12 h of induction in both HMS174(DE3)-based strains producing Fabx. It is also worth noting that there were differences in the expression of components of the Sec translocation complex between BL21(DE3)- and HMS174(DE3)-based strains. Particularly, increased transcript levels of *secA* were observed in all Fabx-producing strains after 2 h of induction, and *secA* still showed higher transcript levels after 12 h in B < oFabx > . Transcription of *secF* and the operon containing *secY* were upregulated in both B < oFabx > and B < oFTN2 > but downregulated in H < oFabx > after 12 h. Indeed, adaption of expression of proteins involved in translocation was observed in response to different induction strengths during production of periplasmic human Growth Hormone in *E. coli* [72].To investigate the implication of differential expression of Sec translocon components on transcript level, further studies on the protein level are necessary.

Our data clearly show a negative impact of Fab expression on the IM and indicate a role of the Sec translocation capacity in the observed response. Jamming of the Sec translocon and the subsequent degradation of SecY might be responsible for the observed saturation effect. Increased SecY turnover was described by van Stelten et al*.* [73] when translation was blocked by chloramphenicol. Hence, efficiency of translation may influence saturation of the Sec translocon capacity which could in part explain why some secretory proteins seem to saturate Sec translocation capacity while others can be produced at acceptable levels in the periplasm without causing signs of Sec translocon saturation. Balancing GOI expression with the cellular protein expression machinery is an important approach for improving cell fitness and protein yields. This has been demonstrated for example in the BL21(DE3)-based LEMO21(DE3) [74] and “Walker” strains C41(DE3) and C43(DE3) [75]. These strains decrease target gene expression either via mutations in the lacUV5 promoter or by regulation of T7 RNAP activity with T7 lysozyme, an inhibitor of T7 RNAP [74]. Tuning the expression rate by L-arabinose was successfully applied for production of membrane and toxic proteins in a growth-decoupled T7 expression system [76]. In order to enhance the production of challenging secretory proteins such as Fabs, we recommend using weaker expression systems or tunable systems as described by Schuller et al*.* to decrease the expression strength and thereby, the metabolic burden caused by transcription of the recombinant gene [77, 78].

Enhancing translocation capacity was proposed as an approach to achieve higher periplasmic expression yields. YccA, for instance, prevents FtsH-mediated proteolysis of jammed Sec translocation complexes (especially SecY) [79] and overexpression of YccA, as well as introducing a mutation that renders YccA unsusceptible to FtsH degradation, have been shown to decrease toxicity of a LamB-LacZ hybrid protein [73]. Protease knockout is another strategy used in recombinant protein production. FtsH is essential, but lethality of FtsH deletion can be suppressed by the mutation sfhC21 [80]. Improved yields of an antibody fragment produced in the *E. coli* periplasm and subtilisin inhibitor in *Streptomyces lividans* were observed upon overexpression of PspA [81]. However, Vrancken et al*.* [82] reported the positive effect was dependent on the respective recombinant protein. As membrane proteins and periplasmic proteins both rely on translocation, it seems plausible, to apply strategies for the overexpression of membrane proteins also for periplasmic production.

*Alterations in the cell envelope induced by Fab production.* During both the early (2 h after induction) and later (12 h after induction) stages of the Fab production process, upregulation of multiple genes associated with the cell envelope and involved in processes such as LPS metabolism, peptidoglycan maturation, undecaprenyl phosphate (Und-P) recycling, and synthesis of osmo-regulated periplasmic glucans was observed, which is described in the following sections.

Transcription upregulation of two genes implicated in LPS metabolism was observed after 12 h of induction in all Fab-producing strains: *lpxP* coding for palmitoleoyl acyltransferase, and *lpxL* coding for lauroyl acyltransferase. *E. coli* adjusts membrane fluidity according to temperature by shifting the lipid composition in a process called “homeoviscous adaptation” [83]. LpxL mediates incorporation of laureate into lipid A, the membrane anchor of LPS, while LpxP catalyzes incorporation of palmitoleate instead of laureate. An increase in unsaturated fatty acids (UFA) is induced during cold shock to increase membrane fluidity [84, 85]. Adaption of membrane fluidity ensures maintenance of membrane functions [86]. Stimuli other than temperature have been described for increased incorporation of UFA, e.g. in biofilm forming *Pseudomonas aeruginosa* [87]. Softening and fluidization of *E. coli* BL21(DE3) cells producing the peptide human somatostatin-28 in the periplasm during a C-limited fed-batch process very similar to the one used in this study was reported by Weber et al*.* [88]. In contrast, Ami et al*.* [89] reported a decrease in membrane fluidity and permeability of BL21(DE3) due to aggregation of cytosolic recombinant protein. The effect of recombinant protein production on the lipid composition may be influenced by different factors, such as cultivation conditions, sampling timepoints, the specific protein produced, and probably also the localization of the recombinant protein. Gradual loss of membrane potential in vivo upon UFA depletion in *E. coli* has been demonstrated. Conversely, excessive membrane fluidity has been shown to induce elevated proton permeability in vitro [90]. In our study, the observed transcriptional upregulation of *lpxL* and especially *lpxP* suggests possible alterations in the LPS lipid composition of the OM. To validate this hypothesis (which, if confirmed, could clarify the results reported by Weber et al. [88]) it is essential to measure the lipid composition. The molecular trigger of *lpxL* and *lpxP* upregulation of in our study remains unknown.

Interestingly, HMS174(DE3)-based strains upregulated transcription factor FabR after 2 h of induction, along with repressed transcription of *fabA* and *fabB* at both tested timepoints. FabA and FabB are enzymes involved in the synthesis of UFA and are positively and negatively regulated by transcription factors FadR and FabR, respectively [91].

Additionally, upregulation of *lpxT* was observed in both HMS174(DE3)-based strains after 2 h of induction. Transcript levels were still increased in H < oFabx > after 12 h. LpxT activity enhances the net negative charge of LPS molecules, which mediates interactions with ions like Mg^2+^ for enhancing OM stability [92].

Transcription of several penicillin binding proteins (PBPs) was affected by Fab production, indicating a possible effect on peptidoglycan synthesis and its maturation steps in the periplasm. *E. coli* possesses several high and low molecular weight PBPs that catalyze polymerization of the glycan strand and cross-linking between glycan chains, respectively [93, 94]. Expression upregulation was observed for *mrcA* (PGP1a), *mrcB* (PGP1b), *mrdA* (PBP2), *dacB* (PGP4), *yfeW* (PBP4b), *dacA* (PGP5), *dacC* (PGP6), *dacD* (PGP6b), *pbpG* (PBP7) and *ampH*, albeit with differences depending on strain, Fab and timepoint. Impaired LPS transport could be a possible explanation for the increased levels of DD-carboxypeptidases PBP5 (DacA) and PBP6a (DacC) [95].

Moreover, increased transcript levels of *ldtC* were noted in all Fab-producing strains while upregulation of *ldtD* transcription was observed in all HMS174(DE3)-based strains after 12 h. LdtC is responsible for linking peptidoglycan to the OM by attaching it to the OM-anchored Lpp. LdtD plays a crucial role in remodeling peptidoglycan in response to cell envelope stress, as 3–3 cross-links in peptidoglycan strengthen the cell envelope in response to defects in OM assembly. Under conditions that impair LPS transport in LptC mutants, LdtD plays a critical role in preventing cell lysis [96].

Upregulation of *pgpC* transcription was observed in all Fab-producing strains after 12 h. PgpC is one of three phosphatidylglycerophosphatases involved in phospholipid synthesis that contributes about 50% of the total phosphatidylglycerophosphatase activity. PgpC generates phosphatidylglycerol (PG) some of which is further converted to carpolipin (CL) [97, 98]. PG and particularly CL contribute to translocation of proteins by stabilizing SecYEG complex, binding SecA and stimulating its ATPase activity [99, 100], which is interesting in the context of the observed saturation of the Sec translocon described in a previous paragraph.

Another indication for cell envelope alterations is the upregulation of the MarA-activated *mlaFEDCB* operon in all Fab-producing strains during the early phase of production. MlaFEDB is an IM complex involved in intermembrane phospholipid trafficking (presumably export of phospholipids to the OM) together with shuttle protein MlaC and OM complex MlaA-OmpC/F [101].

Transcription of two genes involved in synthesis of osmo-regulated periplasmic glucans (OPGs) were upregulated after 2 h of induction either in all (*opgB*) or HMS174(DE3)-based Fab-producing strains (*opgE*). Transcription of *opgB* was downregulated in HMS174(DE3)-based strains after 12 h, while *opgE* transcript levels were still upregulated in H < oFTN2 > . Synthesis of OPGs in *E. coli* increases upon growth in low osmotic strength media [102]. The accumulation of OPGs in the periplasm leads to the concentration of protons and cations in this compartment, due to their negative charge [103]. Notably, osmolarity of the cultivation media would affect the wild-type strains as well. The trigger for *opgB* and *opgE* upregulation in our study remains unclear but seems related to Fab production.

Two genes coding for major OM membrane proteins with upregulated transcript levels in all Fab-producing strains especially during the early production phase were *ompA* and *slyB*. To make the translocation capacity of the cell available for POIs, one would assume that knocking out periplasmic proteins or proteins of the OM would be attractive to free up cellular resources for translocation. However, targets must be carefully selected, since OM proteins, especially OmpA, contributes to OM stability in *E. coli* [104]. SlyB was described to contribute to OM integrity in *Burkholderia multivorans* [105]. Transcription of *slyB* is controlled by PhoP in *E. coli* [106]. All Fab-producing strains upregulated transcription of *tolQ* 12 h after induction, which encodes the IM component of the Tol-Pal system. The Tol-Pal system plays a role in OM invagination and septal peptidoglycan processing during cell division and is also implicated in OM integrity [107].

HMS174(DE3)-based Fab-producing strains exhibited transcriptional upregulation of *lnt* throughout the process. Apolipoprotein N-acyltransferase Lnt is involved in the maturation of lipoproteins thereby ensuring correct localization of lipoproteins directed towards the OM via the Lol pathway [108]. Depletion of Lnt leads to structural alterations in the cell envelope [109]. Components of the Lol lipoprotein trafficking pathway were also upregulated after 2 h in HMS174(DE3)-based expression systems and after 12 h in all Fab-producing strains.

*Potential impact on intracellular pH by impaired IM integrity* Bacteria maintain cytosolic pH homeostasis at a wide pH range. Hence, they are able to acidify or alkalinize the cytoplasm according to external pH [110]. Among the genes upregulated in all Fab-producing strains were two that respond to an increase in pH. Transcript levels of *chaA* coding for potassium/sodium:proton antiporter were upregulated in all Fab-producing strains at both tested timepoints. Increased expression of monovalent cation/proton antiporters such as ChaA is a strategy for maintaining pH homeostasis under conditions of alkaline pH by importing protons to the cytoplasm [110]. Furthermore, transcription upregulation of *alx* was observed after 2 and 12 h, which is a periplasmic protein with putative redox modulator function whose expression is mediated by high pH as well as CpxR [111, 112]. Transcription of other genes that can be impacted by alkaline pH was non-conclusive and depended strongly on the host strain used. Flagellar and chemotaxis genes, as well as *nuo* and *cyo* (all involved in processes removing protons from the cytoplasm) are described to be downregulated at high pH, while proton importing ATPase is upregulated [111]. The *nuo* operon was downregulated in all Fab-producing strains, while ATPase components were downregulated in HMS174(DE3)-strains producing Fabx, subsiding with decreased growth. During the fed-batch process, pH in the culture medium was monitored and controlled at a neutral level, accordingly. Hence, transcription regulation of the pH responsive genes cannot be connected to extracellular pH but rather must have been mediated by changes in intracellular proton concentrations or other, unknown factors.

*Starvation for essential nutrients during Fab production.* Indications for starvation for some essential nutrients were found in all Fab-producing strains: Mg^2+^ starvation during the early (2 h after induction) and phosphate and sulfur starvation during the later phase of the production process (12 h after induction). The levels of gene transcripts encoding the IM sensor histidine kinase PhoQ (*phoQ*) and transcription factor PhoP (*phoP*) were found to be upregulated in all Fab producing strains. Both proteins belong to the PhoPQ two-component signal transduction system, which can activate or repress transcription of various genes, depending on the phosphorylation state of PhoP [113]. Activation of the PhoPQ system can be triggered by several stimuli, including limitation of Mg^2+^ and Ca^2+^, antimicrobial peptides, and osmotic upshift [114]. Transcription of several genes known to be part of the PhoPQ regulon were affected in all or some of the Fab-producing strains, such as *mgtA, mgtS, mgrB, pmrD, rstA, rstB, slyB, hemL, pmrD, ompT, ompX, ychH*, *ybjX, rstB and rstA*. There might be several implications for the host cells in our process, as Mg^2+^ is involved in many cellular processes, particularly protein synthesis. Pontes et al*.* [115] established that free intracellular Mg^2+^ regulates ATP and rRNA levels in *Salmonella* and *E. coli*. Mg^2+^ depletion in mutant strains lacking an adequate response led to increased ATP levels and synthesis of translation-incompetent ribosomal subunits in their study. Hence, in conditions of low cytosolic Mg^2+^ levels, *E. coli* maintains translation homeostasis through the PhoPQ two-component system. The apparent starvation underlines the different nutrient demand under conditions of recombinant protein production. This was demonstrated by the improved recombinant protein yields of interleukin-2 upon supplementation of magnesium acetate by Sarkandy et al*.* [116]. Furthermore, Lippa and Goulian [117] reported that PhoPQ can be stimulated by a decrease in oxidizing activity in the periplasm of *E. coli*. Disulfide bond formation in the periplasm is mediated by DsbA and DsbB, and PhoPQ system activation was triggered in *dsbA* and *dsbB* mutants. As each Fab molecule requires the formation of four intra- and one interchain disulfide bond, increased production of disulfide bond-containing proteins during periplasmic Fab production could potentially act as an additional trigger of PhoPQ. No PhoPQ activation was observed after 12 h of induction, which coincides with stagnating Fab production during later stages of the process (Additional file 1: Figure S3).

Phosphorus is mainly taken up as phosphate (P_i_) and plays an important role in many cellular processes such as energy storage/transfer and signal transduction [118]. The cell responds to P_i_-limiting conditions via the PhoRB two-component system. Transcript levels of *phoR* and *phoB* as well as phosphate starvation inducible protein *psiE* were upregulated in all Fab-producing strains 12 h after induction, indicating P_i_ starvation. High affinity P_i_ transporter encoded by *pstSCAB* was upregulated in HMS174(DE3)-based strains. Surprisingly, transcription levels of *phoA* were downregulated in HMS174(DE3)-based Fabx-producing strains. This is especially interesting considering P_i_ limitation is sometimes used to induce recombinant protein expression under the control of *phoA* promotor [119–121]. Increased glucose consumption and acetate production rates in an *E. coli* K-12 strain as physiological consequences of P_i_ limitation as well as P_i_-dependency of respiratory chain activity were reported by Marzan and Shimizu [122]. Lower P_i_ led to lower cell concentrations due to decreased ATP production in their work. This has been exploited in recombinant protein production, to reallocate cellular resources from growth towards recombinant protein production [123] however, at the cost of inducing metabolic changes. Optimizing the available P_i_ levels has to be approached with care, as excessive cytoplasmic P_i_ is toxic. Upon assimilation, negative charges of P_i_ are neutralized by cations, mostly Mg^2+^.

Sulfur assimilation into the cytoplasmic cysteine pool is mediated by the Cys regulon, which includes *cysPUWAM*, *cysDNC*, *cysJIH*, and *cysK* [124]. When the preferred sulfur source, sulfate, becomes scarce, *E. coli* responds by expressing the *ssuEADCB* and *tauABCD* regulons in the absence of sulfate and cysteine, as sulfur can be assimilated from alternative sources such as taurine and alkanesulfonates [125]. Parts of the *ssu, cys* and *tau* operons were upregulated in all Fab producing strains. Furthermore, the operon *hscBA-fdx-iscX*, which includes genes involved in the machinery responsible for the biogenesis of Fe-S clusters, exhibited upregulation throughout the production process in HMS174(DE3)-based Fab-producing strains. The transcription factor IscR governs the transcription of *hscBA-fdx-iscX* and *iscRSUA* as well as the *suf* operon [126]. Interestingly, the regulation of the *isc* and *suf* operons did not coincide with that of *hscBA-fdx-iscX* under our experimental conditions, suggesting the involvement of an alternative regulatory mechanism. Depletion of sulfur could also impact translation accuracy, as sulfur modifications (among many other different modifications) are introduced post-transcriptionally in tRNAs by IscS and MnmA. Sulfur is supplied by a sulfur relay system involving TusA, TusBCD complex, and TusE [127]. Transcription of parts of the sulfur relay system was upregulated throughout the process with differences between the HMS174(DE3)-based expression systems. tRNAs undergo different modifications during maturation. Some modifications contain sulfur, like 4-thiouridine (s^4^U) which is mediated by the *thiI* gene product and is reported to promote tRNA stability and prevent their degradation [128]. The sulfur in thiouridines is derived from intracellular L-cysteine. Transcript levels of *thiI* were upregulated in all Fab-producing strains.

*Additional effects of Fab production early after induction (2 h).* Transcription of gene *tqsA* coding for a AI-2 transporter, which has been shown to control biofilm formation, was upregulated in all Fab-producing strains. It was suggested TqsA acts as an exporter of the quorum sensing signal AI-2 and decreased biofilm formation was observed in a *tsqA* knockout strain [129]. No upregulation of the enzyme producing AI-2 (LuxS) was observed however, diguanylate cyclase DgcZ showed increased transcript levels. DgcZ regulates motility and biofilm formation via the second messenger cyclic dimeric GMP (c-di-GMP) [130]. It was suggested that TqsA might also export other signalling molecules such as c-di-GMP [131]. Transcription of *dgcZ* is activated by CpxA when the Cpx stress response is triggered. Intracellular c-di-GMP concentrations are controlled by diguanylate cyclases (synthesis) and phosphodiesterases (degradation) [132]. Transcription of phosphodiesterase PdgR was upregulated mainly after 2 h of induction, while DgcZ transcript levels were still upregulated after 12 h. The implication for actual c-di-GMP levels and effects of c-di-GMP signaling cannot be deduced from transcriptome data.

The tRNA-specific adenosine deaminase encoded by the essential gene *tadA* catalyzes conversion of adenosine to inosine at the wobble position (position 34) of tRNA^Arg2^. It therefore enables recognition of three synonymous codons by a single tRNA species. tRNA^Arg2^ is the only tRNA that decodes the arginine codon CGA, which is very rare [133]. Upregulation might just be a consequence of the increased translational demand.

Transcription upregulation of the *marRAB* operon was observed after 2 and 12 h of induction in all Fab-producing strains. We have previously described upregulation of the *marRAB* operon in response to Fab production in recombinant BL21(DE3)-based strains producing different Fabs [71]. In the respective study we also observed transcription activation of *soxS* coding for a transcription factor, which has structural similarity to MarA [134]. In the present study we show transcriptional upregulation of *soxS* and *marRAB* also for the HMS174(DE3)-based Fab-producing strains. Transcription of *soxS* is activated by reactive oxygen species and we have measured increased superoxide levels in BL21(DE3)-based Fab-producing strains. In accordance with the previous study, members of the SoxS regulon were not upregulated, despite apparent activation of SoxR which is discussed in more detail elsewhere [71].

*Additional effects of Fab production after prolonged induction (12 h).* Unsurprisingly, recombinant protein expression had an impact on expression of genes connected to translation. Ribosome assembly is a complex process involving rRNAs, ribosomal proteins and accessory cofactors in vivo [135]. Transcription of *rimP* coding for a protein assisting maturation of the 30S subunit was induced in all Fab-producing strains. Other factors associated with ribosome maturation were upregulated in some of the strains and include *deaD, csdA*, *srmB, era, rimM, rimN, ybeB, yhbY,* and *yibL.* Furthermore, transcript levels of some ribosomal proteins were upregulated. rRNA synthesis is dependent on growth rate and the levels of ribosomal proteins are subject to translational feedback [136]. Due to the complexity of ribosome biogenesis, it is not possible to deduce the effect thereof on actual levels of active 70S ribosomes that contribute to translational capacity.

A general difference between HMS174(DE3)- and BL21(DE3)-based strains observed under C-limited conditions during fed-batch cultivation was different transcription of genes coding for flagellum proteins. Independently of Fab production BL21(DE3) upregulated transcription of *flgBCDEF*, *flgGHIJ, flgKL, flgMN,* and *flhABE,* which are regulated by transcription factors FlhD and FlhC. Transciption of the operon coding for the transcription factors (*flhDC*) was also upregulated. Contrary, the operons were down- rather than upregulated in HMS174(DE3), already 2 h after induction. BL21(DE3) strains with deletions in certain flagellum genes have demonstrated enhanced recombinant protein yields [137]. Knockout of *flg* genes therefore seems to be a strategy to free cellular resources for recombinant protein production, despite BL21(DE3) being non-motile due to the absence of flagellum biosynthesis genes [138].

### Characterization of the physiological impact of Fabx production on the host cells and the induced gene expression response

In our experiments, biomass formation was impaired strongest in strains producing Fabx and Fabx yields were considerably lower than FTN2. Our aim was to identify the challenges associated with Fabx production by examining the gene expression response triggered though production of this particular Fab.

DGE analysis revealed transcription activation of several genes encoding proteins involved in ribosome stalling and rescue. Translation elongation factor EF-P facilitates peptide bond formation at di- and polyprolyl motifs, including PPG and alleviates ribosome stalling at these sequences [31]. Stalled ribosomes are released either by the major ribosome rescue system, *trans*-translation (SsrA and SmpB), or one of two ribosome rescue factors, ArfA and ArfB [43, 46]. Enhanced transcription of *efp*, *arfA*, and *arfB*, along with increased transcript levels of EF-P-activating enzymes (*yjeA*, *yjeK*, and *yfcM*) prompted us to take a closer look at the amino acid sequences of the Fabs. We concluded that the PPG sequence located in the Fabx HC likely induces peptide-mediated ribosome stalling.

*BL21(DE3) and HMS174(DE3) react differently to ribosome stalling on Fabx mRNA.* The gene expression response to ribosome stalling on Fabx mRNA differed substantially in BL21(DE3)- and HMS174(DE3)-based expression systems. While B < oFabx > exhibited upregulation only of *efp* transcription, H < oFabx > showed upregulation of transcript levels of *yjeA*, *yjeK*, *yfcM*, *smpB*, *arfA*, and *arfB*. Downregulation of *ssrA* transcript levels in H < oFTN2 > was unexpected but might be attributed to an interdependency with SmpB. Moore et al*.* [139] found expression of *ssrA* from a plasmid only significantly increased above wild-type SsrA levels in combination with SmpB, indicating stabilization of SsrA by SmpB. Depletion of SmpB during FTN2 production could possibly lead to lower SsrA levels. It was estimated that during exponential growth, only 0.4 – 0.5% of proteins are tagged for degradation by *trans*-translation. Thus, *trans*-translation seems to operate below capacity [140, 141]. Nevertheless, on average each ribosome is rescued by *trans*-translation once per cell cycle and impairment of translation can be assumed in case these ribosomes are not released [139]. ArfA and ArfB are thought to act as backup rescue systems when *trans*-translation becomes saturated in *E. coli* [43]. Recombinant Fabx and FNT2 mRNAs were among the most abundant mRNAs in the cell (data not shown). Stalling on Fabx mRNA could have exceeded both EF-P and *trans*-translation capacities, necessitating activation of the alternative rescue factors in H < oFabx > . Utilization of modified versions of SsrA, which add tags other than the naturally added ssrA degradation tag [139, 142], could be useful in investigating whether *trans*-translation indeed acts on Fabx mRNA. Thereby, instead of targeting Fabx for degradation by ClpXP/ClpAP or Tsp proteases [143], detection of tagged Fabx HC would be possible. *Trans*-translation activity is also increased upon depletion of tRNAs and release factors that enable ribosome recycling [141]. Monitoring tagging by *trans*-translation in H < oFTN2 > would be compelling, as H < oFTN2 > also differentially expressed *ssrA* and *smpB* after 12 h of induction. Increased tagging could indicate depletion of translational components as a general implication of recombinant protein overexpression. Additional proteomic analysis would provide a more comprehensive understanding of the observed gene expression response and the actual effects thereof on the cell.

Considering there are some genotypic differences between BL21(DE3) and HMS174(DE3), differences in gene expression response elicited by these two widely used strains are to be expected. A comparison by Studier et al*.* [144] reports alignment of the basic genomes of selected B and K strains with ~ 99% bp identity over ~ 92% of their genomes, with more than half of the protein coding sequences being identical. Present divergences lead to phenotypical differences e.g. in the presence of proteases, in acetate metabolism or motility [145]. The observed dissimilarity in gene expression response to ribosome stalling was striking, considering that ribosome rescue is such a vital process.

*Ribosome stalling on Fabx mRNA impacted envelope stress responses and protein aggregation.* Production of Fabx induced earlier elicitation of membrane stress responses Cpx and Psp. In B < oFabx > , the Cpx stress response was activated 2 h after induction, indicating that protein aggregation in the periplasm occurred earlier than in B < oFTN2 > . This is probably caused by less efficient translation of HCs due to stalling, leading to an excess of LCs, which are more prone to aggregation. This is supported by WB analysis, which revealed a higher LC:Fab ratio in IBs of B < oFabx > than in B < oFTN2 > and a higher LC:Fab ratio in both the soluble and IB fractions of H < oFabx > , compared to H < oFTN2 > (Fig. 1C). Ribosome stalling on Fabx mRNA appeared to contribute to early activation of the Psp response. Upregulation was observed in B < oFabx > and H < oFabx > , but not in B < oFTN2 > and H < oFTN2 > after 2 h. A connection between ribosome release and resistance to envelope stress was also found by Hobbs et al*.* [146] in a *ssrA* deletion strain. Furthermore, stalled translation has been shown to promote misfolding of nascent polypeptide chains [147], potentially resulting in increased levels of aggregated recombinant proteins in strains producing Fabx shortly after induction. Notably, transcript levels of heat shock proteins, including HslVU protease and ClpB, were upregulated earliest in B < oFabx > and H < oFabx > . Additional upregulation of the small heat shock proteins IbpA and IbpB suggests higher levels of cytoplasmic aggregation in Fabx-producing strains after 2 h. IbpA and IbpB work in tandem to promote disaggregation of protein aggregates through the action of chaperones [148].

*Ribosome stalling significantly impacted recombinant product yield but not growth of BL21(DE3) in a fed-batch process.* In wild-type *E. coli*, translation of proteins that include di- and polyprolyl motifs is facilitated by the action of EF-P, which enhances peptidyl transferase activity of the ribosome [149]. Ribosome profiling data indicate accumulation of ribosomes at polyproline or XPP/PPX motifs even when EF-P is present [29, 31], however most stalls on intact mRNAs are short-lived [46]. Increased stalling at PPP and XPP/PPX motifs is observed in Δ*efp* mutants [35]. Estimates of the number of EF-P molecules in the cell differ. An et al*.* reported rather low copy numbers of ca. 0.1 per ribosome [150]. However, ribosomes and EF-P form a complex at a ratio of 1:1 in vitro [151]. Since T7-based expression results in high gene dosage [61], we hypothesized that translational stalling at PPG on the recombinant Fabx mRNA might strain the cells capacity to alleviate stalling. Consequently, this would lead to ribosomes being retained on the highly abundant recombinant mRNA, making them unavailable for new cycles of translation. Thus, stalling could cause lower global translation efficiency and limitation of the cell’s translation machinery. If free ribosomes are limited, strong ribosome binding sites like the one used for expression of Fabx have an advantage competing for available ribosomes [50], whereby translation of endogenous proteins could potentially become restrained [79, 152]. Therefore, we anticipated high-level expression of a recombinant protein containing a ribosome stalling sequence to cause impairment of endogenous protein homeostasis and hence reduced growth. Contrary to our expectations, cell growth was not significantly impacted by removal or introduction of the stalling sequence PPG into the recombinant protein sequence during fed-batch production (Fig. 5, Additional file 1: Figure S3). There are two possible explanations. It was demonstrated by Tollerson et al*.* that EF-P-relieved ribosome pausing at PPX motifs is growth rate-dependent [79]. Therefore, the effect of ribosome stalling in the absence of EF-P is probably masked by slow growth rates such as 0.1 h^−1^ used in our study. Furthermore, ribosomes stalled on Fabx mRNA could have been released by one of the three ribosome rescue mechanisms, *trans*-translation, ArfA and ArfB at sufficiently high rates to support the set growth rate of 0.1 h^−1^. Even though cell growth was not negatively impacted, cell fitness was improved upon removal of PPG from the Fabx sequence, as less cell lysis and consequently higher intracellular Fabx yields were observed. As anticipated, removal or introduction of the ribosome stalling sequence had a significant impact on Fab yields, as Fabx yield could be improved up to 49%. The replacement of PPG within the Fabx sequence effectively demonstrated the higher translation efficiency in the absence of the ribosome stalling sequence. The yield remained rather low indicating additional, yet unidentified challenges in Fabx expression. Nevertheless, our data demonstrate the importance of the amino acid sequence of a POI for its efficient expression. While ribosome stalling due to rare codons is frequently addressed, peptide-mediated ribosome stalling has been largely overlooked in the context of recombinant protein production. This is surprising, considering that the human genome encodes for > 7000 proteins that contain PPP, and > 15,000 proteins that contain XPP/PPX, respectively [30], including many potential biopharmaceutical targets. Generally, changes in POI amino acid sequence should be carefully considered as the amino acid sequence impacts protein folding, structure and function [153] and the impact on Fabx functionality was not tested within this study.

## Conclusion

We investigated the impact of producing two different Fabs, Fabx and FTN2, on cell growth and recombinant protein yield in industrially relevant C-limited fed-batch processes. RNA-seq was used to determine the transcriptional response to Fab production on a genome-wide scale. Our data indicate various effects of recombinant Fab production on the host cells. Most noticeable was the impact on IM and cell envelope. Induction of the Cpx and Psp envelope stress responses was presumably caused by aggregation of the recombinant and probably also endogenous proteins and impairment of the Sec translocation machinery, respectively. The observed gene expression suggests alterations in the envelope such as changes in LPS synthesis, adjustment of membrane permeability, peptidoglycan maturation and/or remodeling, and production of osmo-regulated periplasmic glucans. Furthermore, signs of changes in intracellular pH were observed, which might be connected to impairment of the IM. Furthermore, apparent Fab production-dependent depletion of some essential nutrients was observed. Activation of the PhoPQ two-component signal transduction system indicated Mg^2+^ starvation early after induction and, in addition, signs of sulfur and phophate starvation were observed during later stages of the process. This highlights the altered requirements of cells under conditions of recombinant protein production. Through our comprehensive approach we aimed at providing a basis for future studies, to get a more complete understanding of implications of Fab production for the host cells. Thereby, we identified potential gene targets to enable more targeted cell engineering approaches for improving cell fitness and Fab yields. These gene targets will be verified in future experiments.

In contrast to many well described challenges in recombinant protein production, the role of ribosome stalling caused by the nascent peptide has been largely overlooked so far. In a biopharmaceutical context, not only specificity and functionality of a target protein, but also manufacturability play a role during research and development. Hence, consideration should be given not only to codon usage, but also amino acid sequence properties of a POI during protein design.

The two commonly used *E. coli* strains BL21(DE3) and HMS174(DE3) differ in certain aspects of cellular metabolism and physiology. Accordingly, differences in gene expression response were observed during Fab production. Remarkably, the two strains also showed a different gene expression response to such a pivotal process as ribosome stalling during production of Fabx. Simply put, HMS174(DE3) provided a stronger gene expression response under our study conditions, rendering it a more rewarding strain for investigations into gene expression, while BL21(DE3) proved to be more robust.

## Methods

### Strains and model proteins

All genome integrated *E. coli* expression systems used within the study originated from BL21(DE3) (NEB, USA) and HMS174(DE3) (Novagen, USA) and are listed in Table 1 along with the respective wild-type strains. Genome integration is indicated by “ <  > ” in the strain abbreviations. Two therapeutic Fabs served as model proteins: FTN2, a humanized Fab, that specifically binds to and neutralizes tumor necrosis factor α and Fabx, which is specific to antigen x (not described in more detail due to confidentiality issues). Fab LCs and HCs were fused to the post-translational translocation signal sequence of *E. coli* protein OmpA. The Fab sequences were codon harmonized [49]. Single Fab gene copies were genome integrated and expressed from bicistronic constructs with a ribosome binding site for both LC and HC. Generation of the Fabx and FTN2 expression systems is described in detail by Fink et al*.* [49]. For cloning of B < oFabx(P40A) > and B < oFTN2(A40P) > , pET30acer vectors containing the Fabx and FTN2 gene constructs [49] were amplified using the primer pairs Fabx(P40A)_sense and Fabx(P40A)_antisense, and FTN2(P40A)_sense and FTN2(P40A)_antisense (purchased from Sigma Aldrich, USA), respectively. Thereby, point mutations were introduced to exchange the Pro at position 40 for Arg in the case of Fabx (exchange of CCG to GCG) and Arg for Pro in the case of FTN2 (exchange of GCG to CCG) to replace the ribosome stalling sequence PPG with APG in the Fabx sequence and vice versa in FTN2. The cloning strain NEB5alpha was transformed with the re-ligated vectors. The vectors were purified, and the integration cassette was amplified using primers TN7_HO1 and TN7_HO2 (purchased from IDT, USA). All primers used during cloning of B < oFabx(P40A) > and B < oFTN2(A40P) > are listed in Table 2. Genome integration was done at the attTN7 site of *E. coli* BL21(DE3) and was executed according to the antibiotic free integration method described by Egger et al*.* [154]. Enzymes and kits used to generate the expression systems were purchased from NEB, USA. Sanger sequencing (Microsynth AG, Switzerland) was used to confirm the correct DNA sequences of all constructs.

**Table 2:** Primer pairs used during cloning of B  < oFabx(P40A) >and B <oFTN2(A40P)>

Primer	Sequence
Fabx(P40A)_sense	5ʹ—GCGCCGGGCAAGGGC—3ʹ
Fabx(P40A)_antisense	5ʹ—CTGGCGGATCCAATCGACG-3ʹ
FTN2(P40A)_sense	5ʹ—CCGCCGGGTAAGGGTTT-3’
FTN2(P40A)_antisense	5ʹ—CTGGCGCACCCAGTT-3’
TN7_HO1	5ʹ—AGATGACGGTTTGTCACATGGAGTTGGCAGGATGTTTGATTAAAAACATAGTAGTAGGTTGAGGCCGTTG—3'
TN7_HO2	5ʹ—CAGCCGCGTAACCTGGCAAAATCGGTTACGGTTGAGTAATAAATGGATGCCGGATATAGTTCCTCCTTTCAG—3ʹ

The regions of the sequences introducing point mutations are underlined

### Cultivation conditions

Master and working cell banks were prepared for all strains as described elsewhere [71]. Working cell banks were used for preparation of the inoculum for fed-batch cultivations. Fed-batch cultivations were conducted in the DASGIP Parallel Bioreactor System (Eppendorf AG, Germany) with 0.6 L batch volume (6 g final CDM) and 1.2 L final working volume (40 g theoretical CDM). The temperature was controlled at 37 ± 0.5 °C during the batch phase und was decreased to 30 ± 0.5 °C at feed start. The pH was maintained at 7 ± 0.05 by addition of 12.5% ammonia solution (w w^−1^). Dissolved oxygen (DO) was regulated via the stirrer speed, aeration rate and ingas composition. The growth rate was set to 0.1 h^−1^ via the glucose-limited feed. 3 h into the feed phase, Fab production was induced with 2 µmol IPTG g^−1^ CDM. PPG200 antifoam (BASF, Germany) was added on demand to prevent foaming. The process, cultivation media, as well as gravimetric determination of CDM are described in detail by Vazulka et al*.* [71]. Samples corresponding to 1 mg CDM were drawn for qualitative and quantitative analysis of Fab expression. The cells were pelleted (13,000 ×*g* for 10 min at 4 °C) and frozen at -20 °C. RNA-seq samples were drawn 3 h after feed start, immediately before induction (0 h), and after 2, 12, and 16 h of induction. The cell suspension was transferred to 0.5 × the volume of a 5% phenol in ethanol solution on ice. Aliquots of 3 mg CDM were pelleted (13,000 ×*g* for 2 min at 4 °C) and frozen at − 80 °C. A simplified illustration of the experimental setup is depicted in Additional file 1: Figure S5.

### Fab analytics

Fab expression was analyzed qualitatively by WB and quantitatively by enzyme-linked immunosorbent assay (ELISA). Cell lysates were prepared by enzymatical disruption of the cells as described by [49].

*Fab WB*. Fab expression in the soluble and IB factions of cell lysates was analyzed using anti‐human κ‐LC (bound and free) goat antibody, conjugated to alkaline phosphatase (A3813; Sigma‐Aldrich, USA) in LC-specific WBs as described by [49]. The ratio of LC to Fab was estimated using ImageQuantTL (Version 7.0) analysis software.

*Fab ELISA.* Soluble, correctly folded Fab was quantified from cell lysates and the culture supernatant via sandwich ELISA, using anti-human IgG (Fab-specific) goat antibody (I5260; Sigma-Aldrich, USA), anti-human IgG mouse antibody [2A11] (ab7497; Abcam, UK), and anti-mouse IgG (Fab-specific) goat antibody conjugated to peroxidase (A2304; Sigma-Aldrich, USA) as described elsewhere [49].

### Gene expression analysis

*RNA extraction.* Cells were disrupted and RNA extracted using the Direct-zol RNA Miniprep Kit (Zymo Research, USA) as described by [71]. RNA integrity (RIN > 8) and absence of genomic DNA in extracted RNA samples were analyzed using an Agilent 21,000 Bioanalyzer in combination with the RNA 6000 Nano Kit (Agilent Technologies, USA) according to manufacturers’ instructions and stored at − 80 °C.

*RNA-seq library preparation and sequencing.* rRNA depletion of the RNA samples, preparation of RNA-seq libraries and sequencing in single-read mode on a HiSeq 2500 system (Illumina, USA) were performed by the Next Generation Sequencing Facility at Vienna BioCenter Core Facilities (VBCF), member of the Vienna BioCenter (VBC), Austria.

*Pre-processing and mapping of sequencing reads.* The pre-processing of RNA-seq raw reads and read mapping onto the reference genomes is described in detail by [71]. FastQC [155] and MultiQC [156] were used for quality check of the RNA-seq reads. Trimming of the adaptor sequences and removal of low-quality reads was done with Trimmomatic v0.38 [157]. HISAT2 [158] was used for mapping of the processed reads onto the respective reference genomes. The reference genome of *E. coli* BL21(DE3) had been previously determined in-house [159]. As a reference genome for HMS174(DE3) was not available, the sequenced genome of HMS174 (NCBI accession LM993812) described by Mairhofer et al*.* [160] was used for mapping of reads originating from HMS174(DE3)-based strains.

*DGE analysis.* DGE analysis is described in detail by [71]*.* Read counts per gene were determined with HTSeq [161]. DESeq2 [162] was used for differential gene expression analysis. The change in gene expression was computed by comparing samples drawn after 2 and 12 h of induction against the non-induced sample (0 h) for each expression system or wild-type strain. The end point sample (16 h) was not included within this work. The samples of the 12 h induced cultures were drawn after onset of growth disturbances and lysis and should therefore sufficiently cover relevant changes connected to cell death.

### Supplementary Information

**Additional file 1: Figure S1.** Fabx and FTN2 mRNA levels [tpm] prior to and after 2, 12 and 16 h of induction in C-limited fed-batch processes of recombinant BL21(DE3)- **A** and HMS174(DE3)-based expression systems **B**. Biological triplicates were analyzed (n=3). **Figure S2.** Volcano plots of differentially expressed genes in BL21(DE3)- and HMS174(DE3)-based expression systems producing Fabx **A** and FTN2 **B** in C-limited fed-batch processes after 2 and 12 h of induction relative to the respective non-induced control. DGE was determined with DESeq2. Genes also differentially expressed in the respective wild-types were excluded. Biological triplicates were analyzed (n=3). **Figure S3.** Cell growth **A** and Fab yields **B** of two recombinant *E. coli* expression systems producing ompA^SS^-FTN2 and ompA^SS^-FTN2(A40P) in C-limited fed-batch processes for B<oFTN2> (n=3) and for B<oFTN2(A40P)> (n=1). **Figure S4.** Specific soluble Fab yields (intracellular and extracellular fractions) [mg g^−1^] of Fabx **A** and FTN2 **B** produced in C-limited fed-batch cultivations. Cultivations were done in triplicates Fab yields at the different time points were analysed from two to three biological replicates (n=2 or n=3). **Figure S5.** Simplified illustration of the experimental setup. Two Fabs (Fabx and FTN2) were produced in genome-integrated *E.* *coli* strains BL21(DE3) and HMS174(DE3) in C-limited fed-batch cultivations in the DASGIP parallel bioreactor system in lab scale. CDM [g], Fab expression patterns and Fab yields [mg L^−1^] were determined from samples drawn at different time points during the process. Different expression after 2 and 12 h of induction was analysed relative to the non-induced samples by means of RNA-seq.

**Additional file 2: Table S1.** Differentially expressed genes in B<oFabx> during fed-batch cultivation after 2 h of induction relative to the sample drawn immediately before induction of Fabx expression. Genes also differentially expressed in wild-type BL21(DE3) were excluded. **Table S2.** Differentially expressed genes in B<oFTN2> during fed-batch cultivation after 2 h of induction relative to the sample drawn immediately before induction of FTN2 expression. Genes also differentially expressed in wild-type BL21(DE3) were excluded. **Table S3.** Differentially expressed genes in B<oFabx> during fed-batch cultivation after 12 h of induction relative to the sample drawn immediately before induction of Fabx expression. Genes also differentially expressed in wild-type BL21(DE3) were excluded. **Table S4.** Differentially expressed genes in B<oFTN2> during fed-batch cultivation after 12 h of induction relative to the sample drawn immediately before induction of FTN2 expression. Genes also differentially expressed in wild-type BL21(DE3) were excluded. **Table S5.** Differentially expressed genes in H<oFabx> during fed-batch cultivation after 2 h of induction relative to the sample drawn immediately before induction of Fabx expression. Genes also differentially expressed in wild-type HMS174(DE3) were excluded. **Table S6.** Differentially expressed genes in H<oFTN2> during fed-batch cultivation after 2 h of induction relative to the sample drawn immediately before induction of FTN2 expression. Genes also differentially expressed in wild-type HMS174(DE3) were excluded. **Table S7.** Differentially expressed genes in H<oFabx> during fed-batch cultivation after 12 h of induction relative to the sample drawn immediately before induction of Fabx expression. Genes also differentially expressed in wild-type HMS174(DE3) were excluded. **Table S8.** Differentially expressed genes in H<oFTN2> during fed-batch cultivation after 12 h of induction relative to the sample drawn immediately before induction of FTN2 expression. Genes also differentially expressed in wild-type HMS174(DE3) were excluded.

## Data Availability

Gene expression data sets generated by RNA-seq are deposited under the SRA BioProject PRJNA750301.
