# Supplementary material for: RNA-seq reveals multifaceted gene expression response to Fab production in Escherichia coli fed-batch processes with particular focus on ribosome stalling
Source: Microb Cell Fact. 2024 Jan 5;23:14. doi: 10.1186/s12934-023-02278-w (PMC10768439; doi:10.1186/s12934-023-02278-w)

Additional file materials

RNA-seq reveals multifaceted gene expression response to Fab production in *Escherichia coli* fed-batch processes with particular focus on ribosome stalling

Sophie Vazulka[1], Matteo Schiavinato[2], Christopher Tauer[1], Martin Wagenknecht[3], Monika Cserjan-Puschmann[1*], Gerald Striedner[1]

[1]Christian Doppler Laboratory for production of next-level biopharmaceuticals in *E. coli*, Department of Biotechnology, University of Natural Resources and Life Sciences, Muthgasse 18, A-1190 Vienna, Austria

[2]Department of Biotechnology, Institute of Computational Biology, University of Natural Resources and Life Sciences, Muthgasse 18, A-1190 Vienna, Austria

[3]Boehringer Ingelheim RCV GmbH & Co KG, Dr.-Boehringer-Gasse 5-11, A-1120 Wien

* Corresponding Author: Email: monika.cserjan@boku.ac.at

Figure S1. Fabx and FTN2 mRNA levels [tpm] prior to and after 2, 12 and 16 hours of induction in C-limited fed-batch processes of recombinant BL21(DE3)- (A) and HMS174(DE3)-based expression systems (B). Biological triplicates were analyzed (n = 3).

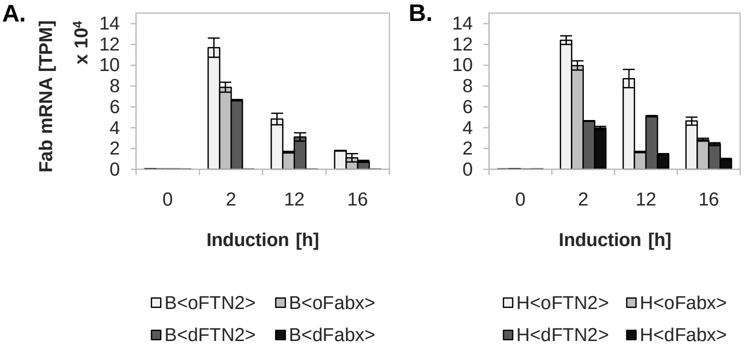

Figure S2. Volcano plots of differentially expressed genes in BL21(DE3)- and HMS174(DE3)-based expression systems producing Fabx (A) and FTN2 (B) in C-limited fed batch processes after 2 and 12 h of induction relative to the respective non-induced control. DGE was determined with DESeq2. Genes also differentially expressed in the respective wildtypes were excluded. Biological triplicates were analyzed (n = 3).

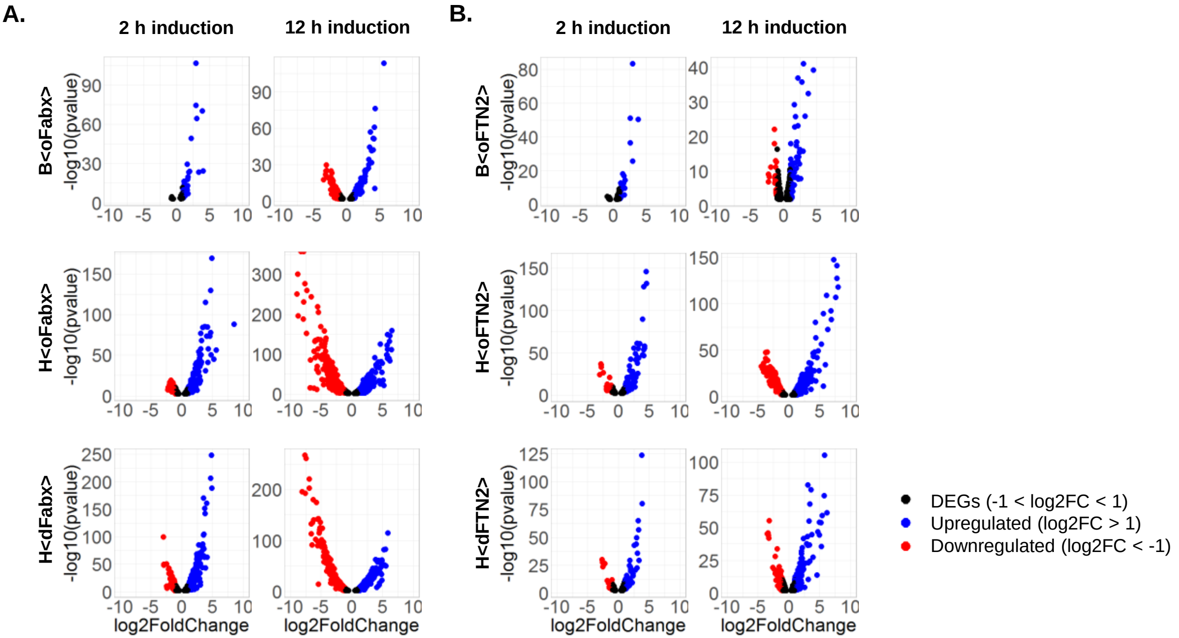

Figure S3. Cell growth (A) and Fab yields (B) of two recombinant *E. coli* expression systems producing ompA^{SS}-FTN2 and ompA^{SS}-FTN2(A40P) in C-limited fed-batch processes (n = 3 for B<oFTN2>, n = 1 for B<oFTN2(A40P)>).

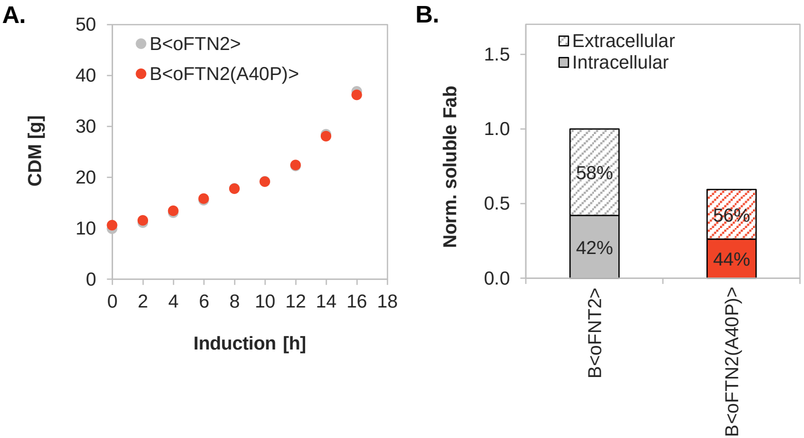

Figure S4. Specific soluble Fab yields (intracellular and extracellular fractions) [mg g^{-1}] of Fabx (A) and FTN2 (B) produced in C-limited fed-batch cultivations. Cultivations were done in triplicates, Fab yields at the different timepoints were analyzed from two to three biological replicates (n = 2 or n = 3).

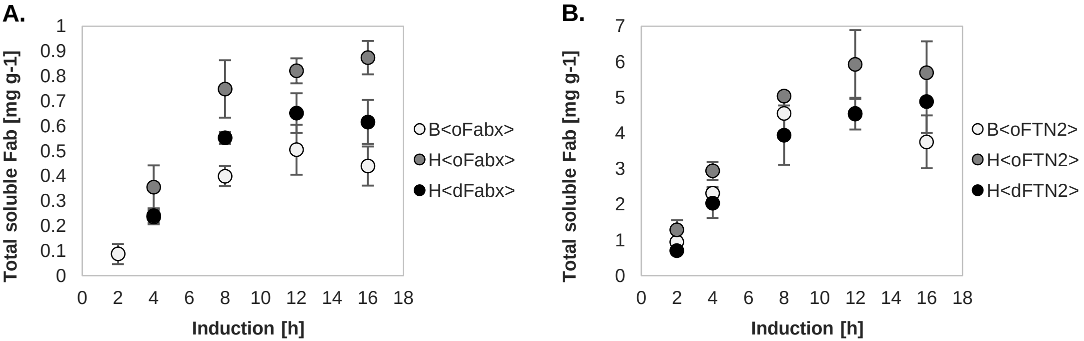

Figure S5. Simplified illustration of the experimental setup. Two Fabs (Fabx and FTN2) were produced in genome-integrated *E. coli* strains BL21(DE3) and HMS174(DE3) in C-limited fed-batch cultivations in the DASGIP parallel bioreactor system in lab scale. CDM [g], Fab expression patterns and Fab yields [mg/L] were determined from samples drawn at different timepoints during the process. Differential gene expression after 2 and 12 h of induction was analyzed relative to the non-induced samples by means of RNA-seq.

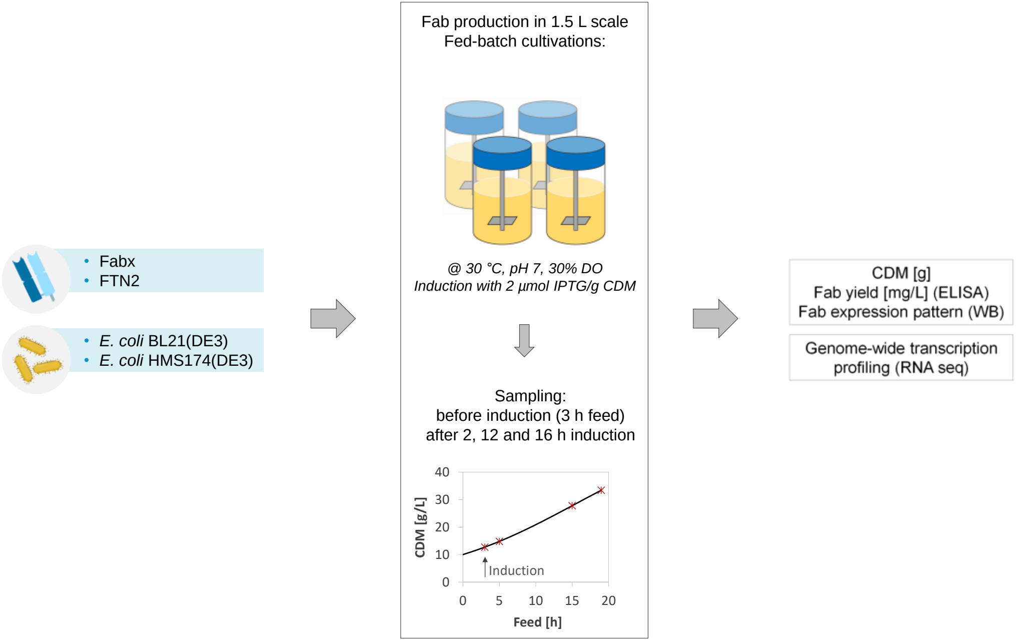

Supplement: Supplementary file 1 — Additional file 1: Figure S1. Fabx and FTN2 mRNA levels [tpm] prior to and after 2, 12 and 16 h of induction in C-limited fed-batch processes of recombinant BL21(DE3)- A and HMS174(DE3)-based expression systems B. Biological triplicates were analyzed (n=3). Figure S2. Volcano plots of differentially expressed genes in BL21(DE3)- and HMS174(DE3)-based expression systems producing Fabx A and FTN2 B in C-limited fed-batch processes after 2 and 12 h of induction relative to the respective non-induced control. DGE was determined with DESeq2. Genes also differentially expressed in the respective wild-types were excluded. Biological triplicates were analyzed (n=3). Figure S3. Cell growth A and Fab yields B of two recombinant E. coli expression systems producing ompASS-FTN2 and ompASS-FTN2(A40P) in C-limited fed-batch processes for B (n=3) and for B<oFTN2(A40P)> (n=1). Figure S4. Specific soluble Fab yields (intracellular and extracellular fractions) [mg g−1] of Fabx A and FTN2 B produced in C-limited fed-batch cultivations. Cultivations were done in triplicates Fab yields at the different time points were analysed from two to three biological replicates (n=2 or n=3). Figure S5. Simplified illustration of the experimental setup. Two Fabs (Fabx and FTN2) were produced in genome-integrated E. coli strains BL21(DE3) and HMS174(DE3) in C-limited fed-batch cultivations in the DASGIP parallel bioreactor system in lab scale. CDM [g], Fab expression patterns and Fab yields [mg L−1] were determined from samples drawn at different time points during the process. Different expression after 2 and 12 h of induction was analysed relative to the non-induced samples by means of RNA-seq. [file 12934_2023_2278_MOESM1_ESM.pdf]
