# Supplementary material for: RNA-seq reveals multifaceted gene expression response to Fab production in Escherichia coli fed-batch processes with particular focus on ribosome stalling
Source: Microb Cell Fact. 2024 Jan 5;23:14. doi: 10.1186/s12934-023-02278-w (PMC10768439; doi:10.1186/s12934-023-02278-w)
Supplement: Supplementary file 2 — Additional file 2: Table S1. Differentially expressed genes in B during fed-batch cultivation after 2 h of induction relative to the sample drawn immediately before induction of Fabx expression. Genes also differentially expressed in wild-type BL21(DE3) were excluded. Table S2. Differentially expressed genes in B during fed-batch cultivation after 2 h of induction relative to the sample drawn immediately before induction of FTN2 expression. Genes also differentially expressed in wild-type BL21(DE3) were excluded. Table S3. Differentially expressed genes in B during fed-batch cultivation after 12 h of induction relative to the sample drawn immediately before induction of Fabx expression. Genes also differentially expressed in wild-type BL21(DE3) were excluded. Table S4. Differentially expressed genes in B during fed-batch cultivation after 12 h of induction relative to the sample drawn immediately before induction of FTN2 expression. Genes also differentially expressed in wild-type BL21(DE3) were excluded. Table S5. Differentially expressed genes in H during fed-batch cultivation after 2 h of induction relative to the sample drawn immediately before induction of Fabx expression. Genes also differentially expressed in wild-type HMS174(DE3) were excluded. Table S6. Differentially expressed genes in H during fed-batch cultivation after 2 h of induction relative to the sample drawn immediately before induction of FTN2 expression. Genes also differentially expressed in wild-type HMS174(DE3) were excluded. Table S7. Differentially expressed genes in H during fed-batch cultivation after 12 h of induction relative to the sample drawn immediately before induction of Fabx expression. Genes also differentially expressed in wild-type HMS174(DE3) were excluded. Table S8. Differentially expressed genes in H during fed-batch cultivation after 12 h of induction relative to the sample drawn immediately before induction of FTN2 e [file 12934_2023_2278_MOESM2_ESM.pdf]

Additional file Tables

RNA-seq reveals multifaceted gene expression response to Fab production in *Escherichia coli* fed-batch processes with particular focus on ribosome stalling

Sophie Vazulka[1], Matteo Schiavinato[2], Christopher Tauer[1], Martin Wagenknecht[3], Monika Cserjan-Puschmann[1*], Gerald Striedner[1]

[1]Christian Doppler Laboratory for production of next-level biopharmaceuticals in E. coli, Department of Biotechnology, University of Natural Resources and Life Sciences, Muthgasse 18, A-1190 Vienna, Austria

[2]Department of Biotechnology, Institute of Computational Biology, University of Natural Resources and Life Sciences, Muthgasse 18, A-1190 Vienna, Austria

[3]Boehringer Ingelheim RCV GmbH & Co KG, Dr.-Boehringer-Gasse 5-11, A-1120 Wien

* Corresponding Author: Email: monika.cserjan@boku.ac.at

Supplementary Tables

Table S1: Differentially expressed genes in B<oFabx> during fed-batch cultivation after 2 h of induction relative to the sample drawn immediately before induction of Fabx expression. Genes also differentially expressed in wildtype BL21(DE3) were excluded.

Gene	baseMean	log2FoldChange	lfcSE	pvalue	padj
rstB	2266	3.0	0.13	2.90E-107	1.97E-104
rstA	2047	3.0	0.16	3.36E-75	1.91E-72
mgtA	3642	3.9	0.22	1.03E-70	5.00E-68
ybjX	2981	3.1	0.18	6.21E-65	2.65E-62
slyB	7068	2.2	0.15	5.17E-50	1.96E-47
phoQ	1397	1.6	0.14	4.67E-30	1.32E-27
ibpB	674	4.0	0.40	6.76E-25	1.77E-22
ycfS	456	2.0	0.19	9.36E-25	2.28E-22
yneM	1263	3.4	0.34	3.60E-24	8.17E-22
chaA	557	1.9	0.18	6.34E-24	1.35E-21
phoP	1653	1.7	0.18	2.14E-20	4.06E-18
yebO	812	1.7	0.19	9.25E-19	1.55E-16
yccA	5477	1.2	0.13	4.39E-17	6.79E-15
hemL	3452	1.2	0.14	4.37E-16	6.20E-14
tatC	1696	1.1	0.14	6.13E-14	8.35E-12
ompX	5547	1.7	0.23	7.25E-14	9.50E-12
yebE	693	1.5	0.20	8.98E-14	1.13E-11
tatD	537	1.1	0.15	2.86E-12	3.36E-10
maeA	1755	0.9	0.13	3.60E-12	4.08E-10
ompA	35032	1.6	0.23	7.05E-12	7.50E-10
clpB	3834	1.5	0.23	1.39E-11	1.39E-09
yfhB	720	1.2	0.18	8.21E-11	7.77E-09
borD	799	1.6	0.27	2.74E-10	2.49E-08
mlaF	1335	1.0	0.16	2.78E-10	2.49E-08
hslU	2110	1.4	0.23	6.46E-10	5.64E-08
speG	676	1.1	0.19	1.78E-08	1.48E-06
ynfB	582	1.1	0.20	3.97E-08	3.22E-06
tadA	436	0.9	0.18	7.30E-08	5.52E-06
bglA	1532	0.9	0.17	7.26E-08	5.52E-06
mgrB	139	1.8	0.37	1.02E-07	7.58E-06
htpX	1807	0.9	0.17	1.23E-07	8.88E-06
yceI	852	1.1	0.22	1.99E-07	1.41E-05
pspA	2244	1.3	0.27	2.43E-07	1.69E-05
ycjX	328	1.1	0.22	6.40E-07	4.27E-05
tqsA	142	1.2	0.27	9.56E-07	6.26E-05
ampH	1100	0.7	0.16	1.64E-06	1.05E-04
ybeD	467	1.0	0.22	2.58E-06	1.60E-04
mdoB	1496	0.7	0.16	2.93E-06	1.78E-04
hslV	403	1.3	0.32	4.13E-06	2.47E-04
pmrD	280	0.9	0.24	1.43E-05	8.26E-04

Supplementary Tables

mlaE	748	0.8	0.21	1.74E-05	9.87E-04
yfeY	894	-0.7	0.19	2.14E-05	1.17E-03
secA	4062	0.6	0.19	4.97E-05	2.65E-03
dacC	3190	0.8	0.26	8.20E-05	4.17E-03
yebZ	418	0.6	0.18	1.16E-04	5.70E-03
pspB	345	1.1	0.42	1.18E-04	5.73E-03
yqjA	509	0.8	0.29	1.23E-04	5.92E-03
ydeH	351	0.7	0.25	1.70E-04	7.93E-03
ybbN	1309	0.6	0.23	2.68E-04	1.22E-02
pspD	273	0.9	0.40	2.77E-04	1.24E-02
gmr	1127	0.7	0.30	2.88E-04	1.27E-02
mlaD	689	0.6	0.21	3.23E-04	1.38E-02
efeO	665	-0.7	0.28	3.83E-04	1.61E-02
stpA	202	-0.8	0.35	3.92E-04	1.63E-02
pspC	434	0.9	0.47	5.39E-04	2.18E-02
yobA	563	0.6	0.24	5.86E-04	2.35E-02
yfiD	1428	0.6	0.27	6.02E-04	2.38E-02
yoaK	127	-0.7	0.35	6.93E-04	2.68E-02
ycjF	432	0.6	0.30	7.11E-04	2.69E-02
bax	5435	-0.6	0.23	7.05E-04	2.69E-02
fxsA	217	0.7	0.33	8.03E-04	3.00E-02
mzrA	196	0.7	0.37	1.08E-03	3.97E-02
yobF	1233	-0.6	0.30	1.18E-03	4.22E-02
smpA	2774	-0.5	0.20	1.20E-03	4.25E-02
yfgD	1128	-0.5	0.20	1.21E-03	4.25E-02

Table S2: Differentially expressed genes in B<oFTN2> during fed-batch cultivation after 2 h of induction relative to the sample drawn immediately before induction of FTN2 expression. Genes also differentially expressed in wildtype BL21(DE3) were excluded.

Gene	baseMean	log2FoldChange	lfcSE	pvalue	padj
ybjX	3358	3.0	0.15	4.68E-84	3.81E-81
rstA	2131	2.6	0.17	7.20E-52	4.89E-49
mgtA	3622	3.8	0.25	5.75E-51	3.34E-48
rstB	2318	2.6	0.20	4.02E-37	1.82E-34
yneM	1165	3.0	0.28	2.28E-26	9.29E-24
phoP	1691	1.4	0.16	9.01E-19	2.62E-16
slyB	7461	1.7	0.20	1.35E-17	3.44E-15
borD	934	1.8	0.25	5.36E-15	1.21E-12
ompX	4771	1.2	0.16	2.95E-13	6.00E-11
ycfS	546	1.6	0.23	3.64E-13	7.07E-11
phoQ	1359	1.3	0.19	4.57E-12	8.46E-10
yebO	896	1.4	0.20	5.33E-12	9.43E-10
chaA	525	1.5	0.23	1.84E-11	2.99E-09

mgrB	153	1.7	0.31	2.45E-10	3.84E-08
ompA	38333	1.0	0.17	1.00E-09	1.36E-07
yfhB	877	0.9	0.14	9.80E-10	1.36E-07
tus	298	1.2	0.21	4.97E-09	6.13E-07
yccA	5764	0.9	0.16	1.47E-08	1.71E-06
mlaF	1555	0.9	0.17	1.78E-07	1.86E-05
yqjA	555	1.0	0.19	2.02E-07	2.06E-05
speG	811	0.9	0.18	5.09E-07	5.06E-05
ynfB	759	0.8	0.18	1.56E-06	1.52E-04
yfdE	73	1.7	0.50	3.15E-06	2.98E-04
tatC	1987	0.7	0.15	3.38E-06	3.06E-04
yebE	771	0.9	0.23	1.17E-05	9.74E-04
ydcW	11048	-0.9	0.24	2.63E-05	1.99E-03
mzrA	187	0.9	0.25	5.07E-05	3.63E-03
pstS	832	0.6	0.16	5.73E-05	4.03E-03
pstA	268	0.7	0.20	5.95E-05	4.11E-03
gmr	1228	0.7	0.20	6.22E-05	4.22E-03
pmrD	337	0.7	0.19	6.38E-05	4.26E-03
mdoB	1841	0.7	0.18	6.68E-05	4.39E-03
ugpC	2384	-0.6	0.16	7.88E-05	5.10E-03
yceI	939	0.8	0.24	8.24E-05	5.22E-03
astE	2299	-0.8	0.23	8.83E-05	5.45E-03
rbsA	119	1.0	0.37	9.36E-05	5.69E-03
yfdX	57	1.2	0.48	1.10E-04	6.40E-03
ugpA	1125	-0.7	0.22	1.10E-04	6.40E-03
ugpQ	1802	-0.6	0.16	1.19E-04	6.82E-03
glmS	4102	-0.5	0.15	1.45E-04	8.09E-03
ytfT	481	-0.7	0.22	1.53E-04	8.41E-03
ydeH	330	0.7	0.22	1.71E-04	9.29E-03
tatD	567	0.8	0.25	1.86E-04	9.98E-03
grxB	1553	-0.7	0.24	2.12E-04	1.12E-02
rbsC	287	0.7	0.24	2.84E-04	1.46E-02
ugpE	701	-0.6	0.19	2.93E-04	1.47E-02
matA	65	1.1	0.58	3.28E-04	1.63E-02
efeO	976	-0.5	0.16	3.54E-04	1.70E-02
yrbL	304	0.6	0.21	3.51E-04	1.70E-02
ytfL	1870	-0.5	0.14	3.87E-04	1.83E-02
hemL	4247	0.6	0.21	3.96E-04	1.85E-02
matB	55	1.1	0.66	4.04E-04	1.87E-02
alx	142	0.8	0.37	5.22E-04	2.36E-02
mtr	436	0.6	0.20	5.66E-04	2.53E-02
mlaD	1076	0.6	0.21	6.16E-04	2.73E-02
tqsA	125	0.8	0.33	6.45E-04	2.82E-02
flgB	2754	0.6	0.23	6.94E-04	3.01E-02

Supplementary Tables

yfiD	1399	0.5	0.19	7.12E-04	3.02E-02
nudK	415	-0.5	0.19	7.12E-04	3.02E-02
ydcV	1334	-0.7	0.27	7.54E-04	3.17E-02
mlaE	974	0.5	0.19	7.77E-04	3.18E-02
cfa	10805	-0.5	0.18	7.81E-04	3.18E-02
htpX	2436	0.5	0.18	7.99E-04	3.22E-02
rbsD_2	37	0.9	0.59	8.48E-04	3.32E-02
pstC	296	0.5	0.20	9.41E-04	3.62E-02
fbaB	3034	-0.5	0.17	9.58E-04	3.65E-02
dacC	3587	0.5	0.19	1.06E-03	3.95E-02
glmU	2487	-0.4	0.14	1.20E-03	4.41E-02
maeA	2244	0.5	0.22	1.24E-03	4.50E-02
gcd	2055	-0.6	0.24	1.30E-03	4.70E-02
tadA	454	0.5	0.19	1.39E-03	4.93E-02
cynX	273	0.7	0.33	1.39E-03	4.93E-02

Table S3: Differentially expressed genes in B<oFabx> during fed-batch cultivation after 12 h of induction relative to the sample drawn immediately before induction of Fabx expression. Genes also differentially expressed in wildtype BL21(DE3) were excluded.

Gene	baseMean	log2FoldChange	lfcSE	pvalue	padj
ibpB	1953	5.7	0.25	3.58E-114	1.47E-110
ssuD	924	4.4	0.23	8.26E-77	3.77E-74
pspA	12233	4.2	0.25	2.43E-61	5.26E-59
yebE	2380	3.6	0.22	1.48E-57	2.43E-55
ssuA	444	4.0	0.26	2.63E-52	3.60E-50
soxS	1397	4.1	0.27	4.98E-52	6.59E-50
pspD	1005	3.5	0.24	2.77E-45	2.75E-43
psiE	407	4.0	0.29	7.22E-43	6.44E-41
pspB	1449	3.8	0.28	3.17E-42	2.65E-40
ycfS	1026	3.4	0.27	4.26E-35	2.49E-33
cspA	50411	3.8	0.32	1.84E-32	8.58E-31
pspC	1456	3.3	0.29	1.16E-30	5.03E-29
sgrT	29944	-3.0	0.26	1.89E-30	7.91E-29
cpxP	6092	3.3	0.30	1.06E-27	3.77E-26
marA	683	3.1	0.29	2.34E-26	7.27E-25
ssuC	272	2.9	0.27	3.34E-26	1.03E-24
ydeN	1103	-3.0	0.28	6.65E-26	1.96E-24
ssuB	427	2.8	0.26	1.53E-25	4.33E-24
uspF	1402	-3.1	0.30	1.85E-25	5.08E-24
rbsB	1641	-2.3	0.22	2.19E-25	5.99E-24
ychH	1175	-2.2	0.21	5.66E-24	1.47E-22
pspG	226	2.8	0.28	8.16E-24	2.08E-22
malM	8693	-3.2	0.32	1.21E-22	2.86E-21

Supplementary Tables

ybjH	539	2.9	0.30	4.32E-21	8.82E-20
dctA	4314	-3.0	0.32	1.04E-20	2.07E-19
eutM	234	-2.4	0.25	1.13E-20	2.21E-19
yqjA	1272	2.7	0.28	1.95E-20	3.77E-19
spy	1299	2.6	0.28	3.72E-20	7.00E-19
rplQ	20750	2.1	0.23	9.62E-20	1.77E-18
cstA	5067	-2.7	0.30	1.60E-19	2.88E-18
chaA	789	2.5	0.27	2.20E-19	3.95E-18
tnaA	308	-2.2	0.24	1.13E-18	1.92E-17
marR	216	2.8	0.31	1.73E-18	2.89E-17
glpK	1647	-2.2	0.24	1.76E-18	2.93E-17
alx	441	2.3	0.25	1.88E-18	3.08E-17
yjhX	85	-3.4	0.40	3.30E-18	5.34E-17
mzrA	379	2.3	0.26	5.25E-18	8.26E-17
yfaW	272	-2.1	0.24	3.58E-17	5.31E-16
rplT	29034	2.4	0.28	3.95E-17	5.81E-16
yeiT	314	-2.2	0.26	4.84E-17	7.05E-16
marB	288	2.8	0.34	1.32E-16	1.88E-15
ptsG	7417	2.2	0.26	1.64E-16	2.32E-15
rplY	6237	2.1	0.25	2.09E-16	2.92E-15
bax	3915	-2.4	0.29	3.07E-16	4.25E-15
yjjM	220	-2.4	0.30	4.87E-16	6.64E-15
ybdD	269	-2.1	0.25	1.70E-15	2.24E-14
ompX	7642	2.3	0.29	7.83E-15	9.97E-14
priB	5289	1.9	0.24	1.21E-14	1.51E-13
ydeM	319	-2.2	0.28	2.77E-14	3.33E-13
ygdR	569	-2.0	0.25	3.04E-14	3.65E-13
pyrD	1005	1.9	0.25	3.80E-14	4.52E-13
rpsB	24473	1.8	0.23	6.08E-14	7.14E-13
yccA	8823	2.1	0.27	6.34E-14	7.42E-13
hslO	1813	1.8	0.23	8.07E-14	9.26E-13
guaB	2622	1.6	0.21	8.67E-14	9.88E-13
rpsG	14298	1.9	0.24	8.72E-14	9.91E-13
aceB	2037	-2.1	0.28	1.58E-13	1.76E-12
rpmI	17912	2.2	0.30	1.87E-13	2.08E-12
ompW	214	-2.0	0.26	4.38E-13	4.80E-12
ygiW	2568	2.2	0.29	5.43E-13	5.86E-12
rhaR	299	-1.9	0.26	5.85E-13	6.29E-12
infA	5698	2.1	0.29	1.06E-12	1.12E-11
mscS	2158	1.9	0.26	1.43E-12	1.49E-11
rpsD	30059	1.7	0.23	1.66E-12	1.72E-11
rpsL	11091	1.8	0.24	1.86E-12	1.91E-11
yihY	377	-1.6	0.22	2.97E-12	3.01E-11
fusA	49013	1.6	0.22	3.22E-12	3.25E-11

Supplementary Tables

rpoA	42758	1.6	0.23	3.63E-12	3.64E-11
glpF	545	-1.8	0.26	4.02E-12	4.02E-11
rpmF	10928	1.8	0.25	6.20E-12	6.07E-11
yceD	10294	1.8	0.26	6.34E-12	6.19E-11
rpmG	8968	2.1	0.30	7.17E-12	6.90E-11
grpE	8398	1.9	0.28	7.93E-12	7.60E-11
ybl115	874	-1.8	0.26	8.46E-12	8.08E-11
ndh	909	1.7	0.25	9.46E-12	8.99E-11
rpmA	15370	1.9	0.27	1.13E-11	1.07E-10
yeiP	1140	1.9	0.27	1.22E-11	1.14E-10
ybl162	410	-1.6	0.23	1.64E-11	1.53E-10
hslR	609	1.6	0.24	1.73E-11	1.61E-10
serA	13121	1.5	0.21	2.31E-11	2.13E-10
treC	364	-1.7	0.25	2.56E-11	2.34E-10
ucpA	1340	-1.5	0.22	2.88E-11	2.62E-10
rpsN	14696	1.5	0.22	2.99E-11	2.71E-10
yeiA	376	-1.8	0.26	3.99E-11	3.59E-10
nmpC_1	345	-2.1	0.32	4.35E-11	3.89E-10
asr	195	1.8	0.27	5.71E-11	5.05E-10
yhcN	333	4.3	0.73	5.81E-11	5.12E-10
rpsH	13267	1.6	0.24	7.09E-11	6.20E-10
rpsE	16701	1.6	0.24	7.31E-11	6.38E-10
rpsU	11793	1.9	0.28	9.13E-11	7.90E-10
ivbL	1246	-2.0	0.31	9.80E-11	8.47E-10
aceA	4406	-1.9	0.29	1.09E-10	9.38E-10
tufA	31026	1.5	0.22	1.67E-10	1.41E-09
cbrB	214	2.1	0.32	1.85E-10	1.55E-09
aphA	492	-1.5	0.22	1.93E-10	1.61E-09
aroG	7097	1.5	0.22	1.97E-10	1.64E-09
rpsA	60096	1.5	0.22	2.65E-10	2.20E-09
gpt	2151	1.7	0.26	3.20E-10	2.62E-09
cpdB	2422	-1.4	0.21	3.37E-10	2.75E-09
rplX	20857	1.6	0.24	3.98E-10	3.23E-09
yncL	86	-2.4	0.39	4.64E-10	3.75E-09
yacH	262	1.7	0.26	4.88E-10	3.94E-09
suhB	1252	1.5	0.23	5.70E-10	4.57E-09
mqo	400	1.5	0.23	6.45E-10	5.15E-09
eutQ	84	-2.1	0.34	6.70E-10	5.34E-09
rplU	7846	1.7	0.26	7.78E-10	6.14E-09
cusC	1040	-1.7	0.28	9.38E-10	7.36E-09
yaiZ	185	-1.7	0.27	9.39E-10	7.36E-09
tsf	17834	1.5	0.23	1.09E-09	8.50E-09
atoC	298	-1.5	0.24	1.18E-09	9.15E-09
ascF	300	-1.5	0.24	1.20E-09	9.31E-09

lpxP	1238	1.6	0.25	1.33E-09	1.03E-08
rplR	3016	1.4	0.22	1.56E-09	1.20E-08
gltJ	1748	-1.5	0.24	1.69E-09	1.29E-08
yncJ	71	2.2	0.37	1.77E-09	1.36E-08
nlpD	6344	-1.6	0.26	1.93E-09	1.47E-08
queD	559	1.7	0.28	2.01E-09	1.52E-08
yhjE	2545	1.3	0.21	2.37E-09	1.78E-08
rpsK	16070	1.4	0.24	3.64E-09	2.71E-08
ybl116	360	-1.8	0.30	4.20E-09	3.11E-08
rpsT	9476	1.7	0.28	4.64E-09	3.43E-08
ybl114	1413	-1.8	0.29	4.75E-09	3.50E-08
cbrA	255	1.6	0.27	4.97E-09	3.65E-08
yhbW	1319	1.3	0.22	5.43E-09	3.96E-08
atoS	204	-1.4	0.24	6.44E-09	4.66E-08
ybl139	374	1.6	0.27	7.20E-09	5.18E-08
nanE	174	-1.6	0.27	7.60E-09	5.46E-08
livJ	8705	-1.5	0.26	1.03E-08	7.28E-08
gpmM	2752	1.3	0.23	1.05E-08	7.46E-08
ompA	28819	1.3	0.22	1.07E-08	7.55E-08
yhcH	215	-1.4	0.24	1.07E-08	7.58E-08
nadA	532	-1.5	0.25	1.21E-08	8.48E-08
dsbA	3863	1.6	0.27	1.23E-08	8.58E-08
yihX	551	-1.3	0.22	1.47E-08	1.02E-07
aceK	1007	-1.5	0.26	1.52E-08	1.05E-07
ptsH	9031	1.3	0.23	1.53E-08	1.06E-07
yciH	419	1.5	0.26	1.74E-08	1.20E-07
ygaXY	72	2.0	0.35	1.99E-08	1.36E-07
nuoJ	2283	-1.7	0.29	2.46E-08	1.68E-07
pal	16137	1.3	0.24	2.54E-08	1.73E-07
yrbN	49	2.4	0.43	2.96E-08	2.00E-07
gapA	58218	1.5	0.26	3.48E-08	2.34E-07
rplO	3671	1.3	0.23	4.01E-08	2.68E-07
yobB	573	1.5	0.27	4.17E-08	2.78E-07
prfC	1826	1.3	0.23	4.18E-08	2.79E-07
rpmJ	9488	1.5	0.27	4.56E-08	3.02E-07
pnuC	586	-1.3	0.23	4.57E-08	3.02E-07
prpD	457	-1.5	0.26	4.62E-08	3.06E-07
nuoH	3163	-1.7	0.31	5.11E-08	3.36E-07
secA	5045	1.2	0.23	5.65E-08	3.70E-07
rplN	23857	1.3	0.24	5.87E-08	3.84E-07
cusB	561	-1.6	0.29	6.16E-08	4.01E-07
orf28	69	1.8	0.33	6.18E-08	4.01E-07
nuoB	4720	-1.5	0.27	6.65E-08	4.30E-07
nanK	165	-1.4	0.26	6.70E-08	4.33E-07

Supplementary Tables

secY	40468	1.5	0.27	7.04E-08	4.53E-07
ybhP	100	-1.6	0.30	7.17E-08	4.61E-07
cusF	443	-1.7	0.32	7.23E-08	4.64E-07
ybiS	1782	1.3	0.23	7.60E-08	4.86E-07
abgR	222	-1.4	0.25	8.57E-08	5.43E-07
lldP	355	-1.4	0.25	9.27E-08	5.85E-07
pyrF	1316	1.3	0.24	9.68E-08	6.10E-07
glpT	443	-1.6	0.29	9.77E-08	6.15E-07
exuT	481	-1.2	0.23	1.02E-07	6.37E-07
ydeA	261	1.3	0.24	1.04E-07	6.51E-07
sulA	807	1.3	0.24	1.06E-07	6.61E-07
aaeA	255	1.7	0.32	1.21E-07	7.52E-07
nadB	642	-1.2	0.23	1.29E-07	8.03E-07
eutT	77	-1.8	0.33	1.50E-07	9.26E-07
xdhA	310	-1.3	0.25	2.12E-07	1.30E-06
fdoG	3154	-1.1	0.21	2.40E-07	1.47E-06
pitA	3355	1.2	0.24	2.71E-07	1.65E-06
putP	2150	-1.4	0.27	2.75E-07	1.67E-06
gabD	406	-1.4	0.27	2.86E-07	1.73E-06
thiI	1618	1.2	0.24	2.88E-07	1.75E-06
mrcA	1776	1.2	0.23	2.95E-07	1.78E-06
yeeN	801	1.4	0.27	2.96E-07	1.79E-06
nuoG	9116	-1.6	0.31	3.35E-07	2.01E-06
nuoI	1683	-1.5	0.30	3.44E-07	2.06E-06
garD	369	-1.3	0.25	3.72E-07	2.22E-06
rph	1220	1.2	0.24	3.84E-07	2.29E-06
maeB	5200	-1.4	0.28	4.05E-07	2.41E-06
mdtL	245	1.4	0.29	4.41E-07	2.61E-06
yjhP	193	-1.4	0.28	4.48E-07	2.65E-06
nuoE	2163	-1.6	0.32	4.64E-07	2.74E-06
bcsZ	766	-1.1	0.23	5.01E-07	2.95E-06
nuoA	3929	-1.4	0.29	5.18E-07	3.05E-06
nuoL	5168	-1.5	0.30	5.20E-07	3.06E-06
plsX	5208	1.4	0.29	5.85E-07	3.44E-06
nuoF	5714	-1.6	0.32	6.52E-07	3.81E-06
nuoC	5230	-1.6	0.32	6.75E-07	3.92E-06
cbrC	441	1.3	0.27	7.17E-07	4.16E-06
nuoK	664	-1.5	0.30	7.46E-07	4.32E-06
yjhQ	97	-1.5	0.31	7.46E-07	4.32E-06
tisB	3626	1.3	0.26	7.76E-07	4.48E-06
rlmG	383	1.1	0.22	8.03E-07	4.63E-06
rpsM	19253	1.3	0.26	8.19E-07	4.71E-06
ydeH	470	1.5	0.30	8.60E-07	4.93E-06
yccK	132	1.5	0.31	8.68E-07	4.97E-06

yebO	677	1.4	0.28	8.77E-07	5.01E-06
gcvH	1002	1.3	0.27	9.62E-07	5.49E-06
grxA	259	1.5	0.30	9.85E-07	5.60E-06
araG	278	-1.2	0.26	1.16E-06	6.57E-06
carA	2223	1.1	0.22	1.17E-06	6.60E-06
gltK	1293	-1.2	0.25	1.17E-06	6.62E-06
azuC	31	2.0	0.43	1.20E-06	6.77E-06
eutS	26	-2.3	0.49	1.22E-06	6.87E-06
yahN	121	-1.5	0.32	1.26E-06	7.07E-06
mrdA	1018	1.2	0.26	1.34E-06	7.47E-06
gabT	594	-1.2	0.26	1.44E-06	8.05E-06
hsdM	959	1.0	0.22	1.94E-06	1.07E-05
yebK	846	-1.2	0.27	2.10E-06	1.16E-05
ybgF	5560	1.1	0.24	2.31E-06	1.27E-05
yiaJ	428	1.1	0.23	2.33E-06	1.28E-05
yidD	311	1.1	0.24	2.53E-06	1.39E-05
araH	252	-1.2	0.27	2.71E-06	1.48E-05
yneE	151	1.5	0.33	2.80E-06	1.53E-05
fabB	3454	-1.0	0.22	2.80E-06	1.53E-05
ral	424	1.3	0.29	2.81E-06	1.53E-05
uxaC	393	-1.1	0.24	2.81E-06	1.53E-05
eutL	136	-1.3	0.27	2.87E-06	1.55E-05
ppiC	597	1.3	0.28	3.15E-06	1.70E-05
setA	623	-1.1	0.23	3.22E-06	1.74E-05
yfcL	789	1.3	0.27	3.26E-06	1.75E-05
rbsK	412	-1.0	0.23	3.32E-06	1.78E-05
ychN	1517	-1.2	0.27	3.36E-06	1.80E-05
ybhC	245	1.1	0.24	3.37E-06	1.81E-05
pdhR	779	1.2	0.27	3.40E-06	1.82E-05
gltP	602	1.1	0.25	3.55E-06	1.90E-05
infC	25047	1.2	0.26	3.56E-06	1.90E-05
yifE	3787	1.3	0.28	3.56E-06	1.90E-05
argR	2161	1.3	0.29	3.65E-06	1.94E-05
sugE	232	-1.2	0.26	3.89E-06	2.06E-05
acnB	30986	-1.3	0.28	4.19E-06	2.21E-05
nanT	201	-1.1	0.25	5.15E-06	2.70E-05
xseA	820	1.1	0.24	5.50E-06	2.87E-05
queA	434	1.1	0.25	5.57E-06	2.90E-05
yieH	540	1.2	0.27	5.64E-06	2.94E-05
gam	138	1.3	0.28	5.68E-06	2.96E-05
nuoM	4140	-1.3	0.29	5.85E-06	3.04E-05
epd	2040	1.1	0.24	6.01E-06	3.12E-05
iscX	970	1.1	0.25	6.05E-06	3.13E-05
yecJ	152	1.2	0.28	6.30E-06	3.26E-05

xdhB	81	-1.5	0.33	6.34E-06	3.28E-05
hepA	877	1.0	0.22	6.94E-06	3.58E-05
glpQ	985	-1.1	0.25	7.23E-06	3.72E-05
yhaL	222	-1.2	0.27	7.27E-06	3.74E-05
glnK	78	1.4	0.32	7.67E-06	3.94E-05
hisQ	1545	-1.0	0.22	7.92E-06	4.06E-05
guaA	4656	0.9	0.22	8.30E-06	4.24E-05
tsx	1503	1.2	0.27	8.44E-06	4.31E-05
yibT	485	1.1	0.25	9.37E-06	4.77E-05
yncD	711	1.0	0.23	9.49E-06	4.82E-05
yabI	537	-1.0	0.24	9.56E-06	4.85E-05
nfsB	1523	1.0	0.24	9.90E-06	5.00E-05
yeaV	126	-1.2	0.28	1.01E-05	5.09E-05
qseB	339	1.0	0.23	1.05E-05	5.29E-05
pyrE	414	1.2	0.28	1.11E-05	5.59E-05
yjgH	124	-1.2	0.29	1.17E-05	5.87E-05
rseB	3234	-0.9	0.22	1.32E-05	6.60E-05
yibI	78	-1.3	0.32	1.37E-05	6.82E-05
ynfK	536	-1.0	0.23	1.42E-05	7.08E-05
hypD	178	-1.1	0.26	1.43E-05	7.09E-05
prpE	572	-1.1	0.27	1.43E-05	7.10E-05
dos	737	-1.0	0.23	1.54E-05	7.61E-05
pmrD	124	-1.3	0.32	1.59E-05	7.85E-05
hflC	5387	1.1	0.25	1.65E-05	8.13E-05
pheA	5484	1.0	0.23	1.79E-05	8.79E-05
bet	241	1.2	0.29	1.79E-05	8.80E-05
argS	2928	1.0	0.23	1.81E-05	8.87E-05
ea22	270	1.0	0.25	1.81E-05	8.88E-05
eutK	108	-1.2	0.28	1.89E-05	9.23E-05
tesB	884	1.0	0.25	1.98E-05	9.65E-05
yceA	1991	1.0	0.25	2.01E-05	9.78E-05
qseC	452	1.0	0.23	2.25E-05	1.09E-04
yoaD	159	-1.1	0.27	2.31E-05	1.12E-04
ttcA	935	1.1	0.26	2.93E-05	1.41E-04
pphA	97	-1.3	0.32	3.06E-05	1.47E-04
feaB	522	-0.9	0.23	3.11E-05	1.49E-04
mltF_1	394	1.1	0.28	3.14E-05	1.51E-04
lolD	733	1.0	0.25	3.58E-05	1.71E-04
lldR	78	-1.4	0.35	3.61E-05	1.72E-04
tktA	12693	0.9	0.21	4.01E-05	1.90E-04
rdgB	537	0.9	0.22	4.13E-05	1.95E-04
hisM	650	-1.0	0.24	4.49E-05	2.11E-04
lnt	879	0.9	0.23	4.68E-05	2.19E-04
yhjQ	379	-0.9	0.23	4.74E-05	2.22E-04

Supplementary Tables

dinI	450	1.0	0.26	4.83E-05	2.26E-04
ybl100	57	-1.3	0.35	4.84E-05	2.26E-04
tufB	21545	0.9	0.24	4.90E-05	2.28E-04
ybaV	160	-1.1	0.29	5.41E-05	2.51E-04
prpB	440	-1.1	0.28	5.46E-05	2.53E-04
panD	1753	0.9	0.23	5.79E-05	2.68E-04
sgcQ	247	-1.0	0.26	5.93E-05	2.74E-04
cpxA	2347	0.9	0.24	6.10E-05	2.81E-04
rlmH	652	1.0	0.26	6.10E-05	2.81E-04
argE	1516	0.8	0.21	6.80E-05	3.13E-04
yeeA	330	-0.9	0.24	7.13E-05	3.28E-04
ybiV	483	1.0	0.28	7.38E-05	3.38E-04
ydcP	957	0.9	0.25	7.42E-05	3.40E-04
nfuA	5102	0.9	0.24	7.44E-05	3.40E-04
nfsA	882	0.9	0.24	7.54E-05	3.44E-04
serC	18023	0.9	0.24	7.54E-05	3.44E-04
yfdC	125	-1.1	0.31	7.67E-05	3.49E-04
yajQ	2630	1.1	0.29	7.67E-05	3.49E-04
aroA	3304	0.8	0.21	9.14E-05	4.14E-04
eno	44848	1.0	0.28	9.32E-05	4.21E-04
agaF	76	-1.1	0.32	9.56E-05	4.31E-04
carB	5074	0.9	0.23	9.62E-05	4.34E-04
putA	1245	-1.0	0.27	9.72E-05	4.37E-04
glyA	32117	0.9	0.23	1.00E-04	4.51E-04
yqaE	162	1.2	0.33	1.01E-04	4.52E-04
yciA	454	1.1	0.30	1.02E-04	4.56E-04
murA	3054	0.8	0.21	1.07E-04	4.78E-04
ybjD	721	-0.9	0.23	1.08E-04	4.81E-04
gatR_1	33	-1.6	0.45	1.09E-04	4.84E-04
rutA	71	1.2	0.34	1.10E-04	4.88E-04
mppA	695	-0.9	0.23	1.11E-04	4.90E-04
yccX	140	-1.1	0.30	1.12E-04	4.95E-04
sgcR	197	-0.9	0.25	1.15E-04	5.08E-04
pcnB	2062	0.8	0.21	1.20E-04	5.28E-04
eutR	163	-1.1	0.31	1.23E-04	5.39E-04
holD	180	1.0	0.27	1.24E-04	5.44E-04
yibL	888	1.0	0.29	1.27E-04	5.59E-04
sdaA	2351	1.0	0.26	1.30E-04	5.68E-04
yhbS	1113	-0.8	0.22	1.31E-04	5.74E-04
nudG	196	1.0	0.28	1.32E-04	5.77E-04
tolQ	1740	0.9	0.26	1.36E-04	5.94E-04
yaiY	46	-1.4	0.39	1.37E-04	5.96E-04
mnmA	2004	0.8	0.23	1.54E-04	6.70E-04
cusA	778	-1.0	0.27	1.54E-04	6.71E-04

Supplementary Tables

gsk	794	0.8	0.21	1.56E-04	6.77E-04
tsgA	229	1.1	0.30	1.57E-04	6.81E-04
ygaW	67	1.4	0.39	1.59E-04	6.88E-04
ygiS	891	-0.9	0.24	1.67E-04	7.22E-04
ybaY	378	1.0	0.28	1.71E-04	7.36E-04
eutP	26	-1.7	0.50	1.77E-04	7.61E-04
amtB	362	1.0	0.27	1.80E-04	7.76E-04
yeiR	336	0.9	0.25	1.82E-04	7.81E-04
ybjC	164	1.0	0.30	1.85E-04	7.94E-04
cbpM	102	-1.1	0.33	1.85E-04	7.94E-04
yggT	766	0.9	0.26	1.87E-04	8.02E-04
gabP	156	-1.1	0.32	1.92E-04	8.20E-04
eutC	139	-1.0	0.29	2.03E-04	8.66E-04
ydiL	31	-1.6	0.47	2.07E-04	8.81E-04
gloA	1096	0.9	0.25	2.09E-04	8.89E-04
aaeB	355	0.9	0.26	2.11E-04	8.96E-04
ruvC	1230	0.8	0.22	2.21E-04	9.33E-04
iraP	346	-1.0	0.30	2.29E-04	9.69E-04
ygaH	142	1.0	0.29	2.32E-04	9.80E-04
spoT	3312	0.8	0.23	2.37E-04	9.99E-04
yebG	630	0.9	0.27	2.45E-04	1.03E-03
ypdA	346	0.9	0.25	2.47E-04	1.04E-03
ybiI	212	-0.9	0.25	2.51E-04	1.05E-03
ilvN	1579	-1.0	0.30	2.52E-04	1.06E-03
secF	3209	0.9	0.25	2.61E-04	1.09E-03
erfK	467	-0.9	0.25	2.64E-04	1.11E-03
dacA	3460	0.9	0.25	2.71E-04	1.13E-03
ybl192	129	-1.0	0.29	2.76E-04	1.15E-03
yfiM	96	-1.0	0.31	2.79E-04	1.17E-03
proQ	4912	0.8	0.24	2.81E-04	1.17E-03
yqaB	568	0.8	0.25	2.84E-04	1.18E-03
ygiB	2070	0.8	0.22	2.91E-04	1.21E-03
pflD	242	-0.9	0.26	2.91E-04	1.21E-03
gntP	235	-1.0	0.29	2.98E-04	1.24E-03
gltL_1	2493	-0.9	0.25	3.03E-04	1.26E-03
tabA	179	0.9	0.28	3.15E-04	1.30E-03
eamA	535	-0.8	0.22	3.18E-04	1.31E-03
yqeC	185	-0.9	0.27	3.29E-04	1.36E-03
endA	74	1.1	0.33	3.32E-04	1.37E-03
glpG	644	0.8	0.21	3.45E-04	1.41E-03
sgcE	106	-1.0	0.31	3.45E-04	1.41E-03
hisP	1660	-0.9	0.25	3.46E-04	1.42E-03
idnK	107	-1.0	0.30	3.57E-04	1.46E-03
grxD	2865	0.9	0.26	3.68E-04	1.50E-03

Supplementary Tables

O	352	0.9	0.25	3.80E-04	1.55E-03
tolR	1038	0.8	0.25	3.83E-04	1.56E-03
cpxR	1231	0.9	0.25	3.89E-04	1.58E-03
mrdB	613	0.8	0.23	3.92E-04	1.59E-03
bhsA	57	1.3	0.40	3.97E-04	1.61E-03
ybl67	1045	0.9	0.29	4.00E-04	1.62E-03
recN	732	0.9	0.26	4.24E-04	1.71E-03
ppa	12101	0.9	0.27	4.31E-04	1.73E-03
nusB	1936	0.8	0.22	4.45E-04	1.78E-03
rimM	9597	0.9	0.29	4.49E-04	1.80E-03
rimK	717	0.8	0.25	4.64E-04	1.86E-03
rlmE	3115	0.9	0.27	4.73E-04	1.89E-03
truB	1118	0.8	0.24	4.75E-04	1.90E-03
miaB	2116	0.8	0.23	4.79E-04	1.91E-03
trmA	572	0.9	0.27	4.80E-04	1.91E-03
rnpA	298	0.8	0.23	4.92E-04	1.95E-03
emtA	287	0.8	0.23	4.94E-04	1.96E-03
yggU	111	0.9	0.29	4.97E-04	1.97E-03
ygbI	401	-0.8	0.22	5.02E-04	1.99E-03
ybl27	328	-0.9	0.28	5.06E-04	2.00E-03
hypC	25	-1.5	0.50	5.42E-04	2.14E-03
fryB	33	-1.4	0.46	5.53E-04	2.18E-03
map	2099	0.9	0.29	5.55E-04	2.19E-03
mipA	1894	0.8	0.23	5.89E-04	2.32E-03
phoB	429	0.8	0.25	5.95E-04	2.34E-03
proS	5356	0.7	0.21	5.97E-04	2.35E-03
nuoN	3668	-0.8	0.26	6.19E-04	2.43E-03
xdhC	176	-0.8	0.27	6.54E-04	2.56E-03
srlB	32	-1.3	0.45	6.66E-04	2.61E-03
folD	1342	0.7	0.21	6.96E-04	2.72E-03
mdoC	150	1.1	0.37	7.06E-04	2.76E-03
ydcZ	362	0.8	0.23	7.24E-04	2.82E-03
yiaW	16	-1.8	0.65	7.26E-04	2.83E-03
aes	232	-0.8	0.24	7.39E-04	2.87E-03
lysS	6466	0.7	0.20	7.60E-04	2.95E-03
gloB	693	-0.8	0.24	7.76E-04	3.00E-03
yciU	1361	0.9	0.30	7.77E-04	3.00E-03
recO	405	0.8	0.24	7.79E-04	3.01E-03
sthA	3709	-0.8	0.25	8.02E-04	3.09E-03
trxB	4751	0.8	0.24	8.30E-04	3.20E-03
cpdA	2094	-0.7	0.22	8.32E-04	3.20E-03
rhaM	86	-1.0	0.35	8.42E-04	3.24E-03
pgi	4781	0.7	0.22	8.45E-04	3.24E-03
tyrS	4834	0.7	0.23	8.71E-04	3.34E-03

ysaB	38	-1.2	0.41	8.87E-04	3.39E-03
amiA	619	0.8	0.25	9.31E-04	3.56E-03
bcsG	845	-0.7	0.22	9.38E-04	3.58E-03
yaaH	399	0.9	0.29	9.44E-04	3.60E-03
dgsA	937	-0.7	0.23	9.46E-04	3.61E-03
purB	2661	0.7	0.21	9.57E-04	3.64E-03
yhfZ	202	-0.8	0.28	9.69E-04	3.68E-03
bcsA	1421	-0.7	0.22	9.78E-04	3.71E-03
aroK	3719	0.8	0.27	9.96E-04	3.77E-03
yhbY	1995	0.9	0.29	9.96E-04	3.77E-03
cmk	1320	0.8	0.24	1.01E-03	3.81E-03
dcuS	470	-0.7	0.24	1.01E-03	3.84E-03
lhgO	192	-0.8	0.27	1.02E-03	3.85E-03
hpt	2096	0.8	0.28	1.04E-03	3.92E-03
tdcA	171	-0.9	0.32	1.09E-03	4.09E-03
secD	6932	0.8	0.24	1.09E-03	4.11E-03
pheT	5909	0.7	0.23	1.09E-03	4.11E-03
cybB	1520	0.8	0.26	1.12E-03	4.21E-03
nupG	538	-0.7	0.21	1.12E-03	4.21E-03
ygaZ	721	0.8	0.26	1.13E-03	4.22E-03
fdx	1097	0.7	0.23	1.13E-03	4.23E-03
envC	732	0.7	0.24	1.17E-03	4.39E-03
msbA	2130	0.8	0.24	1.17E-03	4.39E-03
ycdZ	358	0.7	0.22	1.17E-03	4.39E-03
ptsI	30262	0.7	0.23	1.24E-03	4.64E-03
yiaY	97	-0.9	0.31	1.28E-03	4.77E-03
ibsB2	15	-1.9	0.75	1.30E-03	4.83E-03
mreB	4243	0.7	0.21	1.31E-03	4.86E-03
rbsR	259	-0.8	0.25	1.33E-03	4.93E-03
hisJ	5503	-0.6	0.19	1.34E-03	4.97E-03
dgoD	173	-0.8	0.29	1.35E-03	5.01E-03
mnmG	2611	0.7	0.21	1.37E-03	5.07E-03
yidC	5485	0.7	0.22	1.43E-03	5.26E-03
ea59_1	807	-0.9	0.30	1.43E-03	5.26E-03
yiiF	107	-0.9	0.32	1.43E-03	5.26E-03
rluC	824	0.8	0.25	1.44E-03	5.27E-03
uhpB	208	0.8	0.25	1.44E-03	5.27E-03
rsmC	814	0.7	0.23	1.44E-03	5.27E-03
dsdC	219	-0.7	0.25	1.45E-03	5.33E-03
dsbB	588	0.8	0.27	1.48E-03	5.41E-03
yqfA	561	-0.8	0.26	1.49E-03	5.44E-03
eutB	214	-0.8	0.28	1.51E-03	5.51E-03
plsC	645	0.7	0.25	1.53E-03	5.56E-03
dapD	4260	0.7	0.25	1.57E-03	5.70E-03

tdcC	182	-0.8	0.26	1.59E-03	5.76E-03
kpsS	103	-1.0	0.35	1.61E-03	5.83E-03
ybeB	686	0.8	0.25	1.63E-03	5.92E-03
srmB	704	0.7	0.21	1.64E-03	5.94E-03
kefA	1882	0.7	0.23	1.66E-03	6.02E-03
efp	5652	0.8	0.30	1.68E-03	6.08E-03
uxaB	220	-0.7	0.24	1.70E-03	6.13E-03
topA	6419	0.7	0.22	1.70E-03	6.14E-03
recA	5170	0.7	0.21	1.72E-03	6.19E-03
ybeZ	1463	0.7	0.23	1.77E-03	6.35E-03
xylH	147	-0.8	0.29	1.80E-03	6.47E-03
yigM	482	0.7	0.24	1.81E-03	6.51E-03
accD	8870	0.7	0.22	1.83E-03	6.57E-03
trxA	11093	0.8	0.27	1.84E-03	6.59E-03
rlmB	1159	0.7	0.24	1.85E-03	6.60E-03
srlR	295	-0.7	0.25	1.85E-03	6.61E-03
yciX_1	100	-0.9	0.32	1.85E-03	6.62E-03
tolB	8263	0.7	0.23	1.87E-03	6.69E-03
melA	178	-0.7	0.25	1.94E-03	6.89E-03
ldhA	661	0.7	0.24	1.94E-03	6.89E-03
yceJ	54	1.0	0.37	1.99E-03	7.08E-03
ebgC	31	-1.2	0.46	2.05E-03	7.26E-03
yeiQ	470	-0.7	0.25	2.05E-03	7.28E-03
sdaB	209	0.7	0.24	2.07E-03	7.32E-03
ade	255	-0.7	0.24	2.08E-03	7.36E-03
exo	161	0.8	0.30	2.09E-03	7.37E-03
rhaA	258	-0.8	0.28	2.09E-03	7.37E-03
yegQ	660	0.7	0.21	2.10E-03	7.40E-03
srlE	120	-0.9	0.32	2.18E-03	7.68E-03
ydjF	299	-0.7	0.24	2.19E-03	7.69E-03
orf60a	24	1.3	0.52	2.20E-03	7.75E-03
ybl32	109	0.8	0.30	2.21E-03	7.77E-03
rluA	309	0.7	0.25	2.39E-03	8.36E-03
ybl222	155	-0.8	0.30	2.40E-03	8.38E-03
yhfY	137	-0.8	0.27	2.45E-03	8.55E-03
trmH	399	0.7	0.25	2.47E-03	8.60E-03
minD	3435	-0.6	0.21	2.47E-03	8.62E-03
ahpF	1913	0.7	0.22	2.50E-03	8.71E-03
phoU	1133	0.7	0.26	2.53E-03	8.80E-03
yijD	1913	-0.7	0.26	2.59E-03	9.01E-03
yccF	209	0.8	0.28	2.62E-03	9.08E-03
E	696	0.7	0.22	2.64E-03	9.13E-03
inaA	586	0.7	0.24	2.66E-03	9.21E-03
fucA_1	55	-1.0	0.38	2.68E-03	9.28E-03

Supplementary Tables

infB	10387	0.7	0.24	2.73E-03	9.42E-03
ea10	293	0.8	0.31	2.73E-03	9.42E-03
talB	7932	0.6	0.21	2.77E-03	9.58E-03
yoaC	276	-0.8	0.29	2.81E-03	9.70E-03
vacJ	1364	0.7	0.25	2.83E-03	9.75E-03
yfeH	464	0.7	0.25	2.84E-03	9.77E-03
uxaA	397	-0.7	0.23	2.86E-03	9.84E-03
fryC	85	-0.9	0.33	2.91E-03	9.98E-03
yjiX	44	1.1	0.44	2.95E-03	1.01E-02
sgcC	243	-0.7	0.25	3.03E-03	1.04E-02
yieG	2861	0.7	0.23	3.06E-03	1.04E-02
rhaD	101	-0.8	0.31	3.07E-03	1.05E-02
ygfI	108	-0.8	0.31	3.12E-03	1.06E-02
yciB	404	0.7	0.26	3.15E-03	1.07E-02
ybiO	322	-0.7	0.23	3.20E-03	1.09E-02
phoR	414	0.7	0.23	3.21E-03	1.09E-02
srlD	215	-0.7	0.27	3.39E-03	1.15E-02
acpP	37197	0.7	0.27	3.49E-03	1.18E-02
uup	1551	0.6	0.22	3.51E-03	1.19E-02
yeeX	5113	0.7	0.28	3.57E-03	1.21E-02
yihA	1207	0.7	0.25	3.62E-03	1.22E-02
yeaH	130	-0.9	0.35	3.63E-03	1.23E-02
mdoD	1572	0.7	0.25	3.64E-03	1.23E-02
proP	707	0.7	0.24	3.66E-03	1.23E-02
sohA	543	-0.8	0.31	3.71E-03	1.25E-02
puuP	760	-0.7	0.25	3.78E-03	1.27E-02
uspA	4883	-0.6	0.21	3.82E-03	1.28E-02
yhdN	262	0.7	0.27	3.92E-03	1.32E-02
sirA	111	0.8	0.30	3.97E-03	1.33E-02
rep	694	0.6	0.20	3.99E-03	1.34E-02
ulaD	42	-1.0	0.41	4.02E-03	1.34E-02
pntA	5090	0.6	0.22	4.03E-03	1.35E-02
yohK	204	0.7	0.26	4.05E-03	1.35E-02
ilvB	5116	-0.7	0.28	4.19E-03	1.40E-02
serB	788	0.6	0.21	4.27E-03	1.42E-02
rdoA	2739	0.8	0.31	4.31E-03	1.44E-02
yedZ	81	-0.8	0.32	4.36E-03	1.45E-02
uspE	4756	-0.6	0.24	4.37E-03	1.45E-02
topB	821	-0.6	0.20	4.43E-03	1.47E-02
dnaB	1340	0.6	0.21	4.44E-03	1.47E-02
yqjH	114	0.8	0.30	4.47E-03	1.48E-02
purR	2554	0.6	0.22	4.54E-03	1.50E-02
ftnA	5205	0.7	0.25	4.55E-03	1.50E-02
glmU	2811	0.6	0.22	4.59E-03	1.52E-02

Supplementary Tables

dgoR	255	-0.6	0.24	4.61E-03	1.52E-02
rlmN	2436	0.6	0.22	4.68E-03	1.54E-02
rihA	379	-0.7	0.28	4.69E-03	1.54E-02
fdoH	242	-0.6	0.24	4.73E-03	1.56E-02
ydgI	195	0.7	0.28	4.84E-03	1.59E-02
torY	66	-0.8	0.34	4.85E-03	1.59E-02
yjgM	526	0.7	0.26	4.87E-03	1.60E-02
gdhA	4604	0.6	0.23	4.89E-03	1.60E-02
yggW	503	0.6	0.21	4.96E-03	1.62E-02
era	1437	0.6	0.22	5.03E-03	1.64E-02
yjiN	247	-0.7	0.26	5.06E-03	1.65E-02
cII_2	25	1.1	0.49	5.10E-03	1.66E-02
mreC	1203	0.6	0.21	5.11E-03	1.66E-02
ygjI	69	-0.9	0.41	5.16E-03	1.68E-02
tolA	2452	0.6	0.23	5.18E-03	1.69E-02
yeaZ	586	0.6	0.22	5.20E-03	1.69E-02
tatC	1331	0.6	0.22	5.33E-03	1.73E-02
rraB	2517	0.7	0.25	5.53E-03	1.79E-02
potF	2025	0.6	0.21	5.54E-03	1.79E-02
tnaC	21	-1.3	0.63	5.55E-03	1.80E-02
hflK	5166	0.6	0.22	5.63E-03	1.82E-02
metG	4545	0.6	0.20	5.70E-03	1.84E-02
lhr	961	-0.6	0.20	5.72E-03	1.85E-02
yecN	643	-0.7	0.27	5.82E-03	1.88E-02
yhaV	861	-0.7	0.28	5.94E-03	1.91E-02
gutQ	356	-0.6	0.22	5.99E-03	1.93E-02
yfgG	267	0.8	0.33	6.03E-03	1.94E-02
mscL	1037	-0.7	0.27	6.06E-03	1.95E-02
rnk	1105	-0.7	0.28	6.09E-03	1.95E-02
phr	338	-0.6	0.23	6.22E-03	1.99E-02
yjbQ	194	-0.7	0.26	6.22E-03	1.99E-02
panF	352	0.6	0.23	6.30E-03	2.01E-02
yihM	218	-0.7	0.27	6.35E-03	2.03E-02
yeeY	728	-0.6	0.20	6.38E-03	2.03E-02
yfaU	168	-0.6	0.25	6.48E-03	2.06E-02
ygjD	771	0.6	0.22	6.55E-03	2.08E-02
mfd	2044	0.6	0.21	6.63E-03	2.11E-02
rlmI	1117	0.6	0.23	6.70E-03	2.13E-02
minE	1727	-0.6	0.24	6.74E-03	2.14E-02
eutN	36	-1.0	0.45	6.79E-03	2.15E-02
hsdS_2	97	0.7	0.32	6.84E-03	2.17E-02
ybeY	731	0.6	0.22	6.91E-03	2.19E-02
rsmD	334	0.6	0.24	6.92E-03	2.19E-02
kdpA	155	0.7	0.31	6.99E-03	2.21E-02

Supplementary Tables

yehY	223	-0.7	0.31	7.00E-03	2.21E-02
ydhO	457	-0.6	0.24	7.05E-03	2.23E-02
csrD	617	-0.6	0.21	7.08E-03	2.23E-02
glpE	499	0.6	0.25	7.20E-03	2.27E-02
recF	520	0.6	0.23	7.26E-03	2.29E-02
rbn	350	0.6	0.24	7.35E-03	2.31E-02
glnB	1817	0.7	0.29	7.37E-03	2.32E-02
sodA	6213	0.6	0.22	7.51E-03	2.36E-02
bcp	4214	0.6	0.26	7.61E-03	2.38E-02
ydcA	253	-0.6	0.25	7.65E-03	2.40E-02
ydcY	515	0.7	0.31	7.80E-03	2.44E-02
yohJ	111	0.7	0.28	7.86E-03	2.46E-02
rsmF	482	0.6	0.21	7.87E-03	2.46E-02
smpB	822	0.6	0.22	7.91E-03	2.47E-02
nusG	5735	0.6	0.23	7.97E-03	2.49E-02
abgB	136	-0.7	0.29	8.03E-03	2.50E-02
rnc	971	0.6	0.25	8.07E-03	2.51E-02
yacL	385	-0.6	0.25	8.10E-03	2.52E-02
ygjH	67	-0.7	0.33	8.10E-03	2.52E-02
sodB	3503	0.6	0.24	8.14E-03	2.53E-02
cspE	745	0.8	0.34	8.23E-03	2.55E-02
ilvC	29835	-0.6	0.22	8.23E-03	2.55E-02
alaS	13649	0.6	0.22	8.24E-03	2.55E-02
yeaW	71	-0.8	0.36	8.28E-03	2.56E-02
csiR	201	-0.6	0.24	8.32E-03	2.57E-02
yneH	495	0.6	0.25	8.51E-03	2.63E-02
yaaY	150	-1.6	0.95	8.54E-03	2.64E-02
gnd	9298	0.6	0.21	8.67E-03	2.67E-02
kup	709	-0.5	0.20	8.73E-03	2.69E-02
yiiD	1015	-0.6	0.21	8.75E-03	2.70E-02
uspC	442	-0.6	0.26	8.81E-03	2.71E-02
yhfK	932	-0.5	0.21	8.85E-03	2.72E-02
yehX	160	-0.7	0.30	8.88E-03	2.73E-02
ygcN	313	0.6	0.24	8.98E-03	2.76E-02
cutC	1148	-0.6	0.27	9.03E-03	2.77E-02
yeiG	665	-0.6	0.24	9.07E-03	2.78E-02
uidR	302	-0.6	0.26	9.11E-03	2.79E-02
ybl218	677	-0.6	0.22	9.15E-03	2.80E-02
miaA	6353	0.6	0.25	9.26E-03	2.83E-02
citC	47	-1.1	0.55	9.27E-03	2.83E-02
ascB	284	-0.6	0.25	9.32E-03	2.85E-02
ycbJ	185	-0.6	0.26	9.33E-03	2.85E-02
yecH	96	-0.7	0.30	9.37E-03	2.86E-02
ygcP	80	-0.7	0.31	9.39E-03	2.86E-02

Supplementary Tables

xylB	305	-0.7	0.28	9.55E-03	2.91E-02
dacB	496	0.5	0.21	9.61E-03	2.92E-02
ispB	2445	0.5	0.21	9.61E-03	2.92E-02
bcsF	78	-0.7	0.34	9.61E-03	2.92E-02
ybl123	278	-0.6	0.22	9.84E-03	2.99E-02
hycI	221	-0.6	0.25	1.01E-02	3.06E-02
rfe	1046	0.6	0.25	1.02E-02	3.09E-02
ydfH	704	0.6	0.26	1.03E-02	3.12E-02
yfiF	2446	0.6	0.22	1.03E-02	3.12E-02
yagP	80	-0.7	0.30	1.03E-02	3.12E-02
trmJ	1162	0.6	0.22	1.04E-02	3.13E-02
yfeW_1	101	-0.7	0.29	1.04E-02	3.14E-02
ygbJ	110	-0.7	0.33	1.04E-02	3.14E-02
hcaF	35	-0.9	0.43	1.05E-02	3.15E-02
chpR	159	-0.6	0.27	1.05E-02	3.16E-02
ycjD	67	-0.7	0.33	1.06E-02	3.18E-02
yafK	1485	0.6	0.22	1.06E-02	3.20E-02
crcB	392	0.7	0.29	1.07E-02	3.23E-02
mdaB	307	0.6	0.28	1.08E-02	3.24E-02
mazG	839	-0.5	0.21	1.09E-02	3.26E-02
can	5980	0.6	0.24	1.10E-02	3.30E-02
yneG	86	0.7	0.29	1.13E-02	3.38E-02
rimP	1351	0.6	0.25	1.15E-02	3.42E-02
ydhL	119	-0.7	0.30	1.15E-02	3.42E-02
elbB	834	-0.6	0.23	1.15E-02	3.44E-02
sxy	262	-0.6	0.23	1.16E-02	3.47E-02
yfeC	236	-0.7	0.33	1.17E-02	3.48E-02
ptsN	1017	-0.5	0.21	1.18E-02	3.50E-02
acrA	4943	0.6	0.25	1.18E-02	3.50E-02
pth	614	0.6	0.25	1.18E-02	3.51E-02
mgrB	39	-0.9	0.50	1.19E-02	3.52E-02
ybgC	1057	0.6	0.27	1.20E-02	3.55E-02
tomB	676	0.6	0.25	1.20E-02	3.55E-02
dcrB	3491	0.6	0.24	1.21E-02	3.60E-02
yrfG	616	0.5	0.22	1.23E-02	3.64E-02
mdtM	164	-0.6	0.26	1.25E-02	3.69E-02
ykgR	12	-1.2	0.69	1.29E-02	3.79E-02
yfjF	381	0.6	0.28	1.36E-02	3.98E-02
yejM	1541	0.6	0.23	1.37E-02	4.01E-02
cyaA	4841	0.5	0.22	1.37E-02	4.03E-02
fklB	1971	0.5	0.20	1.38E-02	4.05E-02
aaeX	52	0.9	0.46	1.40E-02	4.10E-02
acrB	10176	0.6	0.24	1.40E-02	4.10E-02
yibK	152	0.7	0.31	1.41E-02	4.13E-02

rluE	100	0.7	0.31	1.42E-02	4.14E-02
yidZ	268	0.6	0.25	1.42E-02	4.16E-02
yidL	156	-0.6	0.26	1.43E-02	4.17E-02
bioA	491	0.5	0.23	1.45E-02	4.22E-02
frmB	193	-0.6	0.27	1.45E-02	4.22E-02
yjdM	184	-0.6	0.26	1.46E-02	4.25E-02
murJ	608	0.5	0.21	1.48E-02	4.31E-02
cyaY	969	0.6	0.29	1.50E-02	4.36E-02
ybbA	401	0.5	0.23	1.50E-02	4.36E-02
gyrA	7580	0.5	0.21	1.50E-02	4.36E-02
aroF	6017	-0.6	0.25	1.50E-02	4.36E-02
nanM	177	-0.6	0.29	1.52E-02	4.41E-02
yhgF	1574	0.5	0.21	1.53E-02	4.42E-02
prlC	2571	0.5	0.22	1.53E-02	4.43E-02
srlA	57	-0.8	0.41	1.55E-02	4.49E-02
araA	159	-0.6	0.25	1.56E-02	4.49E-02
tdk	251	0.7	0.32	1.56E-02	4.50E-02
ybl103	201	0.6	0.30	1.57E-02	4.52E-02
fimE	249	-0.6	0.31	1.58E-02	4.54E-02
phnP	106	-0.6	0.27	1.58E-02	4.54E-02
ybl117	268	-0.5	0.24	1.60E-02	4.60E-02
kefC	486	-0.5	0.23	1.60E-02	4.60E-02
potI	415	0.5	0.22	1.61E-02	4.63E-02
pagP	175	0.6	0.26	1.62E-02	4.63E-02
ysaA	198	0.6	0.25	1.62E-02	4.63E-02
kdsB	1921	0.5	0.21	1.64E-02	4.70E-02
prfB	2968	0.5	0.22	1.65E-02	4.72E-02
ung	509	0.5	0.24	1.65E-02	4.72E-02
hflD	604	0.5	0.20	1.68E-02	4.79E-02
rhaS	207	-0.6	0.25	1.68E-02	4.80E-02
yciF	35	-0.8	0.44	1.69E-02	4.80E-02
udk	746	0.5	0.23	1.69E-02	4.81E-02
rimI	208	0.6	0.25	1.70E-02	4.85E-02
ybgT	818	0.6	0.32	1.71E-02	4.87E-02
yajD	728	0.6	0.25	1.73E-02	4.90E-02
yqhD	560	0.5	0.22	1.74E-02	4.92E-02
yhbE	479	0.6	0.25	1.74E-02	4.94E-02

Table S4: Differentially expressed genes in B<oFTN2> during fed-batch cultivation after 12 h of induction relative to the sample drawn immediately before induction of FTN2 expression. Genes also differentially expressed in wildtype BL21(DE3) were excluded.

Gene	baseMean	log2FoldChange	lfcSE	pvalue	padj
cpxP	4661	3.0	0.22	8.93E-42	6.13E-40

ssuA	203	4.5	0.34	5.36E-40	3.39E-38
ycfS	735	2.2	0.17	1.09E-37	6.08E-36
yebE	2050	2.8	0.22	1.84E-36	9.87E-35
ssuD	439	3.7	0.31	4.03E-33	1.86E-31
puuE	1344	1.6	0.14	6.97E-30	2.82E-28
soxS	1083	3.2	0.30	1.50E-26	4.76E-25
puuC	1471	1.8	0.17	1.99E-26	6.23E-25
yqjA	1031	2.1	0.21	7.92E-24	2.17E-22
puuB	1104	1.7	0.16	1.70E-23	4.59E-22
bax	5470	-1.4	0.13	8.53E-23	2.24E-21
mzrA	318	2.0	0.22	4.63E-19	9.39E-18
uspF	2331	-1.4	0.15	1.06E-18	2.07E-17
fadE	10015	1.1	0.11	1.10E-18	2.15E-17
spy	1345	1.9	0.22	4.32E-18	8.13E-17
cstA	10118	-0.9	0.10	4.61E-17	8.16E-16
asr	445	2.5	0.30	6.07E-17	1.06E-15
psiE	175	3.0	0.37	1.67E-16	2.75E-15
ssuC	165	2.4	0.31	3.50E-16	5.67E-15
ibpB	292	2.3	0.30	8.05E-15	1.19E-13
ssuB	242	2.0	0.26	9.76E-15	1.43E-13
rplR	8042	1.1	0.13	3.31E-14	4.51E-13
pspB	644	2.2	0.31	1.10E-13	1.43E-12
ybdD	489	-1.2	0.15	1.17E-13	1.51E-12
rbsB	2877	-1.0	0.13	2.09E-13	2.60E-12
alx	208	1.8	0.25	4.19E-13	5.08E-12
chaA	527	1.5	0.21	5.04E-13	6.07E-12
pspA	5090	2.4	0.36	7.83E-13	9.29E-12
rpsG	11880	1.1	0.14	1.14E-12	1.34E-11
puuA	3649	1.2	0.16	4.08E-12	4.61E-11
puuD	2186	1.3	0.18	5.45E-12	6.11E-11
fabB	4569	-1.2	0.16	7.77E-12	8.50E-11
yjhX	113	-1.9	0.29	7.86E-12	8.58E-11
pspC	737	1.9	0.29	1.43E-11	1.54E-10
rplT	19422	1.4	0.21	1.84E-11	1.95E-10
rpsH	12066	1.0	0.14	3.25E-11	3.36E-10
rpsN	15161	1.0	0.14	9.44E-11	9.41E-10
dos	1045	-0.9	0.12	1.12E-10	1.11E-09
rpsL	10131	1.0	0.15	2.09E-10	2.02E-09
marR	122	1.7	0.26	2.21E-10	2.12E-09
rpmI	13008	1.4	0.22	5.62E-10	5.31E-09
cusC	1066	-2.3	0.40	9.05E-10	8.40E-09
fbaA	18388	-0.8	0.11	1.07E-09	9.82E-09
rpsE	16906	1.0	0.15	1.54E-09	1.39E-08
rpsD	28636	1.0	0.15	2.33E-09	2.08E-08

Supplementary Tables

malM	17555	-1.2	0.20	2.41E-09	2.14E-08
marA	352	1.2	0.19	3.64E-09	3.18E-08
cusB	625	-2.1	0.40	4.20E-09	3.64E-08
yihY	634	-1.1	0.17	4.29E-09	3.70E-08
ychH	2384	-1.2	0.21	5.49E-09	4.72E-08
pspD	528	1.7	0.31	6.65E-09	5.68E-08
fusA	49933	0.9	0.15	6.78E-09	5.75E-08
nlpD	12824	-1.0	0.15	7.11E-09	6.02E-08
yncJ	83	2.1	0.40	7.76E-09	6.50E-08
nadA	1153	-0.9	0.14	8.29E-09	6.92E-08
pspG	149	2.1	0.39	8.76E-09	7.28E-08
yoaD	185	-1.3	0.22	9.51E-09	7.86E-08
bcsG	1212	-0.7	0.12	9.88E-09	8.13E-08
yccA	6278	1.1	0.18	1.17E-08	9.55E-08
bcsZ	1197	-0.8	0.13	1.45E-08	1.17E-07
yhcN	133	2.6	0.52	1.60E-08	1.29E-07
rutA	47	2.3	0.45	2.02E-08	1.62E-07
copA	1327	-1.0	0.16	2.06E-08	1.65E-07
rpoA	40579	0.9	0.15	2.19E-08	1.75E-07
amtB	416	1.0	0.17	2.40E-08	1.90E-07
rseB	4776	-0.7	0.12	2.94E-08	2.30E-07
ydeH	375	1.0	0.18	2.97E-08	2.31E-07
rplQ	16582	1.1	0.19	3.68E-08	2.82E-07
secY	40080	0.9	0.16	3.79E-08	2.89E-07
cbpM	153	-1.3	0.23	6.71E-08	5.00E-07
puuR	1178	1.0	0.17	6.85E-08	5.08E-07
cpdB	3985	-0.7	0.12	8.07E-08	5.91E-07
priB	4398	1.0	0.17	9.66E-08	6.95E-07
ydeN	2197	-1.1	0.20	1.04E-07	7.42E-07
cusF	456	-2.3	0.50	1.24E-07	8.77E-07
rpsB	21686	0.8	0.14	1.97E-07	1.38E-06
ybhP	159	-1.3	0.26	2.90E-07	1.99E-06
yihX	900	-0.9	0.16	3.69E-07	2.50E-06
yjjM	411	-1.0	0.19	4.13E-07	2.79E-06
tnaA	578	-1.1	0.20	4.21E-07	2.84E-06
rpmJ	9629	1.1	0.20	4.60E-07	3.10E-06
degQ	1537	-0.7	0.12	4.76E-07	3.20E-06
csiD	628	1.4	0.28	5.77E-07	3.85E-06
yhbS	1702	-0.8	0.14	6.38E-07	4.21E-06
yhjQ	531	-0.8	0.15	6.97E-07	4.57E-06
rpsK	16121	0.8	0.15	7.57E-07	4.95E-06
rplO	5537	0.8	0.16	8.28E-07	5.39E-06
rpsM	18735	0.9	0.17	9.01E-07	5.83E-06
yfdX	64	1.6	0.36	9.86E-07	6.36E-06

Supplementary Tables

apaG	679	-0.8	0.15	1.31E-06	8.42E-06
tesB	1026	0.7	0.14	1.57E-06	1.00E-05
cspA	15339	1.3	0.28	1.77E-06	1.12E-05
ydeM	499	-0.8	0.17	2.34E-06	1.47E-05
cbrA	136	1.2	0.24	2.64E-06	1.65E-05
ucpA	2512	-0.7	0.14	3.18E-06	1.96E-05
yeeA	476	-0.7	0.14	3.23E-06	1.99E-05
ybhC	219	0.9	0.20	4.96E-06	2.98E-05
ptsN	1476	-0.7	0.13	4.96E-06	2.98E-05
rplY	4721	0.8	0.17	5.54E-06	3.31E-05
rplX	17953	0.8	0.16	6.77E-06	4.02E-05
yfhM	4193	-0.5	0.11	8.06E-06	4.77E-05
nuoE	3977	-0.7	0.14	8.21E-06	4.85E-05
sulA	1003	1.1	0.25	8.33E-06	4.91E-05
rplN	22506	0.6	0.13	8.92E-06	5.25E-05
hisJ	8288	-0.6	0.11	1.08E-05	6.29E-05
yjaH	1188	-0.7	0.14	1.10E-05	6.38E-05
rpmG	6402	1.3	0.29	1.17E-05	6.76E-05
yeiP	808	0.8	0.18	1.23E-05	7.07E-05
tsf	16322	0.7	0.15	1.25E-05	7.21E-05
infA	5014	1.2	0.29	1.62E-05	9.31E-05
yfaW	455	-1.0	0.23	1.77E-05	1.01E-04
eutM	454	-0.9	0.20	1.82E-05	1.04E-04
nadB	1144	-0.6	0.12	1.85E-05	1.05E-04
pgk	10948	-0.5	0.11	2.08E-05	1.18E-04
dctA	9063	-0.9	0.21	2.57E-05	1.43E-04
ybl88	307	0.8	0.18	2.67E-05	1.49E-04
cynX	298	1.0	0.24	2.81E-05	1.56E-04
xseB	536	-0.6	0.14	2.81E-05	1.56E-04
yibI	103	-1.1	0.27	2.95E-05	1.63E-04
erfK	712	-0.7	0.16	3.22E-05	1.77E-04
ybbA	451	0.7	0.14	3.59E-05	1.96E-04
qseC	464	0.9	0.20	3.74E-05	2.04E-04
tisB	4705	1.2	0.32	4.24E-05	2.31E-04
sgcE	151	-1.0	0.24	4.58E-05	2.48E-04
yceD	8588	0.8	0.19	4.95E-05	2.68E-04
ycfM	1120	-0.7	0.15	5.09E-05	2.75E-04
ybaV	251	-0.8	0.20	5.59E-05	3.01E-04
ydeA	144	1.1	0.26	5.70E-05	3.07E-04
ygdR	928	-1.1	0.29	5.78E-05	3.10E-04
yjgH	144	-1.0	0.24	5.83E-05	3.12E-04
pflB	29927	-0.6	0.14	6.30E-05	3.35E-04
ygaXY	76	1.4	0.38	6.69E-05	3.54E-04
ybl152	184	1.0	0.26	6.80E-05	3.59E-04

Supplementary Tables

pnuC	865	-0.8	0.19	7.21E-05	3.80E-04
efeU	384	-0.7	0.16	7.36E-05	3.87E-04
nuoF	10406	-0.6	0.14	7.68E-05	4.02E-04
eno	31928	-0.6	0.13	8.00E-05	4.17E-04
yeeY	949	-0.7	0.16	8.47E-05	4.40E-04
sodB	4860	0.6	0.14	8.71E-05	4.52E-04
yhfG	247	-0.7	0.18	8.75E-05	4.53E-04
pheT	7346	0.5	0.11	9.00E-05	4.65E-04
fdoG	5460	-0.6	0.13	9.72E-05	4.99E-04
prpB	653	-0.9	0.23	9.89E-05	5.06E-04
glpK	3097	-0.8	0.20	1.03E-04	5.24E-04
nuoB	8283	-0.6	0.14	1.06E-04	5.38E-04
rbsK	596	-0.6	0.15	1.07E-04	5.41E-04
bcsA	2134	-0.6	0.15	1.11E-04	5.61E-04
uspA	7362	-0.8	0.21	1.21E-04	6.08E-04
yiiD	1506	-0.6	0.13	1.26E-04	6.31E-04
phoB	538	0.7	0.17	1.40E-04	6.98E-04
topB	1134	-0.5	0.13	1.41E-04	7.04E-04
mltD	11417	-0.5	0.13	1.46E-04	7.23E-04
prfA	1613	-0.5	0.11	1.62E-04	8.04E-04
yfcH	2586	-0.6	0.14	1.69E-04	8.33E-04
ypfH	523	-0.6	0.14	1.72E-04	8.46E-04
yabI	825	-0.5	0.13	2.09E-04	1.02E-03
pyrD	669	0.6	0.16	2.10E-04	1.02E-03
yohD	368	-0.7	0.17	2.19E-04	1.06E-03
ppsR	715	-0.6	0.15	2.21E-04	1.07E-03
qseB	383	0.8	0.23	2.22E-04	1.08E-03
nuoI	3064	-0.6	0.17	2.32E-04	1.12E-03
pheA	5964	0.7	0.17	2.41E-04	1.16E-03
yaiY	70	-1.1	0.33	2.46E-04	1.19E-03
arnA	15248	-0.4	0.10	2.48E-04	1.19E-03
menD	603	-0.7	0.17	2.56E-04	1.23E-03
lhgO	380	0.8	0.23	2.67E-04	1.28E-03
pal	15621	0.7	0.19	2.69E-04	1.29E-03
hisQ	2413	-0.6	0.14	2.70E-04	1.29E-03
cbrB	109	1.0	0.28	2.84E-04	1.36E-03
yfdC	157	-1.1	0.32	2.92E-04	1.39E-03
rsmA	1303	-0.6	0.16	2.95E-04	1.40E-03
yjeS	468	-0.6	0.15	3.02E-04	1.43E-03
yeiG	987	-0.5	0.13	3.17E-04	1.50E-03
rbfA	928	0.6	0.14	3.17E-04	1.50E-03
rpsA	57957	0.6	0.15	3.38E-04	1.59E-03
mdoC	94	1.0	0.28	3.55E-04	1.67E-03
mazG	1185	-0.6	0.15	3.59E-04	1.68E-03

mrcA	1797	0.6	0.14	3.62E-04	1.69E-03
recN	894	0.8	0.23	3.69E-04	1.72E-03
minD	5039	-0.5	0.12	3.80E-04	1.77E-03
sieB	47	1.2	0.36	3.89E-04	1.81E-03
ylaB	429	-0.6	0.16	3.89E-04	1.81E-03
tufA	30008	0.6	0.15	3.97E-04	1.84E-03
hypD	230	-0.9	0.26	4.00E-04	1.85E-03
ravA	739	-0.5	0.14	4.60E-04	2.12E-03
gor	2437	-0.5	0.12	4.95E-04	2.27E-03
sgcR	257	-0.7	0.18	5.28E-04	2.41E-03
lipA	5552	-0.5	0.14	5.30E-04	2.41E-03
aaeA	143	0.8	0.23	5.33E-04	2.42E-03
yobB	530	0.8	0.23	5.38E-04	2.44E-03
relA	4194	-0.5	0.11	5.38E-04	2.44E-03
ybbP	710	0.6	0.15	5.55E-04	2.51E-03
grxA	256	1.0	0.32	5.80E-04	2.62E-03
marB	118	1.0	0.31	6.13E-04	2.76E-03
yhgA	94	1.2	0.38	6.83E-04	3.05E-03
rpmF	8539	0.8	0.23	7.34E-04	3.26E-03
yjhQ	157	-1.1	0.35	7.38E-04	3.27E-03
yjiN	293	-0.8	0.25	7.37E-04	3.27E-03
srlB	50	-1.1	0.36	7.88E-04	3.48E-03
csgG	119	0.8	0.25	8.25E-04	3.63E-03
ygiW	2134	1.0	0.34	8.46E-04	3.72E-03
atoC	494	-0.6	0.17	8.65E-04	3.80E-03
ompW	401	-0.8	0.23	8.87E-04	3.89E-03
cbrC	323	0.7	0.21	9.36E-04	4.09E-03
yhbJ	2129	-0.6	0.16	9.37E-04	4.10E-03
yedZ	107	-0.8	0.27	1.04E-03	4.52E-03
yqfA	678	-0.7	0.19	1.07E-03	4.64E-03
ygfA	612	-0.8	0.24	1.10E-03	4.74E-03
hemN	1818	-0.5	0.13	1.11E-03	4.78E-03
cynS	145	0.7	0.23	1.12E-03	4.82E-03
ygbI	572	-0.5	0.15	1.12E-03	4.82E-03
rimM	10463	0.6	0.17	1.20E-03	5.15E-03
nuoJ	3951	-0.6	0.17	1.26E-03	5.42E-03
yneH	536	0.6	0.19	1.27E-03	5.44E-03
yebT	1629	-0.5	0.12	1.30E-03	5.53E-03
nuoH	5804	-0.6	0.18	1.35E-03	5.75E-03
mqsR	45	1.1	0.41	1.37E-03	5.77E-03
sugE	844	-0.6	0.20	1.41E-03	5.94E-03
infB	12783	0.4	0.12	1.42E-03	5.99E-03
plsC	713	0.6	0.17	1.46E-03	6.12E-03
rbsR	365	-0.6	0.19	1.46E-03	6.12E-03

Supplementary Tables

dsbA	2809	0.6	0.17	1.47E-03	6.15E-03
nuoG	16799	-0.6	0.16	1.49E-03	6.26E-03
ptrB	774	-0.6	0.17	1.54E-03	6.47E-03
nuoC	9896	-0.6	0.18	1.58E-03	6.63E-03
rseC	1106	-0.5	0.16	1.59E-03	6.65E-03
ybl114	2516	-0.6	0.20	1.60E-03	6.69E-03
yhfK	1177	-0.5	0.14	1.61E-03	6.69E-03
feoB	828	-0.5	0.13	1.64E-03	6.82E-03
ybhL	1377	-0.6	0.20	1.66E-03	6.89E-03
queD	482	0.7	0.21	1.69E-03	7.00E-03
hemL	2731	-0.5	0.13	1.69E-03	7.00E-03
mscL	1353	-0.9	0.31	1.74E-03	7.18E-03
nuoL	9557	-0.6	0.17	1.78E-03	7.34E-03
nagB	864	-0.6	0.17	1.79E-03	7.39E-03
pmrD	194	-0.8	0.26	1.81E-03	7.44E-03
ghrB	1651	-0.5	0.13	1.81E-03	7.45E-03
mrdA	975	0.6	0.18	1.86E-03	7.65E-03
abgR	305	-0.6	0.19	1.89E-03	7.75E-03
uspE	6495	-0.6	0.18	1.92E-03	7.87E-03
ydgI	155	0.8	0.29	1.94E-03	7.93E-03
ybiS	1960	0.5	0.15	1.95E-03	7.97E-03
gltJ	3404	-0.6	0.18	1.95E-03	7.97E-03
ychN	2567	-0.6	0.20	1.96E-03	7.97E-03
glpA	79	1.0	0.37	1.96E-03	7.98E-03
cmr	447	0.6	0.19	2.02E-03	8.19E-03
proC	1105	-0.5	0.15	2.02E-03	8.20E-03
purM	3756	-0.5	0.14	2.04E-03	8.25E-03
yebK	1305	-0.5	0.14	2.06E-03	8.31E-03
polA	3641	-0.5	0.15	2.09E-03	8.42E-03
hisP	2644	-0.5	0.13	2.10E-03	8.45E-03
crr	26128	-0.5	0.17	2.11E-03	8.47E-03
yccX	261	-0.9	0.32	2.21E-03	8.88E-03
arnE	1438	-0.6	0.17	2.22E-03	8.89E-03
aphA	831	-0.5	0.15	2.29E-03	9.15E-03
prpD	832	-0.9	0.31	2.29E-03	9.17E-03
dtd	648	-0.6	0.19	2.31E-03	9.22E-03
kefA	2074	0.5	0.13	2.36E-03	9.42E-03
minC	1794	-0.5	0.13	2.42E-03	9.61E-03
gloB	913	-0.5	0.15	2.43E-03	9.63E-03
uxaC	581	-0.7	0.23	2.44E-03	9.65E-03
phnD	45	1.1	0.41	2.43E-03	9.65E-03
rpsU	10401	0.8	0.28	2.45E-03	9.72E-03
glpF	1052	-0.6	0.18	2.52E-03	9.95E-03
glnK	49	1.3	0.56	2.55E-03	1.00E-02

cusS	384	-0.8	0.27	2.64E-03	1.03E-02
ybjH	211	0.8	0.27	2.66E-03	1.04E-02
rlmG	363	0.6	0.20	2.69E-03	1.05E-02
speB	1069	-0.4	0.13	2.79E-03	1.09E-02
cpdA	3390	-0.6	0.19	2.80E-03	1.09E-02
ydcP	949	0.5	0.14	2.85E-03	1.10E-02
yigA	1059	-0.4	0.13	2.85E-03	1.11E-02
ispF	554	-0.5	0.15	2.95E-03	1.14E-02
sstT	1403	-0.8	0.28	2.95E-03	1.14E-02
dgoR	345	-0.5	0.17	2.96E-03	1.14E-02
yhcM	1470	0.5	0.14	2.96E-03	1.14E-02
grpE	6215	0.8	0.29	2.97E-03	1.14E-02
yhbY	2132	0.7	0.24	3.04E-03	1.17E-02
yaeR	350	-0.5	0.17	3.12E-03	1.20E-02
lafU	42	1.1	0.44	3.21E-03	1.23E-02
yjhP	300	-0.9	0.35	3.33E-03	1.27E-02
thiI	1390	0.4	0.13	3.45E-03	1.31E-02
dcuC	105	0.8	0.29	3.58E-03	1.35E-02
eutK	203	-0.6	0.21	3.59E-03	1.36E-02
ydfX	49	1.2	0.54	3.61E-03	1.36E-02
pdxA	1282	-0.4	0.13	3.70E-03	1.39E-02
yeeN	649	0.7	0.25	3.73E-03	1.40E-02
chpA	458	-0.7	0.25	3.73E-03	1.40E-02
ybl115	1371	-0.6	0.23	3.78E-03	1.42E-02
cusR	525	-0.7	0.23	3.80E-03	1.42E-02
arnC	6783	-0.5	0.14	3.90E-03	1.46E-02
xdhB	127	-0.7	0.27	3.91E-03	1.46E-02
yiaJ	467	0.6	0.19	3.92E-03	1.46E-02
ydcA	376	-0.5	0.18	3.96E-03	1.47E-02
rhaR	484	-0.6	0.19	4.08E-03	1.52E-02
ybgF	5731	0.5	0.17	4.14E-03	1.54E-02
maeB	10581	-0.4	0.11	4.18E-03	1.55E-02
yjbM	76	1.2	0.53	4.28E-03	1.59E-02
rpe	2089	-0.4	0.11	4.33E-03	1.60E-02
phnP	131	-0.8	0.30	4.34E-03	1.60E-02
yecN	831	-0.7	0.23	4.36E-03	1.61E-02
rpsT	8269	0.7	0.27	4.47E-03	1.65E-02
iscS	18637	0.4	0.12	4.48E-03	1.65E-02
dcuS	671	-0.5	0.18	4.54E-03	1.67E-02
yeeL_1	33	1.2	0.56	4.54E-03	1.67E-02
dinI	472	0.8	0.31	4.58E-03	1.68E-02
agaD	38	1.1	0.47	4.62E-03	1.69E-02
ompA	29917	0.5	0.16	4.67E-03	1.71E-02
nuoM	7312	-0.4	0.13	4.78E-03	1.75E-02

phr	427	-0.7	0.24	4.90E-03	1.79E-02
gst	1733	-0.6	0.22	4.98E-03	1.81E-02
nudG	175	0.7	0.25	5.15E-03	1.87E-02
kdgR	1913	-0.4	0.13	5.15E-03	1.87E-02
csgB	30	1.1	0.51	5.16E-03	1.87E-02
purH	3996	-0.4	0.14	5.21E-03	1.89E-02
asd	8595	-0.4	0.14	5.22E-03	1.89E-02
hsdM	1169	0.4	0.12	5.37E-03	1.94E-02
uidR	451	-0.6	0.21	5.47E-03	1.98E-02
glpG	661	0.5	0.15	5.49E-03	1.98E-02
azuC	27	1.3	0.69	5.60E-03	2.02E-02
yfiB	165	-0.8	0.30	5.65E-03	2.04E-02
yhjE	2649	0.9	0.37	5.78E-03	2.08E-02
eutL	212	-0.6	0.24	5.89E-03	2.12E-02
cmoA	465	-0.5	0.19	5.90E-03	2.12E-02
cutA	430	-0.5	0.15	5.93E-03	2.13E-02
exbD	185	-0.6	0.23	5.93E-03	2.13E-02
mlaC	2121	-0.4	0.12	5.93E-03	2.13E-02
tolQ	1692	0.5	0.15	5.96E-03	2.13E-02
hemX	2734	-0.4	0.13	6.00E-03	2.14E-02
mdtH	1086	0.5	0.15	6.12E-03	2.18E-02
serA	11430	0.6	0.21	6.14E-03	2.18E-02
ubiF	765	-0.4	0.13	6.21E-03	2.21E-02
rplU	6353	0.6	0.20	6.28E-03	2.23E-02
yaeJ	188	-0.6	0.20	6.35E-03	2.25E-02
yieH	468	0.5	0.19	6.53E-03	2.30E-02
nuoK	1249	-0.5	0.18	6.52E-03	2.30E-02
yjeE	330	-0.5	0.18	6.61E-03	2.33E-02
bet	241	0.9	0.38	6.71E-03	2.36E-02
rbsA	100	0.8	0.33	6.82E-03	2.39E-02
rhsD	199	0.8	0.33	6.95E-03	2.43E-02
yfiC	529	0.4	0.14	7.06E-03	2.46E-02
phoR	477	0.5	0.20	7.13E-03	2.48E-02
ybjD	1052	-0.4	0.13	7.17E-03	2.49E-02
yebG	765	0.7	0.29	7.18E-03	2.49E-02
ycgV	343	0.6	0.24	7.20E-03	2.50E-02
cybC	1690	-0.5	0.17	7.23E-03	2.50E-02
metH	10046	-0.4	0.12	7.23E-03	2.50E-02
mppA	1087	-0.4	0.13	7.29E-03	2.52E-02
yqcA	888	-0.5	0.17	7.50E-03	2.58E-02
feaB	742	-0.4	0.15	7.55E-03	2.60E-02
ftsN	1660	-0.5	0.18	7.59E-03	2.61E-02
rlmE	3304	0.5	0.17	7.91E-03	2.71E-02
xylH	193	-0.6	0.22	7.94E-03	2.72E-02

Supplementary Tables

yceJ	44	0.9	0.42	8.11E-03	2.78E-02
ynfK	832	-0.4	0.13	8.18E-03	2.79E-02
nuoA	6154	-0.4	0.12	8.19E-03	2.80E-02
rpmA	11122	0.6	0.25	8.39E-03	2.86E-02
eutQ	136	-0.9	0.38	8.46E-03	2.87E-02
ydiF	82	1.0	0.53	8.54E-03	2.89E-02
atoE	77	1.0	0.47	8.60E-03	2.91E-02
yajL	585	-0.4	0.16	8.76E-03	2.96E-02
ampD	440	-0.5	0.18	8.83E-03	2.98E-02
amiB	1190	-0.4	0.14	9.00E-03	3.03E-02
pulD	134	1.0	0.49	9.03E-03	3.04E-02
yajD	929	0.5	0.17	9.11E-03	3.06E-02
glk	2284	-0.4	0.12	9.12E-03	3.06E-02
iscU	7383	0.4	0.13	9.13E-03	3.07E-02
amn	1951	-0.4	0.14	9.19E-03	3.08E-02
yfaP	132	0.7	0.27	9.25E-03	3.10E-02
yoaC	402	-0.7	0.32	9.36E-03	3.13E-02
yifN	45	1.1	0.65	9.37E-03	3.13E-02
aaeB	251	0.6	0.26	9.44E-03	3.15E-02
suhB	1085	0.6	0.22	9.50E-03	3.17E-02
yeiT	644	-0.5	0.17	9.57E-03	3.19E-02
gntT	15492	0.5	0.18	9.57E-03	3.19E-02
pulG-3	30	1.1	0.62	9.63E-03	3.20E-02
aec79	72	1.0	0.57	9.84E-03	3.27E-02
dnaT	487	-0.4	0.15	9.94E-03	3.29E-02
hypC	38	-0.9	0.47	9.95E-03	3.29E-02
orf28	71	1.0	0.52	1.00E-02	3.31E-02
yccK	101	0.6	0.27	1.01E-02	3.34E-02
yeaY	2033	-0.5	0.18	1.03E-02	3.39E-02
ydhZ	109	-0.8	0.37	1.03E-02	3.39E-02
dcuD	140	0.9	0.45	1.06E-02	3.48E-02
xanP	2461	-0.4	0.12	1.07E-02	3.52E-02
yfhL	76	0.7	0.31	1.07E-02	3.52E-02
yjjN	122	0.9	0.45	1.09E-02	3.56E-02
lldR	207	-0.6	0.25	1.09E-02	3.56E-02
aroK	4291	0.5	0.19	1.10E-02	3.59E-02
yagQ	191	-0.6	0.23	1.10E-02	3.59E-02
ybiO	422	-0.5	0.18	1.12E-02	3.67E-02
umuC	252	0.6	0.26	1.13E-02	3.69E-02
yaiI	362	0.5	0.18	1.13E-02	3.69E-02
lpxP	1025	0.6	0.24	1.14E-02	3.72E-02
ndk	5283	0.6	0.24	1.15E-02	3.73E-02
yhjV	139	0.7	0.34	1.15E-02	3.74E-02
rimP	1737	0.4	0.15	1.17E-02	3.78E-02

hsdS_1	198	0.6	0.25	1.17E-02	3.78E-02
melA	237	-0.6	0.22	1.18E-02	3.81E-02
yraP	2100	-0.4	0.14	1.18E-02	3.81E-02
ybl172	50	1.1	0.66	1.18E-02	3.82E-02
yfcL	744	0.5	0.21	1.19E-02	3.83E-02
ftnA	6404	0.5	0.20	1.19E-02	3.83E-02
ybl153	126	0.7	0.30	1.19E-02	3.83E-02
yjgF	2808	-0.6	0.27	1.19E-02	3.83E-02
yjbQ	280	-0.5	0.21	1.20E-02	3.83E-02
atoA	28	1.1	0.63	1.20E-02	3.83E-02
emrD	54	1.0	0.52	1.20E-02	3.85E-02
ybl65	25	1.2	0.85	1.20E-02	3.85E-02
fre	2322	-0.4	0.15	1.21E-02	3.85E-02
yncI	69	0.9	0.48	1.21E-02	3.87E-02
ltaE	2050	-0.4	0.13	1.23E-02	3.91E-02
yieK	154	0.7	0.32	1.23E-02	3.92E-02
arnF	856	-0.4	0.16	1.23E-02	3.92E-02
hslR	486	0.5	0.21	1.26E-02	4.02E-02
prs	7893	-0.4	0.17	1.27E-02	4.03E-02
yhhX	1237	-0.4	0.14	1.29E-02	4.09E-02
sthA	6272	-0.5	0.22	1.32E-02	4.19E-02
pyrF	1157	0.4	0.16	1.33E-02	4.21E-02
prpE	951	-0.6	0.26	1.34E-02	4.22E-02
yfeO	355	-0.5	0.19	1.34E-02	4.23E-02
ybl116	515	-0.6	0.25	1.34E-02	4.23E-02
eutT	151	-0.7	0.34	1.39E-02	4.37E-02
ybl154	206	0.6	0.29	1.40E-02	4.39E-02
ral	412	0.8	0.40	1.40E-02	4.39E-02
ygjK	136	0.8	0.40	1.40E-02	4.39E-02
nanK	282	-0.5	0.18	1.40E-02	4.41E-02
yrbN	35	0.8	0.42	1.41E-02	4.41E-02
ynfM	176	0.5	0.23	1.41E-02	4.41E-02
hslO	1307	0.4	0.15	1.44E-02	4.51E-02
endA	58	0.8	0.37	1.45E-02	4.53E-02
yiiM	626	-0.6	0.24	1.46E-02	4.55E-02
yigE	80	0.7	0.34	1.47E-02	4.57E-02
nadD	398	-0.4	0.16	1.48E-02	4.60E-02
ybl37	59	1.0	0.58	1.50E-02	4.66E-02
acnB	61116	-0.4	0.15	1.50E-02	4.67E-02
atoB	132	0.8	0.37	1.51E-02	4.70E-02
truB	1328	0.4	0.17	1.52E-02	4.73E-02
rpoN	4839	-0.4	0.15	1.56E-02	4.84E-02
yajC	5327	0.5	0.21	1.56E-02	4.85E-02
glvC	116	0.9	0.49	1.57E-02	4.85E-02

yjbR	919	-0.4	0.15	1.59E-02	4.91E-02
yhbV	78	0.8	0.38	1.60E-02	4.96E-02
ompN	57	0.9	0.49	1.62E-02	5.00E-02

Table S5: Differentially expressed genes in H<oFabx> during fed-batch cultivation after 2 h of induction relative to the sample drawn immediately before induction of Fabx expression. Genes also differentially expressed in wildtype HMS174(DE3) were excluded.

Gene	baseMean	log2FoldChange	lfcSE	pvalue	padj
rstA	8998	4.9	0.17	8.63E-170	7.08E-167
rstB	9164	4.7	0.19	2.30E-130	1.57E-127
chaA	2828	3.9	0.17	2.81E-115	1.64E-112
ibpB	18985	8.5	0.42	1.37E-88	6.99E-86
slyB	26625	3.8	0.19	1.16E-85	5.26E-83
ybjX	3973	4.2	0.21	3.80E-85	1.56E-82
gmr	1616	3.4	0.17	8.04E-85	2.99E-82
mgrB	507	4.7	0.25	2.33E-78	7.95E-76
pgpC	1396	3.1	0.16	3.74E-77	1.09E-74
ycfS	2323	3.1	0.16	3.64E-77	1.09E-74
tqsA	723	4.1	0.22	4.88E-74	1.33E-71
mgtA	8195	4.6	0.25	5.51E-74	1.41E-71
phoQ	5006	3.2	0.18	1.93E-68	4.65E-66
phoP	5928	3.1	0.19	1.88E-58	4.29E-56
yebO	5628	4.3	0.27	4.22E-58	9.10E-56
ibpA	16281	5.7	0.36	7.85E-57	1.61E-54
hslU	8859	2.8	0.18	6.82E-55	1.33E-52
hslO	3848	2.9	0.19	3.34E-51	6.22E-49
ybjG	3213	4.8	0.32	4.92E-51	8.76E-49
ycjF	2326	3.2	0.21	9.93E-51	1.69E-48
grpE	18272	2.9	0.20	8.48E-47	1.39E-44
ydeH	934	2.9	0.20	1.17E-46	1.85E-44
yneM	5995	5.3	0.38	1.56E-45	2.37E-43
mutM	940	2.6	0.19	2.96E-42	4.32E-40
ydeT	269	3.3	0.24	6.77E-42	9.25E-40
asr	483	4.3	0.32	8.07E-42	1.07E-39
ybeZ	4011	2.4	0.17	5.51E-41	7.05E-39
hslR	1563	2.9	0.21	1.94E-40	2.34E-38
cpxP	16132	3.2	0.24	2.59E-40	2.95E-38
maeA	8177	2.3	0.17	4.64E-39	5.14E-37
tadA	608	3.0	0.23	5.60E-38	6.04E-36
dacC	3508	2.7	0.20	7.14E-38	7.51E-36
yebE	2411	3.0	0.23	1.33E-36	1.36E-34
hslV	2042	2.6	0.21	1.27E-35	1.26E-33
ybbN	5633	2.4	0.19	2.29E-34	2.23E-32

Supplementary Tables

ybeY	2230	2.2	0.18	4.09E-34	3.90E-32
hemL	4374	2.3	0.19	3.45E-32	3.14E-30
mgtL	93	3.9	0.34	2.51E-31	2.24E-29
tus	549	2.8	0.24	4.01E-30	3.50E-28
ybeX	3781	2.0	0.17	1.98E-28	1.69E-26
mlaF	4445	2.2	0.20	2.47E-28	2.07E-26
ftsH	26943	1.9	0.16	3.13E-28	2.57E-26
groS	15203	2.5	0.23	1.48E-27	1.19E-25
mlaD	2232	1.9	0.17	5.84E-26	4.60E-24
ygiV	300	2.9	0.28	1.26E-24	9.73E-23
miaA	14610	2.0	0.19	1.36E-24	1.03E-22
hflK	6542	1.8	0.18	1.29E-21	9.43E-20
mlaE	2063	1.8	0.19	1.82E-20	1.26E-18
sdaA	1025	2.6	0.28	3.43E-20	2.34E-18
hflX	6898	1.5	0.16	4.11E-20	2.76E-18
csgG	826	-1.7	0.19	8.31E-20	5.49E-18
clpB	33651	3.1	0.34	1.21E-19	7.88E-18
prlC	6115	1.8	0.20	3.60E-19	2.30E-17
ybeD	3101	2.2	0.25	7.08E-19	4.46E-17
hflC	5220	1.6	0.17	1.05E-18	6.55E-17
dnaJ	13604	2.6	0.30	1.17E-18	7.14E-17
ampH	2155	2.6	0.30	1.49E-18	8.93E-17
htpG	31376	3.1	0.35	1.50E-18	8.93E-17
htpX	20079	2.4	0.27	3.57E-18	2.06E-16
groL	106509	2.3	0.27	3.75E-18	2.14E-16
clcA	1123	1.5	0.17	7.09E-18	3.97E-16
mlaC	4981	1.7	0.19	7.17E-18	3.97E-16
yccA	22744	1.7	0.20	1.32E-17	7.19E-16
ydeR	68	2.6	0.31	1.69E-17	9.14E-16
yhdV	122	2.3	0.28	2.21E-17	1.17E-15
yfeK	275	-2.0	0.23	3.08E-17	1.62E-15
yjcH	7044	-1.4	0.17	7.99E-17	4.15E-15
dnaK	85601	2.9	0.36	2.76E-16	1.41E-14
yagU	295	-1.7	0.21	5.84E-16	2.95E-14
lpxT	303	2.9	0.36	6.01E-16	3.00E-14
yobA	236	1.8	0.23	1.02E-15	5.04E-14
ompA	106766	1.8	0.22	2.09E-15	1.02E-13
lnt	1141	1.8	0.23	2.70E-15	1.30E-13
ompC	135107	1.5	0.20	3.45E-15	1.64E-13
queE	844	1.9	0.24	3.53E-15	1.66E-13
argF	693	2.0	0.26	5.18E-14	2.39E-12
ydeS	84	2.1	0.28	8.15E-14	3.67E-12
fabR	3593	1.4	0.19	1.29E-13	5.69E-12
pstS	2051	2.2	0.30	1.29E-13	5.69E-12

mlaB	2840	1.5	0.20	1.58E-13	6.90E-12
csgF	226	-2.0	0.27	1.71E-13	7.39E-12
yqjA	1340	1.3	0.18	2.27E-13	9.58E-12
ftnB	2148	1.3	0.18	4.73E-13	1.98E-11
livJ	37084	-1.3	0.18	5.01E-13	2.07E-11
deoR	1664	1.6	0.23	5.38E-13	2.18E-11
rhaB	140	-1.7	0.24	5.35E-13	2.18E-11
sstT	6215	-1.3	0.19	6.35E-13	2.55E-11
gadW	375	1.5	0.22	7.56E-13	3.01E-11
yodB	126	1.8	0.25	7.65E-13	3.01E-11
matA	86	1.8	0.26	8.62E-13	3.36E-11
amiC	1774	1.7	0.24	9.76E-13	3.77E-11
iraM	147	1.6	0.23	1.10E-12	4.18E-11
matB	372	1.7	0.24	1.30E-12	4.88E-11
gatC	79323	-1.5	0.21	1.48E-12	5.49E-11
spy	1311	1.8	0.26	1.66E-12	6.13E-11
ydeN	1090	-2.0	0.28	1.68E-12	6.13E-11
glcB	39299	-1.3	0.19	2.52E-12	9.14E-11
lon	25021	1.9	0.28	2.82E-12	1.01E-10
hscA	4128	1.4	0.20	3.22E-12	1.14E-10
yrfG	671	1.2	0.18	3.33E-12	1.16E-10
topA	18260	1.2	0.17	5.06E-12	1.76E-10
gatB	16541	-1.1	0.15	6.86E-12	2.36E-10
rsmJ	627	1.2	0.18	9.15E-12	3.13E-10
yobB	512	1.9	0.28	9.31E-12	3.15E-10
mzrA	557	1.2	0.18	1.03E-11	3.45E-10
yecD	1066	1.7	0.25	1.29E-11	4.24E-10
nadD	649	1.8	0.28	3.84E-11	1.24E-09
rpoE	11798	-2.1	0.33	4.10E-11	1.31E-09
fxsA	2551	2.4	0.38	4.21E-11	1.33E-09
matC	40	2.3	0.36	4.19E-11	1.33E-09
yeaD	689	1.2	0.18	7.45E-11	2.29E-09
pstC	650	1.5	0.23	7.86E-11	2.40E-09
rhaM	39	-2.2	0.35	9.74E-11	2.93E-09
rhaT	452	-1.6	0.26	1.08E-10	3.24E-09
carA	265	1.7	0.28	1.10E-10	3.26E-09
mfd	5483	1.4	0.23	1.27E-10	3.71E-09
rseB	4948	-1.0	0.16	2.04E-10	5.92E-09
yciM	2145	1.0	0.16	2.28E-10	6.57E-09
yncJ	65	2.2	0.36	3.59E-10	1.02E-08
yjhC	883	-1.8	0.30	5.15E-10	1.45E-08
ydeQ	127	1.5	0.25	6.28E-10	1.76E-08
ybaP	198	1.5	0.26	1.06E-09	2.93E-08
apbE	478	1.7	0.29	1.07E-09	2.95E-08

yjhB	329	-1.2	0.20	1.55E-09	4.21E-08
fdx	1347	1.2	0.21	2.01E-09	5.43E-08
nrdF	424	-1.2	0.21	2.66E-09	7.12E-08
clpP	5717	1.1	0.20	3.32E-09	8.73E-08
pspB	223	1.6	0.29	4.15E-09	1.07E-07
ygiM	2890	-1.0	0.18	4.26E-09	1.09E-07
glcA	6367	-1.0	0.17	4.76E-09	1.21E-07
ygiW	1062	1.7	0.31	6.47E-09	1.63E-07
rhaD	63	-1.7	0.31	8.53E-09	2.13E-07
ygiA	130	1.6	0.30	1.00E-08	2.48E-07
gldA	968	-1.2	0.23	1.24E-08	3.04E-07
pspA	1662	1.5	0.28	1.31E-08	3.18E-07
gabP	415	-1.5	0.29	1.40E-08	3.35E-07
tolC	9563	1.2	0.23	1.55E-08	3.69E-07
yceJ	97	1.3	0.24	1.61E-08	3.81E-07
mdtJ	52	1.8	0.35	1.93E-08	4.51E-07
dsbA	4261	1.1	0.22	2.45E-08	5.69E-07
rlmE	8161	1.4	0.27	2.65E-08	6.11E-07
secA	10562	0.9	0.17	3.96E-08	8.96E-07
ilvX	235	-1.9	0.39	5.02E-08	1.12E-06
dppA	43426	-0.9	0.17	5.42E-08	1.20E-06
gabT	1508	-1.1	0.21	7.40E-08	1.63E-06
asnC	112	1.4	0.29	8.07E-08	1.77E-06
metE	19742	-1.4	0.29	8.79E-08	1.90E-06
cobC	286	1.5	0.31	9.53E-08	2.04E-06
ychH	6923	-2.2	0.47	1.02E-07	2.18E-06
yecE	673	1.9	0.40	1.10E-07	2.33E-06
ldhA	1519	1.4	0.29	1.11E-07	2.34E-06
yfeS	442	-1.1	0.22	1.35E-07	2.84E-06
kgtP	41349	-1.3	0.28	1.66E-07	3.46E-06
cycA	16752	-0.8	0.15	1.77E-07	3.66E-06
ydgU	17	2.5	0.55	1.82E-07	3.73E-06
yciC	1063	0.9	0.18	1.86E-07	3.80E-06
osmB	568	1.1	0.24	1.88E-07	3.82E-06
sra	9358	-1.3	0.27	3.07E-07	6.19E-06
cstA	79317	-1.3	0.28	3.13E-07	6.23E-06
potD	12050	-1.1	0.24	3.13E-07	6.23E-06
ygiB	6342	0.9	0.18	4.57E-07	8.96E-06
yafD	1635	0.9	0.19	4.63E-07	9.03E-06
gatA	31064	-0.8	0.16	4.98E-07	9.67E-06
alx	230	1.1	0.25	5.75E-07	1.11E-05
hupA	24693	-1.0	0.22	6.19E-07	1.19E-05
cybB	817	0.8	0.17	6.38E-07	1.22E-05
hemF	553	1.6	0.36	6.36E-07	1.22E-05

yhdN	602	1.3	0.30	6.79E-07	1.29E-05
nanC	116	-1.1	0.23	6.98E-07	1.32E-05
srlD	409	-1.1	0.25	7.10E-07	1.33E-05
ybaO	180	1.1	0.24	8.74E-07	1.63E-05
torY	89	-1.3	0.31	9.34E-07	1.73E-05
holA	1102	1.1	0.26	9.42E-07	1.74E-05
yebZ	279	1.3	0.31	1.09E-06	1.99E-05
gapA	30392	1.6	0.37	1.11E-06	2.02E-05
borD	32	2.0	0.49	1.21E-06	2.19E-05
nagA	1629	0.9	0.19	1.20E-06	2.19E-05
amiA	794	1.3	0.32	1.35E-06	2.44E-05
agp	7683	-1.0	0.21	1.39E-06	2.50E-05
tusE	1020	2.0	0.48	1.54E-06	2.76E-05
metC	2916	-1.1	0.25	1.67E-06	2.96E-05
srlA	272	-1.2	0.29	1.73E-06	3.06E-05
iscX	920	1.2	0.29	1.81E-06	3.18E-05
phoB	422	0.9	0.21	1.96E-06	3.42E-05
yihN	233	-1.0	0.23	2.04E-06	3.56E-05
hha	558	1.3	0.33	2.37E-06	4.10E-05
yeaY	3567	-1.7	0.44	2.83E-06	4.85E-05
ydcW	1327	-1.2	0.30	2.85E-06	4.86E-05
lhgO	714	-0.8	0.17	2.93E-06	4.98E-05
mprA	985	1.1	0.26	2.97E-06	5.02E-05
zntR	1397	1.3	0.34	3.30E-06	5.56E-05
hfq	8922	0.8	0.17	3.39E-06	5.69E-05
cydA	10420	-0.9	0.21	3.44E-06	5.75E-05
gabD	901	-0.8	0.18	3.67E-06	6.11E-05
eamA	884	-1.0	0.23	3.97E-06	6.59E-05
csgA	160	-1.3	0.34	4.17E-06	6.89E-05
ycfD	2982	0.7	0.16	4.55E-06	7.46E-05
tatC	2295	1.6	0.43	5.65E-06	9.18E-05
acrD	484	0.8	0.18	5.84E-06	9.46E-05
malE	5876	-1.1	0.27	6.07E-06	9.79E-05
pepE	1382	-0.8	0.18	6.45E-06	1.04E-04
ygfF	196	-1.1	0.28	6.50E-06	1.04E-04
ydeJ	74	1.1	0.27	6.84E-06	1.09E-04
ybgE	2312	-1.1	0.28	6.88E-06	1.09E-04
fumC	12729	-1.0	0.25	7.71E-06	1.22E-04
yfaW	710	-1.0	0.26	8.16E-06	1.28E-04
sseB	985	0.8	0.19	8.61E-06	1.35E-04
pliG	1022	-0.8	0.20	1.07E-05	1.67E-04
ybgT	1458	-1.2	0.34	1.14E-05	1.77E-04
srlE	327	-1.0	0.27	1.19E-05	1.83E-04
ydcH	4629	-1.2	0.33	1.19E-05	1.83E-04

lipB	544	0.9	0.24	1.22E-05	1.87E-04
gatD	21411	-0.7	0.17	1.32E-05	2.02E-04
pspD	133	1.2	0.33	1.33E-05	2.03E-04
cydB	7009	-1.1	0.29	1.38E-05	2.10E-04
ycaC	636	-1.2	0.34	1.43E-05	2.16E-04
cutC	2981	-1.6	0.47	1.45E-05	2.18E-04
thiH	4206	-0.9	0.22	1.47E-05	2.20E-04
pmrD	1035	1.0	0.25	1.48E-05	2.21E-04
yiiS	216	-1.0	0.27	1.69E-05	2.51E-04
arfA	377	1.2	0.33	1.84E-05	2.72E-04
macB	1134	0.9	0.23	2.01E-05	2.94E-04
folP	618	0.8	0.19	2.11E-05	3.07E-04
aphA	3101	-0.8	0.19	2.13E-05	3.08E-04
cheW	2598	-1.0	0.28	2.13E-05	3.08E-04
ytfQ	2370	-0.8	0.20	2.15E-05	3.10E-04
thiS	507	-0.9	0.22	2.24E-05	3.21E-04
mdoD	4967	0.8	0.19	2.30E-05	3.29E-04
aceB	59445	-0.7	0.16	2.60E-05	3.69E-04
rpoH	4481	0.9	0.23	2.60E-05	3.69E-04
yfiD	1487	0.8	0.21	2.82E-05	3.98E-04
yiaD	225	1.4	0.42	3.04E-05	4.24E-04
dppF	1858	-1.0	0.27	3.03E-05	4.24E-04
glnH	61982	-0.8	0.19	3.04E-05	4.24E-04
mdtI	71	1.0	0.26	3.14E-05	4.36E-04
add	872	0.8	0.19	3.25E-05	4.50E-04
tatD	1219	0.9	0.23	3.41E-05	4.70E-04
aceA	107622	-0.8	0.19	3.42E-05	4.70E-04
argG	3153	1.0	0.30	3.55E-05	4.86E-04
ycjZ	315	0.8	0.20	3.61E-05	4.94E-04
cysI	3441	-0.6	0.15	3.72E-05	5.06E-04
mqsR	376	-1.1	0.33	3.94E-05	5.35E-04
ea8.5	691	-0.9	0.25	4.08E-05	5.51E-04
xdhD	1178	-0.8	0.22	4.23E-05	5.70E-04
yjeT	119	0.9	0.26	4.45E-05	5.96E-04
pstA	513	0.9	0.26	4.48E-05	5.98E-04
ybbA	599	0.9	0.23	4.57E-05	6.08E-04
rcnB	994	1.0	0.29	4.99E-05	6.61E-04
acs	56241	-0.8	0.20	5.12E-05	6.76E-04
dcp	2977	-0.7	0.16	5.69E-05	7.50E-04
ynaE	26	1.7	0.60	6.01E-05	7.86E-04
ppiA	1557	1.1	0.36	6.04E-05	7.88E-04
ompF	328854	-0.9	0.26	6.15E-05	8.01E-04
rhtB	782	-0.9	0.25	6.46E-05	8.38E-04
ybbM	176	0.9	0.28	6.80E-05	8.77E-04

ygiC	7224	0.7	0.17	6.90E-05	8.85E-04
ytfR	749	-0.9	0.24	7.04E-05	8.99E-04
rseC	958	-0.7	0.20	7.20E-05	9.16E-04
dcyD	2231	-0.8	0.22	7.25E-05	9.19E-04
cusF	47	1.0	0.32	7.38E-05	9.33E-04
torZ	472	-0.9	0.25	7.62E-05	9.60E-04
rdoA	2758	0.7	0.17	7.64E-05	9.60E-04
yahO	677	-1.2	0.38	8.29E-05	1.04E-03
uspA	24777	-0.9	0.28	8.77E-05	1.10E-03
mdfA	306	0.7	0.20	9.63E-05	1.20E-03
ydeP	396	0.7	0.20	9.72E-05	1.20E-03
yebS	290	0.7	0.19	1.01E-04	1.24E-03
ygbE	534	0.7	0.17	1.01E-04	1.25E-03
malP	685	-0.7	0.18	1.11E-04	1.36E-03
aroL	716	0.8	0.23	1.12E-04	1.37E-03
yfgG	363	1.1	0.39	1.13E-04	1.38E-03
uspD	686	-1.0	0.32	1.13E-04	1.38E-03
flu	1173	-0.8	0.22	1.18E-04	1.42E-03
argT	32982	-0.8	0.22	1.20E-04	1.44E-03
ahpC	11736	-0.7	0.20	1.23E-04	1.47E-03
yfhH	284	0.7	0.18	1.24E-04	1.48E-03
clpX	9515	0.8	0.21	1.26E-04	1.49E-03
pspC	252	1.0	0.33	1.32E-04	1.56E-03
ybhB	551	0.8	0.24	1.35E-04	1.60E-03
melA	403	-0.7	0.19	1.36E-04	1.60E-03
yjfY	57	1.0	0.31	1.38E-04	1.61E-03
hipA	338	0.7	0.20	1.39E-04	1.62E-03
fucO	2575	-0.9	0.26	1.40E-04	1.64E-03
ybaL	909	0.7	0.19	1.46E-04	1.69E-03
metQ	16613	-0.9	0.28	1.58E-04	1.83E-03
katG	16348	-0.9	0.29	1.60E-04	1.85E-03
chbF	459	-0.7	0.20	1.71E-04	1.96E-03
glcF	6516	-0.6	0.17	1.79E-04	2.06E-03
ridA	5252	-0.7	0.18	1.83E-04	2.09E-03
zinT	205	-0.8	0.26	1.93E-04	2.19E-03
thiE	2491	-0.7	0.20	2.01E-04	2.28E-03
dacA	2836	1.0	0.36	2.04E-04	2.31E-03
glk	3717	0.7	0.20	2.19E-04	2.47E-03
cheY	542	-0.9	0.31	2.31E-04	2.59E-03
dppD	1498	-0.7	0.21	2.31E-04	2.59E-03
fadM	310	-0.9	0.28	2.34E-04	2.62E-03
yfcD	1283	-0.6	0.17	2.36E-04	2.64E-03
yhdW	481	-0.9	0.28	2.45E-04	2.72E-03
gstB	3543	-0.9	0.29	2.53E-04	2.81E-03

ygjI	144	-0.7	0.20	2.54E-04	2.81E-03
grxB	1819	-0.7	0.20	2.58E-04	2.84E-03
yebQ	193	0.8	0.24	2.63E-04	2.88E-03
yacL	716	-0.7	0.22	2.63E-04	2.88E-03
xdhC	428	-0.8	0.27	2.78E-04	3.04E-03
araA	308	-0.7	0.19	2.88E-04	3.13E-03
yaeH	11801	-0.8	0.28	3.05E-04	3.30E-03
serA	5510	-0.7	0.18	3.08E-04	3.33E-03
ydhR	4963	-0.9	0.28	3.13E-04	3.37E-03
yraH	65	-0.9	0.29	3.26E-04	3.50E-03
dkgA	604	-0.9	0.33	3.41E-04	3.66E-03
ychN	3703	-0.7	0.20	3.64E-04	3.87E-03
sucD	47053	-0.8	0.24	3.73E-04	3.95E-03
ybbP	856	0.7	0.21	3.77E-04	3.98E-03
yciB	1091	0.7	0.22	3.80E-04	4.00E-03
ivy	5259	-0.9	0.32	4.01E-04	4.19E-03
ypfG	574	0.7	0.20	4.00E-04	4.19E-03
thiG	2676	-0.7	0.21	4.00E-04	4.19E-03
ygeA	628	-0.7	0.21	4.05E-04	4.23E-03
ygaH	163	0.7	0.20	4.10E-04	4.26E-03
lolD	835	0.7	0.19	4.29E-04	4.44E-03
ygaZ	579	1.0	0.38	4.33E-04	4.47E-03
yhjE	5077	-0.8	0.25	4.39E-04	4.52E-03
ydbC	1053	-0.7	0.21	4.63E-04	4.75E-03
yhfG	86	1.2	0.54	4.70E-04	4.82E-03
ybiC	1780	-0.7	0.20	4.90E-04	5.00E-03
fes	101	0.8	0.26	4.94E-04	5.03E-03
yfeW	332	0.6	0.17	5.05E-04	5.13E-03
ppc	11140	-0.7	0.20	5.13E-04	5.19E-03
malQ	865	-0.8	0.24	5.13E-04	5.19E-03
hepA	1776	0.6	0.18	5.22E-04	5.27E-03
zapC	2859	0.7	0.21	5.33E-04	5.34E-03
malF	697	-0.8	0.27	5.31E-04	5.34E-03
yafY	65	-0.9	0.30	5.33E-04	5.34E-03
ybeB	725	1.1	0.44	5.35E-04	5.35E-03
iscU	6277	-0.6	0.17	5.38E-04	5.36E-03
cpxA	1961	0.6	0.18	5.41E-04	5.38E-03
dedA	847	0.6	0.19	5.54E-04	5.50E-03
hupB	32962	-0.8	0.30	5.58E-04	5.52E-03
tar	6943	-0.8	0.27	5.74E-04	5.66E-03
ygcE	318	-0.7	0.20	5.76E-04	5.67E-03
yaiW	775	0.6	0.15	5.87E-04	5.76E-03
nlpA	913	-0.6	0.16	5.87E-04	5.76E-03
atpD	38109	-0.7	0.23	6.37E-04	6.21E-03

flgL	5539	-0.9	0.36	6.49E-04	6.31E-03
tap	4161	-0.8	0.25	6.54E-04	6.33E-03
udp	4346	-0.6	0.17	6.54E-04	6.33E-03
aldB	2155	-0.7	0.20	6.70E-04	6.45E-03
marB	79	1.1	0.49	6.71E-04	6.45E-03
ygfM	255	-0.7	0.23	6.73E-04	6.46E-03
glgS	1588	-1.2	0.52	6.83E-04	6.54E-03
elaB	1412	-0.9	0.34	6.86E-04	6.55E-03
dsdX	304	-0.9	0.32	6.93E-04	6.60E-03
dmlA	481	-0.7	0.24	7.10E-04	6.73E-03
srlB	97	-0.8	0.30	7.08E-04	6.73E-03
fhuC	305	0.7	0.20	7.12E-04	6.73E-03
iscA	7206	-0.7	0.22	7.27E-04	6.83E-03
maa	335	1.1	0.48	7.42E-04	6.94E-03
nusB	2066	1.1	0.46	7.65E-04	7.14E-03
ppdB	377	-0.7	0.21	8.04E-04	7.47E-03
ycgR	1013	-0.8	0.28	8.18E-04	7.59E-03
nmpC	13947	0.9	0.36	8.30E-04	7.68E-03
yggW	496	0.7	0.26	8.69E-04	7.99E-03
fliA	5583	-0.9	0.37	8.71E-04	7.99E-03
gdhA	7711	-0.6	0.17	8.70E-04	7.99E-03
dtpB	172	-0.8	0.27	9.04E-04	8.27E-03
dld	10069	-0.7	0.24	9.15E-04	8.35E-03
cspD	113059	-0.9	0.35	9.23E-04	8.41E-03
ybbL	245	0.7	0.23	9.26E-04	8.41E-03
yebA	5178	0.6	0.20	9.35E-04	8.47E-03
yecC	1404	-0.6	0.19	9.62E-04	8.70E-03
yjhG	524	-0.6	0.17	9.79E-04	8.84E-03
ydgA	5337	0.7	0.22	9.84E-04	8.86E-03
ysgA	1747	-0.7	0.22	9.91E-04	8.91E-03
cueO	1657	-0.7	0.22	1.02E-03	9.19E-03
frwB	155	-0.7	0.23	1.04E-03	9.33E-03
xdhA	963	-0.6	0.18	1.06E-03	9.38E-03
nagC	1429	0.6	0.17	1.08E-03	9.54E-03
pbpG	551	0.8	0.32	1.15E-03	1.02E-02
fimA	22451	-0.8	0.28	1.19E-03	1.05E-02
marA	189	1.1	0.52	1.24E-03	1.09E-02
allB	245	-0.6	0.21	1.24E-03	1.09E-02
yigI	761	-0.6	0.21	1.25E-03	1.10E-02
recF	363	0.7	0.27	1.26E-03	1.10E-02
cvpA	1472	0.8	0.29	1.28E-03	1.11E-02
ybdD	5268	-0.6	0.20	1.29E-03	1.12E-02
nagE	12796	-0.6	0.21	1.29E-03	1.12E-02
yrdA	1016	0.7	0.22	1.33E-03	1.16E-02

qor	1677	-0.7	0.26	1.34E-03	1.16E-02
ansB	298	-0.7	0.23	1.35E-03	1.16E-02
ydgI	194	0.8	0.29	1.42E-03	1.23E-02
bioA	476	1.0	0.49	1.45E-03	1.24E-02
lpxL	529	0.6	0.18	1.45E-03	1.24E-02
ybiP	276	0.7	0.22	1.46E-03	1.25E-02
fepC	152	0.6	0.20	1.46E-03	1.25E-02
yghZ	2858	-0.5	0.15	1.47E-03	1.25E-02
lysU	554	-0.6	0.19	1.48E-03	1.26E-02
yifO	199	-0.8	0.35	1.48E-03	1.26E-02
mscL	750	-0.9	0.36	1.49E-03	1.26E-02
cca	2471	-0.5	0.15	1.51E-03	1.27E-02
glpK	4155	-0.7	0.24	1.51E-03	1.28E-02
betA	286	0.6	0.19	1.54E-03	1.29E-02
ybhL	2202	0.7	0.25	1.55E-03	1.29E-02
yjhI	146	-0.6	0.20	1.55E-03	1.30E-02
lolC	823	0.9	0.36	1.58E-03	1.31E-02
mdoB	3995	0.8	0.29	1.59E-03	1.31E-02
mrdA	892	1.0	0.47	1.58E-03	1.31E-02
purK	761	1.0	0.46	1.59E-03	1.31E-02
rlmH	679	1.0	0.49	1.59E-03	1.31E-02
yghU	3299	-0.6	0.21	1.57E-03	1.31E-02
tnaC	353	-0.7	0.27	1.59E-03	1.31E-02
ycfZ	42	0.9	0.38	1.60E-03	1.31E-02
ydeE	211	0.6	0.19	1.61E-03	1.31E-02
iraD	87	-0.7	0.24	1.60E-03	1.31E-02
ybhQ	6374	-0.9	0.40	1.61E-03	1.31E-02
ggt	376	-0.7	0.26	1.60E-03	1.31E-02
yraI	25	-0.9	0.40	1.66E-03	1.35E-02
nanM	197	-0.7	0.28	1.67E-03	1.36E-02
hemH	225	0.7	0.26	1.68E-03	1.36E-02
sppA	1768	0.6	0.19	1.68E-03	1.36E-02
yfaY	1150	-0.7	0.23	1.70E-03	1.37E-02
ebgC	92	-0.7	0.29	1.72E-03	1.39E-02
fpr	1286	-0.7	0.23	1.73E-03	1.39E-02
ppdC	235	-0.6	0.20	1.73E-03	1.39E-02
yeaJ	380	0.7	0.26	1.74E-03	1.40E-02
yfaE	712	-0.6	0.21	1.77E-03	1.41E-02
ykfB	952	-0.9	0.37	1.78E-03	1.42E-02
fucI	589	-0.6	0.17	1.80E-03	1.43E-02
galR	646	0.6	0.21	2.00E-03	1.58E-02
yqiK	191	0.6	0.20	2.05E-03	1.62E-02
xseA	567	0.8	0.35	2.06E-03	1.62E-02
flhE	396	-0.7	0.28	2.06E-03	1.62E-02

Supplementary Tables

yecS	815	-0.6	0.19	2.09E-03	1.64E-02
frlA	32	-0.8	0.35	2.16E-03	1.69E-02
xdhB	270	-0.7	0.24	2.16E-03	1.69E-02
nupG	1993	0.7	0.25	2.17E-03	1.69E-02
yrhB	27	-0.8	0.36	2.18E-03	1.70E-02
yccU	926	-0.7	0.25	2.22E-03	1.72E-02
ydfK	14	1.0	0.56	2.26E-03	1.74E-02
tppB	432	0.6	0.21	2.27E-03	1.75E-02
ribE	1732	0.9	0.44	2.30E-03	1.77E-02
argD	2101	0.7	0.28	2.33E-03	1.78E-02
phoR	331	0.6	0.20	2.33E-03	1.78E-02
yncE	1789	0.6	0.20	2.33E-03	1.78E-02
plsB	4160	-0.6	0.18	2.35E-03	1.79E-02
slmA	1047	-0.6	0.19	2.38E-03	1.81E-02
thiC	8112	-0.6	0.18	2.38E-03	1.81E-02
dppC	1066	-0.6	0.19	2.48E-03	1.88E-02
ychF	1488	0.8	0.32	2.56E-03	1.93E-02
betB	435	0.6	0.18	2.57E-03	1.94E-02
cheR	984	-0.7	0.30	2.60E-03	1.96E-02
mdlA	364	0.5	0.18	2.67E-03	2.01E-02
yceI	2190	0.7	0.25	2.72E-03	2.04E-02
aceK	17152	-0.6	0.18	2.74E-03	2.06E-02
motA	2378	-0.8	0.35	2.77E-03	2.07E-02
lpxM	1182	0.7	0.29	2.81E-03	2.10E-02
smf	394	-0.6	0.24	2.85E-03	2.13E-02
mdtA	173	0.6	0.23	2.89E-03	2.15E-02
hspQ	4102	0.8	0.40	2.90E-03	2.15E-02
xylF	1823	-0.6	0.19	2.92E-03	2.16E-02
flhB	1045	-0.7	0.26	2.97E-03	2.19E-02
idnD	179	-0.7	0.27	3.00E-03	2.21E-02
melB	251	-0.6	0.22	3.02E-03	2.22E-02
ykgC	144	-0.6	0.25	3.05E-03	2.24E-02
rutR	415	0.6	0.21	3.08E-03	2.25E-02
yrbN	52	0.8	0.36	3.08E-03	2.25E-02
ompW	556	-0.6	0.21	3.07E-03	2.25E-02
nuoB	10547	0.9	0.45	3.11E-03	2.26E-02
xanP	1223	0.8	0.36	3.11E-03	2.26E-02
lpp	145818	-0.7	0.30	3.14E-03	2.27E-02
pflD	350	-0.7	0.25	3.16E-03	2.28E-02
holC	495	0.6	0.19	3.25E-03	2.35E-02
yihR	31	-0.7	0.33	3.37E-03	2.43E-02
cytR	755	0.7	0.33	3.41E-03	2.45E-02
yncD	214	0.6	0.20	3.42E-03	2.45E-02
bax	10966	0.6	0.20	3.44E-03	2.46E-02

efeB	191	-0.6	0.21	3.54E-03	2.53E-02
envZ	1800	0.5	0.17	3.58E-03	2.56E-02
frvB	74	-0.6	0.25	3.60E-03	2.56E-02
yqcA	2021	-0.6	0.21	3.61E-03	2.56E-02
tusB	191	0.7	0.28	3.60E-03	2.56E-02
nhaR	333	0.5	0.17	3.66E-03	2.59E-02
cysH	1571	-0.6	0.21	3.69E-03	2.61E-02
thiF	2329	-0.5	0.18	3.72E-03	2.63E-02
bfr	709	-0.8	0.37	3.74E-03	2.63E-02
malM	960	-0.7	0.28	3.74E-03	2.63E-02
glnS	4571	0.6	0.19	3.75E-03	2.63E-02
fadE	6074	-0.6	0.20	3.82E-03	2.68E-02
yidJ	184	-0.6	0.22	3.86E-03	2.71E-02
bglA	3942	0.6	0.21	3.91E-03	2.73E-02
tdh	1469	-0.7	0.27	3.97E-03	2.77E-02
proX	188	-0.6	0.21	4.07E-03	2.83E-02
acrR	386	0.6	0.25	4.11E-03	2.86E-02
ynjE	781	-0.5	0.19	4.11E-03	2.86E-02
ydgC	193	-0.6	0.24	4.18E-03	2.89E-02
tonB	889	0.7	0.33	4.21E-03	2.91E-02
kefA	2845	0.5	0.16	4.22E-03	2.91E-02
ynhF	242	-0.7	0.31	4.34E-03	2.99E-02
rraB	3581	-0.5	0.17	4.50E-03	3.09E-02
putA	3615	-0.6	0.20	4.51E-03	3.09E-02
ybhC	146	0.6	0.26	4.80E-03	3.27E-02
hemA	981	0.7	0.35	4.83E-03	3.29E-02
cynR	110	0.6	0.21	4.87E-03	3.30E-02
malK	548	-0.6	0.23	4.87E-03	3.30E-02
moaD	169	-0.5	0.20	4.86E-03	3.30E-02
asd	12118	-0.6	0.21	4.88E-03	3.30E-02
yjbT	22	-0.8	0.41	4.90E-03	3.31E-02
yajR	391	0.5	0.16	4.98E-03	3.35E-02
bamE	3249	-0.5	0.16	5.04E-03	3.39E-02
yieF	1375	-0.5	0.18	5.19E-03	3.49E-02
ade	430	-0.5	0.20	5.21E-03	3.49E-02
gatZ	82004	-0.5	0.15	5.22E-03	3.49E-02
yiaU	191	0.5	0.18	5.24E-03	3.50E-02
yeiG	4002	-0.6	0.25	5.33E-03	3.56E-02
yebV	421	-0.7	0.31	5.37E-03	3.57E-02
ytjA	171	-0.7	0.36	5.37E-03	3.57E-02
araF	1017	-0.5	0.16	5.38E-03	3.57E-02
rlmA	502	0.8	0.41	5.55E-03	3.67E-02
ompT	9463	0.6	0.24	5.55E-03	3.67E-02
yadE	177	0.5	0.20	5.66E-03	3.74E-02

pdhR	1152	0.6	0.27	5.82E-03	3.83E-02
ychM	650	0.5	0.19	5.84E-03	3.84E-02
yiiF	124	-0.6	0.21	5.84E-03	3.84E-02
efeO	247	-0.7	0.32	5.99E-03	3.93E-02
nfuA	6360	0.5	0.18	6.03E-03	3.95E-02
galK	1412	-0.5	0.21	6.09E-03	3.98E-02
kbl	1131	-0.6	0.21	6.15E-03	4.01E-02
fepB	131	0.7	0.31	6.22E-03	4.04E-02
yabI	1190	-0.7	0.30	6.21E-03	4.04E-02
lsrG	394	-0.6	0.26	6.37E-03	4.14E-02
ytfL	328	0.6	0.23	6.39E-03	4.14E-02
yahD	108	-0.6	0.22	6.40E-03	4.14E-02
prpE	8967	-1.0	0.89	6.49E-03	4.19E-02
ada	315	0.5	0.18	6.51E-03	4.20E-02
nuoA	8609	0.7	0.36	6.61E-03	4.25E-02
bcr	417	0.6	0.24	6.73E-03	4.32E-02
yihX	3417	-0.7	0.31	6.76E-03	4.32E-02
gltF	69	-0.6	0.26	6.76E-03	4.32E-02
purR	3052	0.8	0.42	6.91E-03	4.41E-02
ypjM_3	163	0.6	0.24	6.92E-03	4.41E-02
ybgF	3825	0.5	0.20	6.94E-03	4.41E-02
luxS	2776	-0.6	0.24	6.97E-03	4.42E-02
fepG	94	0.6	0.22	6.96E-03	4.42E-02
gudD	519	-0.5	0.20	7.07E-03	4.48E-02
yibT	650	-0.7	0.39	7.09E-03	4.48E-02
glcG	6278	-0.6	0.24	7.11E-03	4.49E-02
idnO	115	-0.7	0.32	7.17E-03	4.52E-02
pyrD	249	0.8	0.42	7.22E-03	4.54E-02
yfcE	965	-0.5	0.19	7.22E-03	4.54E-02
marR	73	0.8	0.45	7.27E-03	4.56E-02
yihS	123	-0.6	0.24	7.29E-03	4.57E-02
hslJ	560	0.6	0.25	7.45E-03	4.66E-02
yffB	297	0.6	0.24	7.46E-03	4.66E-02
yccX	163	-0.6	0.28	7.57E-03	4.72E-02
yfdI	1577	-0.5	0.19	7.59E-03	4.72E-02
yqeA	317	-0.5	0.19	7.65E-03	4.75E-02
dtpD	242	0.5	0.21	7.81E-03	4.84E-02
ycaL	65	0.7	0.31	7.82E-03	4.84E-02
atpC	19188	-0.6	0.28	7.81E-03	4.84E-02
yggX	849	-0.5	0.21	7.83E-03	4.84E-02
yaaH	136	0.5	0.20	7.85E-03	4.85E-02
yceO	27	0.7	0.34	7.88E-03	4.85E-02
yqfG	60	-0.7	0.37	7.88E-03	4.85E-02
sbmA	488	0.5	0.18	7.91E-03	4.86E-02

yajO	1882	-0.5	0.18	7.94E-03	4.87E-02
aroH	119	0.6	0.30	7.98E-03	4.89E-02
ycbB	832	0.5	0.19	8.04E-03	4.92E-02
ispG	5031	0.6	0.25	8.15E-03	4.98E-02
cheA	4234	-0.6	0.26	8.19E-03	4.99E-02

Table S6: Differentially expressed genes in H<oFTN2> during fed-batch cultivation after 2 h of induction relative to the sample drawn immediately before induction of FTN2 expression. Genes also differentially expressed in wildtype HMS174(DE3) were excluded.

Gene	baseMean	log2FoldChange	lfcSE	pvalue	padj
rstA	6850	4.6	0.17	1.36E-146	9.83E-144
rstB	7617	4.6	0.19	1.33E-132	8.01E-130
mgtA	4690	4.2	0.17	7.50E-129	3.89E-126
ybjX	3184	3.9	0.19	1.35E-90	6.12E-88
pgpC	1265	3.0	0.18	5.27E-62	2.12E-59
slyB	16067	3.5	0.21	2.71E-61	9.81E-59
yneM	3382	4.3	0.27	1.31E-58	3.96E-56
phoP	3748	2.5	0.16	2.94E-56	7.73E-54
ybjG	2473	4.3	0.27	2.98E-56	7.73E-54
gmr	1069	3.2	0.20	6.25E-56	1.51E-53
mgrB	289	4.1	0.27	4.29E-54	9.71E-52
phoQ	3282	2.8	0.18	2.17E-49	4.62E-47
yebO	2100	3.9	0.27	2.71E-48	5.46E-46
asr	325	4.1	0.29	2.03E-47	3.86E-45
tus	463	3.1	0.21	1.97E-45	3.58E-43
tqsA	302	3.3	0.23	2.35E-44	4.05E-42
maeA	6777	2.0	0.14	6.55E-43	1.08E-40
chaA	1122	2.8	0.20	1.49E-42	2.35E-40
tadA	688	2.8	0.20	3.62E-42	5.47E-40
lpxT	267	3.1	0.24	3.48E-38	4.86E-36
prpC	6102	-2.9	0.22	1.66E-37	2.23E-35
prpE	9460	-2.8	0.21	2.18E-36	2.82E-34
hemL	3173	1.8	0.14	3.72E-35	4.65E-33
prpD	9777	-2.8	0.22	7.86E-34	9.50E-32
mlaD	1633	1.9	0.16	9.37E-30	1.03E-27
dacC	2527	2.3	0.21	1.74E-28	1.86E-26
prpB	6906	-2.5	0.22	3.90E-27	4.04E-25
ydeT	106	3.2	0.30	4.98E-26	4.88E-24
ycfS	1186	2.3	0.22	9.23E-26	8.80E-24
yqfA	3639	-3.0	0.29	5.27E-25	4.90E-23
mlaF	2877	1.9	0.20	4.23E-22	3.74E-20
mlaE	1569	1.7	0.18	1.03E-21	8.88E-20
mlaC	3940	1.4	0.15	1.20E-21	1.01E-19

Supplementary Tables

queE	405	1.8	0.19	1.23E-21	1.01E-19
yjcH	7018	-1.4	0.14	1.86E-21	1.50E-19
mgtL	377	2.8	0.30	2.10E-21	1.66E-19
ydeH	369	2.3	0.24	3.81E-21	2.88E-19
ampH	1755	2.4	0.26	4.15E-20	3.07E-18
clcA	944	1.5	0.18	1.69E-18	1.20E-16
matA	54	2.7	0.33	3.00E-16	2.05E-14
mlaB	2253	1.3	0.16	5.21E-16	3.50E-14
fabR	2924	1.3	0.16	4.61E-15	2.93E-13
iraM	94	2.0	0.27	2.10E-14	1.31E-12
ydeN	1043	-1.8	0.26	1.17E-13	7.09E-12
gadW	267	1.6	0.24	2.62E-13	1.53E-11
hscA	3399	1.2	0.17	6.19E-13	3.56E-11
deoR	1472	1.3	0.18	9.11E-13	5.16E-11
matB	256	1.9	0.29	1.02E-12	5.69E-11
yebE	935	1.7	0.26	2.77E-12	1.50E-10
ompF	245017	-1.1	0.16	3.18E-12	1.70E-10
yodB	73	1.9	0.30	5.07E-12	2.66E-10
yobA	164	1.6	0.25	7.35E-12	3.81E-10
csgG	694	-1.2	0.18	2.45E-11	1.23E-09
sstT	6708	-1.2	0.19	4.01E-11	1.99E-09
nadD	519	1.7	0.28	4.22E-11	2.07E-09
amiC	1306	1.3	0.20	5.83E-11	2.77E-09
glgS	977	-1.6	0.27	5.88E-11	2.77E-09
pliG	882	-1.1	0.18	7.37E-11	3.42E-09
cpxP	8860	2.2	0.37	1.16E-10	5.25E-09
yobB	342	1.5	0.25	1.16E-10	5.25E-09
rhaB	109	-2.0	0.34	1.24E-10	5.53E-09
gldA	896	-1.4	0.23	1.32E-10	5.85E-09
yjhC	495	-1.3	0.22	1.90E-10	8.29E-09
rhaT	300	-1.4	0.23	2.79E-10	1.19E-08
dppA	39810	-0.9	0.14	2.81E-10	1.19E-08
ydeS	37	2.3	0.40	2.86E-10	1.19E-08
yccA	17352	1.3	0.21	3.48E-10	1.42E-08
livJ	25779	-1.1	0.17	3.76E-10	1.52E-08
borD	41	2.7	0.46	3.95E-10	1.56E-08
yibT	308	-1.3	0.22	4.64E-10	1.81E-08
fdx	1064	1.2	0.20	5.06E-10	1.94E-08
potD	11048	-1.1	0.18	5.08E-10	1.94E-08
fabB	7989	-1.4	0.25	7.58E-10	2.80E-08
yecD	905	1.5	0.27	8.70E-10	3.19E-08
gabP	356	-1.3	0.23	1.62E-09	5.76E-08
sseB	834	1.1	0.19	1.68E-09	5.92E-08
yhdV	106	1.8	0.34	4.01E-09	1.40E-07

ydcW	904	-1.2	0.21	4.14E-09	1.43E-07
iscX	790	1.3	0.25	5.91E-09	2.02E-07
cobC	225	1.5	0.30	7.04E-09	2.38E-07
ycfZ	35	2.1	0.41	7.35E-09	2.47E-07
holA	1088	1.3	0.25	7.48E-09	2.49E-07
ivy	3388	-0.9	0.16	1.18E-08	3.83E-07
csgA	136	-1.4	0.27	1.21E-08	3.88E-07
hflK	3806	0.9	0.16	1.45E-08	4.57E-07
dcyD	2231	-1.0	0.18	2.44E-08	7.44E-07
rhaD	49	-1.8	0.39	3.05E-08	9.21E-07
ycaC	489	-1.1	0.20	3.93E-08	1.17E-06
ybaP	150	1.3	0.25	3.98E-08	1.17E-06
rpoE	11378	-1.3	0.25	4.11E-08	1.20E-06
ompA	70809	1.1	0.21	5.33E-08	1.53E-06
csgF	196	-1.3	0.27	5.69E-08	1.62E-06
hisJ	9818	-0.8	0.15	5.82E-08	1.65E-06
osmC	1531	-1.1	0.21	6.82E-08	1.90E-06
yecE	575	1.8	0.40	7.03E-08	1.95E-06
gabT	1591	-1.1	0.22	7.28E-08	2.00E-06
ydeR	33	2.2	0.49	7.40E-08	2.02E-06
agp	6536	-1.0	0.19	7.47E-08	2.02E-06
cstA	79495	-1.1	0.22	1.00E-07	2.70E-06
yccU	628	-0.9	0.17	1.21E-07	3.18E-06
grxB	2432	-0.9	0.17	1.25E-07	3.25E-06
ycjZ	222	1.0	0.21	1.69E-07	4.38E-06
ygbE	407	1.0	0.20	1.72E-07	4.41E-06
kgtP	38391	-1.1	0.23	2.08E-07	5.28E-06
ychH	5639	-1.9	0.47	2.71E-07	6.81E-06
amiA	687	1.2	0.26	3.21E-07	7.96E-06
uspA	19899	-1.0	0.20	3.48E-07	8.59E-06
pmrD	890	0.9	0.17	4.41E-07	1.07E-05
yfeW	284	1.1	0.23	5.35E-07	1.27E-05
sra	10378	-1.1	0.25	5.39E-07	1.27E-05
ydeQ	64	1.4	0.34	5.95E-07	1.39E-05
lhgO	759	-0.9	0.18	6.04E-07	1.40E-05
dkgA	468	-1.1	0.25	6.56E-07	1.51E-05
ridA	5678	-0.8	0.15	6.66E-07	1.53E-05
yahO	530	-1.0	0.23	6.94E-07	1.58E-05
argT	27405	-0.8	0.17	8.01E-07	1.82E-05
qor	1462	-1.0	0.22	9.59E-07	2.15E-05
alx	170	1.1	0.26	1.11E-06	2.45E-05
mdfA	215	0.9	0.19	1.36E-06	2.94E-05
acs	65839	-0.9	0.20	1.35E-06	2.94E-05
yjfY	43	1.8	0.50	1.46E-06	3.14E-05

lpp	203938	-1.1	0.25	1.70E-06	3.65E-05
gabD	914	-0.9	0.19	1.72E-06	3.67E-05
hemF	425	1.2	0.30	1.75E-06	3.70E-05
apbE	314	1.1	0.25	1.84E-06	3.87E-05
mprA	912	1.0	0.22	1.85E-06	3.87E-05
tolC	7869	0.9	0.19	1.88E-06	3.91E-05
yecS	834	-0.8	0.17	1.98E-06	4.11E-05
fimA	19747	-0.8	0.16	2.21E-06	4.55E-05
cydA	10125	-0.8	0.16	2.31E-06	4.73E-05
aroL	657	0.9	0.21	2.38E-06	4.85E-05
yagU	252	-1.1	0.26	2.41E-06	4.88E-05
ytfR	633	-0.9	0.20	2.54E-06	5.11E-05
tusE	750	1.5	0.43	2.98E-06	5.97E-05
udp	4179	-0.7	0.15	3.05E-06	6.07E-05
bfr	350	-0.9	0.21	3.28E-06	6.46E-05
cydB	6298	-1.0	0.23	3.47E-06	6.79E-05
ybgT	1152	-1.0	0.25	3.63E-06	7.07E-05
tppB	423	0.8	0.19	3.68E-06	7.14E-05
elaB	1126	-0.9	0.21	4.05E-06	7.81E-05
rhaM	33	-1.9	0.56	4.81E-06	9.22E-05
ydeJ	55	1.3	0.38	5.21E-06	9.95E-05
hupA	22431	-0.8	0.18	5.85E-06	1.10E-04
yfcD	1135	-0.7	0.15	6.64E-06	1.23E-04
fabA	9270	-1.1	0.27	6.65E-06	1.23E-04
miaA	7229	0.7	0.16	7.01E-06	1.29E-04
ybiP	275	0.9	0.20	8.19E-06	1.49E-04
glnH	54012	-0.8	0.17	8.19E-06	1.49E-04
hflC	3331	0.7	0.16	8.80E-06	1.59E-04
ytfQ	2016	-0.8	0.17	9.80E-06	1.76E-04
yfeK	255	-0.8	0.20	9.86E-06	1.76E-04
lnt	739	0.8	0.19	1.01E-05	1.80E-04
dmlA	413	-0.9	0.22	1.07E-05	1.88E-04
ahpC	11980	-0.8	0.19	1.14E-05	2.00E-04
ydhR	4873	-1.0	0.27	1.18E-05	2.05E-04
dld	9269	-0.8	0.19	1.23E-05	2.12E-04
cvrA	327	0.8	0.20	1.26E-05	2.16E-04
csiR	391	-0.9	0.22	1.38E-05	2.36E-04
metC	2691	-0.8	0.20	1.43E-05	2.43E-04
eno	14883	-0.7	0.15	1.51E-05	2.56E-04
melA	354	-0.9	0.23	1.54E-05	2.60E-04
dsbA	3147	0.8	0.19	1.74E-05	2.91E-04
cspD	105479	-1.0	0.25	1.82E-05	3.03E-04
yaeH	10110	-0.9	0.22	1.94E-05	3.21E-04
ytjA	118	-1.0	0.29	2.21E-05	3.63E-04

yfdX	36	1.3	0.42	2.24E-05	3.66E-04
ygiA	85	1.0	0.26	2.37E-05	3.84E-04
yeiG	3507	-0.8	0.21	2.37E-05	3.84E-04
pyrD	218	1.1	0.31	2.44E-05	3.92E-04
dacA	2504	1.0	0.26	2.46E-05	3.94E-04
ea8.5	593	-1.0	0.26	2.48E-05	3.95E-04
mdtJ	32	1.5	0.53	2.49E-05	3.96E-04
metQ	16400	-0.8	0.19	2.57E-05	4.07E-04
ydgI	199	1.0	0.28	2.62E-05	4.12E-04
yebQ	165	0.9	0.24	2.73E-05	4.26E-04
metE	19449	-0.8	0.19	2.93E-05	4.55E-04
gstB	2488	-0.8	0.19	2.94E-05	4.55E-04
emrA	132	1.0	0.28	2.95E-05	4.55E-04
katG	13395	-0.9	0.23	3.03E-05	4.65E-04
yjhB	234	-0.7	0.18	3.41E-05	5.19E-04
maa	264	1.4	0.48	3.43E-05	5.20E-04
yebZ	211	1.0	0.30	3.49E-05	5.27E-04
gatC	81355	-0.8	0.22	3.71E-05	5.55E-04
ftnB	1465	0.8	0.21	3.74E-05	5.58E-04
ybhB	525	0.8	0.21	4.29E-05	6.35E-04
bioA	472	1.2	0.40	4.33E-05	6.39E-04
pspE	9367	-0.6	0.15	4.37E-05	6.41E-04
yfaY	1172	-0.9	0.23	4.51E-05	6.59E-04
yiaD	192	1.1	0.37	5.01E-05	7.23E-04
atpD	35714	-0.8	0.19	5.00E-05	7.23E-04
htpX	7932	0.8	0.21	5.09E-05	7.32E-04
yghU	3090	-0.8	0.20	5.51E-05	7.90E-04
spy	730	0.9	0.24	5.66E-05	8.08E-04
fumC	10964	-0.8	0.23	6.03E-05	8.57E-04
rcnB	977	0.9	0.28	6.17E-05	8.73E-04
cycA	15920	-0.7	0.17	6.61E-05	9.33E-04
argB	706	-1.0	0.33	6.88E-05	9.67E-04
hupB	20091	-0.7	0.18	7.25E-05	1.01E-03
aphA	3253	-0.7	0.19	7.94E-05	1.10E-03
yqiC	1242	-0.6	0.15	7.99E-05	1.11E-03
kbl	1010	-0.7	0.17	8.11E-05	1.12E-03
glmU	2378	-0.7	0.17	8.28E-05	1.14E-03
ybgS	101	-0.9	0.29	8.72E-05	1.19E-03
yfaE	609	-0.7	0.18	9.26E-05	1.26E-03
yihX	2287	-0.8	0.23	9.50E-05	1.29E-03
yajR	373	0.7	0.17	1.04E-04	1.40E-03
ydcH	3817	-1.0	0.32	1.05E-04	1.41E-03
yqcA	2227	-0.7	0.19	1.07E-04	1.43E-03
flu	875	-0.8	0.24	1.08E-04	1.44E-03

srlB	95	-0.9	0.28	1.09E-04	1.44E-03
gdhA	6802	-0.6	0.15	1.10E-04	1.44E-03
pagP	42	1.1	0.37	1.14E-04	1.50E-03
argH	1594	-1.1	0.41	1.18E-04	1.54E-03
degP	6473	0.8	0.22	1.26E-04	1.63E-03
mscL	494	-0.9	0.26	1.27E-04	1.64E-03
ybfP	44	1.4	0.56	1.28E-04	1.64E-03
tktB	733	-0.7	0.20	1.28E-04	1.64E-03
ykfB	860	-0.9	0.26	1.36E-04	1.74E-03
tpx	24836	-0.6	0.16	1.47E-04	1.86E-03
hisC	1626	-0.7	0.21	1.54E-04	1.95E-03
ycgR	1072	-0.7	0.18	1.55E-04	1.95E-03
fhuC	240	0.7	0.20	1.56E-04	1.96E-03
purK	734	0.9	0.30	1.66E-04	2.08E-03
glcB	37053	-0.7	0.20	1.66E-04	2.08E-03
ybeX	1825	0.6	0.17	1.70E-04	2.10E-03
yeaY	3106	-1.0	0.33	1.70E-04	2.10E-03
ybgE	2040	-0.8	0.23	1.71E-04	2.10E-03
rraB	3193	-0.6	0.17	1.74E-04	2.13E-03
ppiA	1156	1.0	0.35	1.76E-04	2.15E-03
tatC	1621	1.1	0.41	1.77E-04	2.15E-03
sucD	40116	-0.7	0.21	1.80E-04	2.19E-03
mrdA	768	1.1	0.41	1.83E-04	2.21E-03
ybdD	4210	-0.6	0.15	1.86E-04	2.23E-03
phoH	6247	-0.8	0.25	1.87E-04	2.24E-03
rpoD	16629	-0.6	0.17	2.00E-04	2.38E-03
ydeE	201	0.8	0.23	2.03E-04	2.42E-03
yfcJ	78	0.8	0.25	2.07E-04	2.45E-03
tap	4305	-0.7	0.20	2.08E-04	2.46E-03
ybeB	641	1.0	0.33	2.10E-04	2.47E-03
glpK	4261	-0.8	0.22	2.17E-04	2.53E-03
uspF	7841	-0.8	0.25	2.18E-04	2.54E-03
galR	533	0.7	0.18	2.40E-04	2.79E-03
dsdX	238	-0.8	0.26	2.42E-04	2.80E-03
tdh	1288	-0.7	0.21	2.45E-04	2.82E-03
yadE	124	0.8	0.23	2.58E-04	2.96E-03
yccJ	351	-0.8	0.23	2.72E-04	3.11E-03
soxS	540	-0.8	0.24	2.75E-04	3.13E-03
fadM	205	-0.8	0.25	2.96E-04	3.35E-03
nanC	78	-0.9	0.33	3.01E-04	3.40E-03
aldB	2328	-0.7	0.18	3.06E-04	3.44E-03
yigI	628	-0.7	0.19	3.11E-04	3.48E-03
yfgG	252	1.0	0.40	3.15E-04	3.52E-03
hflX	4076	0.6	0.17	3.20E-04	3.56E-03

hisB	2089	-0.6	0.16	3.29E-04	3.65E-03
dcp	2707	-0.6	0.16	3.45E-04	3.81E-03
yihS	90	-0.8	0.27	3.66E-04	4.02E-03
cusF	45	1.0	0.41	3.70E-04	4.06E-03
mltA	790	0.7	0.21	4.05E-04	4.41E-03
cueO	1421	-0.6	0.19	4.13E-04	4.48E-03
atpC	18427	-0.8	0.24	4.17E-04	4.51E-03
tyrP	123	0.7	0.23	4.20E-04	4.53E-03
ilvX	187	-1.1	0.47	4.24E-04	4.54E-03
ysgA	1639	-0.6	0.18	4.23E-04	4.54E-03
oppA	26501	-0.6	0.16	4.24E-04	4.54E-03
zapC	2467	0.7	0.22	4.42E-04	4.72E-03
yiiS	142	-0.7	0.21	4.50E-04	4.78E-03
cutC	3683	-1.0	0.37	4.59E-04	4.82E-03
yedV	135	1.0	0.43	4.58E-04	4.82E-03
argE	941	-0.8	0.27	4.57E-04	4.82E-03
fpr	1247	-0.7	0.21	4.60E-04	4.82E-03
hipA	264	0.7	0.22	4.66E-04	4.85E-03
mqsR	391	-0.8	0.29	4.66E-04	4.85E-03
yacL	500	-0.7	0.22	4.66E-04	4.85E-03
yijP	629	0.6	0.18	4.84E-04	5.01E-03
rpiA	2273	-0.6	0.16	4.85E-04	5.01E-03
rhtB	710	-0.8	0.25	4.89E-04	5.04E-03
ynhF	519	-1.0	0.39	4.94E-04	5.07E-03
yfhH	229	0.7	0.20	4.97E-04	5.08E-03
glmM	4888	-0.7	0.19	4.96E-04	5.08E-03
tar	7506	-0.6	0.19	5.23E-04	5.30E-03
srlA	233	-0.8	0.28	5.29E-04	5.34E-03
cheR	910	-0.7	0.23	5.41E-04	5.45E-03
yihN	168	-0.7	0.20	5.53E-04	5.55E-03
hpf	4454	-0.6	0.15	5.55E-04	5.55E-03
recA	7250	-0.5	0.14	5.81E-04	5.80E-03
cynR	85	0.8	0.27	5.85E-04	5.82E-03
pbpG	466	0.7	0.23	5.87E-04	5.83E-03
cheW	2350	-0.7	0.23	6.02E-04	5.94E-03
ygaU	573	-0.6	0.16	6.13E-04	6.04E-03
rmf	141751	-0.7	0.21	6.16E-04	6.06E-03
nagA	1444	0.6	0.15	6.22E-04	6.09E-03
lpxM	1046	0.7	0.22	6.26E-04	6.11E-03
melB	165	-0.7	0.22	6.27E-04	6.11E-03
yecC	1462	-0.6	0.17	6.36E-04	6.18E-03
nrdF	392	-0.9	0.37	6.53E-04	6.32E-03
aroM	189	0.8	0.27	6.54E-04	6.32E-03
tufA	46233	-0.6	0.19	6.56E-04	6.32E-03

hisD	1962	-0.7	0.21	6.80E-04	6.54E-03
acrD	310	0.6	0.20	6.86E-04	6.58E-03
rsxC	874	0.7	0.21	7.11E-04	6.78E-03
ftsH	12456	0.5	0.14	7.17E-04	6.83E-03
yeaJ	322	0.7	0.22	7.22E-04	6.85E-03
xanP	1282	0.8	0.28	7.49E-04	7.09E-03
asnC	93	0.8	0.26	7.71E-04	7.26E-03
purP	1084	0.7	0.20	7.83E-04	7.33E-03
srlD	396	-0.8	0.28	7.92E-04	7.38E-03
slmA	947	-0.6	0.16	7.90E-04	7.38E-03
ugpB	2198	-0.6	0.16	8.01E-04	7.45E-03
pstS	1085	0.8	0.28	8.10E-04	7.51E-03
gnsB	484	-0.6	0.16	8.18E-04	7.57E-03
murP	352	0.6	0.19	8.71E-04	8.03E-03
hemH	192	0.6	0.19	8.74E-04	8.04E-03
atpG	21560	-0.5	0.16	9.26E-04	8.49E-03
yhaH	334	-0.7	0.23	9.44E-04	8.64E-03
acnB	116515	-0.5	0.15	9.50E-04	8.68E-03
lolD	751	0.5	0.16	9.90E-04	9.02E-03
yfaW	671	-0.7	0.26	1.00E-03	9.09E-03
ybhM	53	0.9	0.40	1.02E-03	9.23E-03
cvpA	1299	0.7	0.23	1.03E-03	9.27E-03
dedA	556	0.6	0.20	1.03E-03	9.27E-03
rlmH	651	0.9	0.38	1.04E-03	9.37E-03
yicC	1983	-0.6	0.17	1.05E-03	9.37E-03
rhlE	94	0.7	0.26	1.09E-03	9.71E-03
xylA	459	-0.6	0.16	1.10E-03	9.77E-03
ycfD	2436	0.5	0.14	1.15E-03	1.02E-02
cspA	2029	0.8	0.35	1.16E-03	1.03E-02
flgL	5228	-0.7	0.26	1.17E-03	1.04E-02
hisI	1435	-0.6	0.19	1.18E-03	1.04E-02
srlE	265	-0.7	0.23	1.20E-03	1.06E-02
xseA	559	0.7	0.26	1.24E-03	1.09E-02
mdtI	35	0.8	0.35	1.26E-03	1.10E-02
ygiV	79	0.8	0.29	1.27E-03	1.10E-02
ydbC	925	-0.6	0.20	1.27E-03	1.10E-02
yaiW	649	0.6	0.21	1.29E-03	1.12E-02
sppA	1664	0.6	0.19	1.31E-03	1.14E-02
dhaK	1661	-0.6	0.21	1.34E-03	1.16E-02
sucC	42035	-0.5	0.14	1.35E-03	1.16E-02
yigZ	946	-0.6	0.21	1.35E-03	1.16E-02
sbmA	387	0.7	0.23	1.40E-03	1.20E-02
argI	593	-0.9	0.39	1.41E-03	1.20E-02
hha	340	0.9	0.42	1.42E-03	1.21E-02

degQ	1151	-0.6	0.18	1.42E-03	1.21E-02
fepA	243	0.6	0.21	1.44E-03	1.22E-02
ybjE	139	0.7	0.25	1.45E-03	1.22E-02
dppF	1991	-0.7	0.23	1.46E-03	1.23E-02
yifO	143	-0.8	0.37	1.46E-03	1.23E-02
fhuD	110	0.7	0.27	1.50E-03	1.25E-02
zapB	4645	-0.5	0.17	1.49E-03	1.25E-02
yaiA	328	0.6	0.20	1.51E-03	1.26E-02
yfcE	839	-0.5	0.16	1.53E-03	1.28E-02
garK	407	-0.6	0.22	1.53E-03	1.28E-02
hisF	1016	-0.5	0.16	1.54E-03	1.28E-02
cybB	699	0.5	0.16	1.61E-03	1.33E-02
nagE	12332	-0.6	0.17	1.61E-03	1.33E-02
dtpD	186	0.6	0.20	1.63E-03	1.34E-02
yobD	787	0.6	0.20	1.68E-03	1.38E-02
mdh	57868	-0.5	0.15	1.70E-03	1.39E-02
puuA	19112	-0.7	0.27	1.70E-03	1.39E-02
ygaZ	487	0.8	0.33	1.72E-03	1.40E-02
ygiM	3772	-0.5	0.16	1.74E-03	1.41E-02
yhjE	4616	-0.7	0.24	1.78E-03	1.44E-02
mdoB	3617	0.6	0.22	1.81E-03	1.45E-02
ppc	10163	-0.6	0.19	1.80E-03	1.45E-02
yggX	840	-0.6	0.18	1.80E-03	1.45E-02
uraA	191	0.7	0.24	1.82E-03	1.46E-02
alkB	60	0.7	0.27	1.83E-03	1.46E-02
fucO	2061	-0.6	0.21	1.85E-03	1.48E-02
lolC	720	0.7	0.27	1.88E-03	1.49E-02
rlmA	430	0.8	0.38	1.95E-03	1.55E-02
cheY	385	-0.7	0.24	2.00E-03	1.58E-02
uspD	542	-0.7	0.27	2.01E-03	1.59E-02
ykgC	100	-0.7	0.25	2.07E-03	1.63E-02
xdhD	1144	-0.6	0.22	2.09E-03	1.64E-02
dhaL	1037	-0.6	0.20	2.27E-03	1.78E-02
ybiC	1915	-0.6	0.21	2.29E-03	1.79E-02
fliA	5205	-0.7	0.27	2.32E-03	1.81E-02
bssS	2922	-0.7	0.29	2.33E-03	1.81E-02
yieF	1317	-0.5	0.17	2.41E-03	1.86E-02
hisH	865	-0.5	0.16	2.45E-03	1.89E-02
ydhJ	73	0.8	0.35	2.48E-03	1.91E-02
sodB	6768	-0.6	0.20	2.48E-03	1.91E-02
trxC	666	-0.7	0.24	2.52E-03	1.93E-02
tonB	712	0.7	0.25	2.54E-03	1.94E-02
yebV	359	-0.6	0.24	2.55E-03	1.94E-02
ybeL	1657	-0.7	0.26	2.55E-03	1.94E-02

aldA	268169	-0.5	0.16	2.56E-03	1.94E-02
fucI	538	-0.6	0.18	2.61E-03	1.97E-02
yfdE	51	0.7	0.30	2.68E-03	2.02E-02
yshB	304	-0.6	0.19	2.68E-03	2.02E-02
hspQ	1673	-0.7	0.30	2.72E-03	2.04E-02
purF	1908	0.5	0.16	2.75E-03	2.06E-02
argG	2386	-0.6	0.21	2.77E-03	2.07E-02
purU	611	0.8	0.36	2.79E-03	2.08E-02
yrbL	6326	0.6	0.20	2.80E-03	2.08E-02
pck	33906	-0.6	0.19	2.80E-03	2.08E-02
yeeE	950	0.7	0.28	2.83E-03	2.10E-02
ydiY	171	0.9	0.49	2.89E-03	2.14E-02
iraD	65	-0.7	0.28	2.91E-03	2.14E-02
ompC	64788	0.6	0.20	2.92E-03	2.15E-02
yehS	405	0.7	0.25	2.96E-03	2.17E-02
dppD	1463	-0.6	0.20	3.01E-03	2.20E-02
nanM	125	-0.6	0.21	3.07E-03	2.24E-02
fbaB	1208	-0.5	0.17	3.09E-03	2.25E-02
carA	162	0.7	0.30	3.12E-03	2.27E-02
pepE	1305	-0.5	0.17	3.25E-03	2.36E-02
wrbA	228	-0.6	0.23	3.26E-03	2.36E-02
yfeX	4408	-0.5	0.16	3.43E-03	2.47E-02
yghA	82	-0.7	0.29	3.47E-03	2.50E-02
ycaL	66	0.7	0.31	3.49E-03	2.50E-02
mdoD	4133	0.5	0.16	3.50E-03	2.51E-02
yfcL	378	0.6	0.25	3.52E-03	2.51E-02
yffB	225	0.6	0.25	3.53E-03	2.52E-02
yfeS	381	-0.6	0.20	3.55E-03	2.52E-02
ybgQ	45	0.7	0.32	3.57E-03	2.53E-02
ymfA	106	0.7	0.27	3.57E-03	2.53E-02
ydfJ	60	0.7	0.26	3.58E-03	2.53E-02
sdaA	328	0.6	0.23	3.63E-03	2.57E-02
pitA	1590	0.6	0.24	3.84E-03	2.70E-02
ygfZ	5026	-0.5	0.16	3.85E-03	2.70E-02
yhaL	1436	-0.6	0.23	3.84E-03	2.70E-02
yjcD	1119	0.7	0.29	3.86E-03	2.71E-02
prfA	442	0.6	0.21	3.90E-03	2.73E-02
iscA	5859	-0.5	0.19	3.93E-03	2.75E-02
lysU	431	-0.5	0.18	3.96E-03	2.76E-02
cspI	53	0.7	0.33	3.97E-03	2.76E-02
mdoC	129	0.7	0.32	3.99E-03	2.77E-02
mutS	3121	-0.5	0.19	4.02E-03	2.79E-02
ychM	560	0.5	0.18	4.03E-03	2.79E-02
yfdK	53	0.7	0.27	4.05E-03	2.79E-02

pka	12496	-0.5	0.18	4.05E-03	2.79E-02
astE	140	0.6	0.21	4.08E-03	2.80E-02
yebA	4518	0.5	0.16	4.11E-03	2.82E-02
ybbP	665	0.5	0.19	4.27E-03	2.93E-02
yneJ	207	0.6	0.26	4.36E-03	2.98E-02
sulA	271	-0.6	0.22	4.43E-03	3.03E-02
yciC	827	0.5	0.17	4.48E-03	3.04E-02
caiF	118	-0.6	0.21	4.53E-03	3.07E-02
yebS	243	0.6	0.23	4.73E-03	3.20E-02
ydcZ	249	0.5	0.18	4.74E-03	3.20E-02
acnA	5337	-0.5	0.16	4.76E-03	3.21E-02
glpF	1204	-0.5	0.19	4.81E-03	3.24E-02
ygeA	450	-0.5	0.17	4.89E-03	3.29E-02
purR	2716	0.7	0.33	4.95E-03	3.31E-02
ansB	238	-0.5	0.19	4.96E-03	3.31E-02
msyB	175	-0.6	0.22	4.98E-03	3.33E-02
cytR	627	0.6	0.27	5.00E-03	3.33E-02
infA	2034	0.7	0.37	5.01E-03	3.33E-02
gudD	359	-0.6	0.22	5.24E-03	3.48E-02
frvB	57	-0.7	0.31	5.28E-03	3.50E-02
ampG	767	0.6	0.22	5.30E-03	3.51E-02
mdtH	208	0.5	0.18	5.31E-03	3.51E-02
racR	755	0.6	0.22	5.33E-03	3.51E-02
tolQ	645	0.7	0.28	5.36E-03	3.53E-02
glk	2768	0.5	0.18	5.53E-03	3.62E-02
purA	30178	-0.6	0.26	5.53E-03	3.62E-02
ydiZ	103	-0.7	0.31	5.56E-03	3.62E-02
ygfF	134	-0.7	0.29	5.59E-03	3.63E-02
thiH	3696	-0.6	0.23	5.59E-03	3.63E-02
yciB	876	0.5	0.16	5.62E-03	3.64E-02
yhjV	101	0.6	0.26	5.61E-03	3.64E-02
rho	4582	0.7	0.34	5.63E-03	3.64E-02
uspG	4730	-0.6	0.23	5.66E-03	3.65E-02
yjjL	177	-0.6	0.21	5.68E-03	3.66E-02
deoA	541	-0.5	0.19	5.71E-03	3.66E-02
ptsA	169	-0.6	0.24	5.70E-03	3.66E-02
rfaD	2738	-0.5	0.18	5.71E-03	3.66E-02
ppdB	269	-0.6	0.22	5.78E-03	3.69E-02
fhuB	233	0.5	0.20	6.04E-03	3.85E-02
ybhQ	5284	-0.6	0.28	6.16E-03	3.92E-02
tatD	1022	0.6	0.21	6.29E-03	3.99E-02
hemA	893	0.6	0.25	6.28E-03	3.99E-02
fsaB	128	-0.6	0.23	6.42E-03	4.06E-02
yghZ	2679	-0.5	0.19	6.45E-03	4.07E-02

yedK	269	0.6	0.25	6.48E-03	4.09E-02
malT	50494	-0.4	0.14	6.50E-03	4.09E-02
yqjA	890	0.5	0.21	6.64E-03	4.17E-02
yhhX	1158	-0.4	0.15	6.65E-03	4.17E-02
yggW	405	0.5	0.19	6.69E-03	4.19E-02
fepC	128	0.6	0.28	6.72E-03	4.20E-02
rutR	262	0.5	0.21	6.74E-03	4.21E-02
yagX	78	0.6	0.29	6.80E-03	4.23E-02
mzrA	372	0.5	0.19	6.91E-03	4.30E-02
dapE	1023	0.5	0.16	7.00E-03	4.34E-02
glyS	12331	-0.5	0.18	7.01E-03	4.34E-02
pepP	4088	-0.5	0.16	7.06E-03	4.36E-02
xylF	1494	-0.5	0.18	7.14E-03	4.40E-02
uxaA	2074	-0.5	0.19	7.22E-03	4.45E-02
cyoD	4426	-0.5	0.20	7.25E-03	4.45E-02
gatB	15928	-0.5	0.18	7.61E-03	4.63E-02
aceB	50079	-0.5	0.22	7.61E-03	4.63E-02
mscM	373	0.5	0.17	7.62E-03	4.63E-02
yqhA	2076	0.5	0.18	7.58E-03	4.63E-02
yjdC	949	-0.5	0.20	7.60E-03	4.63E-02
rsuA	406	0.7	0.36	7.64E-03	4.63E-02
rlmC	218	0.6	0.28	7.78E-03	4.71E-02
fecR	83	0.6	0.25	7.90E-03	4.77E-02
plsB	4575	-0.5	0.16	7.93E-03	4.78E-02
ilvL	1295	-0.7	0.32	7.93E-03	4.78E-02
katE	457	-0.5	0.20	7.97E-03	4.79E-02
rfbX	90	0.7	0.40	8.06E-03	4.84E-02
luxS	2076	-0.6	0.23	8.11E-03	4.86E-02
ychN	3207	-0.5	0.18	8.14E-03	4.87E-02
glgB	8831	-0.5	0.18	8.17E-03	4.88E-02
polA	5320	-0.5	0.18	8.18E-03	4.88E-02
serA	4918	-0.5	0.20	8.24E-03	4.90E-02
bcr	408	0.5	0.18	8.31E-03	4.94E-02
uspC	286	-0.6	0.24	8.32E-03	4.94E-02
ybhC	124	0.6	0.30	8.40E-03	4.95E-02
aceA	90436	-0.5	0.22	8.37E-03	4.95E-02
endA	36	0.6	0.31	8.44E-03	4.95E-02
rsxA	582	0.6	0.27	8.44E-03	4.95E-02
msrA	837	-0.5	0.16	8.39E-03	4.95E-02
tatA	3748	-0.5	0.21	8.41E-03	4.95E-02
yaeP	269	-0.6	0.24	8.44E-03	4.95E-02

Supplementary Tables

Table S7: Differentially expressed genes in H<oFabx> during fed-batch cultivation after 12 h of induction relative to the sample drawn immediately before induction of Fabx expression. Genes also differentially expressed in wildtype HMS174(DE3) were excluded.

Gene	baseMean	log2FoldChange	lfcSE	pvalue	padj
pspA	46866	6.5	0.24	4.05E-159	5.98E-157
tauC	1612	5.8	0.22	5.76E-150	7.01E-148
pspD	3342	6.2	0.24	2.91E-146	3.44E-144
ssuD	2630	6.2	0.25	1.54E-132	1.45E-130
mtr	2639	4.7	0.19	2.84E-125	2.35E-123
pspG	1525	5.7	0.24	6.62E-123	5.16E-121
ybfA	1671	5.8	0.26	2.15E-112	1.45E-110
bhsA	975	6.5	0.29	3.96E-111	2.64E-109
ssuE	963	5.7	0.27	6.55E-99	3.52E-97
tauA	1864	5.8	0.27	1.34E-98	7.11E-97
trpE	904	3.8	0.18	2.60E-91	1.14E-89
mdtJ	763	5.8	0.29	4.95E-90	2.03E-88
tauB	1913	6.1	0.30	1.46E-89	5.88E-88
ybiJ	792	6.0	0.30	2.05E-88	7.91E-87
ssuA	1392	6.2	0.32	7.52E-84	2.68E-82
ssuB	1189	4.8	0.25	1.01E-81	3.50E-80
yohK	1183	4.3	0.22	9.88E-81	3.35E-79
ssuC	798	5.0	0.26	1.97E-77	6.14E-76
yhdV	772	5.0	0.26	2.88E-77	8.89E-76
alx	1470	4.1	0.22	1.75E-75	5.24E-74
proP	5533	3.9	0.21	2.42E-73	7.08E-72
sbp	2751	4.0	0.23	8.42E-69	2.28E-67
mdtI	539	4.2	0.24	6.97E-68	1.85E-66
sdaA	3882	4.4	0.25	1.47E-66	3.82E-65
inaA	1484	3.4	0.20	4.94E-59	1.18E-57
tsgA	830	3.3	0.20	1.11E-56	2.43E-55
mntP	1431	4.9	0.31	4.05E-55	8.67E-54
yacH	375	3.9	0.24	5.92E-55	1.25E-53
brnQ	1538	3.0	0.19	7.46E-51	1.47E-49
ydeA	617	2.7	0.18	2.39E-48	4.40E-47
lpxP	874	2.9	0.20	7.50E-45	1.26E-43
yhbW	888	2.5	0.18	5.60E-43	9.04E-42
tqsA	389	2.9	0.21	3.52E-40	5.40E-39
cbl	1347	3.2	0.24	2.74E-39	4.12E-38
ydeH	1049	2.9	0.22	1.18E-38	1.76E-37
trpD	646	2.8	0.21	3.02E-38	4.43E-37
glnA	26514	3.4	0.26	5.54E-38	8.01E-37
hslU	6222	1.9	0.15	2.79E-36	3.89E-35
nepI	494	2.5	0.20	2.02E-35	2.77E-34
marB	919	5.1	0.42	9.47E-35	1.22E-33

Supplementary Tables

psiE	572	4.5	0.36	4.32E-34	5.53E-33
yebE	2611	2.9	0.23	6.77E-34	8.58E-33
nac	338	2.7	0.22	1.55E-33	1.95E-32
cyaA	5983	2.0	0.16	2.28E-33	2.84E-32
deaD	9097	2.1	0.17	3.37E-32	4.01E-31
ibpB	1944	4.9	0.42	8.13E-32	9.60E-31
tadA	668	2.9	0.24	1.77E-31	2.08E-30
rpoH	14978	2.9	0.25	3.55E-30	4.08E-29
yedR	140	3.4	0.30	5.27E-30	5.98E-29
glpD	1647	2.4	0.21	6.11E-30	6.91E-29
amtB	779	2.1	0.18	1.96E-29	2.18E-28
asr	190	2.6	0.23	2.69E-29	2.96E-28
mdtL	4837	3.8	0.34	1.95E-28	2.10E-27
marR	719	4.7	0.44	2.18E-26	2.20E-25
marA	1731	4.8	0.45	4.06E-26	4.06E-25
rstA	1729	2.0	0.19	1.24E-25	1.22E-24
cspA	12416	3.3	0.32	2.66E-25	2.58E-24
pdhR	4369	3.0	0.28	4.53E-25	4.39E-24
iap	820	2.7	0.26	2.79E-24	2.66E-23
yjdP	333	2.5	0.24	2.83E-24	2.70E-23
degP	13668	1.9	0.18	3.24E-24	3.08E-23
ibpA	4718	3.5	0.35	1.45E-23	1.35E-22
glnK	176	3.4	0.33	1.73E-23	1.61E-22
wcaC	166	2.8	0.28	3.21E-23	2.95E-22
ybeD	3600	2.2	0.22	3.24E-23	2.97E-22
cysN	6970	2.0	0.20	6.39E-23	5.74E-22
cysC	1841	1.7	0.17	6.93E-22	6.03E-21
eno	57697	1.9	0.20	8.66E-22	7.46E-21
hslV	1812	2.1	0.22	9.11E-22	7.83E-21
wzb	69	5.3	0.56	1.53E-21	1.30E-20
yidZ	2033	3.1	0.33	5.02E-21	4.20E-20
cpxP	14599	2.7	0.29	6.54E-21	5.43E-20
chaA	919	1.8	0.18	6.78E-21	5.61E-20
ybjH	538	2.1	0.22	3.33E-20	2.70E-19
azuC	62	3.6	0.39	1.78E-19	1.40E-18
gpmM	4197	1.9	0.21	8.71E-19	6.63E-18
csdA	818	1.7	0.18	1.01E-18	7.66E-18
ycjF	1232	1.8	0.20	1.29E-18	9.74E-18
ycfS	1308	1.8	0.20	2.23E-18	1.66E-17
hha	1350	2.7	0.31	2.86E-18	2.12E-17
yciW	2581	3.1	0.36	5.42E-18	3.96E-17
yrbN	147	2.7	0.31	7.03E-18	5.13E-17
cysK	69864	1.7	0.20	2.13E-17	1.53E-16
htpX	23677	2.4	0.28	2.62E-17	1.86E-16

grpE	10635	1.7	0.19	2.71E-17	1.92E-16
yibD	172	2.1	0.25	2.85E-17	2.01E-16
cspI	262	2.2	0.26	5.55E-17	3.89E-16
csdE	358	1.7	0.19	5.67E-17	3.96E-16
lipB	978	1.9	0.22	3.31E-16	2.25E-15
pstS	2230	2.1	0.25	3.62E-16	2.46E-15
dacC	2290	1.6	0.19	1.80E-15	1.18E-14
yobB	707	2.2	0.28	2.64E-15	1.73E-14
proY	1041	2.2	0.29	2.81E-15	1.83E-14
nfsA	1130	1.6	0.20	3.06E-15	1.99E-14
yncD	397	1.6	0.20	5.98E-15	3.84E-14
ybeZ	2704	1.4	0.17	7.64E-15	4.89E-14
cysI	9359	1.4	0.17	1.74E-14	1.10E-13
yebO	1622	2.1	0.27	4.58E-14	2.85E-13
ypdK	102	2.5	0.35	1.05E-13	6.44E-13
aaeR	430	1.4	0.18	1.32E-13	8.06E-13
cysU	1734	1.8	0.23	1.43E-13	8.71E-13
acrA	6186	1.3	0.17	1.75E-13	1.06E-12
ybbN	3547	1.2	0.16	2.23E-13	1.34E-12
cysW	1866	1.2	0.16	2.77E-13	1.65E-12
cysD	6175	2.9	0.41	4.13E-13	2.45E-12
yqaE	194	2.6	0.37	4.69E-13	2.77E-12
mutM	559	1.3	0.18	9.54E-13	5.56E-12
soxS	3345	2.5	0.35	1.26E-12	7.31E-12
acrD	749	1.4	0.19	1.37E-12	7.94E-12
serC	18017	1.3	0.17	1.40E-12	8.05E-12
yfgG	867	2.6	0.38	2.41E-12	1.38E-11
typA	13421	1.8	0.26	3.05E-12	1.74E-11
hslO	1790	1.2	0.17	4.53E-12	2.57E-11
yeeO	569	1.3	0.18	4.91E-12	2.78E-11
cysH	4630	1.5	0.21	8.00E-12	4.48E-11
pinQ	75	2.1	0.31	2.28E-11	1.24E-10
dsbA	5433	1.3	0.19	3.41E-11	1.85E-10
ybjC	182	1.7	0.26	5.09E-11	2.74E-10
glnG	1359	1.5	0.23	7.55E-11	4.04E-10
cho	229	1.6	0.24	7.84E-11	4.19E-10
edd	980	1.1	0.17	1.21E-10	6.40E-10
maa	814	2.7	0.43	1.26E-10	6.62E-10
cysJ	7608	1.7	0.27	1.43E-10	7.50E-10
pgi	8008	1.6	0.24	1.47E-10	7.70E-10
gapA	47101	2.1	0.34	1.97E-10	1.03E-09
yhhS	316	1.7	0.28	2.43E-10	1.26E-09
bioA	1090	2.6	0.42	2.84E-10	1.47E-09
ygbE	819	1.3	0.19	3.49E-10	1.80E-09

ybjG	617	1.9	0.31	4.18E-10	2.14E-09
mdtD	210	1.3	0.20	4.48E-10	2.29E-09
pstC	659	1.2	0.18	5.71E-10	2.90E-09
hslR	806	1.4	0.22	6.17E-10	3.12E-09
wcaL	191	1.4	0.21	6.21E-10	3.14E-09
serS	9312	1.4	0.22	8.36E-10	4.20E-09
lpxL	824	1.2	0.20	1.46E-09	7.26E-09
yiiX	246	1.4	0.22	1.94E-09	9.60E-09
hemB	1964	1.2	0.20	2.09E-09	1.03E-08
ygaC	442	1.6	0.26	2.31E-09	1.14E-08
fimB	504	1.7	0.28	3.22E-09	1.56E-08
tilS	760	1.4	0.23	3.85E-09	1.85E-08
gnd	10258	1.2	0.19	4.28E-09	2.05E-08
yaiW	1122	1.0	0.17	5.17E-09	2.45E-08
yiaU	293	1.1	0.19	6.22E-09	2.92E-08
pfkA	4977	1.0	0.16	8.59E-09	3.98E-08
yfcJ	172	1.5	0.26	8.78E-09	4.07E-08
rimK	658	1.3	0.22	1.18E-08	5.40E-08
cysM	3199	1.5	0.26	1.24E-08	5.65E-08
yfcL	795	1.6	0.28	1.66E-08	7.47E-08
iaaA	3353	1.0	0.17	2.44E-08	1.09E-07
mliC	659	1.2	0.21	2.49E-08	1.11E-07
wcaJ	164	1.5	0.26	2.66E-08	1.18E-07
yidA	1171	1.6	0.29	4.23E-08	1.86E-07
sfsB	156	1.3	0.23	4.41E-08	1.93E-07
yhiR	649	1.7	0.31	4.72E-08	2.06E-07
ftsH	19867	0.9	0.16	5.12E-08	2.23E-07
aroA	4245	1.6	0.29	5.61E-08	2.44E-07
ybhB	745	1.2	0.22	5.88E-08	2.55E-07
glnL	730	1.3	0.23	5.93E-08	2.57E-07
rdoA	3663	1.0	0.17	5.99E-08	2.59E-07
tesB	768	1.4	0.25	6.47E-08	2.78E-07
yccA	19691	1.1	0.19	7.37E-08	3.16E-07
nudJ	145	1.4	0.26	7.96E-08	3.40E-07
yeeD	2370	1.7	0.33	8.89E-08	3.76E-07
prfA	801	1.4	0.26	1.16E-07	4.85E-07
ybjS	502	1.2	0.22	1.47E-07	6.15E-07
yecD	1024	1.3	0.24	2.07E-07	8.52E-07
yncJ	65	1.9	0.38	2.24E-07	9.17E-07
ribC	2082	1.2	0.22	2.41E-07	9.86E-07
gadA	106	1.4	0.28	2.46E-07	1.01E-06
ribB	2167	1.0	0.19	2.49E-07	1.02E-06
ycbB	1282	1.1	0.22	2.90E-07	1.18E-06
pykF	8906	1.5	0.29	3.39E-07	1.37E-06

ydjM	39	1.6	0.33	4.45E-07	1.78E-06
tusA	369	1.6	0.33	5.71E-07	2.26E-06
phoB	485	0.9	0.18	7.40E-07	2.90E-06
ydhC	164	1.2	0.23	7.60E-07	2.96E-06
bioB	1415	1.1	0.21	7.68E-07	2.99E-06
rnb	5102	1.8	0.37	8.48E-07	3.29E-06
dcrB	5922	1.2	0.24	9.17E-07	3.54E-06
yeeE	2448	1.8	0.39	9.93E-07	3.82E-06
sbmA	691	0.9	0.18	1.01E-06	3.90E-06
ygaH	225	1.1	0.21	1.04E-06	3.99E-06
pflA	1497	1.5	0.30	1.26E-06	4.80E-06
fliY	10627	1.0	0.19	1.34E-06	5.12E-06
tolB	8796	0.9	0.17	1.39E-06	5.28E-06
rluE	262	1.4	0.28	1.50E-06	5.69E-06
nlpA	2036	1.0	0.20	1.54E-06	5.81E-06
baeS	171	1.1	0.22	1.69E-06	6.38E-06
thiI	1047	1.5	0.32	1.77E-06	6.65E-06
yneM	758	1.7	0.37	2.22E-06	8.28E-06
yfgH	44	1.5	0.32	2.26E-06	8.39E-06
phoP	2074	0.9	0.18	2.48E-06	9.20E-06
yhfG	130	2.0	0.46	2.80E-06	1.03E-05
hflK	4899	0.8	0.17	2.83E-06	1.04E-05
hpt	2151	1.0	0.21	2.98E-06	1.10E-05
tomB	1131	1.6	0.34	3.11E-06	1.14E-05
glpG	700	1.2	0.27	3.16E-06	1.16E-05
wzxC	163	1.3	0.29	3.25E-06	1.19E-05
ptrB	615	0.9	0.19	3.49E-06	1.27E-05
yfaZ	242	0.9	0.19	3.93E-06	1.43E-05
smpB	1475	1.1	0.23	3.98E-06	1.45E-05
dacA	3760	1.4	0.32	5.11E-06	1.83E-05
ycfH	877	0.9	0.20	5.57E-06	1.99E-05
cvrA	498	0.9	0.20	5.81E-06	2.07E-05
yeeX	8685	1.0	0.21	6.13E-06	2.18E-05
gsiA	2632	0.7	0.15	1.02E-05	3.57E-05
dadA	10187	1.1	0.24	1.13E-05	3.95E-05
ygaZ	789	1.5	0.35	1.37E-05	4.71E-05
greB	426	0.8	0.18	1.40E-05	4.83E-05
phoR	437	0.9	0.20	1.46E-05	5.01E-05
ydgU	18	2.3	0.61	1.68E-05	5.75E-05
yfcA	457	0.9	0.20	1.77E-05	6.05E-05
amiC	1551	1.1	0.24	1.78E-05	6.07E-05
usg	1614	0.9	0.21	1.88E-05	6.41E-05
tolR	838	0.8	0.17	1.96E-05	6.64E-05
trmL	112	1.5	0.38	2.08E-05	7.06E-05

Supplementary Tables

ea59_1	1209	1.5	0.37	2.56E-05	8.58E-05
yfcM	755	1.0	0.24	2.70E-05	9.06E-05
fldA	2884	1.2	0.30	2.92E-05	9.73E-05
tusE	1015	1.7	0.46	2.95E-05	9.82E-05
yaeB	498	1.7	0.44	3.19E-05	1.06E-04
ycbC	346	0.9	0.22	3.46E-05	1.14E-04
ydfU	53	1.3	0.33	5.32E-05	1.73E-04
ispH	1791	1.1	0.28	5.61E-05	1.82E-04
sppA	2216	0.8	0.19	6.58E-05	2.12E-04
panD	2883	0.8	0.19	6.62E-05	2.13E-04
lolB	589	1.3	0.35	7.15E-05	2.29E-04
grxA	379	1.0	0.25	7.39E-05	2.36E-04
tolQ	1084	1.3	0.35	7.45E-05	2.38E-04
purD	1380	0.7	0.17	8.08E-05	2.57E-04
hemA	1415	1.3	0.35	8.09E-05	2.57E-04
purH	1365	1.4	0.37	8.09E-05	2.57E-04
ratB	408	1.0	0.25	8.33E-05	2.63E-04
cdh	216	1.4	0.38	8.55E-05	2.70E-04
glpE	407	1.0	0.26	8.57E-05	2.70E-04
yciH	364	1.0	0.26	8.75E-05	2.76E-04
ycaL	89	1.2	0.30	8.79E-05	2.77E-04
yadG	1260	0.8	0.19	9.07E-05	2.86E-04
yagI	572	0.8	0.21	9.94E-05	3.12E-04
rsuA	808	1.7	0.48	1.01E-04	3.15E-04
yaeQ	414	0.9	0.23	1.04E-04	3.26E-04
rlmF	553	1.0	0.26	1.16E-04	3.59E-04
ycfD	3395	0.7	0.16	1.24E-04	3.82E-04
ydhJ	120	1.2	0.34	1.36E-04	4.18E-04
gmk	1175	0.9	0.24	1.44E-04	4.41E-04
ydcO	192	0.8	0.21	1.53E-04	4.66E-04
ydgI	242	1.0	0.27	1.55E-04	4.72E-04
proW	244	0.9	0.25	1.73E-04	5.24E-04
yqjI	393	0.8	0.20	1.86E-04	5.61E-04
pqiA	453	0.8	0.20	1.97E-04	5.94E-04
leuO	264	0.7	0.20	2.34E-04	6.99E-04
apbE	396	1.0	0.28	2.40E-04	7.17E-04
yejH	303	0.9	0.23	2.41E-04	7.17E-04
yecE	564	1.3	0.38	2.52E-04	7.49E-04
rimI	263	1.2	0.33	2.53E-04	7.49E-04
cysA	6048	0.9	0.25	2.86E-04	8.45E-04
guaA	7081	0.6	0.14	3.05E-04	8.98E-04
ibsC	11	1.9	0.65	3.12E-04	9.18E-04
yigL	601	1.1	0.33	3.29E-04	9.62E-04
rcnB	1100	1.0	0.27	3.66E-04	1.06E-03

purK	984	1.4	0.43	3.66E-04	1.06E-03
menF	583	1.0	0.28	3.84E-04	1.11E-03
kdsC	1062	0.7	0.18	4.01E-04	1.16E-03
yeaD	669	0.7	0.21	4.96E-04	1.42E-03
yigM	457	1.3	0.42	5.39E-04	1.53E-03
iscX	881	0.8	0.25	6.00E-04	1.70E-03
yjjG	430	1.1	0.34	6.05E-04	1.71E-03
yciC	1110	0.6	0.18	6.55E-04	1.84E-03
dadX	5754	0.8	0.24	6.75E-04	1.90E-03
folB	107	0.8	0.24	7.26E-04	2.03E-03
argS	3833	0.7	0.19	7.61E-04	2.13E-03
yjaG	862	0.9	0.28	7.73E-04	2.16E-03
yeiR	216	1.1	0.35	8.24E-04	2.29E-03
rdgB	494	1.1	0.34	9.67E-04	2.66E-03
hemH	270	0.8	0.26	9.85E-04	2.71E-03
rimN	765	1.1	0.36	1.03E-03	2.83E-03
metJ	1443	1.0	0.34	1.08E-03	2.95E-03
mmuP	450	0.7	0.20	1.17E-03	3.17E-03
aat	581	0.7	0.19	1.17E-03	3.18E-03
rlmH	804	1.3	0.45	1.18E-03	3.21E-03
dtpB	393	0.8	0.26	1.27E-03	3.43E-03
mgtL	23	1.2	0.41	1.39E-03	3.73E-03
rlmC	314	1.0	0.33	1.40E-03	3.75E-03
metF	2779	1.3	0.48	1.41E-03	3.78E-03
hflC	3883	0.6	0.16	1.43E-03	3.82E-03
mdoC	187	1.0	0.36	1.51E-03	4.03E-03
ybfE	182	0.9	0.30	1.54E-03	4.10E-03
cdgR	388	0.8	0.25	1.66E-03	4.39E-03
yneG	135	0.9	0.31	1.70E-03	4.50E-03
metK	5279	2.2	0.98	1.90E-03	4.97E-03
pstA	521	0.7	0.21	1.93E-03	5.05E-03
rcsF	669	0.9	0.32	2.10E-03	5.47E-03
fkpB	750	0.8	0.26	2.13E-03	5.55E-03
nusB	2269	1.1	0.41	2.14E-03	5.55E-03
insH-6	45	0.9	0.30	2.14E-03	5.56E-03
hemF	472	0.9	0.33	2.20E-03	5.70E-03
prmC	229	0.6	0.20	2.35E-03	6.06E-03
rsmG	336	0.8	0.28	2.41E-03	6.20E-03
recF	417	0.8	0.27	2.42E-03	6.21E-03
cpxA	2203	0.6	0.17	2.67E-03	6.82E-03
trxB	4126	0.7	0.21	2.67E-03	6.83E-03
cmoA	409	1.0	0.39	3.03E-03	7.67E-03
ybjL	927	0.6	0.18	3.04E-03	7.69E-03
yggW	550	0.7	0.24	3.07E-03	7.76E-03

pabA	185	0.8	0.29	3.15E-03	7.93E-03
proB	2546	0.5	0.16	3.23E-03	8.11E-03
cysP	4166	2.0	1.03	3.51E-03	8.76E-03
amiA	724	0.8	0.30	3.62E-03	8.99E-03
mlaA	2155	0.7	0.26	3.70E-03	9.18E-03
yqeI	67	0.9	0.32	3.84E-03	9.49E-03
yceG	736	0.6	0.20	3.85E-03	9.52E-03
erpA	5655	0.7	0.25	3.92E-03	9.66E-03
rimP	1647	0.9	0.32	4.00E-03	9.86E-03
fau	678	0.7	0.23	4.08E-03	1.00E-02
clpP	5135	0.5	0.18	4.11E-03	1.01E-02
lolD	928	0.6	0.18	4.12E-03	1.01E-02
yehS	610	0.7	0.23	4.21E-03	1.03E-02
miaA	9291	0.7	0.24	4.27E-03	1.04E-02
yeaP	705	0.9	0.35	4.42E-03	1.07E-02
gpt	1664	1.7	0.86	4.73E-03	1.14E-02
fdx	1189	0.6	0.19	4.99E-03	1.20E-02
purU	921	1.1	0.46	5.05E-03	1.22E-02
holE	147	0.9	0.36	5.61E-03	1.34E-02
ybeB	744	1.0	0.40	6.09E-03	1.44E-02
ybhA	319	1.1	0.51	6.54E-03	1.54E-02
aaeA	113	0.7	0.29	6.68E-03	1.58E-02
yibL	637	0.7	0.26	6.72E-03	1.58E-02
cmk	1614	0.7	0.29	6.74E-03	1.59E-02
pbpG	598	0.8	0.31	6.89E-03	1.62E-02
exoX	783	0.5	0.19	6.91E-03	1.62E-02
rnt	363	1.0	0.42	7.01E-03	1.64E-02
gltF	139	0.6	0.23	7.25E-03	1.70E-02
trxA	8393	0.7	0.25	7.47E-03	1.74E-02
ybdL	928	0.6	0.24	7.69E-03	1.79E-02
ispE	1067	0.5	0.16	7.86E-03	1.82E-02
asnS	10430	0.6	0.23	7.89E-03	1.83E-02
lolC	889	0.8	0.34	8.05E-03	1.86E-02
ribE	1869	0.9	0.41	8.33E-03	1.92E-02
rsmC	689	0.8	0.31	8.35E-03	1.93E-02
yciB	1171	0.6	0.21	8.38E-03	1.93E-02
pth	470	1.0	0.45	8.83E-03	2.03E-02
yodB	87	0.7	0.26	9.48E-03	2.17E-02
nusA	7718	0.5	0.17	9.66E-03	2.20E-02
pheS	1827	0.8	0.34	1.02E-02	2.30E-02
djlA	538	0.6	0.21	1.03E-02	2.32E-02
yneH	603	0.7	0.31	1.05E-02	2.38E-02
rnc	994	0.8	0.35	1.06E-02	2.39E-02
ppiA	1457	0.8	0.33	1.15E-02	2.58E-02

mrdA	911	0.9	0.43	1.17E-02	2.62E-02
yfiH	523	0.5	0.17	1.20E-02	2.68E-02
yccF	310	0.7	0.27	1.25E-02	2.77E-02
guaC	3218	0.5	0.20	1.36E-02	3.01E-02
grxD	3355	0.5	0.18	1.44E-02	3.17E-02
gsk	1080	0.7	0.31	1.51E-02	3.31E-02
yjeK	701	0.6	0.24	1.53E-02	3.35E-02
ybbA	610	0.6	0.23	1.56E-02	3.41E-02
gntK	67	0.7	0.33	1.57E-02	3.42E-02
purE	457	1.5	0.96	1.58E-02	3.44E-02
lptF	1272	0.5	0.19	1.58E-02	3.45E-02
yfiC	319	0.8	0.35	1.59E-02	3.47E-02
yjjX	317	0.6	0.25	1.62E-02	3.52E-02
hscA	3222	0.5	0.19	1.65E-02	3.58E-02
cydD	1066	0.5	0.21	1.72E-02	3.73E-02
yeeN	1003	0.7	0.31	1.76E-02	3.80E-02
mutL	1261	0.5	0.19	1.77E-02	3.81E-02
yecT	52	0.7	0.34	1.88E-02	4.02E-02
holD	184	0.7	0.34	1.89E-02	4.04E-02
hisG	2050	0.5	0.18	1.94E-02	4.13E-02
ygjP	103	0.6	0.29	2.20E-02	4.65E-02
phnE_1	14	0.9	0.49	2.29E-02	4.81E-02
actP	31875	-8.3	0.21	0.00E+00	0.00E+00
dctA	32058	-7.8	0.18	0.00E+00	0.00E+00
gatB	13520	-8.8	0.23	3.19E-301	3.30E-298
gatA	24286	-7.6	0.21	7.02E-277	5.80E-274
gatD	16421	-7.3	0.21	2.38E-260	1.64E-257
yjcH	6177	-8.9	0.26	2.95E-251	1.74E-248
gatZ	58490	-6.6	0.20	1.60E-244	8.25E-242
acs	44078	-7.8	0.24	4.63E-231	1.91E-228
glcA	5229	-5.6	0.17	5.88E-219	2.21E-216
malT	38373	-5.4	0.18	1.99E-205	5.48E-203
aldA	263678	-8.7	0.29	2.27E-196	5.22E-194
gatC	70498	-7.9	0.27	6.65E-188	1.20E-185
yghZ	2106	-5.0	0.18	8.14E-170	1.35E-167
cstA	69605	-7.3	0.28	2.92E-152	3.77E-150
fadI	2630	-4.2	0.16	1.57E-140	1.76E-138
agp	6368	-5.5	0.22	7.90E-138	8.59E-136
yqeF	4841	-5.1	0.20	3.48E-136	3.69E-134
gatY	34771	-5.8	0.23	7.97E-136	8.24E-134
manX	23147	-5.9	0.24	8.96E-135	9.04E-133
paaK	2754	-4.2	0.17	3.21E-134	3.16E-132
malE	4993	-6.0	0.24	1.91E-132	1.72E-130
mhpR	2495	-4.4	0.18	4.52E-126	3.81E-124

mglC	1483	-4.9	0.20	2.14E-124	1.74E-122
yedF	4845	-4.7	0.19	5.46E-122	4.18E-120
uxaC	1788	-4.8	0.20	1.33E-120	9.79E-119
mtlA	5695	-4.4	0.19	5.68E-120	4.12E-118
manY	16921	-5.5	0.24	9.34E-114	6.54E-112
ydcH	4084	-6.1	0.28	5.00E-108	3.23E-106
feaR	2656	-4.3	0.19	1.14E-107	7.28E-106
pck	21626	-4.7	0.22	2.92E-98	1.53E-96
manZ	25209	-5.6	0.26	4.09E-98	2.11E-96
pka	9797	-4.9	0.23	3.04E-96	1.49E-94
xylF	1401	-4.5	0.22	1.80E-95	8.63E-94
aceA	87604	-4.2	0.20	4.48E-95	2.13E-93
aceK	13293	-4.0	0.19	1.95E-94	9.14E-93
lamB	1674	-4.7	0.23	9.19E-93	4.08E-91
mtfA	11855	-4.7	0.23	2.70E-91	1.18E-89
ompF	272190	-6.0	0.29	3.20E-91	1.38E-89
uxuA	1169	-3.9	0.19	8.49E-91	3.62E-89
ybdD	4067	-5.0	0.24	1.20E-90	5.06E-89
nagE	10092	-4.4	0.22	1.28E-89	5.18E-88
glnH	50748	-4.1	0.20	3.79E-89	1.50E-87
ytfQ	1950	-4.4	0.22	1.05E-88	4.08E-87
fadJ	7515	-4.8	0.24	5.17E-88	1.98E-86
fadL	4839	-4.1	0.20	2.54E-86	9.53E-85
putP	4633	-3.6	0.18	3.74E-85	1.34E-83
aldB	1712	-4.2	0.22	4.40E-81	1.52E-79
cycA	14334	-3.2	0.16	5.24E-80	1.75E-78
aspA	6170	-3.8	0.20	7.68E-80	2.54E-78
araF	787	-3.4	0.18	2.03E-79	6.65E-78
rihC	888	-3.8	0.20	2.07E-79	6.73E-78
paaJ	2536	-3.8	0.20	2.81E-73	8.19E-72
ytfJ	1272	-4.3	0.24	2.32E-72	6.66E-71
uxaA	1584	-4.1	0.22	1.39E-71	3.97E-70
frdB	1488	-3.9	0.21	1.96E-70	5.52E-69
glcF	5341	-3.3	0.18	5.10E-70	1.42E-68
aceB	49232	-3.4	0.19	1.07E-69	2.98E-68
mglA	2272	-5.6	0.32	2.38E-69	6.54E-68
glcE	5415	-3.1	0.17	3.42E-69	9.37E-68
lhgO	589	-3.9	0.22	1.27E-68	3.39E-67
sdhA	18081	-3.4	0.19	6.41E-67	1.68E-65
malM	781	-4.7	0.27	2.86E-63	7.22E-62
frdA	3013	-3.2	0.19	2.62E-62	6.49E-61
cspD	95967	-5.7	0.34	4.89E-62	1.20E-60
frdC	940	-4.3	0.26	5.55E-62	1.36E-60
rnk	4092	-2.6	0.16	8.94E-62	2.17E-60

sdhB	16578	-4.0	0.24	4.96E-59	1.18E-57
malK	446	-3.7	0.22	5.61E-59	1.32E-57
mppA	2040	-2.9	0.17	9.89E-59	2.32E-57
ygiS	3064	-2.8	0.17	7.18E-58	1.64E-56
yfaW	623	-4.0	0.25	4.18E-57	9.39E-56
csiE	607	-3.7	0.23	6.84E-56	1.49E-54
ysgA	1430	-3.8	0.24	5.24E-55	1.11E-53
yidQ	4079	-3.5	0.22	1.99E-54	4.18E-53
uspF	8678	-5.4	0.34	2.43E-54	5.07E-53
fadE	5054	-2.9	0.18	6.63E-54	1.37E-52
malF	595	-3.9	0.25	9.47E-53	1.91E-51
glnQ	8673	-3.1	0.20	3.44E-51	6.79E-50
maeB	15939	-2.6	0.17	7.63E-51	1.49E-49
yedE	5119	-3.9	0.26	2.14E-49	4.06E-48
ddpA	712	-3.2	0.21	2.22E-49	4.18E-48
galS	424	-3.3	0.22	3.13E-49	5.87E-48
fucO	2211	-3.8	0.26	4.81E-49	8.95E-48
glcD	7973	-2.4	0.16	3.62E-48	6.65E-47
yihY	1236	-3.0	0.20	9.87E-48	1.80E-46
fumA	17014	-2.9	0.20	1.09E-47	1.97E-46
ydcI	6930	-2.3	0.15	2.08E-47	3.73E-46
glnP	3775	-3.2	0.22	4.89E-47	8.64E-46
fucR	1694	-2.8	0.19	6.31E-47	1.11E-45
bsmA	1303	-3.8	0.26	1.87E-46	3.28E-45
csgG	886	-2.6	0.18	2.16E-46	3.77E-45
rmuC	1283	-3.1	0.22	6.32E-45	1.07E-43
chbC	1260	-3.4	0.24	3.27E-44	5.43E-43
frdD	1450	-3.6	0.26	1.66E-42	2.64E-41
ugpB	1859	-2.9	0.21	6.19E-42	9.73E-41
ytfR	655	-3.4	0.25	2.54E-41	3.96E-40
gatR_2	1379	-3.3	0.24	1.24E-40	1.93E-39
gabD	805	-2.8	0.21	1.36E-40	2.11E-39
yaiZ	482	-3.1	0.23	1.99E-40	3.07E-39
fucA	286	-2.9	0.21	3.73E-40	5.71E-39
sdhC	6098	-3.2	0.24	4.44E-40	6.77E-39
yjjM	219	-3.1	0.23	1.17E-39	1.78E-38
nrdF	397	-3.1	0.23	8.76E-39	1.31E-37
gabT	1393	-3.1	0.23	1.45E-38	2.15E-37
xdhB	220	-3.9	0.29	2.21E-38	3.25E-37
nuoF	8344	-2.5	0.19	3.14E-37	4.49E-36
ybhQ	5594	-4.8	0.38	1.23E-36	1.75E-35
srlA	251	-3.7	0.29	1.62E-36	2.30E-35
fecA	603	-2.5	0.19	1.73E-36	2.43E-35
lsrA	280	-2.6	0.20	1.78E-36	2.49E-35

araC	1261	-2.8	0.22	2.17E-36	3.04E-35
melR	259	-2.9	0.22	3.61E-36	4.97E-35
atoC	483	-2.6	0.20	2.23E-35	3.02E-34
kbaZ	224	-3.0	0.24	3.42E-35	4.58E-34
fucI	510	-2.4	0.19	5.41E-35	7.09E-34
paaY	1412	-2.5	0.20	5.55E-35	7.26E-34
putA	3104	-2.6	0.21	6.43E-35	8.38E-34
ivbL	462	-4.6	0.37	7.42E-35	9.64E-34
agaV	154	-3.6	0.29	7.84E-35	1.02E-33
malG	358	-3.4	0.28	6.66E-34	8.46E-33
ydbC	925	-2.7	0.22	7.00E-34	8.82E-33
nuoE	4078	-2.6	0.21	4.56E-33	5.58E-32
sgcX	247	-2.7	0.22	1.35E-32	1.63E-31
sdhD	2732	-3.2	0.26	2.12E-32	2.54E-31
ygjI	123	-3.1	0.26	2.45E-32	2.94E-31
ddpX	217	-3.0	0.25	4.93E-32	5.84E-31
hisJ	8968	-2.1	0.18	3.13E-31	3.64E-30
dmlR	766	-2.4	0.20	3.90E-30	4.47E-29
nuoG	16009	-2.5	0.22	1.03E-29	1.16E-28
lsrR	707	-2.9	0.25	1.12E-29	1.26E-28
ppsA	17295	-2.7	0.23	5.24E-29	5.76E-28
chiP	421	-3.3	0.29	6.06E-29	6.64E-28
araA	266	-2.8	0.25	8.13E-29	8.89E-28
chbB	1050	-2.8	0.25	1.40E-28	1.52E-27
leuB	17500	-3.8	0.34	1.87E-28	2.02E-27
srlE	301	-3.2	0.29	5.14E-28	5.42E-27
srlD	394	-2.8	0.25	5.76E-28	6.06E-27
yhfY	168	-2.5	0.23	1.54E-27	1.61E-26
gabP	415	-3.2	0.29	1.63E-27	1.70E-26
sstT	6409	-2.3	0.21	3.68E-27	3.81E-26
atoS	309	-2.3	0.21	9.29E-27	9.55E-26
garD	576	-2.1	0.20	2.04E-26	2.07E-25
yagE	97	-3.3	0.31	5.06E-26	5.05E-25
melA	374	-2.2	0.20	7.86E-26	7.81E-25
araB	156	-2.5	0.24	1.09E-25	1.08E-24
yihX	2918	-3.6	0.34	2.29E-25	2.23E-24
lsrK	1552	-3.0	0.28	3.03E-25	2.94E-24
nanE	220	-2.5	0.24	5.86E-25	5.66E-24
nuoJ	3478	-2.3	0.23	1.34E-24	1.28E-23
ldrD	1363	-3.1	0.31	4.52E-24	4.27E-23
leuA	28928	-4.6	0.46	9.70E-24	9.14E-23
nuoH	5599	-2.9	0.28	1.06E-23	9.90E-23
nuoI	2821	-2.3	0.23	1.09E-23	1.02E-22
yhfZ	352	-2.8	0.27	1.59E-23	1.47E-22

Supplementary Tables

speD	1915	-2.1	0.21	1.97E-23	1.83E-22
ydcS	494	-3.1	0.31	2.05E-23	1.89E-22
ucpA	3940	-2.8	0.28	2.97E-23	2.74E-22
pscG	445	-2.6	0.26	4.06E-23	3.71E-22
ebgC	78	-3.8	0.38	4.34E-23	3.94E-22
agaA	240	-2.4	0.24	4.56E-23	4.12E-22
rayT	344	-2.1	0.21	1.28E-22	1.15E-21
ade	375	-2.4	0.24	1.73E-22	1.54E-21
nanT	326	-2.5	0.25	2.25E-22	1.99E-21
rihA	1014	-3.4	0.34	3.33E-22	2.93E-21
pssA	7791	-1.7	0.18	5.01E-22	4.38E-21
astC	504	-1.9	0.19	6.17E-22	5.38E-21
preT	184	-2.3	0.24	7.76E-22	6.74E-21
cpdA	4691	-2.1	0.21	7.96E-22	6.90E-21
agaW	137	-3.1	0.32	8.34E-22	7.21E-21
xylG	373	-2.5	0.26	2.23E-21	1.89E-20
eutT	94	-2.4	0.26	3.23E-21	2.72E-20
gltK	806	-1.9	0.19	3.58E-21	3.02E-20
yehT	438	-1.9	0.19	3.68E-21	3.09E-20
ygcE	310	-1.8	0.18	6.15E-21	5.12E-20
nuoL	9290	-2.5	0.27	9.31E-21	7.68E-20
pscK	164	-3.2	0.34	1.53E-20	1.26E-19
nuoK	1154	-2.1	0.23	1.58E-20	1.30E-19
ydcW	1315	-2.7	0.29	1.78E-20	1.46E-19
dppD	1440	-2.0	0.21	2.78E-20	2.26E-19
nuoN	7918	-2.2	0.24	3.57E-20	2.89E-19
btuB	4022	-1.6	0.17	4.27E-20	3.43E-19
yahN	138	-2.5	0.27	4.29E-20	3.44E-19
ebgA	289	-1.9	0.20	4.42E-20	3.54E-19
ycaM	444	-1.6	0.17	1.73E-19	1.36E-18
yjhB	357	-1.7	0.19	1.97E-19	1.54E-18
ybaE	238	-2.4	0.27	2.22E-19	1.73E-18
paaX	1007	-1.8	0.20	3.27E-19	2.53E-18
uidA	361	-1.8	0.19	4.41E-19	3.37E-18
nuoM	5211	-1.8	0.19	4.63E-19	3.54E-18
nrdE	722	-3.1	0.35	5.77E-19	4.40E-18
xylH	282	-2.3	0.25	1.15E-18	8.69E-18
rhaS	207	-2.2	0.25	1.16E-18	8.75E-18
yjhX	66	-3.3	0.38	1.40E-18	1.05E-17
yjhQ	139	-2.6	0.30	1.80E-18	1.35E-17
acnB	109533	-1.5	0.17	3.47E-18	2.56E-17
gltL	1782	-1.9	0.22	4.06E-18	2.99E-17
lrp	8721	-2.0	0.23	4.35E-18	3.19E-17
copA	5546	-1.8	0.21	1.01E-17	7.36E-17

malP	678	-1.6	0.19	1.06E-17	7.70E-17
fucP	138	-2.3	0.27	1.64E-17	1.18E-16
yddH	156	-1.9	0.22	1.81E-17	1.30E-16
dppF	1843	-2.2	0.26	2.29E-17	1.64E-16
eutM	185	-2.4	0.29	2.46E-17	1.75E-16
sgcC	241	-1.9	0.22	2.90E-17	2.05E-16
hupB	31197	-2.6	0.30	3.29E-17	2.32E-16
cdaR	630	-1.9	0.22	4.41E-17	3.10E-16
hisP	1059	-1.8	0.21	6.21E-17	4.33E-16
hisM	521	-1.7	0.20	6.50E-17	4.52E-16
modB	466	-1.8	0.21	6.96E-17	4.84E-16
araG	297	-1.8	0.21	7.80E-17	5.41E-16
bglG	175	-2.5	0.30	9.93E-17	6.88E-16
yajO	1762	-1.7	0.20	1.09E-16	7.52E-16
yidF	179	-2.4	0.28	1.24E-16	8.51E-16
prpC	5045	-6.7	0.85	2.56E-16	1.75E-15
ddpD	293	-1.8	0.21	2.66E-16	1.82E-15
surE	1351	-1.6	0.19	3.11E-16	2.12E-15
yegT	151	-1.9	0.23	6.86E-16	4.62E-15
prpD	8361	-6.8	0.89	8.36E-16	5.61E-15
ygfU	279	-1.6	0.19	8.94E-16	5.99E-15
dkgB	636	-1.6	0.19	9.66E-16	6.45E-15
ychH	7585	-3.6	0.45	1.02E-15	6.82E-15
yhdW	466	-2.3	0.29	1.13E-15	7.52E-15
rhaT	513	-2.0	0.25	1.18E-15	7.80E-15
preA	343	-1.8	0.22	1.77E-15	1.17E-14
fecB	310	-1.6	0.20	3.17E-15	2.05E-14
yagN	1431	-2.0	0.25	3.37E-15	2.18E-14
hisQ	1528	-1.6	0.19	4.30E-15	2.77E-14
eutQ	108	-2.2	0.28	4.73E-15	3.05E-14
prpB	4915	-6.1	0.83	1.05E-14	6.72E-14
allB	237	-1.8	0.23	1.25E-14	7.98E-14
modC	890	-1.7	0.22	1.38E-14	8.74E-14
iclR	1017	-1.7	0.22	1.44E-14	9.12E-14
yjhG	512	-1.6	0.20	1.79E-14	1.13E-13
ydiP	518	-1.7	0.22	2.99E-14	1.88E-13
rlmD	1318	-1.8	0.24	3.31E-14	2.07E-13
ybeL	2069	-2.4	0.32	4.56E-14	2.84E-13
modA	1525	-2.4	0.32	4.95E-14	3.08E-13
gldA	1075	-1.7	0.22	8.06E-14	4.97E-13
ycjD	141	-1.7	0.22	8.77E-14	5.39E-13
ilvC	13772	-2.0	0.27	1.27E-13	7.77E-13
yjhH	150	-1.7	0.23	1.65E-13	1.00E-12
yijD	2767	-1.3	0.17	1.92E-13	1.16E-12

yihL	182	-1.6	0.21	2.26E-13	1.36E-12
sgcB	54	-2.9	0.41	3.50E-13	2.09E-12
aes	259	-1.4	0.19	4.39E-13	2.60E-12
ddpB	143	-2.0	0.27	6.39E-13	3.75E-12
ydcV	105	-1.9	0.27	8.85E-13	5.17E-12
relA	5378	-1.4	0.19	1.29E-12	7.48E-12
lsrC	211	-1.7	0.23	1.52E-12	8.77E-12
yeiM	299	-1.5	0.20	2.84E-12	1.63E-11
pykA	6247	-1.3	0.18	4.38E-12	2.49E-11
yihN	255	-1.5	0.21	4.55E-12	2.58E-11
prpE	9246	-5.7	0.86	4.86E-12	2.75E-11
ygcO	38	-2.7	0.40	4.92E-12	2.78E-11
ydcT	150	-1.9	0.28	5.66E-12	3.19E-11
amyA	969	-1.6	0.23	6.12E-12	3.45E-11
fadM	320	-1.8	0.26	6.90E-12	3.87E-11
bax	6342	-1.8	0.26	7.66E-12	4.29E-11
yidE	1142	-1.4	0.19	8.49E-12	4.73E-11
ugpE	91	-1.8	0.27	9.98E-12	5.56E-11
chbA	196	-1.5	0.21	1.05E-11	5.83E-11
ytfT	210	-1.7	0.25	1.24E-11	6.87E-11
pflD	340	-1.9	0.28	1.30E-11	7.20E-11
pmrD	499	-1.8	0.26	1.77E-11	9.70E-11
fhuF	1732	-1.1	0.15	1.96E-11	1.08E-10
dgoR	333	-1.5	0.22	2.22E-11	1.22E-10
flu	1208	-1.6	0.24	2.26E-11	1.23E-10
ykfA	407	-1.5	0.22	2.77E-11	1.51E-10
ygfT	302	-1.3	0.18	3.39E-11	1.84E-10
gntP	229	-1.6	0.24	4.16E-11	2.24E-10
alsB	228	-1.7	0.25	5.32E-11	2.86E-10
yjhI	144	-1.6	0.24	5.98E-11	3.20E-10
yjfF	301	-1.6	0.24	6.95E-11	3.72E-10
nrdI	248	-2.8	0.45	9.31E-11	4.95E-10
dcuR	837	-1.2	0.18	1.19E-10	6.30E-10
yahO	707	-2.3	0.37	1.27E-10	6.67E-10
tdcC	253	-1.5	0.22	1.90E-10	9.95E-10
eutD	106	-1.7	0.27	2.04E-10	1.06E-09
ugpC	513	-1.5	0.23	2.45E-10	1.27E-09
fabG	13609	-1.3	0.19	3.47E-10	1.79E-09
yiiF	124	-1.5	0.23	4.71E-10	2.41E-09
fdoI	675	-1.6	0.25	5.53E-10	2.82E-09
fecC	104	-1.5	0.24	6.14E-10	3.11E-09
malQ	907	-1.5	0.24	6.22E-10	3.14E-09
minE	3167	-1.6	0.25	7.22E-10	3.64E-09
cueO	1717	-1.4	0.23	8.56E-10	4.30E-09

ycjG	2025	-1.7	0.28	9.94E-10	4.97E-09
yidJ	187	-1.5	0.24	2.14E-09	1.06E-08
idnD	183	-1.6	0.27	2.20E-09	1.09E-08
yqeA	320	-1.3	0.21	2.54E-09	1.25E-08
frwB	154	-1.7	0.29	2.64E-09	1.30E-08
tam	297	-1.5	0.24	2.67E-09	1.31E-08
glmM	5708	-1.3	0.22	3.16E-09	1.54E-08
ddpC	246	-1.4	0.24	3.21E-09	1.56E-08
yiaO	60	-1.6	0.27	3.32E-09	1.61E-08
dgoK	169	-1.3	0.22	3.77E-09	1.82E-08
srlR	402	-1.2	0.19	4.35E-09	2.08E-08
rfaH	576	-1.3	0.22	4.62E-09	2.20E-08
xseB	786	-1.2	0.20	5.05E-09	2.39E-08
napG	110	-1.8	0.32	5.72E-09	2.70E-08
dinD	288	-1.3	0.22	5.81E-09	2.74E-08
ybiA	101	-1.5	0.26	6.10E-09	2.87E-08
ygjH	86	-2.2	0.40	7.50E-09	3.49E-08
yagH	161	-1.7	0.29	7.84E-09	3.65E-08
sgcE	89	-1.4	0.24	7.99E-09	3.71E-08
mukF	1976	-1.0	0.16	9.25E-09	4.27E-08
idnO	113	-1.9	0.34	9.96E-09	4.60E-08
yghU	3417	-1.4	0.23	1.01E-08	4.67E-08
fdoH	971	-1.4	0.24	1.14E-08	5.24E-08
xylR	553	-1.3	0.21	1.23E-08	5.61E-08
rpoE	14334	-2.0	0.36	1.26E-08	5.73E-08
fecE	176	-1.2	0.21	1.32E-08	6.01E-08
cyoD	5964	-1.4	0.25	1.35E-08	6.12E-08
yabQ	609	-1.6	0.27	1.40E-08	6.33E-08
bolA	2618	-1.9	0.34	2.03E-08	9.11E-08
ykgF	437	-1.2	0.21	2.40E-08	1.07E-07
yeiE	711	-1.6	0.28	3.16E-08	1.40E-07
ddpF	208	-1.2	0.21	3.27E-08	1.45E-07
minD	6079	-1.1	0.19	3.71E-08	1.63E-07
mdh	46455	-1.1	0.19	3.76E-08	1.66E-07
nanM	217	-1.3	0.23	4.80E-08	2.09E-07
yhbT	2514	-1.4	0.26	6.03E-08	2.61E-07
yagG	107	-1.3	0.24	7.60E-08	3.25E-07
ycaC	709	-1.7	0.33	7.91E-08	3.38E-07
potC	608	-1.0	0.17	8.10E-08	3.44E-07
torY	106	-1.4	0.26	9.18E-08	3.87E-07
yeaV	143	-1.2	0.21	1.18E-07	4.97E-07
xanQ	151	-1.2	0.22	1.43E-07	5.98E-07
yhbS	2006	-1.3	0.24	1.50E-07	6.26E-07
gntT	400	-1.1	0.21	1.57E-07	6.53E-07

rhaR	121	-1.3	0.24	1.63E-07	6.78E-07
yegU	251	-1.1	0.21	1.73E-07	7.19E-07
ygcP	114	-2.0	0.41	1.74E-07	7.22E-07
yedK	198	-1.5	0.28	1.84E-07	7.60E-07
mak	366	-1.1	0.20	1.88E-07	7.78E-07
ftsW	1649	-1.3	0.23	1.93E-07	7.98E-07
ydhF	1098	-0.9	0.16	2.32E-07	9.48E-07
yeiG	4141	-1.4	0.27	2.54E-07	1.03E-06
curA	533	-1.3	0.25	2.93E-07	1.19E-06
ykgD	133	-1.3	0.25	3.23E-07	1.31E-06
yadI	440	-1.0	0.19	3.28E-07	1.33E-06
eamA	1019	-1.2	0.23	3.62E-07	1.46E-06
nanC	139	-1.1	0.22	3.82E-07	1.54E-06
agaS	43	-1.5	0.30	3.86E-07	1.55E-06
dsdC	207	-1.1	0.21	3.94E-07	1.58E-06
rpoS	12170	-1.4	0.28	4.29E-07	1.71E-06
sgcQ	195	-1.0	0.20	5.09E-07	2.02E-06
ilvL	365	-1.4	0.28	5.87E-07	2.32E-06
yjeI	4766	-1.1	0.21	7.00E-07	2.75E-06
ybjD	1075	-1.2	0.24	7.51E-07	2.94E-06
ydbJ	287	-1.4	0.29	7.55E-07	2.95E-06
yhaM	369	-1.0	0.19	7.65E-07	2.98E-06
mmuM	1576	-1.2	0.25	9.62E-07	3.72E-06
yiiD	1899	-1.0	0.20	9.68E-07	3.74E-06
glgA	5915	-1.0	0.19	9.85E-07	3.80E-06
yfcH	2941	-1.0	0.21	1.08E-06	4.15E-06
mukB	10839	-1.3	0.28	1.12E-06	4.29E-06
ldrB	49	-1.7	0.37	1.27E-06	4.85E-06
ybgE	2700	-1.3	0.27	1.30E-06	4.94E-06
dld	10773	-1.3	0.27	1.36E-06	5.19E-06
torZ	543	-1.2	0.24	1.41E-06	5.35E-06
murG	1847	-1.0	0.21	1.58E-06	5.99E-06
pepN	13002	-1.3	0.26	1.66E-06	6.28E-06
ybfN	62	-1.7	0.36	1.77E-06	6.65E-06
ykgE	169	-1.1	0.22	1.85E-06	6.95E-06
malS	289	-1.0	0.20	2.09E-06	7.81E-06
gudP	291	-1.1	0.23	2.12E-06	7.92E-06
guaD	323	-1.0	0.20	2.16E-06	8.06E-06
csiR	527	-1.4	0.30	2.17E-06	8.09E-06
sfsA	2060	-1.2	0.26	3.86E-06	1.40E-05
pcm	1333	-0.9	0.18	4.48E-06	1.62E-05
araD	43	-1.5	0.33	4.58E-06	1.65E-05
ugpA	95	-1.4	0.32	4.76E-06	1.71E-05
mraY	1566	-1.0	0.22	4.95E-06	1.78E-05

csgA	194	-1.4	0.30	5.65E-06	2.02E-05
murR	265	-1.0	0.22	6.67E-06	2.37E-05
ddlB	3094	-1.5	0.34	7.75E-06	2.74E-05
dxs	3738	-0.8	0.16	8.13E-06	2.87E-05
yfbM	80	-1.2	0.27	8.71E-06	3.06E-05
yihS	138	-1.0	0.21	8.74E-06	3.07E-05
uidB	168	-1.1	0.24	9.81E-06	3.45E-05
fecD	81	-1.2	0.27	1.10E-05	3.86E-05
yejF	699	-1.1	0.24	1.17E-05	4.07E-05
yacL	801	-1.1	0.25	1.18E-05	4.11E-05
rtn	677	-0.9	0.20	1.28E-05	4.42E-05
nlpD	33375	-1.2	0.28	1.29E-05	4.46E-05
glgP	10761	-0.9	0.21	1.49E-05	5.10E-05
cydB	8368	-1.2	0.27	1.66E-05	5.69E-05
frwC	161	-1.0	0.23	1.68E-05	5.75E-05
zraS	210	-1.0	0.23	1.82E-05	6.20E-05
yidK	116	-1.0	0.23	2.09E-05	7.09E-05
yihT	71	-1.1	0.25	2.10E-05	7.09E-05
ydeM	119	-1.1	0.25	2.14E-05	7.24E-05
ygdH	4689	-0.9	0.19	2.31E-05	7.78E-05
ynjH	168	-1.3	0.30	2.32E-05	7.81E-05
yfaX	86	-1.2	0.29	2.59E-05	8.69E-05
truD	2102	-1.0	0.23	2.83E-05	9.44E-05
fabA	9081	-1.1	0.25	3.09E-05	1.03E-04
yqiA	811	-0.9	0.22	3.28E-05	1.09E-04
gutM	43	-1.3	0.32	3.79E-05	1.24E-04
cydA	12608	-0.9	0.22	4.31E-05	1.41E-04
paaC	51	-1.2	0.29	5.54E-05	1.79E-04
sgbH	58	-1.1	0.27	6.20E-05	2.00E-04
sgbU	61	-1.1	0.27	6.47E-05	2.08E-04
mdoB	2284	-1.1	0.28	1.06E-04	3.31E-04
hofN	72	-1.0	0.27	1.28E-04	3.95E-04
pflC	150	-0.9	0.25	1.45E-04	4.43E-04
astE	133	-0.9	0.24	1.72E-04	5.21E-04
araH	234	-0.9	0.25	1.91E-04	5.77E-04
yggR	145	-1.0	0.26	2.03E-04	6.10E-04
clpA	41368	-1.2	0.34	2.14E-04	6.41E-04
yjjL	268	-0.8	0.21	2.29E-04	6.84E-04
yqcE	99	-1.2	0.34	2.39E-04	7.12E-04
rseA	27006	-1.1	0.32	2.79E-04	8.24E-04
ybgT	1781	-1.2	0.35	2.86E-04	8.45E-04
yigZ	1115	-0.8	0.22	3.28E-04	9.58E-04
tdcB	148	-0.9	0.24	3.34E-04	9.77E-04
cspC	29789	-0.7	0.19	3.47E-04	1.01E-03

gstB	4195	-1.0	0.30	3.59E-04	1.05E-03
yhjD	201	-0.9	0.25	3.66E-04	1.06E-03
ushA	1890	-0.7	0.21	4.26E-04	1.23E-03
sgcA	64	-0.9	0.26	4.46E-04	1.28E-03
artM	624	-0.7	0.20	5.56E-04	1.58E-03
ykgG	356	-0.8	0.23	6.16E-04	1.74E-03
fabD	5981	-0.6	0.16	6.78E-04	1.90E-03
tdcA	139	-0.8	0.24	9.69E-04	2.67E-03
sxy	392	-0.8	0.25	9.91E-04	2.72E-03
uspA	29939	-1.0	0.31	1.01E-03	2.78E-03
selD	6765	-0.6	0.17	1.08E-03	2.94E-03
mazE	312	-0.8	0.26	1.19E-03	3.23E-03
yajD	802	-0.7	0.23	1.22E-03	3.29E-03
sgcR	141	-0.7	0.23	1.38E-03	3.71E-03
ppsR	776	-0.8	0.25	1.47E-03	3.94E-03
melB	300	-0.7	0.21	1.57E-03	4.16E-03
phoU	1582	-0.7	0.22	1.57E-03	4.17E-03
yejA	730	-0.7	0.20	1.57E-03	4.18E-03
ydhR	6093	-0.9	0.28	1.59E-03	4.21E-03
ssb	4267	-0.6	0.18	1.82E-03	4.78E-03
bfr	833	-1.1	0.37	1.85E-03	4.85E-03
paaE	69	-0.8	0.28	2.15E-03	5.57E-03
ugpQ	538	-0.7	0.24	2.50E-03	6.42E-03
csgB	38	-1.0	0.34	2.72E-03	6.94E-03
dkgA	751	-0.9	0.32	2.81E-03	7.14E-03
yidL	244	-0.7	0.25	2.89E-03	7.34E-03
yagJ	397	-0.8	0.26	3.10E-03	7.81E-03
yceD	9915	-0.6	0.19	3.59E-03	8.95E-03
ykgR	17	-1.1	0.44	3.76E-03	9.32E-03
ybgK	2295	-0.7	0.27	4.58E-03	1.11E-02
yqfA	3623	-1.9	0.97	4.71E-03	1.14E-02
paaB	45	-0.8	0.31	4.85E-03	1.17E-02
sodA	14784	-0.6	0.22	5.25E-03	1.26E-02
paaF	59	-0.7	0.28	5.59E-03	1.33E-02
feaB	949	-0.6	0.23	5.92E-03	1.41E-02
yphF	88	-0.7	0.29	6.39E-03	1.51E-02
ggt	463	-0.7	0.26	7.14E-03	1.67E-02
fumC	16669	-0.7	0.26	7.53E-03	1.75E-02
yheU	153	-0.8	0.31	7.53E-03	1.75E-02
sra	12826	-0.7	0.27	8.13E-03	1.88E-02
ycgB	588	-0.9	0.39	9.45E-03	2.16E-02
alsA	104	-0.6	0.24	1.01E-02	2.29E-02
yahK	539	-0.5	0.18	1.04E-02	2.36E-02
caiF	179	-0.6	0.23	1.17E-02	2.63E-02

ybgJ	1853	-0.7	0.28	1.18E-02	2.64E-02
plaP	2979	-0.7	0.29	1.27E-02	2.83E-02
yabI	1446	-0.7	0.33	1.39E-02	3.06E-02
yifO	250	-0.8	0.35	1.40E-02	3.08E-02
tktB	911	-0.6	0.27	1.47E-02	3.23E-02
yadS	241	-0.5	0.18	1.55E-02	3.39E-02
yjiH	91	-0.7	0.29	1.61E-02	3.50E-02
fixC	84	-0.6	0.25	1.78E-02	3.82E-02
yegE	1106	-0.5	0.19	2.00E-02	4.25E-02
rpiB	113	-0.7	0.30	2.01E-02	4.27E-02
ybiB	2219	-0.6	0.27	2.02E-02	4.29E-02
cptB	896	-0.5	0.20	2.06E-02	4.36E-02
pspE	12574	-0.5	0.19	2.07E-02	4.38E-02
dgt	1458	-0.6	0.24	2.23E-02	4.70E-02
hemD	1047	-0.5	0.19	2.27E-02	4.79E-02
pgpC	1389	2.8	0.20	1.32E-45	2.25E-44
mdtG	369	3.3	0.23	1.22E-43	1.97E-42
yobA	536	3.0	0.21	1.28E-42	2.06E-41
pmrR	275	3.6	0.27	8.66E-40	1.32E-38
yhjX	657	3.0	0.22	1.85E-39	2.79E-38
ybjX	1861	2.8	0.21	3.24E-38	4.73E-37
zntA	1116	2.9	0.22	8.78E-38	1.26E-36
kdpA	448	2.9	0.23	3.30E-36	4.56E-35
rutA	176	3.2	0.27	7.43E-33	9.03E-32
treF	1043	1.8	0.16	9.79E-27	1.00E-25
emrD	183	2.4	0.22	1.06E-26	1.08E-25
lysU	2466	2.3	0.21	1.32E-25	1.30E-24
atoB	658	3.0	0.29	1.97E-24	1.89E-23
nfrB	343	2.2	0.22	4.08E-23	3.71E-22
yafD	2876	1.8	0.18	1.15E-21	9.84E-21
frvX	359	1.9	0.20	9.04E-21	7.47E-20
slp	543	2.8	0.30	1.71E-20	1.40E-19
ldhA	2910	2.4	0.26	6.05E-20	4.83E-19
pfkB	1489	1.7	0.18	7.65E-20	6.08E-19
mgrB	133	2.4	0.26	1.48E-19	1.17E-18
yfeO	474	1.9	0.20	2.09E-19	1.63E-18
kdpC	126	2.5	0.29	1.19E-18	9.01E-18
ddlA	1828	1.7	0.19	1.77E-18	1.33E-17
yebZ	642	2.6	0.30	3.71E-18	2.73E-17
nfrA	501	1.7	0.19	1.01E-17	7.36E-17
soxR	229	1.8	0.21	1.26E-17	9.09E-17
hdeA	1619	1.9	0.22	2.16E-17	1.55E-16
yecJ	544	1.7	0.20	1.42E-16	9.77E-16
gadB	157	2.2	0.28	1.43E-15	9.45E-15

rstB	1624	1.6	0.20	1.83E-15	1.20E-14
yafE	552	1.6	0.20	2.88E-15	1.87E-14
cbpA	777	1.4	0.18	3.15E-14	1.97E-13
gltP	474	1.7	0.22	1.21E-13	7.38E-13
ybcH	132	1.8	0.24	1.46E-13	8.86E-13
slyB	8156	1.5	0.19	1.69E-13	1.02E-12
iraD	297	1.7	0.23	1.97E-13	1.19E-12
lipA	6379	1.7	0.23	2.27E-13	1.36E-12
yibA	551	1.8	0.25	3.77E-13	2.24E-12
mlaF	3595	1.5	0.20	4.70E-13	2.77E-12
ychO	455	1.4	0.19	6.08E-13	3.57E-12
zraP	55	2.1	0.32	1.21E-11	6.70E-11
rutB	75	1.9	0.30	1.79E-10	9.37E-10
ybbB	297	1.3	0.20	2.20E-10	1.14E-09
mlaD	1725	1.0	0.15	2.82E-10	1.46E-09
ybeY	1554	1.2	0.18	3.89E-10	2.00E-09
pinR	50	2.1	0.35	5.72E-10	2.90E-09
fhlA	444	1.2	0.19	8.63E-10	4.33E-09
yciG	61	2.1	0.36	8.71E-10	4.36E-09
rutF	58	2.8	0.48	1.29E-09	6.43E-09
mlaC	4306	1.1	0.17	1.72E-09	8.52E-09
leuE	615	1.2	0.18	2.83E-09	1.38E-08
deoB	8770	1.1	0.17	4.33E-09	2.08E-08
mlaE	1777	1.2	0.19	4.50E-09	2.15E-08
ygjD	1125	1.1	0.18	4.80E-09	2.29E-08
norV	190	1.3	0.22	6.31E-09	2.95E-08
ada	473	1.1	0.18	7.22E-09	3.37E-08
yedJ	747	1.0	0.17	7.32E-09	3.41E-08
nemR	135	1.3	0.23	1.01E-08	4.63E-08
setB	145	1.4	0.25	1.21E-08	5.54E-08
asnC	128	1.4	0.24	1.26E-08	5.75E-08
ybhF	1136	1.1	0.18	1.57E-08	7.09E-08
galP	329	1.4	0.25	3.67E-08	1.62E-07
clpB	18706	1.8	0.33	4.35E-08	1.91E-07
maeA	4699	0.9	0.16	5.82E-08	2.52E-07
nei	329	1.2	0.22	6.24E-08	2.69E-07
gadW	373	1.2	0.22	8.76E-08	3.71E-07
yhbO	114	1.6	0.31	8.90E-08	3.76E-07
yebG	447	1.1	0.20	9.12E-08	3.85E-07
rutC	25	2.5	0.52	2.04E-07	8.42E-07
queE	767	1.4	0.26	2.13E-07	8.78E-07
rutD	59	1.7	0.33	2.15E-07	8.85E-07
hdeB	587	1.2	0.23	3.04E-07	1.23E-06
aceE	47863	1.0	0.19	3.45E-07	1.39E-06

rutE	47	1.8	0.38	3.95E-07	1.58E-06
trmJ	2796	1.0	0.19	5.17E-07	2.05E-06
ybhG	1017	1.2	0.24	7.47E-07	2.92E-06
pdxH	1779	1.1	0.23	1.27E-06	4.84E-06
yoeB	821	1.3	0.26	1.91E-06	7.18E-06
ygaP	252	1.4	0.30	2.02E-06	7.55E-06
bdm	264	1.5	0.32	2.25E-06	8.36E-06
norW	109	1.1	0.24	2.36E-06	8.76E-06
baeR	729	1.0	0.21	2.55E-06	9.42E-06
panE	409	1.0	0.21	2.64E-06	9.74E-06
yajL	633	0.9	0.19	2.80E-06	1.03E-05
eda	2536	1.0	0.21	2.93E-06	1.08E-05
mlaB	2569	0.9	0.20	3.85E-06	1.40E-05
ybaO	228	1.2	0.27	4.14E-06	1.50E-05
hdeD	229	1.2	0.26	4.16E-06	1.51E-05
osmC	5093	1.4	0.32	4.79E-06	1.72E-05
ffh	5640	0.8	0.16	5.42E-06	1.94E-05
nanR	1384	1.0	0.21	5.48E-06	1.96E-05
bioD	595	1.0	0.22	7.11E-06	2.52E-05
bioC	430	0.8	0.18	7.82E-06	2.77E-05
uvrA	2243	0.9	0.20	7.94E-06	2.81E-05
crcB	245	1.2	0.26	9.02E-06	3.17E-05
yeaK	726	0.9	0.20	9.93E-06	3.48E-05
yggI	103	1.7	0.43	1.16E-05	4.03E-05
yebY	818	0.9	0.19	1.16E-05	4.03E-05
proV	312	1.5	0.36	1.18E-05	4.11E-05
ydiE	12	2.5	0.66	1.35E-05	4.64E-05
otsB	211	1.1	0.25	1.37E-05	4.71E-05
alr	1153	0.8	0.18	1.86E-05	6.32E-05
gmr	461	0.9	0.19	1.95E-05	6.61E-05
yeeZ	3132	1.0	0.23	2.89E-05	9.64E-05
ybiH	515	1.1	0.26	2.98E-05	9.91E-05
ypfH	777	0.9	0.22	3.13E-05	1.04E-04
insH-5	177	1.0	0.23	3.39E-05	1.12E-04
ackA	4359	1.2	0.29	3.55E-05	1.17E-04
ybgS	349	1.4	0.35	4.40E-05	1.44E-04
yzcX	54	1.2	0.29	4.45E-05	1.45E-04
yrdB	451	0.8	0.20	4.70E-05	1.53E-04
gltX	7985	1.0	0.25	5.44E-05	1.76E-04
oppA	68078	1.1	0.27	5.62E-05	1.82E-04
idi	253	1.0	0.24	5.97E-05	1.93E-04
rutG	303	1.0	0.26	6.12E-05	1.97E-04
ybgA	42	1.3	0.34	6.84E-05	2.20E-04
ybbL	301	0.9	0.22	7.10E-05	2.27E-04

alkB	138	0.9	0.23	7.39E-05	2.36E-04
yaiI	526	0.8	0.21	8.13E-05	2.58E-04
rhtC	1033	0.8	0.20	9.74E-05	3.06E-04
ymcE	28	1.5	0.43	9.86E-05	3.09E-04
yhiD	134	1.2	0.31	9.89E-05	3.10E-04
bioF	924	0.7	0.16	1.08E-04	3.35E-04
yjiX	36	1.3	0.35	1.13E-04	3.50E-04
adiY	84	1.1	0.28	1.18E-04	3.64E-04
yadE	231	0.8	0.21	1.24E-04	3.83E-04
ygaV	34	1.3	0.35	1.27E-04	3.92E-04
aroE	842	0.9	0.25	1.30E-04	4.01E-04
mltF	327	0.9	0.23	1.42E-04	4.35E-04
phoQ	1515	0.7	0.18	1.48E-04	4.52E-04
yahA	279	0.8	0.19	1.53E-04	4.65E-04
gadC	520	1.1	0.29	1.69E-04	5.14E-04
mgtA	1080	0.9	0.23	1.78E-04	5.37E-04
alkA	278	0.9	0.23	1.85E-04	5.59E-04
ygjV	193	0.8	0.21	2.74E-04	8.10E-04
yhaK	203	0.9	0.24	3.05E-04	8.99E-04
dxr	1397	0.7	0.19	3.16E-04	9.28E-04
frvR	119	0.9	0.24	3.26E-04	9.54E-04
sieB	42	1.2	0.36	3.51E-04	1.02E-03
ycdZ	730	0.8	0.22	3.62E-04	1.05E-03
yhhK	174	0.8	0.23	3.71E-04	1.08E-03
yfhR	42	1.0	0.30	3.83E-04	1.11E-03
yebS	345	0.8	0.21	4.05E-04	1.17E-03
yjeM	183	0.9	0.25	4.26E-04	1.23E-03
mfd	4837	0.8	0.22	4.34E-04	1.25E-03
rem	137	0.8	0.24	4.55E-04	1.31E-03
yedL	219	0.9	0.26	4.61E-04	1.32E-03
ppk	2583	0.8	0.21	4.72E-04	1.35E-03
ynfN	22	1.6	0.55	5.18E-04	1.48E-03
endA	70	1.0	0.31	5.23E-04	1.49E-03
metA	2162	0.8	0.24	5.28E-04	1.51E-03
yfdS	33	1.1	0.33	5.31E-04	1.51E-03
kdsB	3112	0.6	0.15	5.47E-04	1.55E-03
nirB	162	0.8	0.24	6.03E-04	1.71E-03
ybaS	80	1.0	0.29	6.15E-04	1.74E-03
mdlA	464	0.7	0.21	6.29E-04	1.77E-03
sodB	15647	0.9	0.25	6.37E-04	1.79E-03
hsrA	328	0.8	0.22	6.78E-04	1.90E-03
cspG	220	1.1	0.35	7.79E-04	2.17E-03
gadX	462	1.4	0.46	7.98E-04	2.22E-03
gsiD	455	0.7	0.18	8.40E-04	2.33E-03

ybbM	191	0.8	0.26	8.41E-04	2.34E-03
mcbA	105	1.2	0.38	8.70E-04	2.41E-03
chaC	1246	0.8	0.23	8.75E-04	2.42E-03
lpxT	127	1.0	0.33	8.93E-04	2.47E-03
rssB	993	0.6	0.17	8.98E-04	2.48E-03
gltS	541	0.6	0.18	9.16E-04	2.53E-03
yicG	57	0.9	0.27	1.01E-03	2.78E-03
dnaK	38305	1.1	0.36	1.05E-03	2.87E-03
zntR	1329	1.0	0.30	1.05E-03	2.88E-03
yaeJ	250	1.0	0.34	1.08E-03	2.95E-03
orf-314_1	24	1.1	0.37	1.08E-03	2.95E-03
yjeA	533	1.0	0.34	1.14E-03	3.10E-03
yafQ	1131	0.8	0.24	1.15E-03	3.13E-03
ydcM	1925	0.7	0.21	1.15E-03	3.13E-03
hdhA	1528	0.7	0.19	1.20E-03	3.24E-03
yliI	133	0.7	0.21	1.29E-03	3.48E-03
pta	7587	0.6	0.19	1.42E-03	3.79E-03
yeaJ	447	0.8	0.26	1.46E-03	3.92E-03
nirD	28	1.1	0.38	1.50E-03	4.00E-03
nfsB	3299	0.6	0.17	1.50E-03	4.01E-03
fldB	1455	0.6	0.19	1.65E-03	4.35E-03
pepD	8524	0.9	0.32	1.67E-03	4.41E-03
nudL	235	0.7	0.23	1.71E-03	4.51E-03
yhdN	546	0.8	0.26	1.88E-03	4.93E-03
ysaA	153	0.7	0.21	2.12E-03	5.52E-03
ybiT	5357	0.7	0.22	2.17E-03	5.61E-03
rdgC	1654	1.0	0.36	2.20E-03	5.68E-03
ratA	633	0.7	0.24	2.22E-03	5.73E-03
yfcR	97	0.7	0.25	2.32E-03	5.97E-03
ymdC	768	0.5	0.16	2.37E-03	6.08E-03
yefM	321	0.9	0.32	2.48E-03	6.37E-03
trpL	291	1.0	0.38	2.63E-03	6.74E-03
pgk	17611	1.0	0.37	2.68E-03	6.84E-03
yigB	746	1.2	0.44	2.69E-03	6.86E-03
gmhB	1041	0.6	0.20	2.96E-03	7.51E-03
torT	161	0.7	0.23	2.98E-03	7.56E-03
torS	282	0.7	0.22	3.00E-03	7.61E-03
yihD	847	0.6	0.20	3.01E-03	7.62E-03
ftsB	643	0.6	0.20	3.05E-03	7.70E-03
hemL	2125	0.5	0.16	3.07E-03	7.75E-03
can	5808	0.8	0.27	3.13E-03	7.89E-03
nfo	1375	0.6	0.21	3.21E-03	8.05E-03
ligT	82	0.9	0.32	3.61E-03	8.99E-03
aaeX	16	1.1	0.46	3.79E-03	9.39E-03

ybjN	1209	0.8	0.28	3.86E-03	9.53E-03
ygeO	52	0.8	0.29	3.86E-03	9.53E-03
yacC	592	0.6	0.21	4.03E-03	9.91E-03
ydhT	125	0.7	0.24	4.05E-03	9.95E-03
npr	679	0.6	0.18	4.11E-03	1.01E-02
yeaR	43	0.9	0.33	4.12E-03	1.01E-02
yhhN	660	0.6	0.20	4.13E-03	1.01E-02
yeaM	190	0.6	0.22	4.23E-03	1.03E-02
dps	4158	0.7	0.23	4.24E-03	1.03E-02
ygiD	420	0.6	0.22	4.41E-03	1.07E-02
orn	661	0.8	0.28	4.43E-03	1.08E-02
tldD	2022	0.5	0.17	4.74E-03	1.14E-02
rffA	745	0.5	0.17	4.97E-03	1.20E-02
yqhC	204	0.6	0.23	5.09E-03	1.22E-02
dmsA	235	0.6	0.19	5.12E-03	1.23E-02
ydcY	1128	0.8	0.29	5.18E-03	1.24E-02
ynaE	19	1.1	0.46	5.20E-03	1.25E-02
yafO	143	0.8	0.31	5.26E-03	1.26E-02
pdxY	1388	0.8	0.32	5.27E-03	1.26E-02
ydfP	76	0.7	0.26	5.30E-03	1.27E-02
cadC	156	0.6	0.21	5.53E-03	1.32E-02
mrp	3847	0.6	0.19	5.63E-03	1.34E-02
yqgF	799	0.6	0.23	5.92E-03	1.41E-02
kdpE	169	0.6	0.20	6.09E-03	1.44E-02
cybB	868	0.6	0.21	6.11E-03	1.45E-02
pabB	231	0.7	0.28	6.16E-03	1.46E-02
yegL	110	0.6	0.23	6.60E-03	1.56E-02
yhcC	88	0.7	0.25	6.68E-03	1.58E-02
yhhX	2064	0.5	0.19	6.83E-03	1.61E-02
uxuR	765	0.5	0.18	6.94E-03	1.63E-02
yahJ	474	0.5	0.17	7.35E-03	1.72E-02
flk	614	0.5	0.17	7.63E-03	1.78E-02
queA	535	1.0	0.44	7.68E-03	1.78E-02
torR	305	0.6	0.22	7.93E-03	1.84E-02
ygfI	140	0.6	0.25	8.03E-03	1.86E-02
dsbC	1048	0.5	0.16	8.80E-03	2.02E-02
uof	13	1.1	0.49	8.87E-03	2.04E-02
cueR	617	0.5	0.19	9.32E-03	2.13E-02
yieP	1007	0.7	0.26	9.51E-03	2.17E-02
fabI	13329	0.6	0.24	9.61E-03	2.19E-02
yjeN	48	0.7	0.30	9.61E-03	2.19E-02
rhsA	141	0.6	0.25	9.67E-03	2.20E-02
artP	1135	0.8	0.36	9.68E-03	2.20E-02
yejG	3862	0.5	0.20	9.92E-03	2.26E-02

ylbG	171	0.6	0.21	9.95E-03	2.26E-02
btuF	345	0.7	0.31	1.01E-02	2.29E-02
yeiI	784	0.5	0.16	1.04E-02	2.35E-02
yfiP	141	0.7	0.28	1.09E-02	2.45E-02
rarA	1592	0.5	0.18	1.09E-02	2.45E-02
cedA	80	0.7	0.30	1.11E-02	2.49E-02
ylaB	308	0.5	0.20	1.14E-02	2.56E-02
exbB	840	0.8	0.38	1.16E-02	2.61E-02
ycjZ	334	0.6	0.21	1.17E-02	2.63E-02
pagP	103	0.7	0.30	1.23E-02	2.75E-02
yhhA	398	0.7	0.30	1.24E-02	2.76E-02
afuC	79	0.6	0.26	1.24E-02	2.77E-02
narG	224	0.5	0.19	1.26E-02	2.81E-02
rnhA	471	0.6	0.21	1.28E-02	2.85E-02
ydbH	683	0.5	0.16	1.36E-02	3.00E-02
allR	1911	0.5	0.19	1.42E-02	3.14E-02
ubiG	1996	0.6	0.22	1.44E-02	3.17E-02
yfcT	56	0.6	0.28	1.44E-02	3.17E-02
comR	821	0.6	0.22	1.45E-02	3.19E-02
yegQ	2075	0.9	0.41	1.49E-02	3.26E-02
hepA	1910	0.4	0.16	1.52E-02	3.33E-02
proC	1191	0.5	0.18	1.54E-02	3.37E-02
nadD	439	0.6	0.27	1.64E-02	3.57E-02
caiA	136	0.5	0.21	1.65E-02	3.58E-02
kdsD	870	0.5	0.18	1.65E-02	3.59E-02
ydfT	184	0.6	0.22	1.67E-02	3.62E-02
yidI	133	0.6	0.24	1.67E-02	3.62E-02
yciY	237	0.6	0.26	1.68E-02	3.65E-02
ahpF	4340	0.7	0.33	1.74E-02	3.75E-02
prlC	3787	0.5	0.18	1.74E-02	3.75E-02
xerD	966	0.5	0.18	1.75E-02	3.76E-02
ydhI	38	0.7	0.32	1.75E-02	3.77E-02
yneL	30	0.7	0.34	1.79E-02	3.84E-02
yfcP	97	0.6	0.24	1.80E-02	3.85E-02
glpR	804	0.4	0.15	1.86E-02	3.98E-02
sapA	666	0.5	0.19	1.91E-02	4.07E-02
frsA	1419	0.4	0.16	1.93E-02	4.11E-02
ypdA	303	0.5	0.22	1.95E-02	4.14E-02
ycgE	1258	0.5	0.19	1.97E-02	4.20E-02
ispD	511	0.5	0.21	2.03E-02	4.30E-02
rof	2135	0.6	0.27	2.06E-02	4.36E-02
yjjA	555	0.6	0.27	2.14E-02	4.53E-02
nemA	591	0.6	0.24	2.26E-02	4.76E-02
L	74	0.6	0.24	2.36E-02	4.96E-02

Supplementary Tables

ptsG	16631	-4.5	0.16	2.81E-158	4.00E-156
nmpC	5011	-6.7	0.34	2.15E-85	7.88E-84
aroP	3946	-2.9	0.17	3.61E-64	9.15E-63
yobD	475	-3.0	0.21	5.14E-44	8.43E-43
glpK	3558	-3.2	0.26	3.16E-33	3.90E-32
atpG	18659	-2.2	0.19	3.57E-31	4.13E-30
nanA	308	-2.6	0.22	2.69E-29	2.96E-28
glpF	986	-2.8	0.26	1.24E-26	1.26E-25
atpF	20257	-1.7	0.16	1.49E-25	1.46E-24
atpD	35058	-2.4	0.23	3.26E-24	3.09E-23
eptB	3014	-1.9	0.18	1.36E-22	1.21E-21
dppC	979	-2.0	0.20	6.17E-22	5.38E-21
atpE	15165	-1.8	0.19	2.32E-20	1.89E-19
tsx	2685	-2.2	0.24	3.62E-20	2.92E-19
atpC	17212	-2.6	0.29	9.93E-20	7.85E-19
potF	2305	-1.9	0.20	4.26E-19	3.28E-18
yagF	316	-1.8	0.19	4.34E-19	3.33E-18
nupG	1009	-2.2	0.26	9.33E-18	6.80E-17
tisB	870	-2.6	0.32	6.34E-17	4.42E-16
yehU	342	-2.0	0.25	1.94E-16	1.33E-15
yihM	294	-2.0	0.25	3.98E-16	2.70E-15
cpdB	1608	-1.6	0.19	5.90E-16	3.99E-15
cspE	62471	-2.3	0.28	6.03E-16	4.07E-15
murP	253	-1.7	0.20	1.87E-15	1.22E-14
mltD	10758	-1.8	0.24	1.08E-13	6.60E-13
nanK	174	-1.7	0.22	1.42E-13	8.67E-13
mokB	73	-2.0	0.27	1.97E-13	1.19E-12
nlpC	1062	-1.5	0.19	2.02E-13	1.22E-12
potG	686	-1.7	0.23	3.85E-13	2.29E-12
atpA	36057	-1.6	0.22	4.07E-13	2.42E-12
atpH	13307	-1.6	0.22	4.39E-13	2.60E-12
murQ	314	-1.5	0.20	4.88E-13	2.87E-12
malX	180	-2.0	0.29	2.39E-12	1.37E-11
gltJ	872	-1.5	0.22	7.61E-12	4.27E-11
glmS	5908	-1.4	0.20	1.61E-11	8.91E-11
nuoC	6156	-1.9	0.29	1.69E-11	9.30E-11
yfeW	208	-1.3	0.19	1.70E-11	9.35E-11
nrdH	101	-3.2	0.49	3.10E-11	1.68E-10
dppB	1153	-1.7	0.25	4.35E-11	2.35E-10
glpT	272	-1.3	0.19	4.89E-11	2.64E-10
slmA	1065	-1.3	0.20	2.82E-10	1.46E-09
nrdB	3299	-1.1	0.16	3.09E-10	1.60E-09
yebK	827	-1.7	0.27	8.27E-10	4.16E-09
ulaC	92	-1.7	0.28	1.22E-09	6.09E-09

nuoA	3970	-2.2	0.37	1.42E-09	7.05E-09
iraP	1027	-1.5	0.25	2.04E-09	1.01E-08
ilvI	6581	-1.3	0.21	2.33E-09	1.15E-08
ilvH	3023	-1.8	0.30	2.76E-09	1.35E-08
eutS	20	-2.7	0.48	3.81E-09	1.83E-08
phoA	1075	-1.3	0.22	5.66E-09	2.68E-08
dosC	761	-1.4	0.24	9.17E-09	4.24E-08
yiaK	120	-1.5	0.26	1.39E-08	6.28E-08
uxaB	222	-1.3	0.21	1.40E-08	6.33E-08
potI	351	-1.5	0.26	2.05E-08	9.16E-08
yhjR	460	-1.4	0.24	2.28E-08	1.02E-07
znuA	4648	-0.9	0.16	2.40E-08	1.07E-07
fhuA	1522	-0.9	0.16	3.26E-08	1.44E-07
intB	1052	-1.4	0.25	6.98E-08	2.99E-07
modF	1895	-0.9	0.17	7.93E-08	3.39E-07
livG	1944	-1.3	0.24	1.15E-07	4.83E-07
livM	2742	-1.4	0.26	1.66E-07	6.90E-07
yobF	630	-1.2	0.21	1.89E-07	7.81E-07
deoA	622	-1.0	0.17	2.06E-07	8.48E-07
atpB	9235	-1.5	0.29	2.21E-07	9.07E-07
tatA	4337	-1.4	0.26	3.27E-07	1.33E-06
speE	1906	-1.0	0.18	3.60E-07	1.45E-06
ptsI	33656	-1.1	0.20	3.79E-07	1.52E-06
paaA	66	-1.4	0.28	3.90E-07	1.57E-06
ilvX	288	-1.8	0.38	3.98E-07	1.59E-06
livH	1459	-1.1	0.20	4.19E-07	1.67E-06
crr	31186	-1.1	0.21	4.50E-07	1.79E-06
potH	267	-1.3	0.26	4.57E-07	1.82E-06
rne	6929	-0.9	0.16	4.60E-07	1.83E-06
ydjF	367	-1.1	0.22	6.04E-07	2.38E-06
yiiR	246	-1.3	0.25	6.07E-07	2.39E-06
yjcO	1070	-1.0	0.19	6.20E-07	2.44E-06
yhcH	217	-1.0	0.20	6.72E-07	2.64E-06
rhtB	886	-1.2	0.25	7.01E-07	2.75E-06
glpQ	943	-1.3	0.26	7.53E-07	2.94E-06
ulaA	138	-1.2	0.24	7.58E-07	2.96E-06
uhpT	3491	-1.7	0.35	7.94E-07	3.09E-06
ulaD	141	-1.1	0.21	8.27E-07	3.21E-06
dosP	623	-1.0	0.19	8.67E-07	3.36E-06
eutP	22	-1.9	0.40	9.97E-07	3.84E-06
ulaB	56	-1.5	0.32	1.79E-06	6.74E-06
tdcD	119	-1.2	0.24	1.92E-06	7.21E-06
rpoB	85949	-1.0	0.21	2.00E-06	7.49E-06
lepB	4795	-1.0	0.21	2.30E-06	8.54E-06

livK	4297	-1.0	0.19	2.31E-06	8.55E-06
yiaL	25	-1.6	0.36	2.79E-06	1.03E-05
bcsG	1057	-0.8	0.16	3.08E-06	1.13E-05
yegX	339	-1.1	0.24	3.68E-06	1.34E-05
iscU	7089	-0.9	0.18	4.12E-06	1.49E-05
rpoC	91411	-1.2	0.25	4.74E-06	1.71E-05
iscA	8180	-1.0	0.22	5.07E-06	1.82E-05
tyrB	4886	-0.9	0.20	6.62E-06	2.35E-05
livF	2260	-1.1	0.24	6.84E-06	2.42E-05
bamA	19555	-1.0	0.21	8.10E-06	2.86E-05
tufB	14578	-1.0	0.21	1.12E-05	3.90E-05
potB	767	-0.8	0.17	1.16E-05	4.05E-05
gdhA	8612	-0.9	0.20	1.21E-05	4.21E-05
secF	4812	-0.9	0.21	1.23E-05	4.26E-05
yahB	168	-1.1	0.25	1.64E-05	5.63E-05
barA	1049	-0.8	0.17	1.94E-05	6.58E-05
tatB	3366	-0.9	0.19	2.23E-05	7.53E-05
yhgE	429	-0.8	0.19	2.36E-05	7.94E-05
add	533	-1.1	0.26	2.43E-05	8.17E-05
thiP	330	-0.9	0.22	2.45E-05	8.25E-05
mukE	1153	-0.8	0.19	2.75E-05	9.21E-05
dsbB	1345	-0.9	0.20	2.82E-05	9.43E-05
yegV	166	-1.0	0.23	2.82E-05	9.43E-05
yraN	674	-1.1	0.26	3.28E-05	1.09E-04
ygfQ	301	-0.8	0.20	3.29E-05	1.09E-04
lpoA	2947	-0.7	0.16	3.35E-05	1.11E-04
atpI	2008	-1.4	0.36	3.55E-05	1.17E-04
cdsA	2868	-0.8	0.17	3.70E-05	1.22E-04
bcsE	2002	-0.7	0.17	3.90E-05	1.28E-04
treA	239	-0.9	0.20	3.92E-05	1.29E-04
ispU	4831	-0.9	0.20	3.99E-05	1.31E-04
ftsZ	13115	-0.8	0.20	4.54E-05	1.48E-04
rcnA	1434	-1.1	0.28	4.71E-05	1.54E-04
rseP	5388	-0.8	0.18	4.97E-05	1.62E-04
ftsP	1933	-0.8	0.19	5.16E-05	1.68E-04
cusF	21	-1.6	0.42	5.32E-05	1.73E-04
nagK	1932	-0.9	0.23	5.49E-05	1.78E-04
thiQ	190	-0.9	0.22	6.93E-05	2.22E-04
yeiQ	468	-0.8	0.20	7.59E-05	2.42E-04
alaA	1851	-0.7	0.18	7.69E-05	2.45E-04
cmtB	36	-1.4	0.36	7.78E-05	2.48E-04
matP	705	-0.8	0.21	8.18E-05	2.59E-04
yrfF	3088	-0.9	0.22	8.65E-05	2.73E-04
dhaK	2124	-1.1	0.28	9.45E-05	2.97E-04

lpp	163479	-1.2	0.31	9.73E-05	3.06E-04
pntB	10310	-1.0	0.25	1.02E-04	3.18E-04
pepQ	4459	-0.7	0.16	1.06E-04	3.30E-04
ycbX	2466	-0.8	0.19	1.15E-04	3.58E-04
kdgR	2067	-0.8	0.21	1.16E-04	3.59E-04
pepA	5295	-0.7	0.16	1.18E-04	3.65E-04
rimJ	1231	-0.7	0.18	1.27E-04	3.92E-04
eamB	261	-1.0	0.27	1.41E-04	4.31E-04
dhaL	1327	-1.0	0.27	1.51E-04	4.60E-04
hlyE	79	-0.9	0.24	1.51E-04	4.61E-04
murC	2218	-0.7	0.18	1.60E-04	4.85E-04
fruA	2975	-1.0	0.27	1.63E-04	4.95E-04
pptA	121	-0.9	0.25	2.13E-04	6.40E-04
ftsK	7446	-0.8	0.21	2.47E-04	7.35E-04
yhjC	221	-1.0	0.28	2.50E-04	7.42E-04
yfhM	5934	-0.9	0.26	2.52E-04	7.48E-04
secY	62492	-0.7	0.18	2.69E-04	7.96E-04
dut	1702	-0.6	0.16	3.23E-04	9.46E-04
ridA	6352	-0.7	0.18	3.23E-04	9.46E-04
fruK	1365	-0.8	0.22	3.52E-04	1.03E-03
yhjY	142	-0.9	0.25	3.86E-04	1.12E-03
fucU	512	-1.0	0.28	3.89E-04	1.12E-03
ygcW	53	-1.3	0.41	4.33E-04	1.25E-03
nuoB	4733	-1.4	0.43	4.44E-04	1.28E-03
rplO	29211	-0.7	0.20	4.68E-04	1.34E-03
rpmD	5614	-0.6	0.17	4.68E-04	1.34E-03
idnK	68	-1.0	0.31	5.09E-04	1.45E-03
yciE	103	-1.0	0.30	5.31E-04	1.51E-03
ea8.5	852	-0.9	0.25	5.32E-04	1.52E-03
recB	1994	-0.7	0.20	5.35E-04	1.52E-03
rnd	1197	-0.9	0.25	5.40E-04	1.53E-03
spr	9899	-2.4	0.90	6.26E-04	1.77E-03
recD	1054	-0.9	0.25	6.49E-04	1.83E-03
nrdG	46	-1.0	0.32	6.50E-04	1.83E-03
hemY	2221	-0.6	0.18	6.84E-04	1.92E-03
yniC	750	-0.7	0.18	7.18E-04	2.01E-03
pagB	835	-0.8	0.22	8.34E-04	2.32E-03
hcaD	217	-0.8	0.24	9.12E-04	2.52E-03
kbaY	65	-0.9	0.28	9.22E-04	2.54E-03
cynS	85	-0.9	0.27	9.47E-04	2.61E-03
arcA	12499	-0.8	0.23	9.51E-04	2.62E-03
rpsE	28431	-0.6	0.17	9.86E-04	2.71E-03
torI	52	-1.0	0.33	9.98E-04	2.74E-03
sixA	4074	-0.7	0.21	1.08E-03	2.94E-03

pntA	14031	-0.7	0.21	1.15E-03	3.13E-03
tufA	53816	-0.7	0.21	1.19E-03	3.23E-03
allE	699	-0.8	0.23	1.20E-03	3.25E-03
infC	36821	-0.7	0.21	1.25E-03	3.36E-03
fimA	26894	-0.9	0.29	1.27E-03	3.42E-03
sbcC	1365	-0.8	0.26	1.31E-03	3.52E-03
shoB	223	-0.9	0.31	1.36E-03	3.64E-03
gnsB	484	-0.8	0.25	1.48E-03	3.96E-03
yeaX	177	-0.8	0.27	1.60E-03	4.24E-03
mrr	873	-0.7	0.23	1.62E-03	4.30E-03
ydcA	174	-0.7	0.22	1.63E-03	4.31E-03
cynT	60	-1.0	0.35	1.64E-03	4.35E-03
yibI	41	-0.9	0.30	1.89E-03	4.96E-03
aqpZ	296	-0.8	0.27	1.90E-03	4.97E-03
parC	3275	-0.6	0.20	1.90E-03	4.97E-03
fis	4379	-0.7	0.23	1.97E-03	5.14E-03
ftsQ	1593	-0.9	0.29	1.98E-03	5.16E-03
tsf	21349	-0.5	0.16	1.98E-03	5.16E-03
ihfB	11344	-0.6	0.18	2.14E-03	5.55E-03
ygcN	392	-0.8	0.28	2.16E-03	5.61E-03
ydiL	44	-0.9	0.31	2.27E-03	5.87E-03
fur	4932	-0.7	0.24	2.28E-03	5.89E-03
deoD	5557	-0.6	0.17	2.29E-03	5.90E-03
yjeJ	84	-0.8	0.27	2.29E-03	5.90E-03
eutK	91	-0.8	0.27	2.32E-03	5.98E-03
casC	173	-0.8	0.25	2.37E-03	6.10E-03
acpP	42390	-0.6	0.17	2.65E-03	6.80E-03
ygeI	18	-1.2	0.44	2.66E-03	6.82E-03
pepB	3131	-0.6	0.21	2.77E-03	7.06E-03
xylE	157	-0.8	0.26	2.86E-03	7.28E-03
thrC	4082	-0.6	0.19	2.94E-03	7.47E-03
pbpC	418	-0.6	0.17	3.08E-03	7.76E-03
lpxB	2495	-0.6	0.20	3.15E-03	7.92E-03
yphH	362	-0.6	0.18	3.17E-03	7.98E-03
intD	1282	-0.7	0.22	3.35E-03	8.38E-03
yebA	3723	-0.6	0.19	3.45E-03	8.63E-03
hns	43442	-0.7	0.25	3.48E-03	8.70E-03
dgoA	57	-0.9	0.32	3.52E-03	8.78E-03
psiF	153	-0.9	0.33	3.58E-03	8.93E-03
tatD	773	-0.7	0.22	3.59E-03	8.95E-03
yhdU	53	-2.0	1.02	3.60E-03	8.97E-03
yqiJ	115	-0.8	0.31	3.60E-03	8.97E-03
ygcU	117	-0.8	0.30	3.71E-03	9.19E-03
paaI	30	-1.0	0.40	3.73E-03	9.26E-03

Supplementary Tables

focA	599	-0.8	0.28	3.99E-03	9.83E-03
mntS	242	-1.1	0.45	4.03E-03	9.92E-03
yjfN	3034	-0.8	0.31	4.05E-03	9.95E-03
ydjG	214	-0.6	0.20	4.08E-03	1.00E-02
fimG	301	-0.8	0.30	4.16E-03	1.02E-02
avtA	2536	-0.6	0.22	4.33E-03	1.05E-02
sthA	5274	-0.5	0.18	4.46E-03	1.08E-02
chbR	566	-0.5	0.17	4.60E-03	1.11E-02
yeiL	57	-1.0	0.39	4.68E-03	1.13E-02
secD	8879	-0.5	0.18	4.73E-03	1.14E-02
rplS	17294	-0.5	0.15	5.14E-03	1.23E-02
yphA	89	-0.8	0.29	5.44E-03	1.30E-02
efeU_1	19	-1.1	0.49	5.77E-03	1.37E-02
yfjJ	48	-0.7	0.28	6.01E-03	1.43E-02
ytfP	1743	-0.7	0.24	6.06E-03	1.44E-02
yafY	81	-0.8	0.32	6.09E-03	1.44E-02
trmD	16252	-0.6	0.19	6.37E-03	1.51E-02
thrS	25477	-0.6	0.24	6.80E-03	1.60E-02
ahpC	14493	-0.6	0.24	6.83E-03	1.61E-02
folX	1011	-0.5	0.18	6.98E-03	1.64E-02
asmA	2081	-0.5	0.17	7.00E-03	1.64E-02
tas	2678	-0.7	0.26	7.20E-03	1.69E-02
alsE	62	-0.7	0.26	7.37E-03	1.72E-02
rplF	29122	-0.5	0.17	7.37E-03	1.72E-02
yihU	39	-0.8	0.33	7.76E-03	1.80E-02
yjbT	28	-0.8	0.34	8.28E-03	1.91E-02
alsC	58	-0.8	0.30	8.36E-03	1.93E-02
smtA	999	-0.5	0.16	8.51E-03	1.96E-02
hcaE	493	-0.5	0.17	8.63E-03	1.99E-02
rraA	6417	-0.7	0.26	8.65E-03	1.99E-02
menA	621	-0.5	0.18	8.82E-03	2.03E-02
ftsA	3574	-0.5	0.19	8.83E-03	2.03E-02
casE	162	-0.5	0.19	9.25E-03	2.12E-02
dcuS	806	-0.6	0.21	9.64E-03	2.20E-02
pqqL	172	-0.5	0.19	9.64E-03	2.20E-02
sugE	142	-0.6	0.25	1.05E-02	2.38E-02
yggX	1032	-0.5	0.20	1.08E-02	2.44E-02
yqfE	33	-0.8	0.32	1.11E-02	2.49E-02
yfcI	229	-0.6	0.25	1.16E-02	2.61E-02
ybcM	292	-0.6	0.22	1.17E-02	2.63E-02
kdgT	172	-0.5	0.21	1.20E-02	2.68E-02
fdnI	186	-0.6	0.25	1.21E-02	2.69E-02
slyX	492	-0.6	0.22	1.21E-02	2.70E-02
rplI	25312	-0.5	0.16	1.22E-02	2.72E-02

seqA	1900	-0.5	0.19	1.30E-02	2.88E-02
yfcO	844	-0.6	0.27	1.31E-02	2.90E-02
selB	1581	-0.5	0.21	1.32E-02	2.92E-02
nudB	2584	-0.5	0.17	1.32E-02	2.92E-02
rssA	624	-0.5	0.18	1.37E-02	3.04E-02
eutL	81	-0.7	0.28	1.48E-02	3.25E-02
yifK	825	-0.7	0.31	1.49E-02	3.28E-02
kduI	148	-0.5	0.21	1.53E-02	3.36E-02
metQ	21565	-0.6	0.28	1.56E-02	3.40E-02
hcaF	77	-0.6	0.25	1.73E-02	3.74E-02
ypfM	295	-0.7	0.29	1.74E-02	3.75E-02
fadR	2296	-0.6	0.25	1.75E-02	3.76E-02
lysR	199	-0.7	0.29	1.76E-02	3.80E-02
chbG	654	-0.6	0.23	1.80E-02	3.85E-02
ygbI	424	-0.6	0.24	1.81E-02	3.89E-02
hypE	229	-0.5	0.22	1.82E-02	3.91E-02
diaA	1273	-0.5	0.19	1.83E-02	3.91E-02
ilvA	940	-0.6	0.25	1.88E-02	4.02E-02
cusB	71	-0.6	0.28	1.94E-02	4.14E-02
fimF	358	-0.7	0.34	1.96E-02	4.17E-02
ydiJ	7309	-0.5	0.19	2.00E-02	4.25E-02
yibH	112	-0.6	0.23	2.00E-02	4.25E-02
yqhA	2000	-0.5	0.19	2.03E-02	4.30E-02
rplR	16092	-0.4	0.17	2.20E-02	4.64E-02
accD	11211	-0.4	0.15	2.28E-02	4.80E-02

Table S8: Differentially expressed genes in H<oFTN2> during fed-batch cultivation after 12 h of induction relative to the sample drawn immediately before induction of FTN2 expression. Genes also differentially expressed in wildtype HMS174(DE3) were excluded.

Gene	baseMean	log2FoldChange	lfcSE	pvalue	padj
pspA	14842	5.3	0.33	4.04E-57	5.14E-55
tauC	2355	6.9	0.35	7.31E-84	1.74E-81
pspD	963	4.8	0.33	9.18E-50	9.45E-48
ssuD	4342	7.3	0.28	2.88E-148	1.10E-144
mtr	436	2.2	0.22	2.17E-23	4.72E-22
pspG	406	4.2	0.38	1.76E-28	6.06E-27
ybfA	359	4.0	0.48	3.83E-18	5.95E-17
bhsA	97	3.1	0.42	4.22E-14	4.73E-13
ssuE	1793	7.6	0.34	8.03E-108	2.36E-105
tauA	2803	7.8	0.32	2.89E-128	2.20E-125
trpE	1170	4.4	0.26	5.06E-64	7.42E-62
mdtJ	525	6.0	0.49	8.78E-35	5.15E-33
tauB	3056	7.8	0.31	6.43E-142	8.18E-139

ybiJ	77	2.4	0.33	9.49E-14	1.01E-12
ssuA	2452	8.0	0.34	1.14E-118	5.45E-116
ssuB	1177	5.6	0.28	5.16E-90	1.31E-87
yohK	156	1.7	0.33	1.14E-07	6.72E-07
ssuC	1065	6.9	0.33	6.30E-93	1.72E-90
yhdV	324	3.5	0.38	9.32E-21	1.68E-19
alx	632	3.3	0.29	2.06E-30	8.37E-29
proP	805	0.7	0.26	7.33E-03	1.86E-02
sbp	8779	6.2	0.28	1.07E-109	3.41E-107
mdtI	424	5.0	0.46	2.03E-28	6.90E-27
sdaA	1258	3.0	0.30	1.19E-22	2.41E-21
inaA	270	1.2	0.25	1.91E-06	9.45E-06
tsgA	126	1.2	0.34	1.63E-04	5.91E-04
mntP	96	1.1	0.33	9.09E-04	2.84E-03
yacH	72	1.3	0.32	1.33E-05	5.80E-05
brnQ	615	1.5	0.23	3.53E-10	2.74E-09
ydeA	169	1.0	0.26	2.92E-04	1.01E-03
lpxP	682	2.7	0.29	4.61E-21	8.49E-20
yhbW	314	0.8	0.23	5.55E-04	1.83E-03
tqsA	106	1.2	0.32	7.84E-05	2.98E-04
cbl	1773	4.0	0.28	2.58E-47	2.23E-45
ydeH	862	3.5	0.30	2.73E-31	1.24E-29
trpD	1266	4.4	0.23	8.44E-81	1.79E-78
glnA	18421	3.0	0.30	6.38E-23	1.32E-21
hslU	3892	1.0	0.23	7.99E-06	3.58E-05
nepI	244	2.2	0.27	6.26E-16	8.12E-15
marB	141	2.1	0.42	8.57E-08	5.13E-07
psiE	240	3.6	0.36	9.67E-25	2.41E-23
yebE	4926	4.2	0.29	6.94E-49	6.79E-47
nac	66	0.8	0.32	1.30E-02	3.09E-02
cyaA	4223	1.5	0.21	3.70E-12	3.55E-11
deaD	7944	1.5	0.34	7.81E-06	3.51E-05
ibpB	603	3.1	0.32	4.28E-23	9.06E-22
tadA	313	1.2	0.23	1.16E-06	5.91E-06
rpoH	6137	1.6	0.26	5.18E-09	3.61E-08
yedR	43	2.0	0.40	6.59E-08	4.01E-07
glpD	1120	2.0	0.23	5.30E-17	7.46E-16
amtB	283	0.7	0.25	3.70E-03	1.01E-02
asr	1668	6.4	0.35	4.44E-73	7.36E-71
mdtL	995	1.8	0.34	5.40E-08	3.31E-07
marR	142	2.7	0.48	2.52E-09	1.80E-08
marA	352	2.4	0.44	6.23E-09	4.33E-08
rstA	476	-0.8	0.23	1.06E-03	3.27E-03
cspA	10155	3.6	0.40	3.47E-20	6.09E-19

pdhR	1482	1.0	0.29	2.73E-04	9.51E-04
iap	400	1.9	0.25	7.66E-14	8.29E-13
yjdP	139	1.7	0.28	2.83E-09	2.00E-08
degP	16993	2.5	0.28	8.18E-19	1.31E-17
ibpA	1273	1.7	0.28	4.88E-10	3.71E-09
glnK	23	1.9	0.50	2.18E-05	9.17E-05
wcaC	51	1.5	0.42	1.37E-04	5.02E-04
ybeD	1561	1.1	0.25	2.56E-05	1.06E-04
cysN	7157	2.1	0.21	9.49E-24	2.14E-22
cysC	1381	1.9	0.22	1.94E-16	2.58E-15
eno	34050	1.1	0.28	8.79E-05	3.30E-04
hslV	839	1.0	0.25	4.50E-05	1.80E-04
wzb	28	3.6	0.73	1.52E-08	1.02E-07
yidZ	525	1.3	0.32	4.43E-05	1.77E-04
cpxP	15648	3.0	0.36	2.20E-17	3.21E-16
chaA	1220	2.8	0.28	9.82E-23	1.99E-21
ybjH	285	1.2	0.27	1.81E-05	7.75E-05
azuC	22	2.5	0.58	1.79E-06	8.88E-06
gpmM	2846	1.2	0.27	1.09E-05	4.81E-05
csdA	463	1.0	0.20	3.09E-06	1.47E-05
ycjF	635	1.0	0.23	3.69E-05	1.49E-04
ycfS	3052	3.6	0.29	3.97E-36	2.40E-34
hha	938	2.8	0.40	5.27E-13	5.36E-12
yciW	2374	3.0	0.29	1.11E-24	2.72E-23
yrbN	96	2.3	0.38	8.22E-10	6.06E-09
cysK	66542	1.7	0.23	2.99E-13	3.11E-12
htpX	22361	2.6	0.28	2.68E-20	4.77E-19
grpE	5743	1.1	0.26	2.46E-05	1.02E-04
yibD	81	2.1	0.35	6.63E-10	4.97E-09
cspI	346	4.0	0.29	7.41E-44	6.01E-42
csdE	222	0.8	0.23	1.84E-03	5.36E-03
lipB	453	0.6	0.24	2.05E-02	4.63E-02
pstS	10868	4.6	0.34	9.56E-43	7.43E-41
dacC	3106	2.4	0.26	4.44E-20	7.73E-19
yobB	1101	3.3	0.29	6.75E-30	2.63E-28
proY	562	1.3	0.29	7.11E-06	3.22E-05
nfsA	679	0.8	0.24	7.21E-04	2.30E-03
yncD	220	1.0	0.29	6.99E-04	2.24E-03
ybeZ	1859	0.8	0.21	5.82E-04	1.90E-03
cysI	9839	1.9	0.24	2.75E-15	3.38E-14
yebO	486	1.2	0.30	3.33E-05	1.35E-04
ypdK	108	2.2	0.37	4.86E-10	3.70E-09
aaeR	237	0.9	0.27	1.37E-03	4.11E-03
cysU	2166	2.1	0.23	8.49E-20	1.45E-18

acrA	3554	0.7	0.21	2.86E-03	8.02E-03
ybbN	2568	0.6	0.22	8.63E-03	2.16E-02
cysW	2429	2.0	0.23	5.93E-18	9.08E-17
cysD	5322	2.9	0.30	3.03E-21	5.66E-20
yqaE	182	2.7	0.41	9.53E-12	8.71E-11
mutM	298	1.0	0.26	8.74E-05	3.29E-04
soxS	2018	1.9	0.33	7.71E-09	5.31E-08
acrD	929	2.6	0.25	1.46E-23	3.27E-22
serC	13546	0.9	0.22	1.06E-04	3.94E-04
yfgG	499	2.4	0.36	3.71E-11	3.19E-10
typA	11985	1.7	0.26	7.09E-11	6.01E-10
hslO	1336	1.0	0.23	1.62E-05	6.99E-05
yeeO	218	0.7	0.24	6.69E-03	1.71E-02
cysH	3995	1.9	0.21	5.52E-20	9.56E-19
pinQ	27	1.5	0.41	1.09E-04	4.05E-04
dsbA	6263	2.0	0.29	8.99E-12	8.25E-11
ybjC	138	1.2	0.36	2.74E-04	9.55E-04
glnG	1194	1.5	0.30	9.26E-07	4.79E-06
cho	141	0.9	0.26	5.59E-04	1.84E-03
edd	521	0.6	0.23	1.93E-02	4.39E-02
maa	491	2.5	0.41	1.57E-10	1.27E-09
cysJ	8888	2.2	0.21	9.18E-25	2.32E-23
pgi	5869	0.9	0.25	3.41E-04	1.17E-03
gapA	37027	1.6	0.35	3.11E-06	1.48E-05
yhhS	159	1.3	0.25	1.14E-06	5.80E-06
bioA	648	1.8	0.37	4.16E-07	2.26E-06
ygbE	597	1.6	0.22	1.65E-13	1.74E-12
ybjG	363	0.8	0.31	1.15E-02	2.77E-02
mdtD	118	0.7	0.27	5.98E-03	1.54E-02
pstC	2154	3.3	0.26	3.57E-37	2.39E-35
hslR	542	0.6	0.26	2.00E-02	4.54E-02
wcaL	95	0.9	0.35	5.48E-03	1.44E-02
serS	5760	0.8	0.25	2.85E-03	8.01E-03
lpxL	677	1.1	0.21	1.45E-06	7.28E-06
yiiX	212	1.1	0.26	1.32E-05	5.79E-05
hemB	1787	0.6	0.23	1.36E-02	3.23E-02
ygaC	228	1.2	0.37	6.10E-04	1.99E-03
fimB	233	0.8	0.26	1.39E-03	4.17E-03
tilS	531	0.6	0.25	1.07E-02	2.61E-02
gnd	6660	0.8	0.25	1.97E-03	5.69E-03
yaiW	1065	1.5	0.24	2.46E-10	1.93E-09
yiaU	202	0.9	0.26	5.64E-04	1.86E-03
pfkA	4394	0.9	0.24	2.06E-04	7.30E-04
yfcJ	123	1.6	0.26	3.83E-10	2.96E-09

rimK	554	1.2	0.22	2.21E-07	1.25E-06
cysM	3091	2.1	0.26	2.88E-15	3.52E-14
yfcL	591	1.4	0.28	2.32E-07	1.31E-06
iaaA	2602	1.0	0.20	1.77E-06	8.78E-06
mliC	602	1.3	0.28	5.92E-06	2.72E-05
wcaJ	75	1.3	0.42	6.94E-04	2.22E-03
yidA	764	1.0	0.29	6.81E-04	2.19E-03
sfsB	102	0.9	0.29	2.38E-03	6.80E-03
yhiR	445	1.2	0.27	1.17E-05	5.18E-05
ftsH	21735	1.5	0.21	8.95E-12	8.24E-11
aroA	3502	1.4	0.27	5.46E-07	2.92E-06
ybhB	647	1.1	0.25	2.67E-05	1.10E-04
glnL	738	1.5	0.31	2.70E-07	1.51E-06
rdoA	3595	1.2	0.25	1.20E-06	6.09E-06
tesB	819	1.6	0.23	1.03E-11	9.36E-11
yccA	24430	1.7	0.27	9.50E-11	7.87E-10
nudJ	69	0.9	0.32	3.86E-03	1.06E-02
yeeD	3842	3.1	0.28	1.80E-26	5.21E-25
prfA	647	1.2	0.24	6.60E-07	3.50E-06
ybjS	432	0.9	0.24	4.52E-04	1.52E-03
yecD	1107	1.7	0.28	7.88E-10	5.83E-09
yncJ	86	3.7	0.45	3.80E-17	5.38E-16
ribC	1618	1.2	0.25	3.05E-06	1.46E-05
gadA	32	-0.9	0.37	8.49E-03	2.13E-02
ribB	1566	0.7	0.24	6.32E-03	1.63E-02
ycbB	1032	1.0	0.24	4.80E-05	1.91E-04
pykF	7373	1.0	0.30	4.56E-04	1.53E-03
ydjM	27	1.3	0.42	4.82E-04	1.60E-03
tusA	268	1.1	0.37	1.29E-03	3.89E-03
phoB	1310	3.1	0.26	1.98E-30	8.11E-29
ydhC	118	1.0	0.27	1.99E-04	7.11E-04
bioB	1111	0.7	0.24	4.68E-03	1.25E-02
rnb	4821	1.9	0.28	2.22E-11	1.95E-10
dcrB	4791	0.9	0.24	3.75E-04	1.28E-03
yeeE	3909	3.2	0.29	1.38E-27	4.40E-26
sbmA	734	1.8	0.27	2.70E-11	2.36E-10
ygaH	244	1.2	0.24	2.61E-06	1.26E-05
pflA	940	1.1	0.30	3.38E-04	1.16E-03
fliY	10008	1.4	0.22	2.54E-09	1.81E-08
tolB	8328	1.0	0.24	2.20E-05	9.24E-05
rluE	179	0.8	0.33	1.02E-02	2.50E-02
nlpA	2461	1.8	0.23	2.17E-14	2.49E-13
baeS	146	0.7	0.25	7.27E-03	1.85E-02
thiI	1107	1.9	0.32	2.30E-09	1.65E-08

yneM	253	-1.0	0.31	7.99E-04	2.52E-03
yfgH	36	1.1	0.39	1.45E-03	4.34E-03
phoP	1000	-0.6	0.21	9.23E-03	2.28E-02
yhfG	70	1.0	0.48	1.66E-02	3.85E-02
hflK	5154	1.3	0.20	1.89E-10	1.51E-09
hpt	1683	0.8	0.23	4.23E-04	1.43E-03
tomB	992	1.8	0.34	4.18E-08	2.63E-07
glpG	535	1.0	0.30	8.73E-04	2.74E-03
wzxC	67	1.0	0.39	4.51E-03	1.21E-02
ptrB	532	1.0	0.24	2.05E-05	8.71E-05
yfaZ	122	0.6	0.26	1.84E-02	4.21E-02
smpB	1366	1.3	0.25	7.33E-07	3.86E-06
dacA	3579	1.6	0.29	3.75E-08	2.38E-07
ycfH	638	0.7	0.25	2.91E-03	8.15E-03
cvrA	365	0.9	0.23	1.73E-04	6.23E-04
yeeX	6591	0.6	0.25	1.19E-02	2.85E-02
gsiA	2201	0.6	0.19	4.05E-03	1.10E-02
dadA	4085	-1.3	0.28	1.51E-06	7.55E-06
ygaZ	804	1.8	0.34	7.89E-08	4.74E-07
greB	379	1.1	0.22	1.85E-06	9.16E-06
phoR	843	2.8	0.27	5.62E-25	1.47E-23
ydgU	44	5.7	0.92	1.08E-11	9.80E-11
yfcA	444	0.7	0.24	7.34E-03	1.86E-02
amiC	1150	0.8	0.22	5.72E-04	1.88E-03
usg	1321	0.9	0.23	3.35E-04	1.15E-03
tolR	823	0.9	0.23	2.55E-04	8.96E-04
trmL	68	1.5	0.41	7.21E-05	2.76E-04
ea59_1	638	1.0	0.38	5.24E-03	1.38E-02
yfcM	523	1.0	0.25	1.62E-04	5.87E-04
fldA	2944	1.7	0.34	3.57E-07	1.96E-06
tusE	983	2.0	0.39	1.38E-07	8.05E-07
yaeB	412	1.2	0.45	2.26E-03	6.49E-03
ycbC	264	0.6	0.24	1.15E-02	2.77E-02
ydfU	30	1.2	0.40	1.18E-03	3.60E-03
ispH	1512	1.1	0.31	2.65E-04	9.29E-04
sppA	2257	1.1	0.22	2.99E-06	1.43E-05
panD	1965	0.8	0.26	2.67E-03	7.53E-03
lolB	593	1.4	0.32	9.65E-06	4.30E-05
grxA	306	0.9	0.31	1.99E-03	5.74E-03
tolQ	1047	1.6	0.31	2.82E-07	1.57E-06
purD	1544	1.2	0.24	5.38E-07	2.88E-06
hemA	1288	1.3	0.28	7.07E-06	3.20E-05
purH	1621	1.8	0.33	4.48E-08	2.79E-07
ratB	235	0.6	0.28	2.15E-02	4.83E-02

cdh	455	3.6	0.36	8.65E-24	1.99E-22
glpE	450	1.3	0.26	3.60E-07	1.97E-06
yciH	363	1.9	0.28	1.32E-11	1.18E-10
ycaL	120	1.9	0.30	2.61E-10	2.04E-09
yadG	872	0.6	0.25	2.14E-02	4.83E-02
yagI	561	0.6	0.22	1.87E-02	4.27E-02
rsuA	810	2.1	0.39	3.96E-08	2.50E-07
yaeQ	314	0.8	0.26	1.65E-03	4.87E-03
rlmF	474	0.7	0.24	5.46E-03	1.43E-02
ycfD	2976	0.7	0.21	1.11E-03	3.40E-03
ydhJ	96	1.3	0.35	6.37E-05	2.47E-04
gmk	1239	1.5	0.23	2.61E-10	2.04E-09
ydcO	179	1.1	0.29	1.05E-04	3.91E-04
ydgI	216	1.1	0.28	1.46E-04	5.31E-04
proW	105	-0.7	0.30	1.55E-02	3.62E-02
yqjI	264	0.7	0.26	5.51E-03	1.44E-02
pqiA	371	1.0	0.23	3.90E-05	1.57E-04
leuO	178	0.6	0.27	1.97E-02	4.47E-02
apbE	320	0.9	0.26	3.32E-04	1.14E-03
yejH	266	0.6	0.22	8.45E-03	2.12E-02
yecE	593	1.7	0.37	1.03E-06	5.29E-06
rimI	217	0.9	0.32	3.94E-03	1.08E-02
cysA	7433	1.6	0.28	9.96E-09	6.74E-08
guaA	7465	0.9	0.24	1.44E-04	5.24E-04
ibsC	32	-1.1	0.40	2.99E-03	8.34E-03
yigL	458	1.1	0.33	4.73E-04	1.58E-03
rcnB	1284	1.4	0.29	1.20E-06	6.09E-06
purK	1070	1.6	0.32	2.77E-07	1.55E-06
menF	503	0.7	0.31	1.39E-02	3.28E-02
kdsC	947	0.6	0.22	4.96E-03	1.32E-02
yeaD	916	1.0	0.24	6.33E-05	2.45E-04
yigM	334	1.0	0.43	8.15E-03	2.05E-02
iscX	709	0.9	0.28	7.15E-04	2.28E-03
yjjG	342	0.8	0.29	3.76E-03	1.03E-02
yciC	1489	1.6	0.22	1.26E-11	1.14E-10
dadX	2510	-1.4	0.26	2.14E-07	1.21E-06
folB	75	1.8	0.37	5.31E-07	2.86E-06
argS	3670	0.7	0.21	2.57E-03	7.28E-03
yjaG	855	1.2	0.27	4.61E-06	2.15E-05
yeiR	179	1.0	0.31	6.01E-04	1.96E-03
rdgB	611	1.9	0.33	8.48E-09	5.80E-08
hemH	236	0.9	0.24	4.24E-04	1.43E-03
rimN	605	0.8	0.35	1.33E-02	3.15E-02
metJ	1694	1.4	0.34	2.67E-05	1.10E-04

mmuP	481	1.2	0.24	9.08E-07	4.70E-06
aat	507	0.9	0.22	1.68E-04	6.06E-04
rlmH	824	1.4	0.37	8.47E-05	3.19E-04
dtpB	109	-1.3	0.31	2.94E-05	1.20E-04
mgtL	73	-0.8	0.38	1.40E-02	3.29E-02
rlmC	257	0.9	0.32	3.95E-03	1.08E-02
metF	4955	2.3	0.43	1.64E-08	1.09E-07
hflC	4749	1.3	0.21	6.41E-09	4.44E-08
mdoC	261	2.1	0.34	5.88E-10	4.44E-09
ybfE	149	1.1	0.32	5.61E-04	1.85E-03
cdgR	295	0.6	0.26	1.59E-02	3.69E-02
yneG	88	1.0	0.44	7.51E-03	1.90E-02
metK	4779	1.5	0.86	1.02E-02	2.49E-02
pstA	1435	2.9	0.28	9.00E-24	2.05E-22
rcsF	635	1.0	0.31	6.25E-04	2.03E-03
fkpB	838	1.0	0.28	2.78E-04	9.67E-04
nusB	2094	1.2	0.38	8.12E-04	2.56E-03
insH-6	43	1.0	0.36	3.48E-03	9.58E-03
hemF	556	1.6	0.30	4.01E-08	2.53E-07
prmC	233	0.8	0.25	1.67E-03	4.91E-03
rsmG	319	1.4	0.34	2.72E-05	1.12E-04
recF	396	1.1	0.28	8.96E-05	3.35E-04
cpxA	2003	0.5	0.22	2.17E-02	4.87E-02
trxB	3756	0.8	0.24	1.38E-03	4.15E-03
cmoA	381	1.2	0.38	5.41E-04	1.79E-03
ybjL	720	0.6	0.20	2.54E-03	7.20E-03
yggW	682	1.4	0.22	2.05E-10	1.63E-09
pabA	118	0.8	0.33	9.65E-03	2.38E-02
proB	2200	0.5	0.21	1.67E-02	3.86E-02
cysP	4996	2.8	0.77	1.39E-05	6.04E-05
amiA	915	1.6	0.28	5.19E-09	3.61E-08
mlaA	1796	0.9	0.27	1.07E-03	3.30E-03
yqeI	65	1.6	0.36	5.20E-06	2.39E-05
yceG	642	0.8	0.22	4.63E-04	1.55E-03
erpA	3908	0.8	0.27	2.84E-03	7.98E-03
rimP	1847	1.5	0.28	1.09E-07	6.41E-07
fau	508	0.8	0.27	4.50E-03	1.21E-02
clpP	4737	0.7	0.22	4.24E-03	1.15E-02
lolD	929	0.8	0.21	3.57E-04	1.22E-03
yehS	549	1.2	0.29	4.71E-05	1.87E-04
miaA	7987	0.8	0.21	7.06E-04	2.25E-03
yeaP	677	1.3	0.32	6.59E-05	2.54E-04
gpt	1718	1.8	0.86	3.43E-03	9.44E-03
fdx	992	0.8	0.25	9.32E-04	2.91E-03

purU	1051	1.9	0.38	3.24E-07	1.79E-06
holE	130	1.1	0.28	5.38E-05	2.13E-04
ybeB	899	1.6	0.32	5.44E-07	2.91E-06
ybhA	306	1.2	0.47	4.06E-03	1.10E-02
aaeA	86	1.5	0.33	2.11E-06	1.03E-05
yibL	535	1.0	0.26	6.57E-05	2.53E-04
cmk	1790	1.1	0.25	2.60E-05	1.08E-04
pbpG	686	1.4	0.25	5.58E-08	3.41E-07
exoX	807	0.7	0.23	2.12E-03	6.12E-03
rnt	333	1.0	0.44	1.11E-02	2.69E-02
gltF	95	1.2	0.31	4.84E-05	1.92E-04
trxA	8194	0.7	0.28	1.08E-02	2.62E-02
ybdL	1268	1.2	0.26	6.25E-06	2.86E-05
ispE	1014	0.6	0.21	9.14E-03	2.27E-02
asnS	9671	0.7	0.24	4.91E-03	1.30E-02
lolC	936	1.2	0.29	5.41E-05	2.13E-04
ribE	1672	1.0	0.36	3.54E-03	9.71E-03
rsmC	755	1.1	0.28	6.98E-05	2.67E-04
yciB	1451	1.3	0.23	1.69E-08	1.12E-07
pth	450	1.1	0.35	7.63E-04	2.42E-03
yodB	107	2.4	0.32	2.16E-14	2.48E-13
nusA	8129	1.0	0.21	1.37E-05	5.97E-05
pheS	2255	1.4	0.31	2.66E-06	1.29E-05
djlA	437	0.7	0.26	5.71E-03	1.49E-02
yneH	524	0.9	0.29	1.67E-03	4.91E-03
rnc	985	1.3	0.34	6.91E-05	2.66E-04
ppiA	1538	1.6	0.35	3.69E-06	1.74E-05
mrdA	991	1.6	0.38	6.85E-06	3.11E-05
yfiH	550	0.7	0.22	1.59E-03	4.70E-03
yccF	269	0.9	0.31	3.94E-03	1.08E-02
guaC	3207	0.6	0.24	1.36E-02	3.22E-02
grxD	3434	1.1	0.25	1.70E-05	7.32E-05
gsk	1014	0.8	0.26	1.74E-03	5.10E-03
yjeK	625	0.7	0.27	7.93E-03	2.00E-02
ybbA	758	1.9	0.27	7.75E-12	7.15E-11
gntK	45	1.3	0.35	8.90E-05	3.33E-04
purE	462	1.4	0.83	1.48E-02	3.48E-02
lptF	1094	0.6	0.21	1.29E-02	3.06E-02
yfiC	292	1.0	0.37	4.11E-03	1.11E-02
yjjX	272	0.9	0.26	5.77E-04	1.89E-03
hscA	2954	0.7	0.23	5.82E-03	1.52E-02
cydD	980	0.6	0.24	1.79E-02	4.13E-02
yeeN	973	1.3	0.31	1.28E-05	5.63E-05
mutL	1318	0.7	0.22	1.96E-03	5.67E-03

yecT	45	1.8	0.46	2.14E-05	9.06E-05
holD	215	1.0	0.28	2.42E-04	8.53E-04
hisG	2510	1.0	0.22	2.04E-05	8.64E-05
ygjP	126	1.1	0.35	1.00E-03	3.12E-03
phnE_1	33	2.0	0.46	2.52E-06	1.22E-05
actP	32032	-4.3	0.38	1.08E-30	4.62E-29
dctA	30299	-3.7	0.25	2.33E-48	2.12E-46
gatB	14573	-1.4	0.28	7.74E-07	4.07E-06
gatA	25557	-1.4	0.33	1.34E-05	5.87E-05
gatD	15509	-1.4	0.34	2.30E-05	9.65E-05
yjcH	6053	-4.1	0.38	2.65E-27	8.08E-26
gatZ	71919	-1.2	0.29	2.80E-05	1.15E-04
acs	54543	-3.3	0.31	8.84E-28	2.83E-26
glcA	4317	-3.0	0.25	5.83E-33	3.01E-31
malT	38205	-2.7	0.23	2.36E-30	9.48E-29
aldA	203311	-3.0	0.27	6.02E-28	1.98E-26
gatC	76910	-1.8	0.34	4.97E-08	3.07E-07
yghZ	2098	-2.7	0.26	4.75E-26	1.35E-24
cstA	66653	-3.8	0.36	1.65E-27	5.21E-26
fadI	3375	-0.5	0.22	2.00E-02	4.54E-02
agp	5454	-3.4	0.29	1.30E-31	6.07E-30
yqeF	4870	-1.3	0.24	2.56E-08	1.64E-07
gatY	49064	-0.6	0.24	9.76E-03	2.40E-02
manX	28846	-0.8	0.28	5.71E-03	1.49E-02
paaK	3077	-2.8	0.25	8.66E-30	3.30E-28
malE	3819	-4.5	0.38	4.02E-33	2.16E-31
mhpR	2200	-3.1	0.28	1.18E-28	4.17E-27
mglC	1314	-2.2	0.32	4.11E-12	3.91E-11
yedF	4922	-2.3	0.25	3.30E-19	5.43E-18
uxaC	1928	-2.1	0.20	3.66E-25	9.75E-24
mtlA	6470	-1.7	0.27	2.23E-10	1.76E-09
manY	21396	-0.7	0.28	1.37E-02	3.24E-02
ydcH	3471	-2.7	0.33	8.97E-17	1.23E-15
feaR	2560	-1.9	0.24	1.28E-15	1.59E-14
pck	31140	-1.5	0.26	8.92E-09	6.09E-08
manZ	30931	-0.8	0.28	3.62E-03	9.94E-03
pka	9649	-2.9	0.24	4.99E-32	2.44E-30
xylF	1100	-3.4	0.26	1.00E-39	7.07E-38
aceA	79758	-1.8	0.25	5.13E-13	5.23E-12
aceK	12154	-1.6	0.23	2.59E-11	2.27E-10
lamB	1174	-4.0	0.32	1.66E-36	1.05E-34
mtfA	11138	-2.0	0.25	1.02E-14	1.19E-13
ompF	259534	-1.5	0.22	1.14E-10	9.35E-10
uxuA	1148	-2.1	0.22	1.81E-21	3.46E-20

ybdD	3177	-3.2	0.29	1.75E-29	6.53E-28
nagE	11065	-1.6	0.27	1.99E-09	1.44E-08
glnH	46915	-2.3	0.22	3.15E-25	8.58E-24
ytfQ	1583	-3.5	0.23	8.71E-49	8.30E-47
fadJ	7687	-1.1	0.26	5.72E-05	2.25E-04
fadL	5490	-1.1	0.27	5.55E-05	2.18E-04
putP	4743	-2.7	0.25	9.62E-26	2.68E-24
aldB	1762	-3.8	0.28	6.86E-42	5.13E-40
cycA	13924	-2.0	0.30	1.25E-11	1.12E-10
aspA	6415	-1.4	0.20	1.70E-11	1.50E-10
araF	655	-2.8	0.24	2.22E-29	8.21E-28
rihC	1003	-2.2	0.20	1.14E-26	3.40E-25
paaJ	2583	-2.4	0.28	3.36E-17	4.80E-16
ytfJ	1366	-1.0	0.25	6.57E-05	2.53E-04
uxaA	1726	-2.1	0.23	1.44E-19	2.44E-18
frdB	1361	-2.8	0.26	1.53E-26	4.45E-25
glcF	4679	-2.6	0.25	9.15E-25	2.32E-23
aceB	43840	-1.9	0.26	1.03E-12	1.03E-11
mglA	2221	-2.1	0.41	1.48E-07	8.63E-07
glcE	4014	-2.5	0.25	2.42E-23	5.23E-22
lhgO	650	-2.6	0.29	3.18E-19	5.24E-18
sdhA	21249	-1.3	0.27	2.26E-06	1.10E-05
malM	635	-3.4	0.30	5.10E-31	2.24E-29
frdA	2867	-2.3	0.24	1.23E-20	2.21E-19
cspD	90109	-3.2	0.28	2.87E-29	1.04E-27
frdC	856	-2.8	0.27	4.47E-24	1.05E-22
rnk	4648	-1.0	0.19	5.35E-07	2.87E-06
sdhB	18505	-1.6	0.29	2.20E-08	1.42E-07
malK	339	-3.7	0.32	9.59E-32	4.51E-30
mppA	2076	-1.4	0.23	1.16E-09	8.46E-09
ygiS	3855	-1.1	0.24	8.02E-06	3.59E-05
yfaW	547	-3.3	0.30	1.55E-28	5.38E-27
csiE	590	-2.1	0.39	4.21E-08	2.64E-07
ysgA	1694	-1.0	0.24	4.11E-05	1.65E-04
yidQ	4480	-2.2	0.22	2.15E-23	4.72E-22
uspF	6748	-2.7	0.28	4.64E-22	9.11E-21
fadE	11553	1.2	0.23	1.93E-07	1.10E-06
malF	404	-3.3	0.31	2.52E-27	7.74E-26
glnQ	10031	-1.8	0.19	3.11E-19	5.17E-18
maeB	13689	-1.1	0.22	1.93E-06	9.48E-06
yedE	6253	-1.5	0.27	7.74E-08	4.66E-07
ddpA	707	-2.6	0.27	6.84E-21	1.25E-19
galS	452	-1.9	0.27	3.50E-12	3.36E-11
fucO	1641	-3.0	0.29	5.62E-25	1.47E-23

glcD	6522	-2.5	0.23	6.14E-26	1.73E-24
yihY	874	-1.9	0.26	3.65E-13	3.76E-12
fumA	14112	-2.3	0.27	6.13E-17	8.55E-16
ydcI	6364	-2.0	0.21	6.98E-20	1.20E-18
glnP	3910	-1.9	0.24	1.21E-14	1.41E-13
fucR	1617	-1.7	0.21	3.86E-16	5.05E-15
bsmA	1245	-2.4	0.28	7.54E-18	1.14E-16
csgG	701	-1.8	0.27	3.82E-11	3.28E-10
rmuC	1231	-2.2	0.28	1.91E-15	2.37E-14
chbC	1222	-2.1	0.24	1.22E-18	1.93E-17
frdD	1334	-2.7	0.26	4.85E-24	1.13E-22
ugpB	2179	-1.1	0.23	2.74E-06	1.32E-05
ytfR	530	-3.0	0.31	7.66E-23	1.57E-21
gatR_2	1421	-1.1	0.32	3.53E-04	1.21E-03
gabD	832	-2.1	0.33	2.30E-10	1.82E-09
yaiZ	497	-2.2	0.27	5.02E-16	6.54E-15
fucA	214	-2.5	0.26	3.37E-21	6.27E-20
sdhC	7684	-0.9	0.27	1.45E-03	4.33E-03
yjjM	199	-2.1	0.29	1.65E-13	1.74E-12
nrdF	393	-1.8	0.38	3.93E-07	2.15E-06
gabT	1486	-2.3	0.34	3.41E-12	3.28E-11
xdhB	212	-3.1	0.31	3.01E-23	6.41E-22
nuoF	9817	-1.1	0.26	7.39E-05	2.82E-04
ybhQ	4474	-2.6	0.33	3.22E-15	3.90E-14
srlA	200	-2.8	0.34	1.33E-16	1.80E-15
fecA	456	-2.6	0.24	1.92E-25	5.27E-24
lsrA	240	-2.4	0.28	7.70E-17	1.06E-15
araC	1294	-1.6	0.23	1.46E-11	1.30E-10
melR	231	-1.9	0.29	1.35E-10	1.10E-09
atoC	417	-2.1	0.27	1.25E-15	1.56E-14
kbaZ	266	-2.1	0.27	1.17E-14	1.37E-13
fucI	444	-2.3	0.26	9.01E-18	1.36E-16
paaY	1038	-1.7	0.20	7.34E-17	1.01E-15
putA	3025	-2.1	0.29	1.61E-12	1.59E-11
ivbL	1483	-1.4	0.31	2.17E-06	1.06E-05
agaV	257	-2.5	0.27	2.88E-20	5.10E-19
malG	286	-2.9	0.34	6.53E-18	9.92E-17
ydbC	770	-2.4	0.26	4.08E-21	7.56E-20
nuoE	4505	-1.1	0.25	2.01E-05	8.55E-05
sgcX	206	-2.9	0.30	3.78E-22	7.47E-21
sdhD	3113	-0.9	0.28	7.58E-04	2.41E-03
ygjI	104	-2.3	0.31	3.05E-13	3.16E-12
ddpX	224	-2.0	0.30	1.05E-11	9.49E-11
hisJ	9318	-1.7	0.24	5.09E-12	4.78E-11

dmlR	810	-1.5	0.27	1.25E-08	8.42E-08
nuoG	17101	-1.0	0.27	2.43E-04	8.57E-04
lsrR	549	-1.9	0.21	3.93E-19	6.40E-18
ppsA	20921	-0.8	0.23	1.16E-03	3.55E-03
chiP	398	-2.0	0.26	7.38E-15	8.76E-14
araA	240	-1.6	0.32	1.49E-07	8.66E-07
chbB	893	-1.6	0.23	5.74E-12	5.36E-11
leuB	14644	-1.3	0.32	4.26E-05	1.71E-04
srlE	221	-2.6	0.33	2.12E-15	2.62E-14
srlD	364	-2.1	0.29	2.97E-13	3.10E-12
yhfY	131	-1.3	0.29	1.02E-05	4.53E-05
gabP	334	-2.7	0.36	3.21E-14	3.67E-13
sstT	6762	-1.8	0.37	2.95E-07	1.63E-06
atoS	263	-2.2	0.28	4.67E-15	5.60E-14
garD	500	-1.0	0.33	2.49E-03	7.07E-03
yagE	116	-0.8	0.34	1.68E-02	3.89E-02
melA	342	-1.8	0.24	8.21E-14	8.84E-13
araB	171	-1.7	0.34	3.55E-07	1.95E-06
yihX	1963	-2.7	0.26	1.48E-23	3.30E-22
lsrK	1339	-2.2	0.25	2.44E-18	3.82E-17
nanE	274	-0.7	0.28	6.67E-03	1.71E-02
nuoJ	3396	-1.1	0.27	4.00E-05	1.61E-04
ldrD	2118	-1.5	0.31	8.13E-07	4.24E-06
leuA	26150	-2.1	0.46	1.09E-06	5.59E-06
nuoH	5832	-1.3	0.30	5.32E-06	2.45E-05
nuoI	2556	-1.1	0.26	8.14E-05	3.08E-04
yhfZ	226	-1.5	0.27	4.90E-08	3.03E-07
speD	1928	-0.7	0.23	2.33E-03	6.68E-03
ydcS	453	-2.7	0.28	7.08E-21	1.28E-19
ucpA	2741	-1.7	0.25	5.05E-12	4.75E-11
pscG	330	-2.1	0.33	1.18E-10	9.71E-10
ebgC	71	-1.3	0.31	3.12E-05	1.27E-04
agaA	240	-1.8	0.27	7.29E-11	6.14E-10
rayT	288	-1.5	0.24	9.38E-10	6.89E-09
ade	328	-1.8	0.26	4.60E-12	4.36E-11
nanT	529	-0.6	0.26	2.23E-02	4.99E-02
rihA	1613	-1.0	0.34	1.42E-03	4.25E-03
pssA	7278	-1.1	0.20	1.02E-07	6.04E-07
astC	320	-1.9	0.27	5.03E-12	4.75E-11
preT	189	-1.1	0.31	2.33E-04	8.22E-04
cpdA	4402	-1.3	0.22	2.97E-08	1.89E-07
agaW	169	-2.4	0.31	7.90E-15	9.35E-14
xylG	280	-2.0	0.31	1.35E-10	1.10E-09
eutT	76	-1.1	0.29	1.31E-04	4.82E-04

gltK	858	-0.9	0.23	1.03E-04	3.84E-04
yehT	355	-0.8	0.26	2.31E-03	6.63E-03
ygcE	165	-1.8	0.30	1.97E-09	1.43E-08
nuoL	9224	-1.3	0.28	3.61E-06	1.70E-05
pscK	145	-1.9	0.32	1.98E-09	1.44E-08
nuoK	1053	-1.1	0.27	4.49E-05	1.80E-04
ydcW	816	-2.8	0.24	3.79E-31	1.70E-29
dppD	1606	-0.7	0.27	8.30E-03	2.08E-02
nuoN	8736	-1.1	0.25	1.87E-05	7.97E-05
btuB	3586	-1.0	0.28	4.46E-04	1.50E-03
yahN	126	-1.8	0.37	2.66E-07	1.49E-06
ebgA	260	-1.3	0.27	2.78E-06	1.33E-05
ycaM	318	-1.2	0.22	3.18E-07	1.75E-06
yjhB	238	-1.2	0.22	7.34E-08	4.45E-07
ybaE	216	-2.2	0.28	1.53E-14	1.77E-13
paaX	941	-1.6	0.23	1.62E-11	1.44E-10
uidA	301	-1.5	0.28	8.84E-08	5.28E-07
nuoM	5549	-0.7	0.26	5.95E-03	1.54E-02
nrdE	804	-1.6	0.33	1.22E-06	6.20E-06
xylH	239	-2.0	0.32	1.66E-10	1.34E-09
rhaS	186	-1.5	0.27	9.55E-08	5.66E-07
yjhX	73	-1.4	0.33	9.00E-06	4.02E-05
yjhQ	151	-1.3	0.31	2.20E-05	9.24E-05
acnB	109760	-1.3	0.23	7.36E-08	4.45E-07
gltL	1879	-0.7	0.23	2.54E-03	7.19E-03
lrp	9339	-0.6	0.23	2.21E-02	4.96E-02
copA	4896	-2.4	0.24	2.60E-23	5.56E-22
malP	461	-1.4	0.25	2.53E-08	1.63E-07
fucP	94	-2.6	0.33	3.42E-16	4.51E-15
yddH	108	-2.2	0.32	2.69E-12	2.64E-11
dppF	2207	-0.8	0.29	5.19E-03	1.37E-02
eutM	193	-0.7	0.30	2.20E-02	4.93E-02
sgcC	137	-1.5	0.28	2.45E-07	1.38E-06
hupB	18768	-1.7	0.22	3.80E-14	4.28E-13
cdaR	363	-0.6	0.22	5.39E-03	1.42E-02
hisP	1046	-1.1	0.23	3.19E-06	1.51E-05
hisM	511	-0.9	0.26	3.08E-04	1.06E-03
modB	399	-1.3	0.23	2.42E-08	1.57E-07
araG	260	-1.7	0.28	7.21E-10	5.38E-09
bglG	128	-1.7	0.39	5.39E-06	2.48E-05
yajO	1456	-1.6	0.24	3.84E-11	3.29E-10
yidF	195	-0.9	0.25	6.53E-04	2.11E-03
prpC	6502	-3.5	0.37	7.71E-23	1.57E-21
ddpD	223	-1.8	0.23	3.24E-14	3.68E-13

Supplementary Tables

surE	1224	-1.1	0.21	1.66E-07	9.57E-07
yegT	123	-0.8	0.25	2.11E-03	6.09E-03
prpD	10336	-3.6	0.33	6.31E-28	2.06E-26
ygfU	170	-2.8	0.42	3.07E-12	2.99E-11
dkgB	575	-1.4	0.27	3.06E-07	1.69E-06
ychH	5760	-3.0	0.40	1.87E-14	2.16E-13
yhdW	295	-2.9	0.24	8.16E-32	3.89E-30
rhaT	326	-1.6	0.28	2.06E-08	1.34E-07
preA	257	-1.0	0.29	4.89E-04	1.63E-03
fecB	215	-1.5	0.26	4.22E-08	2.64E-07
yagN	1535	-1.0	0.24	6.95E-05	2.67E-04
hisQ	1543	-1.3	0.26	6.36E-07	3.38E-06
eutQ	77	-1.6	0.37	4.97E-06	2.30E-05
prpB	7262	-3.2	0.38	3.19E-17	4.58E-16
allB	185	-0.8	0.26	1.58E-03	4.70E-03
modC	730	-1.4	0.21	1.91E-10	1.52E-09
iclR	904	-0.8	0.25	9.82E-04	3.06E-03
yjhG	380	-1.4	0.23	2.24E-09	1.61E-08
ydiP	385	-1.2	0.22	8.92E-08	5.31E-07
rlmD	887	-1.3	0.23	4.07E-08	2.56E-07
ybeL	1506	-1.9	0.28	7.01E-12	6.49E-11
modA	1283	-1.6	0.26	3.84E-10	2.96E-09
gldA	909	-2.1	0.24	5.43E-18	8.38E-17
ycjD	118	-1.2	0.26	2.24E-06	1.10E-05
ilvC	27601	1.0	0.21	4.98E-06	2.30E-05
yjhH	119	-1.7	0.28	5.44E-10	4.11E-09
yijD	2266	-1.0	0.21	4.79E-06	2.22E-05
yihL	137	-0.7	0.30	1.38E-02	3.25E-02
sgcB	39	-1.8	0.41	5.19E-06	2.39E-05
aes	210	-1.3	0.27	8.37E-07	4.36E-06
ddpB	122	-1.8	0.36	2.92E-07	1.62E-06
ydcV	74	-1.8	0.33	1.51E-08	1.01E-07
relA	4165	-0.9	0.22	4.97E-05	1.97E-04
lsrC	142	-1.9	0.28	9.07E-12	8.31E-11
yeiM	230	-1.0	0.23	2.79E-05	1.15E-04
pykA	6642	-1.0	0.22	2.58E-05	1.07E-04
yihN	164	-1.3	0.27	1.48E-06	7.42E-06
prpE	10097	-3.4	0.31	5.53E-28	1.83E-26
ygcO	37	-1.4	0.36	4.41E-05	1.76E-04
ydcT	135	-1.9	0.30	8.74E-11	7.26E-10
amyA	851	-0.8	0.21	3.11E-04	1.07E-03
fadM	219	-1.1	0.29	8.23E-05	3.11E-04
bax	7218	-0.6	0.25	1.27E-02	3.02E-02
yidE	1164	-0.7	0.22	1.40E-03	4.21E-03

ugpE	90	-0.9	0.27	1.28E-03	3.88E-03
chbA	117	-1.5	0.30	1.66E-07	9.57E-07
ytfT	183	-2.1	0.26	9.06E-16	1.16E-14
pflD	292	-1.5	0.27	3.39E-08	2.16E-07
pmrD	416	-2.1	0.27	2.32E-15	2.86E-14
fhuF	1548	-0.7	0.22	1.77E-03	5.16E-03
dgoR	264	-1.3	0.23	7.48E-08	4.52E-07
flu	946	-1.1	0.27	6.55E-05	2.53E-04
ykfA	381	-0.7	0.23	3.37E-03	9.30E-03
ygfT	225	-1.8	0.31	3.74E-09	2.63E-08
gntP	165	-1.2	0.26	2.77E-06	1.33E-05
alsB	172	-1.5	0.30	2.13E-07	1.21E-06
yjhI	87	-1.6	0.33	6.72E-07	3.55E-06
yjfF	231	-2.0	0.30	1.15E-11	1.04E-10
nrdI	249	-0.9	0.34	5.04E-03	1.33E-02
dcuR	699	-1.6	0.21	5.47E-14	6.02E-13
yahO	517	-1.9	0.26	3.07E-13	3.17E-12
tdcC	192	-1.0	0.28	6.60E-04	2.13E-03
eutD	84	-1.2	0.30	5.44E-05	2.14E-04
ugpC	493	-0.7	0.25	4.08E-03	1.11E-02
fabG	14136	-0.6	0.21	4.80E-03	1.28E-02
yiiF	74	-0.9	0.29	1.18E-03	3.59E-03
fdoI	618	-1.4	0.27	6.87E-07	3.63E-06
fecC	90	-1.6	0.32	2.10E-07	1.20E-06
malQ	722	-0.8	0.23	1.68E-03	4.94E-03
minE	2962	-0.8	0.24	7.02E-04	2.24E-03
cueO	1201	-2.3	0.23	2.97E-22	5.94E-21
ycjG	1766	-1.2	0.27	6.84E-06	3.10E-05
yidJ	107	-1.6	0.29	3.80E-08	2.41E-07
idnD	126	-2.1	0.30	6.87E-13	6.91E-12
yqeA	262	-1.0	0.23	3.05E-05	1.24E-04
frwB	96	-1.6	0.30	5.25E-08	3.23E-07
tam	237	-1.4	0.24	8.82E-09	6.03E-08
glmM	5313	-0.8	0.25	1.15E-03	3.52E-03
ddpC	191	-1.9	0.29	1.66E-11	1.48E-10
yiaO	45	-1.0	0.39	4.33E-03	1.17E-02
dgoK	126	-1.2	0.26	5.16E-06	2.38E-05
srlR	412	-0.5	0.22	1.80E-02	4.13E-02
rfaH	686	-0.6	0.25	1.51E-02	3.52E-02
xseB	798	-1.2	0.21	9.78E-08	5.78E-07
napG	105	-1.5	0.28	4.33E-08	2.70E-07
dinD	163	-1.3	0.26	5.36E-07	2.87E-06
ybiA	87	-1.5	0.29	1.78E-07	1.02E-06
ygjH	45	-0.9	0.33	5.96E-03	1.54E-02

yagH	149	-1.2	0.29	2.47E-05	1.03E-04
sgcE	72	-1.4	0.37	7.71E-05	2.93E-04
mukF	1622	-0.7	0.21	3.48E-03	9.58E-03
idnO	98	-1.6	0.38	7.94E-06	3.57E-05
yghU	3392	-0.9	0.24	1.40E-04	5.12E-04
fdoH	924	-1.1	0.25	1.30E-05	5.72E-05
xylR	475	-0.6	0.23	8.05E-03	2.03E-02
rpoE	11209	-2.2	0.26	1.41E-17	2.08E-16
fecE	126	-1.4	0.26	2.53E-08	1.63E-07
cyoD	4333	-1.1	0.29	6.97E-05	2.67E-04
yabQ	450	-1.1	0.25	3.00E-05	1.22E-04
bolA	2468	-0.8	0.30	4.60E-03	1.23E-02
ykgF	327	-0.9	0.24	4.96E-04	1.65E-03
yeiE	589	-1.2	0.29	2.17E-05	9.17E-05
ddpF	160	-1.5	0.31	1.29E-06	6.49E-06
minD	5599	-0.6	0.21	1.45E-02	3.40E-02
mdh	54829	-1.2	0.22	1.15E-07	6.78E-07
nanM	125	-1.1	0.26	1.72E-05	7.36E-05
yhbT	1809	-1.3	0.23	4.82E-08	2.99E-07
yagG	94	-1.2	0.28	2.03E-05	8.63E-05
ycaC	433	-2.8	0.28	5.72E-23	1.19E-21
potC	473	-0.6	0.23	1.48E-02	3.47E-02
torY	66	-1.6	0.32	1.26E-07	7.41E-07
yeaV	83	-1.5	0.32	1.01E-06	5.19E-06
xanQ	113	-1.3	0.30	1.93E-05	8.22E-05
yhbS	1530	-1.0	0.21	2.98E-06	1.43E-05
gntT	691	1.0	0.22	1.40E-05	6.07E-05
rhaR	118	-1.4	0.29	3.13E-06	1.49E-05
yegU	195	-0.6	0.24	1.21E-02	2.91E-02
ygcP	111	-1.0	0.37	3.14E-03	8.73E-03
yedK	165	-0.8	0.31	5.78E-03	1.51E-02
mak	242	-1.1	0.26	5.89E-05	2.31E-04
ftsW	1204	-0.8	0.22	2.28E-04	8.07E-04
ydhF	1035	-0.6	0.20	3.54E-03	9.72E-03
yeiG	3542	-1.4	0.26	1.64E-07	9.46E-07
curA	368	-1.9	0.27	3.38E-12	3.26E-11
ykgD	107	-1.0	0.31	6.53E-04	2.11E-03
yadI	323	-1.1	0.27	6.29E-05	2.44E-04
eamA	559	-1.0	0.26	1.87E-04	6.70E-04
nanC	68	-3.0	0.42	6.50E-14	7.08E-13
agaS	44	-0.9	0.37	6.84E-03	1.75E-02
dsdC	179	-0.9	0.24	1.87E-04	6.70E-04
rpoS	6322	-1.5	0.23	7.69E-10	5.71E-09
sgcQ	134	-1.3	0.31	2.14E-05	9.06E-05

ilvL	1451	-0.9	0.29	1.55E-03	4.63E-03
yjeI	3305	-1.0	0.20	1.55E-06	7.71E-06
ybjD	914	-0.7	0.26	5.20E-03	1.37E-02
ydbJ	162	-0.7	0.29	2.10E-02	4.73E-02
yhaM	265	-0.6	0.27	2.15E-02	4.83E-02
mmuM	1575	-0.8	0.26	1.65E-03	4.87E-03
yiiD	1450	-0.9	0.27	7.94E-04	2.51E-03
glgA	5456	-1.0	0.25	6.27E-05	2.44E-04
yfcH	2818	-0.6	0.23	1.12E-02	2.71E-02
mukB	10052	-0.8	0.26	4.73E-03	1.26E-02
ldrB	68	-0.9	0.38	8.84E-03	2.20E-02
ybgE	2272	-0.9	0.28	1.36E-03	4.11E-03
dld	9550	-1.3	0.26	2.29E-06	1.11E-05
torZ	317	-1.0	0.26	2.27E-04	8.02E-04
murG	1263	-0.8	0.23	6.09E-04	1.99E-03
pepN	9592	-1.3	0.24	2.97E-07	1.64E-06
ybfN	75	-1.1	0.33	1.02E-03	3.17E-03
ykgE	126	-1.0	0.29	7.59E-04	2.41E-03
malS	221	-1.1	0.30	1.81E-04	6.50E-04
gudP	238	-0.9	0.26	5.22E-04	1.73E-03
guaD	234	-0.6	0.23	1.18E-02	2.83E-02
csiR	393	-1.5	0.24	4.82E-10	3.67E-09
sfsA	1221	-1.2	0.22	6.98E-08	4.24E-07
pcm	1097	-1.0	0.20	1.97E-06	9.65E-06
araD	40	-0.8	0.36	1.79E-02	4.13E-02
ugpA	108	-0.8	0.36	1.88E-02	4.30E-02
mraY	1263	-0.6	0.24	1.13E-02	2.74E-02
csgA	157	-1.3	0.27	3.53E-06	1.67E-05
murR	182	-1.1	0.30	1.72E-04	6.20E-04
ddlB	2603	-1.1	0.33	6.58E-04	2.12E-03
dxs	3347	-0.7	0.21	9.32E-04	2.91E-03
yfbM	41	-1.7	0.39	7.53E-06	3.40E-05
yihS	102	-0.9	0.30	3.35E-03	9.25E-03
uidB	96	-1.2	0.32	1.25E-04	4.59E-04
fecD	48	-1.3	0.33	1.01E-04	3.76E-04
yejF	631	-0.7	0.25	4.14E-03	1.12E-02
yacL	511	-1.2	0.23	1.59E-07	9.19E-07
rtn	779	-0.6	0.22	1.14E-02	2.76E-02
nlpD	14668	-1.3	0.22	1.90E-08	1.24E-07
glgP	8187	-0.9	0.24	1.62E-04	5.88E-04
cydB	7117	-1.0	0.29	5.32E-04	1.76E-03
frwC	93	-1.1	0.32	6.31E-04	2.04E-03
zraS	129	-1.4	0.29	9.35E-07	4.83E-06
yidK	69	-1.3	0.38	2.23E-04	7.90E-04

yihT	43	-1.0	0.36	4.66E-03	1.25E-02
ydeM	89	-1.5	0.31	7.78E-07	4.08E-06
ygdH	4627	-0.7	0.21	2.36E-03	6.73E-03
ynjH	153	-1.2	0.26	7.70E-06	3.47E-05
yfaX	63	-1.9	0.35	9.67E-09	6.57E-08
truD	1857	-0.8	0.22	6.47E-04	2.09E-03
fabA	9645	-1.6	0.27	7.96E-09	5.46E-08
yqiA	812	-0.6	0.21	5.87E-03	1.53E-02
gutM	34	-1.2	0.37	4.49E-04	1.51E-03
cydA	11238	-0.8	0.25	1.20E-03	3.65E-03
paaC	33	-1.0	0.42	8.05E-03	2.03E-02
sgbH	44	-1.4	0.36	3.22E-05	1.31E-04
sgbU	54	-0.9	0.36	6.53E-03	1.68E-02
mdoB	2277	-0.8	0.26	3.26E-03	9.02E-03
hofN	41	-1.0	0.38	5.38E-03	1.42E-02
pflC	94	-1.1	0.33	6.70E-04	2.16E-03
astE	83	-1.1	0.29	8.90E-05	3.33E-04
araH	209	-0.8	0.32	1.34E-02	3.17E-02
yggR	79	-1.4	0.32	9.10E-06	4.06E-05
clpA	30648	-1.0	0.27	4.78E-04	1.60E-03
yjjL	182	-1.0	0.25	8.71E-05	3.28E-04
yqcE	58	-1.9	0.40	4.55E-07	2.46E-06
rseA	20160	-1.5	0.24	3.11E-10	2.42E-09
ybgT	1296	-1.1	0.29	1.16E-04	4.29E-04
yigZ	1093	-0.6	0.25	2.16E-02	4.85E-02
tdcB	74	-1.2	0.38	6.73E-04	2.16E-03
cspC	37457	0.7	0.26	1.11E-02	2.68E-02
gstB	2668	-1.0	0.22	9.77E-06	4.35E-05
yhjD	156	-0.7	0.32	1.44E-02	3.37E-02
ushA	1496	-0.7	0.22	1.88E-03	5.45E-03
sgcA	47	-1.1	0.36	9.57E-04	2.98E-03
artM	871	-0.7	0.23	1.91E-03	5.53E-03
ykgG	199	-1.0	0.31	6.92E-04	2.22E-03
fabD	5283	-0.5	0.22	2.14E-02	4.81E-02
tdcA	63	-1.4	0.38	7.94E-05	3.01E-04
sxy	270	-0.6	0.22	9.66E-03	2.38E-02
uspA	20951	-1.4	0.23	8.99E-09	6.12E-08
selD	5332	-0.9	0.23	2.52E-04	8.85E-04
mazE	170	-1.2	0.27	6.42E-06	2.92E-05
yajD	1170	1.0	0.23	2.71E-05	1.11E-04
sgcR	95	-0.7	0.26	1.22E-02	2.91E-02
ppsR	706	-0.6	0.23	1.16E-02	2.78E-02
melB	165	-1.3	0.27	8.82E-07	4.57E-06
phoU	3260	1.5	0.25	2.14E-09	1.54E-08

yejA	735	-0.6	0.21	9.20E-03	2.28E-02
ydhR	5764	-0.9	0.29	1.58E-03	4.69E-03
ssb	2815	-0.7	0.20	1.03E-03	3.20E-03
bfr	348	-1.6	0.23	1.87E-11	1.65E-10
paaE	51	-1.1	0.32	5.21E-04	1.73E-03
ugpQ	440	-0.6	0.25	1.90E-02	4.34E-02
csgB	27	-1.3	0.57	6.31E-03	1.63E-02
dkgA	512	-1.3	0.28	4.96E-06	2.30E-05
yidL	150	-1.1	0.27	9.56E-05	3.57E-04
yagJ	323	-0.8	0.30	5.83E-03	1.52E-02
yceD	16900	0.8	0.23	4.80E-04	1.60E-03
ykgR	33	-2.0	0.48	4.25E-06	1.99E-05
ybgK	1890	-0.7	0.27	9.09E-03	2.25E-02
yqfA	4045	-3.2	0.31	2.26E-24	5.43E-23
paaB	26	-1.0	0.46	1.25E-02	2.97E-02
sodA	13760	-0.7	0.23	4.97E-03	1.32E-02
paaF	41	-1.5	0.36	1.74E-05	7.43E-05
feaB	615	-0.7	0.21	1.90E-03	5.50E-03
yphF	52	-1.4	0.35	1.86E-05	7.95E-05
ggt	341	-0.8	0.27	2.16E-03	6.22E-03
fumC	9980	-2.1	0.25	3.06E-17	4.40E-16
yheU	145	-0.8	0.36	1.24E-02	2.95E-02
sra	10086	-2.1	0.27	7.92E-15	9.35E-14
ycgB	394	-1.3	0.37	2.09E-04	7.43E-04
alsA	51	-1.7	0.39	2.86E-06	1.37E-05
yahK	367	-0.9	0.22	1.66E-04	5.99E-04
caiF	234	0.8	0.25	1.74E-03	5.09E-03
ybgJ	1291	-0.6	0.26	1.73E-02	3.99E-02
plaP	6650	1.7	0.27	1.08E-10	8.94E-10
yabI	1021	-0.8	0.26	1.81E-03	5.26E-03
yifO	157	-1.2	0.36	4.07E-04	1.38E-03
tktB	709	-1.5	0.24	1.77E-10	1.42E-09
yadS	275	0.9	0.28	1.50E-03	4.48E-03
yjiH	49	-1.4	0.40	1.57E-04	5.69E-04
fixC	50	-1.0	0.35	1.67E-03	4.91E-03
yegE	812	-0.7	0.22	2.68E-03	7.56E-03
rpiB	48	-1.2	0.39	7.42E-04	2.36E-03
ybiB	1464	-1.4	0.28	5.54E-07	2.95E-06
cptB	723	-0.6	0.22	1.64E-02	3.81E-02
pspE	9298	-1.2	0.24	1.55E-06	7.72E-06
dgt	1192	-0.6	0.24	1.80E-02	4.13E-02
hemD	795	-0.7	0.24	4.09E-03	1.11E-02
phnD	91	3.8	0.43	1.48E-19	2.50E-18
yqjA	2145	2.2	0.24	5.44E-19	8.74E-18

phnC	58	4.4	0.55	2.48E-17	3.60E-16
purP	2514	2.2	0.25	2.78E-17	4.01E-16
mzrA	765	1.8	0.24	5.86E-14	6.40E-13
lysA	907	1.9	0.26	1.34E-13	1.43E-12
pstB	2295	2.0	0.28	6.60E-13	6.66E-12
metB	4031	1.8	0.25	1.07E-12	1.07E-11
cysB	2883	1.6	0.24	2.73E-11	2.37E-10
ybbP	1164	1.5	0.23	4.00E-11	3.42E-10
metE	54116	1.4	0.23	9.57E-10	7.02E-09
ybiP	369	1.3	0.23	1.58E-08	1.05E-07
purF	3081	1.3	0.23	1.74E-08	1.15E-07
ftnA	2055	1.7	0.30	1.79E-08	1.18E-07
yjcD	2051	1.8	0.33	1.83E-08	1.20E-07
torC	43	2.3	0.43	2.08E-08	1.35E-07
cvpA	2038	1.5	0.27	2.57E-08	1.64E-07
metL	6112	1.2	0.21	2.79E-08	1.79E-07
ybdH	1035	1.2	0.21	3.77E-08	2.39E-07
ansP	176	1.4	0.26	4.31E-08	2.70E-07
yoaF	362	1.2	0.22	9.23E-08	5.49E-07
pitA	2476	1.4	0.27	1.50E-07	8.72E-07
arnB	214	1.3	0.25	1.57E-07	9.10E-07
ybfP	63	2.1	0.46	7.90E-07	4.14E-06
ilvM	45	1.7	0.35	8.05E-07	4.20E-06
trpC	2026	1.2	0.24	1.06E-06	5.45E-06
mdfA	259	1.1	0.22	1.10E-06	5.62E-06
nudE	2102	1.1	0.22	1.94E-06	9.57E-06
frr	6749	1.1	0.22	2.60E-06	1.26E-05
aroM	259	1.3	0.28	2.62E-06	1.26E-05
ais	32	2.0	0.47	3.38E-06	1.60E-05
glpA	99	1.9	0.45	3.81E-06	1.79E-05
rluB	1532	1.3	0.27	4.33E-06	2.02E-05
arnC	95	1.3	0.30	4.79E-06	2.22E-05
torA	175	1.1	0.23	6.20E-06	2.84E-05
tgt	4261	1.1	0.23	7.20E-06	3.25E-05
yggU	268	1.1	0.23	7.58E-06	3.41E-05
yajR	473	1.0	0.22	1.00E-05	4.46E-05
purT	665	1.2	0.26	1.19E-05	5.25E-05
glpB	99	1.6	0.41	2.17E-05	9.17E-05
purM	2223	1.7	0.44	2.34E-05	9.81E-05
sseB	915	1.1	0.25	2.38E-05	9.94E-05
rho	6733	1.5	0.37	2.58E-05	1.07E-04
rluD	868	1.0	0.23	2.86E-05	1.17E-04
trpB	2245	1.0	0.23	3.21E-05	1.30E-04
rimO	1291	1.1	0.27	3.64E-05	1.47E-04

ydiY	249	1.8	0.47	3.79E-05	1.53E-04
yceJ	88	1.4	0.35	4.36E-05	1.75E-04
proS	7049	1.1	0.27	4.70E-05	1.87E-04
rpmI	26928	1.0	0.25	6.20E-05	2.42E-04
ydbK	2264	1.0	0.25	6.75E-05	2.59E-04
purR	3695	1.3	0.36	9.98E-05	3.72E-04
rplK	12282	1.1	0.29	1.23E-04	4.53E-04
yggT	891	0.9	0.22	1.27E-04	4.69E-04
ybgF	4666	1.0	0.25	1.32E-04	4.83E-04
ilvE	1799	1.0	0.26	1.37E-04	5.02E-04
rpmB	13513	1.3	0.38	1.89E-04	6.77E-04
ygiQ	1926	0.9	0.22	1.92E-04	6.87E-04
udk	640	1.3	0.36	2.01E-04	7.16E-04
purN	960	1.1	0.30	2.01E-04	7.16E-04
yfhL	88	1.5	0.46	2.69E-04	9.42E-04
rsxC	1040	0.9	0.24	2.70E-04	9.43E-04
rplT	49145	1.0	0.26	2.76E-04	9.62E-04
xanP	1515	1.1	0.29	2.94E-04	1.02E-03
trmA	677	1.2	0.35	3.03E-04	1.05E-03
rpmG	11003	1.2	0.35	3.55E-04	1.22E-03
mreD	365	1.0	0.28	3.62E-04	1.24E-03
cld	8100	1.0	0.27	3.72E-04	1.27E-03
cysS	2297	1.0	0.28	3.99E-04	1.36E-03
insN-2	137	1.0	0.30	4.14E-04	1.40E-03
racR	943	0.9	0.25	4.65E-04	1.56E-03
rplQ	27296	1.1	0.31	4.76E-04	1.59E-03
ftnB	1549	0.8	0.21	5.42E-04	1.79E-03
fabF	10940	0.8	0.21	5.69E-04	1.87E-03
yjiK	154	1.1	0.33	5.71E-04	1.88E-03
arnA	338	0.8	0.23	6.14E-04	2.00E-03
mltA	913	0.9	0.24	6.15E-04	2.00E-03
yjgM	407	0.8	0.22	6.16E-04	2.00E-03
parE	1825	0.7	0.20	6.86E-04	2.20E-03
rpmH	2189	0.9	0.27	7.42E-04	2.36E-03
rplA	32726	0.9	0.27	7.59E-04	2.41E-03
prfC	2541	0.8	0.23	7.84E-04	2.48E-03
yieH	337	0.8	0.24	8.40E-04	2.64E-03
hsdM	599	1.0	0.29	8.80E-04	2.75E-03
ampG	937	0.8	0.25	9.84E-04	3.07E-03
yccS	667	0.7	0.20	1.01E-03	3.14E-03
dapD	5784	0.8	0.23	1.08E-03	3.34E-03
folP	521	0.7	0.22	1.11E-03	3.40E-03
suhB	1690	2.1	0.86	1.18E-03	3.59E-03
rlmE	5288	0.8	0.24	1.19E-03	3.64E-03

ydcP	1318	0.7	0.21	1.21E-03	3.68E-03
secG	10540	1.0	0.33	1.24E-03	3.76E-03
ychJ	719	0.9	0.29	1.31E-03	3.97E-03
mreB	7687	0.9	0.30	1.34E-03	4.04E-03
hflX	4601	0.7	0.20	1.39E-03	4.18E-03
rplL	47112	0.8	0.24	1.42E-03	4.25E-03
nusG	5826	1.0	0.31	1.56E-03	4.65E-03
yciA	306	1.0	0.31	1.56E-03	4.65E-03
rcsB	5205	0.8	0.23	1.57E-03	4.66E-03
yacG	271	0.8	0.26	1.62E-03	4.79E-03
ribA	1214	0.8	0.23	1.65E-03	4.86E-03
rlmL	1555	0.7	0.21	1.72E-03	5.05E-03
glnB	1898	1.0	0.34	1.73E-03	5.08E-03
yafK	1323	1.1	0.38	1.79E-03	5.21E-03
mhpB	63	0.9	0.30	1.80E-03	5.24E-03
mreC	2026	0.9	0.31	2.01E-03	5.80E-03
era	2072	0.7	0.21	2.28E-03	6.54E-03
proQ	5636	0.8	0.26	2.34E-03	6.69E-03
yigF	70	1.0	0.34	2.34E-03	6.69E-03
yqaB	834	0.7	0.22	2.41E-03	6.87E-03
yijE	197	0.8	0.24	2.44E-03	6.92E-03
queF	871	0.7	0.21	2.61E-03	7.38E-03
kefA	3925	0.8	0.25	2.69E-03	7.60E-03
rph	1301	0.8	0.26	2.70E-03	7.62E-03
yrdA	825	0.8	0.25	2.71E-03	7.64E-03
yoaK	93	0.8	0.28	2.87E-03	8.04E-03
degS	692	0.8	0.26	2.88E-03	8.08E-03
yigE	38	1.1	0.38	2.92E-03	8.16E-03
rpoA	62702	0.8	0.27	2.92E-03	8.17E-03
sspB	2326	0.7	0.24	2.93E-03	8.18E-03
nhaR	309	0.7	0.23	2.99E-03	8.34E-03
pheT	7659	0.7	0.24	3.08E-03	8.57E-03
aaeB	179	0.8	0.27	3.25E-03	8.99E-03
lpxH	635	1.2	0.48	3.40E-03	9.37E-03
rpmF	15914	0.7	0.24	3.40E-03	9.38E-03
lgt	614	0.7	0.23	4.07E-03	1.11E-02
gntU	99	0.8	0.28	4.12E-03	1.12E-02
yggS	570	0.8	0.29	4.31E-03	1.16E-02
purC	4695	0.8	0.28	4.53E-03	1.22E-02
yciX	261	0.9	0.33	4.60E-03	1.23E-02
adk	5631	1.1	0.46	4.68E-03	1.25E-02
yedV	131	1.0	0.40	4.80E-03	1.28E-02
arnD	118	0.8	0.27	4.82E-03	1.28E-02
ybhK	480	0.7	0.23	4.87E-03	1.29E-02

infB	17186	0.7	0.23	4.92E-03	1.31E-02
rpsT	10089	1.1	0.46	5.09E-03	1.34E-02
abrB	78	0.8	0.30	5.19E-03	1.37E-02
metN	2970	0.9	0.35	5.47E-03	1.44E-02
plsC	642	0.9	0.34	5.59E-03	1.46E-02
evgA	781	0.8	0.31	5.91E-03	1.53E-02
yhfL	38	1.0	0.42	5.92E-03	1.53E-02
tig	32956	1.0	0.39	5.96E-03	1.54E-02
ppiB	4627	1.0	0.39	6.47E-03	1.67E-02
yaiE	3107	0.7	0.25	6.49E-03	1.67E-02
yacF	487	0.7	0.26	6.50E-03	1.67E-02
glpC	82	1.0	0.42	6.52E-03	1.67E-02
purL	7696	0.7	0.24	6.62E-03	1.70E-02
rpsJ	27873	0.8	0.28	6.77E-03	1.73E-02
prmB	2378	0.7	0.24	6.84E-03	1.75E-02
hscB	926	0.8	0.30	7.06E-03	1.80E-02
ycgL	573	0.7	0.25	7.09E-03	1.81E-02
appY	37	0.9	0.37	7.28E-03	1.85E-02
yceA	1053	1.5	0.77	8.13E-03	2.05E-02
yajQ	3783	0.8	0.31	8.77E-03	2.19E-02
recR	1642	1.0	0.43	8.78E-03	2.19E-02
rfbC	103	1.0	0.45	8.84E-03	2.20E-02
pyrH	1069	0.7	0.25	8.85E-03	2.21E-02
yeiW	27	1.1	0.53	8.92E-03	2.22E-02
lolA	1554	0.6	0.22	9.16E-03	2.27E-02
yehR	60	0.9	0.35	9.23E-03	2.28E-02
ydgK	370	0.9	0.39	9.35E-03	2.31E-02
nudG	130	0.7	0.28	9.43E-03	2.33E-02
yedZ	84	0.7	0.28	9.47E-03	2.33E-02
rlmA	442	0.9	0.38	9.66E-03	2.38E-02
mdoG	4483	1.0	0.43	9.72E-03	2.39E-02
rlmB	983	0.6	0.21	9.91E-03	2.43E-02
ppiC	845	0.6	0.22	9.98E-03	2.45E-02
dgkA	635	0.6	0.23	1.01E-02	2.48E-02
rsmD	214	0.7	0.26	1.04E-02	2.55E-02
rpsO	25114	0.7	0.27	1.04E-02	2.55E-02
stpA	180	1.0	0.43	1.07E-02	2.62E-02
rpsD	45643	0.7	0.27	1.08E-02	2.62E-02
trmH	476	0.7	0.25	1.08E-02	2.62E-02
rplU	8442	0.9	0.36	1.08E-02	2.63E-02
rplM	21555	0.7	0.26	1.10E-02	2.67E-02
arsB	78	0.7	0.28	1.13E-02	2.72E-02
secM	1321	0.6	0.22	1.16E-02	2.78E-02
thrT	1243	0.9	0.38	1.17E-02	2.80E-02

lspA	2120	0.6	0.24	1.18E-02	2.83E-02
rpsF	19675	0.7	0.26	1.20E-02	2.88E-02
nirC	47	0.8	0.35	1.20E-02	2.88E-02
dusB	7399	0.8	0.34	1.25E-02	2.99E-02
yeiS	95	0.8	0.33	1.26E-02	2.99E-02
thrL	279	0.6	0.23	1.26E-02	2.99E-02
secE	2570	0.7	0.31	1.32E-02	3.14E-02
clpX	8160	0.6	0.23	1.34E-02	3.17E-02
ypjM_3	124	0.7	0.31	1.34E-02	3.18E-02
hybE	196	0.6	0.26	1.38E-02	3.27E-02
torD	71	0.7	0.29	1.39E-02	3.28E-02
rluC	808	0.9	0.38	1.41E-02	3.31E-02
prfB	5757	0.7	0.29	1.42E-02	3.34E-02
yjaA	25	0.9	0.43	1.49E-02	3.50E-02
ptsH	14080	0.8	0.34	1.50E-02	3.50E-02
tolA	2461	0.6	0.24	1.55E-02	3.62E-02
bcp	3845	0.8	0.33	1.58E-02	3.67E-02
yfjW	308	0.9	0.43	1.59E-02	3.69E-02
ilvD	1547	0.6	0.26	1.60E-02	3.72E-02
ygdD	278	0.6	0.24	1.68E-02	3.89E-02
rpmA	17136	0.7	0.29	1.78E-02	4.11E-02
yafJ	1030	0.7	0.32	1.80E-02	4.13E-02
rlmI	1029	0.6	0.26	1.82E-02	4.18E-02
rplC	46089	0.7	0.29	1.83E-02	4.19E-02
srmB	1177	0.7	0.30	1.87E-02	4.27E-02
nudC	470	0.5	0.22	1.94E-02	4.42E-02
ebgR	309	0.9	0.44	1.95E-02	4.44E-02
pal	15060	0.6	0.24	1.95E-02	4.44E-02
yhdE	723	0.5	0.21	1.98E-02	4.50E-02
cyaY	485	0.8	0.35	2.00E-02	4.53E-02
rpsP	8493	0.6	0.26	2.01E-02	4.54E-02
rpsM	29434	0.6	0.26	2.03E-02	4.59E-02
mcrB	292	0.6	0.27	2.04E-02	4.60E-02
yhdJ	82	0.7	0.32	2.17E-02	4.87E-02
dacB	428	0.7	0.30	2.23E-02	4.99E-02
gloA	1076	0.6	0.25	2.24E-02	5.00E-02
phoH	6054	-1.7	0.27	2.03E-10	1.61E-09
yjgB	177	-1.6	0.25	5.12E-10	3.88E-09
fabB	8758	-1.6	0.26	6.78E-10	5.08E-09
qor	1526	-1.4	0.25	1.25E-08	8.39E-08
ykgC	95	-1.6	0.29	1.84E-08	1.21E-07
adhP	508	-1.3	0.24	8.72E-08	5.21E-07
yccJ	354	-1.4	0.26	2.65E-07	1.48E-06
gadE	56	-1.6	0.33	4.45E-07	2.41E-06

wrbA	222	-1.4	0.27	6.42E-07	3.40E-06
cdd	707	-1.2	0.24	1.50E-06	7.49E-06
yjdC	928	-1.2	0.24	1.92E-06	9.48E-06
moaE	580	-1.3	0.28	2.34E-06	1.14E-05
sulA	268	-1.2	0.26	2.73E-06	1.32E-05
elaB	1230	-1.1	0.23	2.83E-06	1.36E-05
kbl	1036	-1.1	0.23	4.08E-06	1.91E-05
acnA	5220	-1.1	0.22	4.89E-06	2.27E-05
moaB	1059	-1.1	0.25	6.27E-06	2.86E-05
ydjK	64	-1.5	0.35	1.05E-05	4.66E-05
dmlA	453	-1.1	0.24	1.33E-05	5.82E-05
moaC	529	-1.2	0.27	1.68E-05	7.23E-05
yahD	97	-1.3	0.31	1.71E-05	7.34E-05
yebV	361	-1.2	0.29	2.46E-05	1.02E-04
yncL	55	-1.3	0.35	7.63E-05	2.90E-04
hpf	4686	-0.8	0.20	8.58E-05	3.23E-04
rbbA	2001	-1.1	0.28	1.24E-04	4.59E-04
tdh	1389	-1.0	0.25	1.30E-04	4.78E-04
yeaH	281	-0.9	0.23	1.43E-04	5.24E-04
sgbE	84	-1.1	0.29	1.57E-04	5.70E-04
yieL	76	-1.2	0.33	2.01E-04	7.16E-04
ycaI	315	-1.0	0.27	2.29E-04	8.09E-04
moaD	135	-1.1	0.32	3.02E-04	1.04E-03
sbmC	558	-0.8	0.22	3.05E-04	1.05E-03
yigI	681	-0.9	0.24	3.55E-04	1.22E-03
yghA	88	-1.1	0.31	3.72E-04	1.27E-03
yccU	734	-0.8	0.20	3.78E-04	1.29E-03
ssrA	2677459	-1.0	0.29	4.65E-04	1.56E-03
icd	95905	-0.8	0.24	6.80E-04	2.19E-03
bglB	69	-1.0	0.30	8.63E-04	2.71E-03
entB	134	-0.9	0.27	1.08E-03	3.33E-03
zraR	188	-0.9	0.28	1.09E-03	3.35E-03
yegP	169	-0.9	0.29	1.12E-03	3.43E-03
ampE	651	-0.8	0.22	1.13E-03	3.47E-03
frvB	59	-1.1	0.38	1.16E-03	3.55E-03
smf	342	-0.9	0.26	1.25E-03	3.79E-03
xapA	49	-1.1	0.36	1.27E-03	3.85E-03
bssS	3166	-1.0	0.33	1.27E-03	3.85E-03
sodC	476	-0.7	0.20	1.57E-03	4.68E-03
ymdF	151	-0.9	0.27	1.63E-03	4.83E-03
yjjU	429	-0.8	0.27	1.71E-03	5.04E-03
yfcG	54	-1.1	0.39	1.76E-03	5.14E-03
bglJ	51	-1.1	0.36	1.81E-03	5.26E-03
kefC	444	-0.8	0.25	1.89E-03	5.50E-03

yjbQ	260	-0.7	0.23	1.97E-03	5.70E-03
matB	84	-1.0	0.32	2.24E-03	6.45E-03
ygaM	271	-0.9	0.28	2.41E-03	6.85E-03
yjbJ	176	-0.9	0.31	2.42E-03	6.87E-03
bglH	66	-1.0	0.35	2.42E-03	6.88E-03
ydcJ	583	-0.9	0.31	3.04E-03	8.46E-03
mipA	3803	-0.8	0.27	3.19E-03	8.84E-03
adiA	118	-0.7	0.25	4.27E-03	1.15E-02
yjjV	306	-0.7	0.26	4.48E-03	1.21E-02
yphG	149	-0.8	0.28	4.57E-03	1.23E-02
artI	2749	-0.6	0.20	4.57E-03	1.23E-02
yeaG	1213	-0.8	0.29	4.76E-03	1.27E-02
yihQ	56	-0.9	0.32	4.76E-03	1.27E-02
sgrT	234	-0.8	0.31	5.42E-03	1.43E-02
paaD	30	-1.1	0.46	5.58E-03	1.46E-02
yahF	72	-0.9	0.37	5.63E-03	1.47E-02
msyB	193	-0.7	0.25	5.79E-03	1.51E-02
lhr	1127	-0.6	0.22	5.84E-03	1.52E-02
msrA	903	-0.6	0.22	5.90E-03	1.53E-02
aslB	249	-0.7	0.24	5.96E-03	1.54E-02
talA	221	-0.7	0.26	7.25E-03	1.85E-02
yicO	54	-0.8	0.33	7.57E-03	1.92E-02
gpmA	13296	-0.6	0.21	7.89E-03	2.00E-02
nanS	40	-1.0	0.41	8.09E-03	2.04E-02
narY	107	-0.8	0.32	8.30E-03	2.08E-02
ydjJ	52	-0.9	0.38	9.05E-03	2.25E-02
nagA	1042	-0.6	0.21	9.19E-03	2.28E-02
paaZ	69	-0.8	0.31	9.24E-03	2.28E-02
ygeR	698	-0.5	0.20	1.01E-02	2.48E-02
lpoB	936	-0.6	0.23	1.02E-02	2.49E-02
ytfN	1449	-0.7	0.26	1.03E-02	2.51E-02
ylbH	92	-0.9	0.36	1.08E-02	2.62E-02
metC	3244	-0.6	0.23	1.10E-02	2.67E-02
yieF	1471	-0.6	0.22	1.13E-02	2.72E-02
xylB	186	-0.7	0.27	1.23E-02	2.93E-02
Nin221_2	80	-0.7	0.29	1.35E-02	3.19E-02
ydcU	75	-0.8	0.33	1.45E-02	3.39E-02
fpr	1463	-0.6	0.24	1.56E-02	3.65E-02
yhdX	64	-0.7	0.31	1.58E-02	3.67E-02
zitB	373	-0.7	0.29	1.76E-02	4.06E-02
paaH	72	-0.7	0.32	1.88E-02	4.30E-02
higB	23	-0.9	0.45	1.90E-02	4.34E-02
yhhI	25	-0.9	0.44	1.96E-02	4.45E-02
hemC	2578	-0.6	0.23	2.06E-02	4.65E-02

Supplementary Tables

allD	34	-0.8	0.42	2.16E-02	4.86E-02
bglA	2490	-0.6	0.23	2.21E-02	4.96E-02
yjdN	34	-0.8	0.41	2.23E-02	4.99E-02
